# Consecutive Ligand‐Based Electron Transfer in New Molecular Copper‐Based Water Oxidation Catalysts

**DOI:** 10.1002/anie.202104020

**Published:** 2021-07-16

**Authors:** Marcos Gil‐Sepulcre, Pablo Garrido‐Barros, Jan Oldengott, Ignacio Funes‐Ardoiz, Roger Bofill, Xavier Sala, Jordi Benet‐Buchholz, Antoni Llobet

**Affiliations:** ^1^ Institute of Chemical Research of Catalonia (ICIQ) Barcelona Institute of Science and Technology (BIST) Av. Països Catalans 16 43007 Tarragona Spain; ^2^ Departament de Química Universitat Autònoma de Barcelona Cerdanyola del Valles 08193 Barcelona Spain; ^3^ Departamento de Química Centro de Investigación en Síntesis Química (CISQ) Universitad de La Rioja 26006 Logroño Spain

**Keywords:** first-row transition metal complexes, reaction mechanisms, redox non-innocent ligand, water oxidation catalysis, water splitting

## Abstract

Water oxidation to dioxygen is one of the key reactions that need to be mastered for the design of practical devices based on water splitting with sunlight. In this context, water oxidation catalysts based on first‐row transition metal complexes are highly desirable due to their low cost and their synthetic versatility and tunability through rational ligand design. A new family of dianionic bpy‐amidate ligands of general formula H_2_LN^*n*−^ (LN is [2,2′‐bipyridine]‐6,6′‐dicarboxamide) substituted with phenyl or naphthyl redox non‐innocent moieties is described. A detailed electrochemical analysis of **[(L4)Cu]^2−^** (L4=4,4′‐(([2,2′‐bipyridine]‐6,6′‐dicarbonyl)bis(azanediyl))dibenzenesulfonate) at pH 11.6 shows the presence of a large electrocatalytic wave for water oxidation catalysis at an η=830 mV. Combined experimental and computational evidence, support an all ligand‐based process with redox events taking place at the aryl‐amide groups and at the hydroxido ligands.

## Graphical abstract

A family of Cu‐based complexes bearing dianionic bpy‐amidate ligands have been prepared and characterized by spectroscopic and electrochemical techniques. The new complexes are capable of performing water oxidation catalysis at overpotentials between 557–830 mV pH 11.6. A detailed electrocatalytic study in combination with computational calculations revealed the all ligand‐based nature of the electron transfer processes involved in the catalytic cycle.

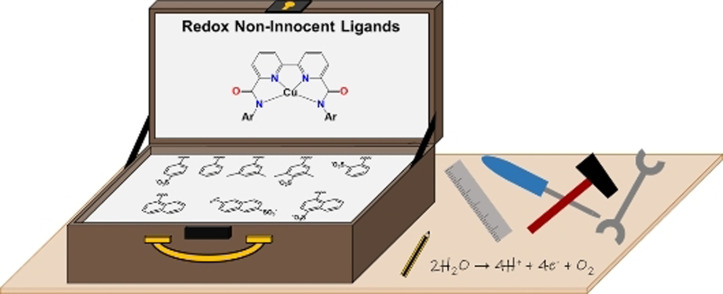

## Introduction

Molecular water oxidation catalysis driven by transition metal complexes[^1^, ^2^, ^3^, ^4^] is an active field of research at present due to its implication in the generation of solar fuels.[^5^, ^6^, ^7^, ^8^] The molecular approach offers the possibility of a thorough mechanistic analysis, including the isolation and spectroscopic characterization of relevant reaction intermediates.^[9]^ Further, the molecular toolkit provides an ample variety of ligands that—if properly used—can help in the rational design of extremely efficient and oxidatively rugged molecular water oxidation catalysts (WOCs).[^10^, ^11^] At present, there is a large body of WOCs based on Ru complexes with very good performances,[^12^, ^13^, ^14^] and a few based on first raw transition metals.[^15^, ^16^, ^17^, ^18^, ^19^, ^20^]

Most of the WOCs described today achieve reactive species using metal‐based Proton Couple Electron Transfer (PCET) processes from M‐OH_*x*_ (*x*=0–2) groups.^[21]^ A new body of transition metal complexes capable of oxidizing water to dioxygen is starting to emerge, where redox non‐innocent ligands play a key role.[^22^, ^23^, ^24^, ^25^, ^26^, ^27^] This is particularly important for first‐row transition metal complexes where the energy difference between different oxidation states is generally much wider than for instance second‐row transition metals such as Ru.[^9^, ^10^, ^28^, ^29^] The achievement of highly reactive species by a combination of metal‐ and ligand‐based electron transfer processes is also a strategy followed by nature to achieve highly reactive species at relatively low energies for oxidative transformations, as is the case of Cyt‐P450.[^30^, ^31^, ^32^]

We have recently shown that for the Cu‐amidate complex containing a redox active ligand (N1,N1′‐(1,2‐phenylene)bis(N2‐methyloxalamide)), its overpotential (*η*) for the catalytic water oxidation reaction could be reduced by more than 500 mV, simply changing the electron donating nature of the organic substituents on the parent ligand.^[17]^

**Figure 1:**
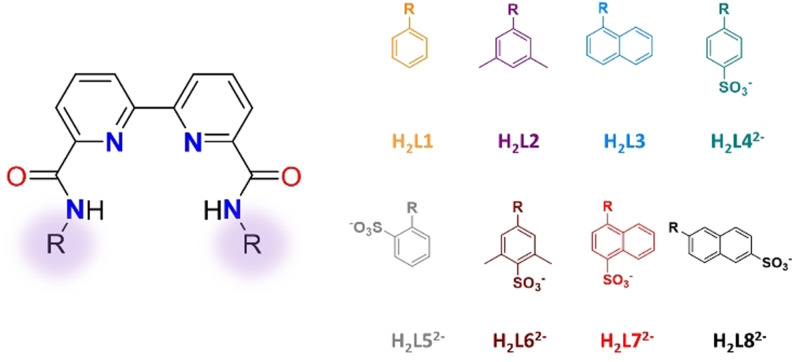
Drawings of H_2_LN^*n*−^ (N=1–3, *n*=0; N=4–8; *n*=2) bpy‐amidate ligands used in this work.

In the present work, we take a step forward and we present a family of molecular WOCs based on Cu complexes where all the electron transfer processes involved in the catalytic cycle are ligand‐based only. This strategy is attractive because there is a huge variety of ligands that can be used and therefore it can provide the means for an exquisite control of the kinetics and thermodynamics involved in water oxidation catalysis. Here on, we describe the synthesis, spectroscopic, redox properties and the water oxidation mechanism of a family of Cu complexes, **[(LN)Cu^II^]** (N=1–3) and **[(LN)Cu^II^]^2−^** (N=4–8), containing a dianonic bpy‐amidate scaffold [2,2′‐bipyridine]‐6,6′‐dicarboxamide (LN) with a variety of aromatic substituents (neutral and anionic) as depicted in Figure 1, that can fine tune the water oxidation properties of the catalyst.

## Results and Discussion

### Synthesis and Structure

**Figure 2:**
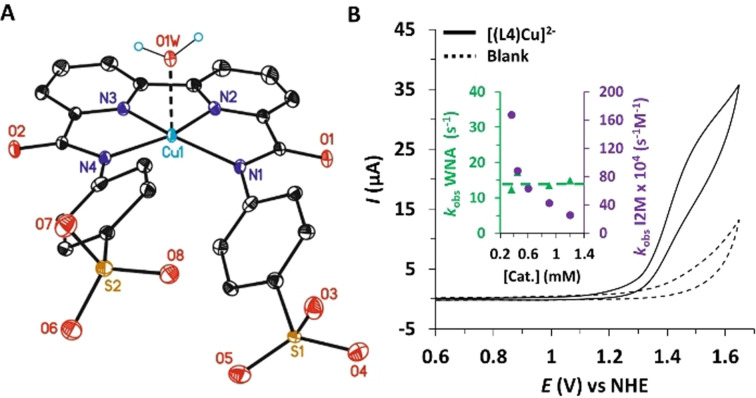
A) ORTEP drawing of **[(L4)Cu]^2−^** at 50 % probability level. The solvent molecules, sodium and hydrogen atoms have been omitted for clarity. Color code: C, black; N, blue; O, red; S, yellow; Cu, light blue. B) Cyclic Voltammogram of 1 mM solution of **[(L4)Cu]^2−^** in 0.1 M phosphate buffer at pH 11.6 at scan rate of 100 mV s^−1^. Inset: Plot of *k*_obs_ vs. **[(L4)Cu]^2−^** calculated by FOWA analysis assuming a WNA mechanism (green trace) or an I2M mechanism (purple trace).

The synthesis of a family of ligands H_2_LN^*n*−^ (N=1–3, *n*=0; N=4–8; *n*=2; see Figure 1) and their Cu^II^ complexes is described in detail, together with their characterization based on NMR (Figures S1–S9), UV/Vis (Figures S29,S30), Electron Paramagnetic Resonance (EPR, Figure S38), MS (Figures S11–S26) and EA that is reported in the Supporting Information (SI). The Zn^II^ analogue with the H_2_L4^2−^ deprotonated ligand, **[(L4)Zn]^2−^**, has also been prepared in a similar manner and its synthesis and characterization is reported in the Supporting Information (see Experimental Section and Figure S10). In this family of ligands, the aromatic delocalization along the bipyridyl‐di‐amidate groups places their four coordinating N‐atoms in a nearly planar manner, so that all their Cu^II^ complexes obtained display a distorted square planar geometry, as can be seen in the X‐ray crystal structure for **[(L1‐L7)Cu]** ^***n*****−**^ (*n*=0 or 2), whose ORTEP are drawn in Figure 2 A and Figures S31–S37.^[33]^ In addition, they also display a contact in the apical position with an oxygen atom coming from a H_2_O or MeOH molecule, or a sulfonate from a neighbor complex unit (the Cu–O distances span between 2.33–2.61 Å). The Cu−N bond lengths range from 1.94 to 2.01 Å, similar to other previously reported tetraamidate copper complexes.[^17^, ^22^] Whereas the Cu–N_bpy_ bond lengths fall within 1.94 to 1.97 Å, the Cu–N_amide_ bonds are slightly larger, 1.96 to 2.01 Å. (Table S2). The *cis* N‐Cu‐N angles are within the expected range of 118.4° to 120.8° for N_amide_‐Cu‐N_amide_ and 77.4° to 78.5° for N_bpy_‐Cu‐N_bpy_ (Table S3).

### Redox Properties and Water Oxidation Catalysis

The redox properties of the ligands and complexes described have been investigated in aqueous solution by using Cyclic Voltammetry (CV), Differential Pulse Voltammetry (DPV) and Controlled Potential Electrolysis (CPE) experiments.

The **[(L4)Cu]^2−^** complex is selected as the model for initial evaluation of the electrochemical properties and catalytic performances with regard to water oxidation catalysis because of its higher solubility in aqueous solutions thanks to the sulfonate group when compared to their analogues with no sulfonate, **[(L1–L3)Cu]**.

In aqueous solution at pH 11.6 the CV of complex **[(L4)Cu]^2−^** shows only a large electrocatalytic wave with its onset potential at approx. 1.3 V (Figure 2 B and Figures S42–S44), which is related to the electrocatalytic oxidation of water to dioxygen. The DPV shows a wave at 1.35 V (*η*=830 mV) that can be associated with the redox couple responsible for the catalytic activity.

The generation of dioxygen was confirmed by a CPE experiment where a gas phase Clark‐type electrode had been placed at the headspace of the working compartment (Figure S50). A Faradaic efficiency (FE) of 76 % was obtained for **[(L4)Cu]^2−^**. No Cu‐based heterogeneous oxide species (CuOx) were detected at the electrode as ascertained electrochemically by CV when placing the working electrode in a clean electrolyte solution after the O_2_ detection experiment (Figure S50B) and neither by Scanning Electron Microscopy (SEM) or Energy Dispersive X‐ray (EDX) spectroscopy measurements of the electrode (Figures S54A and S55A). The relatively low FE can be associated with the potential catalyst degradation via hydroxylation of the aromatic ring, specially at the cationic radical stage, and further oxidative pathways when the phenolate group is generated.[^14^, ^34^, ^35^]

A kinetic analysis of the electrocatalytic wave based on the Foot‐of‐the‐wave analysis (FOWA)[^36^, ^37^, ^38^] methodology was carried out (see Figure S48 and SI for details). A TOF_max_ of 10.5 s^−1^ was obtained for **[(L4)Cu]^2−^**, which is within the range observed for previous Cu‐based catalysts at basic pH.[^17^, ^22^, ^39^, ^40^] Further, first order behavior with regard to the concentration of **[(L4)Cu]^2−^** was also obtained that points to a hydroxide nucleophilic attack, mimicking traditional water nucleophilic attack (WNA) mechanism and discarding the interaction of two MO units (I2M mechanism), as can be observed in the inset of Figure 2 B. This is also consistent with the mechanistic scenario proposed for previous Cu WOCs containing redox non‐innocent ligands.^[29]^

**Figure 3:**
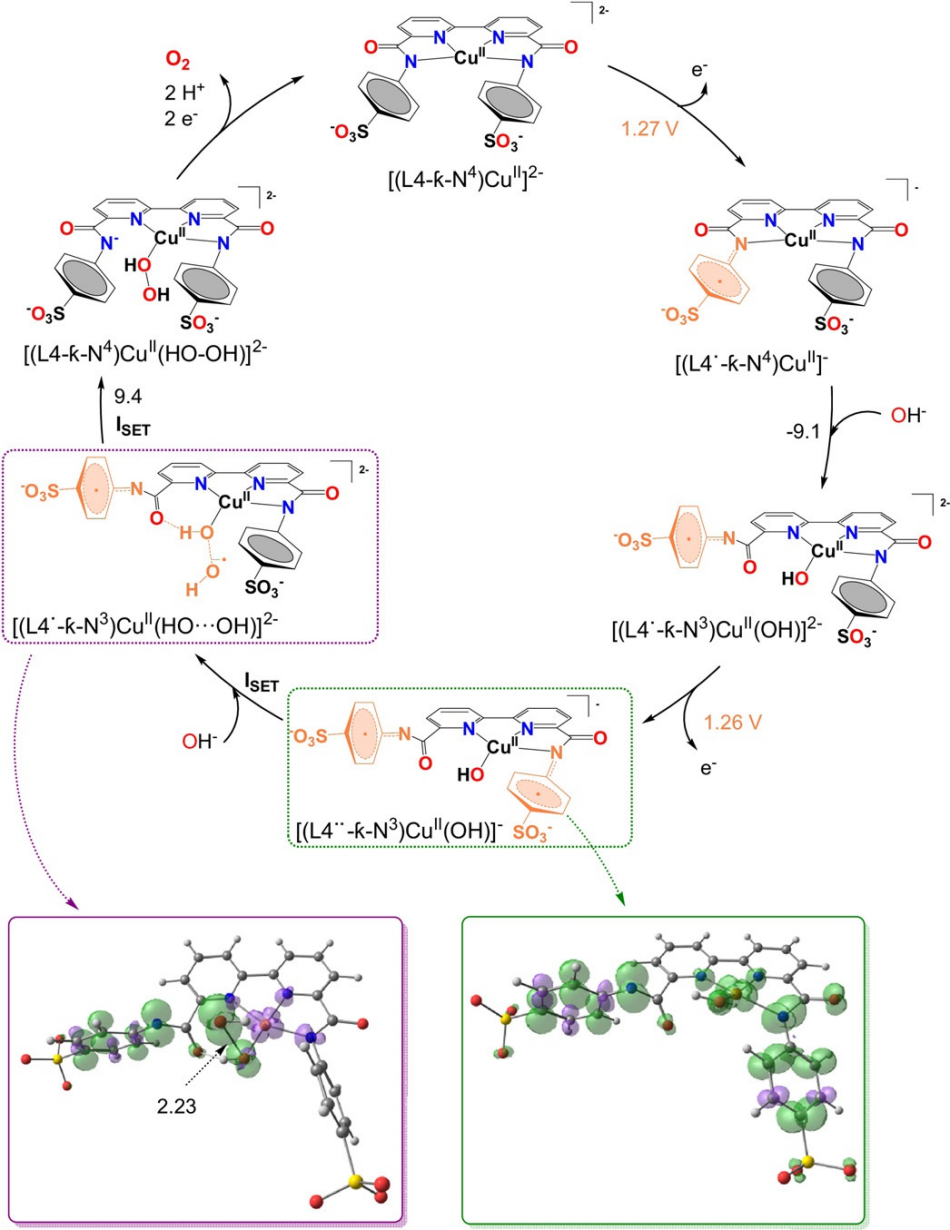
Catalytic cycle for **[(L4)Cu]^2−^** ([(L4‐κ‐N^4^)Cu^II^]^2−^) via the SET‐WNA mechanism. Free energy changes are indicated in black in kcal mol^−1^ and the calculated redox potentials are shown in orange in V vs. NHE. Spin density distribution is shown for selected intermediates. Bond lengths are indicated in Angstroms. The radical character of the ligands is indicated in the formula.

A computational analysis by means of DFT calculations using B3LYP‐D3 in combination with the SMD solvation model was carried out to further characterize the catalytic cycle to support and complement available experimental data. The main results obtained for the [(L4‐κ‐N^4^)Cu^II^]^2−^ catalyst are summarized in Figure 3 and described in detail in the SI. Note that in the DFT analysis the calculated species involved in the catalytic cycle contain the L4 ligand bonded in different coordination modes. Therefore, the number and nature of the atoms bonded to the metal center will be indicated from now on in the corresponding formula. Also, the ligands based radical species will also be indicated with a dot.

The calculated structure for complex [(L4‐κ‐N^4^)Cu^II^]^2−^ at pH 11.6 is similar to the one obtained by X‐ray diffraction analysis (see Table S4 and Figure 2 A; S56). Upon one electron removal, the most stable oxidized species consists of a triplet Cu^II^ complex with a radical cation mainly centered on one of the aryl‐amidate moieties, as shown in Figures S57 and S58, [(L4^.^‐κ‐N^4^)Cu^II^]^−^. The calculated oxidation potential of 1.27 V is in good agreement with the experimental value of 1.37 V obtained from DPV (Table S5). As expected, this ligand oxidation causes a decrease in the electron‐donating character of the amidate group that is reflected in a significant increase in the Cu–N_amide_ distance from 2.05 Å to 2.14 Å, and thus weakening the Cu−N bond.

The next step involves the coordination of an ‐OH ligand at the equatorial position, replacing the just described elongated Cu‐amidate bond, thus generating [(L4^.^‐κ‐N^3^)Cu^II^(OH)]^2−^, which is exergonic by 9.1 kcal mol^−1^. A second oxidation occurs now at 1.26 V giving rise to the formation of a diradical, [(L4^..^‐κ‐N^3^)Cu^II^(OH)]^−^. The similarity of the two redox potentials reflects the relative independence of the amidate fragments within the complex with no or very small electronic interaction, and is consistent with the absence of precatalytic waves. The doubly oxidized quartet species [(L4^..^‐κ‐N^3^)Cu^II^(OH)]^−^ features again an increased Cu–N_amide_ distance, from 2.07 Å to 2.21 Å. In this case, its substitution by a second incoming hydroxo group to form [(L4^..^‐κ‐N^2^)Cu^II^(OH)_2_]^2−^ is exergonic by 2.7 kcal mol^−1^. Electronically, both doubly oxidized structures are quartets, presenting an unpaired electron on the Cu^II^ center and two other unpaired electrons on each aryl‐amidate fragment. We have considered both mono‐ and di‐hydroxido doubly oxidized species as possible intermediates for the O−O bond formation, since both of them give similar energy penalties. The monohydroxido mechanism is described below whereas the dihydroxido species is reported in the SI (Figures S61–S63).

Approaching an OH^−^ group to [(L4^..^‐κ‐N^3^)Cu^II^(OH)]‐ at distances as long as 4 Å already promotes a single electron transfer (SET) process, reducing one of the aryl‐amidate groups (SET‐WNA; Figure S60), similar to the previously described [Cu(L)(OH_2_)_2_] (L=6,6′‐dihydroxy‐2,2′‐bipyridine) catalyst.^[39]^ This highly favored electron transfer is likely a result of a low energy reorganization process and thus precludes the acquisition of a transition state. This new electronic structure evolves to an intermediate [(L4^.^‐κ‐N^3^)Cu^II^(HO⋅⋅⋅OH^.^)]^2−^ where both oxygen atoms form a two center‐three electron bond (2c‐3e^−^) with a formal order of 0.5 and a bond length of 2.23 Å (Figure 3 and Figure S62). Further decreasing the distance between the two oxygen centers fosters a second single electron transfer that reduces the second phenyl‐amidate moiety concomitant with the formation of a neutral hydroperoxo species bonded to the Cu center, [(L4‐κ‐N^3^)Cu^II^(HOOH)]^2−^, where the Cu center has doublet electronic state. The initial quartet species are connected by a minimum energy crossing point (MECP).^[41]^ A 9.4 kcal mol^−1^ energy barrier is obtained for this second electron transfer as estimated from the potential energy relaxed scan (Figure 3 and Figure S60). DFT calculation of the OH^−^ using the experimental value for the energy of solvation (−102.8 kcal mol^−1^)^[42]^ suggests a likely underestimation of this value using SMD, which in turn would be reflected in a lower activation energy for the O−O bond formation (see Figure S59).

The all ligand‐based ET process suggested by DFT is in agreement with the need of highly anionic/σ‐and π‐donating ligand environment coordinated to the Cu metal center, in order to be able to observe the III/II redox couple at relatively low potentials. For instance the [Cu^II^‐OPBAN]^2−^ (OPBAN is N1,N1′‐(1,2‐phenylene)bis(N2‐methyl‐oxalamide), where the OPBAN ligand provided four negatives charges directly bonded to the metal center, has a Cu^III/II^ couple at 0.56 V at pH 11.5. In the present case the LN ligands are bonded to the metal center by two neutral N of the bpy group and two anionic N of the amide groups, thus effectively act as a dianonic ligand to the metal center. For Ru complexes, where a large number of complexes have been electrochemically characterized, the effect of exchanging an anionic ligand by a neutral one increases the redox potential by roughly 400–500 mV per anionic group.[^43^, ^44^]

Thus, in the present case the LN ligands will not have sufficient electron‐donation capacity to stabilize the Cu^III/II^ couple below or at the potential where catalysis is occurring.[^17^, ^45^, ^46^] An additional evidence for the ligand based process is provided by the electrochemical properties of the homologue Zn complex, **[(L4)Zn]^2−^**, where no metal based ET can occur. A CV of **[(L4)Zn]^2−^** (see Figure S41) under the same conditions as **[(L4)Cu]^2−^** shows a ligand‐based wave (non‐catalytic) at the same potential where the Cu homologue shows catalytic activity.

Finally, a compelling evidence for the all ligand‐based ET in the water oxidation catalysis is provided by the correlation between the substituent effect on the amidate ligand in the potential observed for water oxidation catalysis, that is described in the next section.

### Substituent Effects

**Figure 4:**
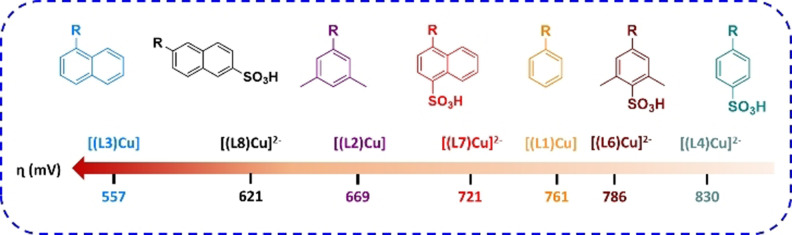
Influence of the substituents in [(LN)Cu]^*n*−^ (*n*=0 or 2) complexes on the *η* (mV) of the water oxidation reaction.

As described above, both electrochemical experiments and DFT calculations clearly point out that the O−O bond formation and thus water oxidation catalysis that occurs using **[(L4)Cu]^2−^** is an all ligand‐based process where the Cu center remains at oxidation state II. Hence, the tuning of the redox active aryl group should have a strong impact into the catalytic properties. Due to the high modularity of the ligand synthesis, the family of complexes indicated in Figure 4 constitutes an excellent platform to test how electronic perturbations exerted by electron‐donating and electron‐withdrawing groups as well as by π‐extended systems such as naphthyls influence the electrocatalytic water oxidation performance.

**Figure 5:**
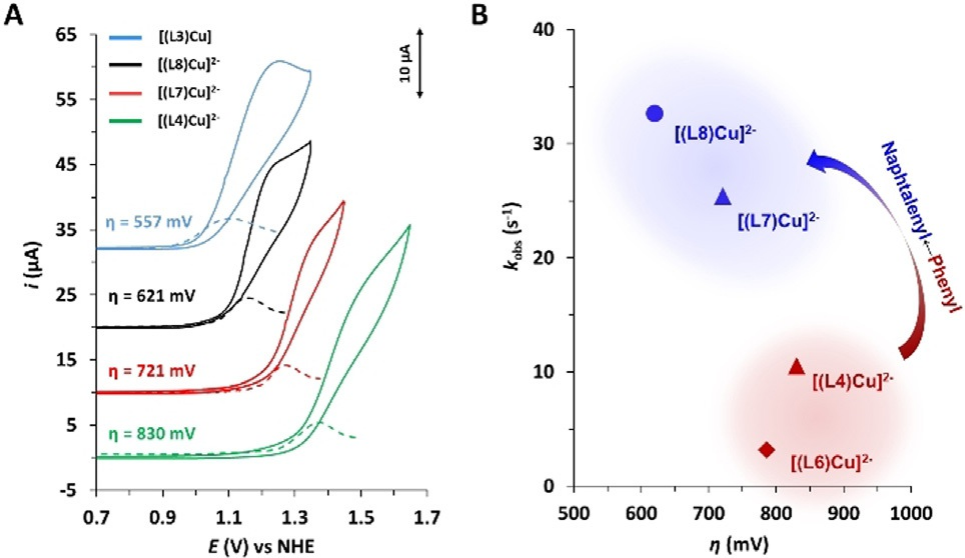
A) CVs of a 1 mM solution of **[(L3)Cu]** (blue line), **[(L4)Cu]^2−^** (green line), **[(L7)Cu]^2−^** (red line) and **[(L8)Cu]^2−^** (black line) in 0.1 M phosphate buffer at pH 11.6. *Note*: In case of complex **[(L3)Cu]** the experiments were performed in a mixture of 0.1 M phosphate buffer/TFE (6:4) for solubility reasons. B) plot of *k*_obs_ (s^−1^) for the water oxidation catalysis vs. *η* (mV) for selected cases.

The CV of complexes **[(L1‐L8)Cu]** ^***n*****−**^ (except **[(L5)Cu]^2−^**) recorded at pH 11.6 are similar to that of **[(L4)Cu]^2−^**, showing an electrocatalytic wave at anodic potentials associated with the water oxidations catalysis (Figures S45 and S47). However, the substituent clearly influences the overpotential at which catalysis occurs, as can be seen in Figure 4 and Figure 5 A (also in Figures S45–S47). Within the family of phenyl substituents (H_2_LN^*n*−^, where N=1, 2, 4 and 6) electron‐donor moieties decrease the overpotential (*η*) whereas electron‐withdrawing groups increase it, as expected from phenyl‐centered oxidations. Similarly, the same trend occurs for the naphthyl substituted cases. In addition, the π‐delocalization (phenyl vs. naphthyl) results in a significant decrease of *η*, as expected.^[47]^

It is interesting to see that all complexes **[(L1‐L8)Cu]** ^***n*****−**^ (*n*=0 or 2−) display a catalytic wave in the range of 1.0–1.6 V, except for **[(L5)Cu]^2−^**, that basically shows no activity (Figures S45 and S51). This can be attributed to the coordination ability of the sulfonate group situated in the ortho position, which has the right spatial disposition to generate a weak coordination bond with the Cu center, as can be seen in its crystal structure (Figure S35). This contact can partially disfavor the OH^−^ coordination needed for catalysis by stabilizing the Cu–N_amide_ bond (Figure S64).

A kinetic analysis of the electrocatalytic performance of catalysts **[(LN)Cu]^n−^** was undertaken based on FOWA. Complexes **[(L4)Cu]^2−^**, **[(L6)Cu]^2−^**, **[(L7)Cu]^2−^** and **[(L8)Cu]^2−^** were analyzed in aqueous solution at pH 11.6 whereas for complexes **[(L1‐L3)Cu]** we used a mixture of 40 % TFE and 60 % aqueous pH 11.6, due to the their low solubility in water, and all the results are shown in Figure S49. A plot of *k*_obs_ (*k*_obs_=TOF_max_) vs. *η* is offered in Figure 5 B. As can be observed, the *k*_obs_ obtained are in the range of 5–35 s^−1^. For the two phenyl substituted complexes **[(L4)Cu]^2−^** and **[(L6)Cu]^2−^** the increase in rates correlates with an increase of overpotential, as expected from linear free energy relationships. However, from the graph it is further evident that the naphthyl substituted complexes have higher rates than the phenyl substituted ones in spite of the higher overpotential and thus higher thermodynamic driving force for water oxidation found for the latter. This is consistent with the lower reorganization energy of the naphthyl fragment versus the phenyl group upon electron transfer due to the more extended π‐delocalized system and also with the potentially closer proximity of the Cu‐HOOH^.^ group to the aromatic ring in the 2c‐3 e^−^ species.[^48^, ^49^, ^50^] Both complexes **[(L7)Cu]^2−^** and **[(L8)Cu]^2−^** feature subsequent oxidations in the ligands where the resulting spin distribution is delocalized within the naphthalenyl amides as shown by DFT (Figure S68), following similar trends to **[(L3)Cu]** complex.

Faradaic efficiencies of 52 % and 40 % for **[(L7)Cu]^2−^** and **[(L8)Cu]^2−^** respectively were obtained that are a bit lower than that of **[(L4)Cu]^2−^**, indicating again that the naphthyl groups are easier to oxidize/degrade than the corresponding phenyl group (Figures S52–S55 and S27, S28).

## Conclusion

The Cu^II^ complexes of general formula **[(LN)Cu^II^]** (N=1–3) and **[(LN)Cu^II^]^2−^** (N=4–8) containing aryl substituted bpy‐diamidate ligands have proven to be an excellent platform to evaluate ligands effects on water oxidation catalytic properties. The electron transfer processes to enable O−O bond formation in this family of Cu^II^ complexes occurs exclusively on the ligand while the Cu centers remain at oxidation state II.

At the initial stages of the catalytic cycle, the Cu^II^ complexes undergo two one‐electron oxidation process followed by the critical O−O bond formation step. The role of the Cu center here is mainly as a scaffold that holds the two bpy‐amide ligand bonded to the metal center as it undergoes different denticity along the intermediates involved in the catalytic cycle, and at the same time the Cu center allows for OH^−^ coordination and activation. Further, once the OH^−^ is coordinated, the Cu‐OH group will be attacked by exogenous OH^−^ via a radical‐nucleophilic pathway, generating an oxygen‐oxygen bond leading to a Cu‐(HOOH) species. This process involves an initial intramolecular single ET event between the phenyl (or naphthyl) radical cation and the initial Cu‐OH group, and therefore the metal center is responsible for placing the two groups sufficiently close so that fast ET can occur. The aryl substituent plays a critical role both from a thermodynamic perspective, because it will tune the overpotential at which catalysis occurs, and from a kinetic perspective, because of the low reorganization energy of extended polyacenes upon ET, which can ensure fast kinetic processes.

The “all ligand‐based” strategy reported here is extremely attractive because of the large variety of organic substituents that can be potentially used together with the straightforward synthesis of both the ligands and their Cu^II^ complexes. This therefore opens up a completely new avenue for the design of new ligand‐based water oxidation catalysts that eventually could lead to metal free WOCs.

A key challenging feature that remains unsolved here is the low FE obtained for these complexes that is due to competitive deactivation pathways. We are confident that the synthetic versatility of molecular transition metal complexes ensured by the large variety of potential ligands that can be used will lead to rugged and efficient all ligand‐based WOCs in the near future.

## Conflict of interest

The authors declare no conflict of interest.

## Supporting information

As a service to our authors and readers, this journal provides supporting information supplied by the authors. Such materials are peer reviewed and may be re‐organized for online delivery, but are not copy‐edited or typeset. Technical support issues arising from supporting information (other than missing files) should be addressed to the authors.

Supporting Information

Supporting Information
