# Supplementary material for: Consecutive Ligand‐Based Electron Transfer in New Molecular Copper‐Based Water Oxidation Catalysts
Source: Angew Chem Int Ed Engl. 2021 Jul 16;60(34):18639–44. doi: 10.1002/anie.202104020 (PMC8456863; doi:10.1002/anie.202104020)

checkCIF/PLATON report

Structure factors have been supplied for datablock(s) CuL1, CuL2, CuL3, CuL4, CuL5, CuL6, CuL7, H2L5

THIS REPORT IS FOR GUIDANCE ONLY. IF USED AS PART OF A REVIEW PROCEDURE FOR PUBLICATION, IT SHOULD NOT REPLACE THE EXPERTISE OF AN EXPERIENCED CRYSTALLOGRAPHIC REFEREE.

No syntax errors found.   CIF dictionary   Interpreting this report

Datablock: CuL1

```
Bond precision:  C-C = 0.0025 A          Wavelength=0.71073

Cell:           a=15.9944(5)    b=11.4267(3)        c=12.8270(4)
                alpha=90        beta=107.431(1)     gamma=90
Temperature:    100 K
```

	Calculated	Reported
Volume	2236.65(12)	2236.65(12)
Space group	P 21/c	P 21/c
Hall group	-P 2ybc	-P 2ybc
Moiety formula	C24 H18 Cu N4 O3, C H4 O, H2 O	?
Sum formula	C25 H24 Cu N4 O5	C25 H24 Cu N4 O5
Mr	524.03	524.02
Dx,g cm-3	1.556	1.556
Z	4	4
Mu (mm-1)	1.024	1.024
F000	1084.0	1084.0
F000’	1085.69	
h,k,lmax	22,16,18	22,16,18
Nref	6978	6674
Tmin,Tmax	0.940,0.980	0.696,0.746
Tmin’	0.815	

```
Correction method= # Reported T Limits: Tmin=0.696 Tmax=0.746
AbsCorr = MULTI-SCAN

Data completeness= 0.956          Theta(max)= 30.740

R(reflections)= 0.0345( 5337)     wR2(reflections)= 0.0853( 6674)

S = 1.027                   Npar= 337
```

```
The following ALERTS were generated. Each ALERT has the format
       test-name_ALERT_alert-type_alert-level.
Click on the hyperlinks for more details of the test.
```

Alert level C

```
PLAT220_ALERT_2_C NonSolvent   Resd 1  C   Ueq(max)/Ueq(min) Range        3.2 Ratio
PLAT911_ALERT_3_C Missing FCF Refl Between Thmin & STh/L=    0.600         29 Report
```

Alert level G

```
PLAT232_ALERT_2_G Hirshfeld Test Diff (M-X)  Cu1      --O3       .         5.3 s.u.
PLAT432_ALERT_2_G Short Inter X...Y Contact  C1       ..C8                3.20 Ang.
                                           1-x,-1/2+y,1/2-z  =      2_645 Check
PLAT720_ALERT_4_G Number of Unusual/Non-Standard Labels .........           3 Note
PLAT794_ALERT_5_G Tentative Bond Valency for Cu1       (I)       .        1.29 Info
PLAT883_ALERT_1_G No Info/Value for _atom_sites_solution_primary .     Please Do !
PLAT910_ALERT_3_G Missing # of FCF Reflection(s) Below Theta(Min).          1 Note
PLAT912_ALERT_4_G Missing # of FCF Reflections Above STh/L=  0.600        275 Note
PLAT933_ALERT_2_G Number of OMIT Records in Embedded .res File ...          1 Note
PLAT941_ALERT_3_G Average HKL Measurement Multiplicity ..........         3.5 Low
PLAT978_ALERT_2_G Number C-C Bonds with Positive Residual Density.         12 Info
```

```
   0 ALERT level A = Most likely a serious problem - resolve or explain
   0 ALERT level B = A potentially serious problem, consider carefully
   2 ALERT level C = Check. Ensure it is not caused by an omission or oversight
  10 ALERT level G = General information/check it is not something unexpected

   1 ALERT type 1 CIF construction/syntax error, inconsistent or missing data
   5 ALERT type 2 Indicator that the structure model may be wrong or deficient
   3 ALERT type 3 Indicator that the structure quality may be low
   2 ALERT type 4 Improvement, methodology, query or suggestion
   1 ALERT type 5 Informative message, check
```

Datablock: CuL2

```
Bond precision:   C-C = 0.0033 A          Wavelength=0.71073

Cell:             a=17.396(5)     b=23.321(7)        c=14.534(4)
                  alpha=90        beta=110.595(8)    gamma=90
Temperature:      100 K
```

	Calculated	Reported
Volume	5520(3)	5520(3)
Space group	P 21/c	P 21/c
Hall group	-P 2ybc	-P 2ybc
Moiety formula	4(C29 H28 Cu N4 O3), 3(C H4 O)	?
Sum formula	C119 H124 Cu4 N16 O15	C29.75 H31 Cu N4 O3.75
Mr	2272.54	568.12
Dx,g cm-3	1.367	1.367
Z	2	8
Mu (mm-1)	0.832	0.832
F000	2372.0	2372.0
F000'	2375.38	
h,k,lmax	25,34,21	24,34,21
Nref	18660	17725
Tmin,Tmax	0.847,0.920	0.637,0.746
Tmin'	0.847	

Correction method= # Reported T Limits: Tmin=0.637 Tmax=0.746
AbsCorr = MULTI-SCAN

Data completeness= 0.950 Theta(max)= 31.684

R(reflections)= 0.0460(11544) wR2(reflections)= 0.1420(17725)

S = 1.041 Npar= 782

The following ALERTS were generated. Each ALERT has the format
test-name_ALERT_alert-type_alert-level.
Click on the hyperlinks for more details of the test.

Alert level C

```
PLAT220_ALERT_2_C NonSolvent   Resd 1  C   Ueq(max)/Ueq(min) Range        4.5 Ratio
PLAT222_ALERT_3_C NonSolvent Resd 1  H   Uiso(max)/Uiso(min) Range        5.3 Ratio
PLAT905_ALERT_3_C Negative K value in the Analysis of Variance ...     -0.282 Report
PLAT911_ALERT_3_C Missing FCF Refl Between Thmin & STh/L=    0.600         92 Report
```

Alert level G

```
PLAT002_ALERT_2_G Number of Distance or Angle Restraints on AtSite         14 Note
PLAT003_ALERT_2_G Number of Uiso or Uij Restrained non-H Atoms ...         12 Report
PLAT007_ALERT_5_G Number of Unrefined Donor-H Atoms ..............          7 Report
PLAT045_ALERT_1_G Calculated and Reported Z Differ by a Factor ...       0.25 Check
PLAT172_ALERT_4_G The CIF-Embedded .res File Contains DFIX Records          7 Report
PLAT177_ALERT_4_G The CIF-Embedded .res File Contains DELU Records          3 Report
PLAT178_ALERT_4_G The CIF-Embedded .res File Contains SIMU Records          3 Report
PLAT186_ALERT_4_G The CIF-Embedded .res File Contains ISOR Records          3 Report
PLAT230_ALERT_2_G Hirshfeld Test Diff for    O1T'     --C1T'     .        7.5 s.u.
PLAT232_ALERT_2_G Hirshfeld Test Diff (M-X)  Cu1B     --O1K      .        6.9 s.u.
PLAT301_ALERT_3_G Main Residue  Disorder .............(Resd  1  )          5% Note
PLAT302_ALERT_4_G Anion/Solvent/Minor-Residue Disorder (Resd  3  )       100% Note
PLAT302_ALERT_4_G Anion/Solvent/Minor-Residue Disorder (Resd  4  )       100% Note
```

```
PLAT302_ALERT_4_G Anion/Solvent/Minor-Residue Disorder (Resd  5  )       100% Note
PLAT302_ALERT_4_G Anion/Solvent/Minor-Residue Disorder (Resd  6  )       100% Note
PLAT304_ALERT_4_G Non-Integer Number of Atoms in ..... (Resd  3  )       3.60 Check
PLAT304_ALERT_4_G Non-Integer Number of Atoms in ..... (Resd  4  )       2.40 Check
PLAT304_ALERT_4_G Non-Integer Number of Atoms in ..... (Resd  5  )       2.05 Check
PLAT304_ALERT_4_G Non-Integer Number of Atoms in ..... (Resd  6  )       0.95 Check
PLAT413_ALERT_2_G Short Inter XH3 .. XHn     H19E     ..H1SA     .       2.11 Ang.
                                                     x,y,z  =      1_555 Check
PLAT720_ALERT_4_G Number of Unusual/Non-Standard Labels ..........         31 Note
PLAT794_ALERT_5_G Tentative Bond Valency for Cu1B      (I)       .       1.30 Info
PLAT860_ALERT_3_G Number of Least-Squares Restraints .............        195 Note
PLAT883_ALERT_1_G No Info/Value for _atom_sites_solution_primary .     Please Do !
PLAT910_ALERT_3_G Missing # of FCF Reflection(s) Below Theta(Min).          1 Note
PLAT912_ALERT_4_G Missing # of FCF Reflections Above STh/L=  0.600        783 Note
PLAT933_ALERT_2_G Number of OMIT Records in Embedded .res File ...          4 Note
PLAT941_ALERT_3_G Average HKL Measurement Multiplicity ...........        3.4 Low
PLAT978_ALERT_2_G Number C-C Bonds with Positive Residual Density.          7 Info
```

```
   0 ALERT level A = Most likely a serious problem - resolve or explain
   0 ALERT level B = A potentially serious problem, consider carefully
   4 ALERT level C = Check. Ensure it is not caused by an omission or oversight
  29 ALERT level G = General information/check it is not something unexpected

   2 ALERT type 1 CIF construction/syntax error, inconsistent or missing data
   8 ALERT type 2 Indicator that the structure model may be wrong or deficient
   7 ALERT type 3 Indicator that the structure quality may be low
  14 ALERT type 4 Improvement, methodology, query or suggestion
   2 ALERT type 5 Informative message, check
```

Datablock: CuL3

```
Bond precision:  C-C = 0.0036 A          Wavelength=0.71073

Cell:           a=13.5604(5)    b=15.8885(5)          c=14.3149(5)
                alpha=90        beta=114.8570(11)     gamma=90
Temperature:    100 K
```

	Calculated	Reported
Volume	2798.49(17)	2798.49(17)
Space group	P 21/c	P2(1)/c
Hall group	-P 2ybc	-P 2ybc
Moiety formula	C33 H24 Cu N4 O3, C H4 O	?
Sum formula	C34 H28 Cu N4 O4	C68 H56 Cl0 Cu2 N8 O8
Mr	620.15	1240.28
Dx,g cm-3	1.472	1.472
Z	4	2
Mu (mm-1)	0.829	0.829
F000	1284.0	1284.0
F000’	1285.74	
h,k,lmax	17,19,17	17,19,17
Nref	5811	5807
Tmin,Tmax	0.820,0.920	0.880,0.922
Tmin’	0.780	

Correction method= # Reported T Limits: Tmin=0.880 Tmax=0.922
AbsCorr = MULTI-SCAN

Data completeness= 0.999 Theta(max)= 26.539

R(reflections)= 0.0367(4250) wR2(reflections)= 0.0815(5807)

S = 1.019 Npar= 515

The following ALERTS were generated. Each ALERT has the format
test-name_ALERT_alert-type_alert-level.
Click on the hyperlinks for more details of the test.

Alert level C

```
PLAT041_ALERT_1_C Calc. and Reported SumFormula    Strings  Differ     Please Check
PLAT334_ALERT_2_C Small Aver. Benzene C-C Dist C23’     -C32’            1.37 Ang.
```

Alert level G

```
CELLZ01_ALERT_1_G Difference between formula and atom_site contents detected.
CELLZ01_ALERT_1_G ALERT: Large difference may be due to a
             symmetry error - see SYMMG tests
            From the CIF: _cell_formula_units_Z    2
            From the CIF: _chemical_formula_sum  C68 H56 Cl0 Cu2 N8 O8
            TEST: Compare cell contents of formula and atom_site data

            atom    Z*formula  cif sites diff
            C        136.00    136.00    0.00
            H        112.00    112.00    0.00
            Cl         2.00      0.00    2.00
            Cu         4.00      4.00    0.00
            N         16.00     16.00    0.00
            O         16.00     16.00    0.00
PLAT003_ALERT_2_G Number of Uiso or Uij Restrained non-H Atoms ...          26 Report
PLAT017_ALERT_1_G Check Scattering Type Consistency of CO1      as          CU
```

```
PLAT045_ALERT_1_G Calculated and Reported Z Differ by a Factor ...       2.00 Check
PLAT177_ALERT_4_G The CIF-Embedded .res File Contains DELU Records          2 Report
PLAT178_ALERT_4_G The CIF-Embedded .res File Contains SIMU Records          2 Report
PLAT186_ALERT_4_G The CIF-Embedded .res File Contains ISOR Records          2 Report
PLAT301_ALERT_3_G Main Residue  Disorder ..............(Resd  1  )        32% Note
PLAT335_ALERT_2_G Check Large C6 Ring C-C Range C23'     -C32'           0.24 Ang.
PLAT335_ALERT_2_G Check Large C6 Ring C-C Range C24'     -C29'           0.21 Ang.
PLAT413_ALERT_2_G Short Inter XH3 .. XHn     H33C     ..H28'     .       2.11 Ang.
                                            -x,-1/2+y,1/2-z  =      2_545 Check
PLAT720_ALERT_4_G Number of Unusual/Non-Standard Labels ..........          4 Note
PLAT811_ALERT_5_G No ADDSYM Analysis: Too Many Excluded Atoms ....          ! Info
PLAT860_ALERT_3_G Number of Least-Squares Restraints .............        632 Note
PLAT883_ALERT_1_G No Info/Value for _atom_sites_solution_primary .     Please Do !
PLAT910_ALERT_3_G Missing # of FCF Reflection(s) Below Theta(Min).          1 Note
PLAT912_ALERT_4_G Missing # of FCF Reflections Above STh/L=  0.600          3 Note
PLAT978_ALERT_2_G Number C-C Bonds with Positive Residual Density.          9 Info
```

```
   0 ALERT level A = Most likely a serious problem - resolve or explain
   0 ALERT level B = A potentially serious problem, consider carefully
   2 ALERT level C = Check. Ensure it is not caused by an omission or oversight
  19 ALERT level G = General information/check it is not something unexpected

   6 ALERT type 1 CIF construction/syntax error, inconsistent or missing data
   6 ALERT type 2 Indicator that the structure model may be wrong or deficient
   3 ALERT type 3 Indicator that the structure quality may be low
   5 ALERT type 4 Improvement, methodology, query or suggestion
   1 ALERT type 5 Informative message, check
```

Datablock: CuL4

```
Bond precision:  C-C = 0.0026 A          Wavelength=0.71073

Cell:           a=7.1558(3)       b=12.3988(5)      c=18.4833(7)
                alpha=70.559(1)   beta=89.324(1)    gamma=80.683(1)
Temperature:    100 K
```

	Calculated	Reported
Volume	1524.44(11)	1524.44(11)
Space group	P -1	P-1
Hall group	-P 1	-P 1
Moiety formula	C24 H23.50 Cu N4 Na2 O12.75 S2, 2.9(H0.20 O0.10)	?
Sum formula	C24 H27.50 Cu N4 Na2 O14.75 S2	C24 H27.50 Cu N4 Na2 O14.75 S2
Mr	781.62	781.64
Dx,g cm-3	1.703	1.703
Z	2	2
Mu (mm-1)	0.961	0.961
F000	801.0	801.0
F000’	802.62	
h,k,lmax	11,19,29	11,19,28
Nref	12341	11681
Tmin,Tmax	0.625,0.972	0.863,0.972
Tmin’	0.612	

Correction method= # Reported T Limits: Tmin=0.863 Tmax=0.972
AbsCorr = MULTI-SCAN

Data completeness= 0.947   Theta(max)= 33.916

R(reflections)= 0.0452(9977)   wR2(reflections)= 0.1165(11681)

S = 1.120   Npar= 965

The following ALERTS were generated. Each ALERT has the format
test-name_ALERT_alert-type_alert-level.
Click on the hyperlinks for more details of the test.

Alert level C

```
PLAT077_ALERT_4_C Unitcell Contains Non-integer Number of Atoms ..     Please Check
PLAT220_ALERT_2_C NonSolvent   Resd 1  O   Ueq(max)/Ueq(min) Range        5.8 Ratio
PLAT222_ALERT_3_C NonSolvent Resd 1  H   Uiso(max)/Uiso(min) Range        9.6 Ratio
PLAT334_ALERT_2_C Small Aver. Benzene C-C Dist C4       -C5X             1.37 Ang.
PLAT906_ALERT_3_C Large K Value in the Analysis of Variance ......      2.090 Check
PLAT911_ALERT_3_C Missing FCF Refl Between Thmin & STh/L=    0.600         56 Report
PLAT977_ALERT_2_C Check Negative Difference Density on H3WA            -0.33 eA-3
PLAT977_ALERT_2_C Check Negative Difference Density on H12J            -0.36 eA-3
```

Alert level G

```
PLAT002_ALERT_2_G Number of Distance or Angle Restraints on AtSite         29 Note
PLAT003_ALERT_2_G Number of Uiso or Uij Restrained non-H Atoms ...        100 Report
PLAT004_ALERT_5_G Polymeric Structure Found with Maximum Dimension          2 Info
PLAT007_ALERT_5_G Number of Unrefined Donor-H Atoms .............          18 Report
PLAT154_ALERT_1_G The s.u.’s on the Cell Angles are Equal ..(Note)      0.001 Degree
PLAT172_ALERT_4_G The CIF-Embedded .res File Contains DFIX Records         28 Report
```

```
PLAT177_ALERT_4_G The CIF-Embedded .res File Contains DELU Records          2 Report
PLAT178_ALERT_4_G The CIF-Embedded .res File Contains SIMU Records          2 Report
PLAT186_ALERT_4_G The CIF-Embedded .res File Contains ISOR Records          6 Report
PLAT300_ALERT_4_G Atom Site Occupancy of O3WA       Constrained at       0.65 Check
PLAT300_ALERT_4_G Atom Site Occupancy of O11W       Constrained at        0.5 Check
PLAT300_ALERT_4_G Atom Site Occupancy of O3WB       Constrained at        0.2 Check
PLAT300_ALERT_4_G Atom Site Occupancy of O3WC       Constrained at        0.1 Check
PLAT300_ALERT_4_G Atom Site Occupancy of O4WB       Constrained at        0.3 Check
PLAT300_ALERT_4_G Atom Site Occupancy of O4WA       Constrained at       0.35 Check
PLAT300_ALERT_4_G Atom Site Occupancy of O11B       Constrained at        0.3 Check
PLAT300_ALERT_4_G Atom Site Occupancy of O11C       Constrained at        0.2 Check
PLAT300_ALERT_4_G Atom Site Occupancy of O4WC       Constrained at        0.1 Check
PLAT300_ALERT_4_G Atom Site Occupancy of O12B       Constrained at       0.25 Check
PLAT300_ALERT_4_G Atom Site Occupancy of O12C       Constrained at       0.15 Check
PLAT300_ALERT_4_G Atom Site Occupancy of O12W       Constrained at       0.35 Check
PLAT300_ALERT_4_G Atom Site Occupancy of O5WA       Constrained at        0.3 Check
PLAT300_ALERT_4_G Atom Site Occupancy of H3WA       Constrained at       0.65 Check
PLAT300_ALERT_4_G Atom Site Occupancy of H3WB       Constrained at       0.65 Check
PLAT300_ALERT_4_G Atom Site Occupancy of H11X       Constrained at        0.5 Check
PLAT300_ALERT_4_G Atom Site Occupancy of H11Y       Constrained at        0.5 Check
PLAT300_ALERT_4_G Atom Site Occupancy of H3WC       Constrained at        0.2 Check
PLAT300_ALERT_4_G Atom Site Occupancy of H3WD       Constrained at        0.2 Check
PLAT300_ALERT_4_G Atom Site Occupancy of H3WE       Constrained at        0.1 Check
PLAT300_ALERT_4_G Atom Site Occupancy of H3WF       Constrained at        0.1 Check
PLAT300_ALERT_4_G Atom Site Occupancy of H4WC       Constrained at        0.3 Check
PLAT300_ALERT_4_G Atom Site Occupancy of H11J       Constrained at        0.3 Check
PLAT300_ALERT_4_G Atom Site Occupancy of H11K       Constrained at        0.3 Check
PLAT300_ALERT_4_G Atom Site Occupancy of H11L       Constrained at        0.2 Check
PLAT300_ALERT_4_G Atom Site Occupancy of H11M       Constrained at        0.2 Check
PLAT300_ALERT_4_G Atom Site Occupancy of H4WD       Constrained at        0.3 Check
PLAT300_ALERT_4_G Atom Site Occupancy of H12J       Constrained at       0.25 Check
PLAT300_ALERT_4_G Atom Site Occupancy of H12K       Constrained at       0.25 Check
PLAT300_ALERT_4_G Atom Site Occupancy of H12L       Constrained at       0.15 Check
PLAT300_ALERT_4_G Atom Site Occupancy of H12M       Constrained at       0.15 Check
PLAT300_ALERT_4_G Atom Site Occupancy of H12X       Constrained at       0.35 Check
PLAT300_ALERT_4_G Atom Site Occupancy of H12Y       Constrained at       0.35 Check
PLAT300_ALERT_4_G Atom Site Occupancy of H4WA       Constrained at       0.35 Check
PLAT300_ALERT_4_G Atom Site Occupancy of H4WB       Constrained at       0.35 Check
PLAT300_ALERT_4_G Atom Site Occupancy of H4WE       Constrained at        0.1 Check
PLAT300_ALERT_4_G Atom Site Occupancy of H4WF       Constrained at        0.1 Check
PLAT300_ALERT_4_G Atom Site Occupancy of H5WA       Constrained at        0.3 Check
PLAT300_ALERT_4_G Atom Site Occupancy of H5WB       Constrained at        0.3 Check
PLAT300_ALERT_4_G Atom Site Occupancy of O9WA       Constrained at        0.6 Check
PLAT300_ALERT_4_G Atom Site Occupancy of H9WA       Constrained at        0.6 Check
PLAT300_ALERT_4_G Atom Site Occupancy of H9WB       Constrained at        0.6 Check
PLAT300_ALERT_4_G Atom Site Occupancy of O9WB       Constrained at        0.3 Check
PLAT300_ALERT_4_G Atom Site Occupancy of H9WC       Constrained at        0.3 Check
PLAT300_ALERT_4_G Atom Site Occupancy of H9WD       Constrained at        0.3 Check
PLAT300_ALERT_4_G Atom Site Occupancy of O9WC       Constrained at        0.1 Check
PLAT300_ALERT_4_G Atom Site Occupancy of H9WE       Constrained at        0.1 Check
PLAT300_ALERT_4_G Atom Site Occupancy of H9WF       Constrained at        0.1 Check
PLAT301_ALERT_3_G Main Residue  Disorder .............(Resd  1  )        52% Note
PLAT302_ALERT_4_G Anion/Solvent/Minor-Residue Disorder (Resd  3  )       100% Note
PLAT302_ALERT_4_G Anion/Solvent/Minor-Residue Disorder (Resd  4  )       100% Note
PLAT302_ALERT_4_G Anion/Solvent/Minor-Residue Disorder (Resd  5  )       100% Note
PLAT335_ALERT_2_G Check Large C6 Ring C-C Range C4       -C5             0.22 Ang.
PLAT335_ALERT_2_G Check Large C6 Ring C-C Range C4       -C5'            0.15 Ang.
PLAT335_ALERT_2_G Check Large C6 Ring C-C Range C4       -C5X            0.18 Ang.
PLAT335_ALERT_2_G Check Large C6 Ring C-C Range C19      -C24            0.23 Ang.
PLAT335_ALERT_2_G Check Large C6 Ring C-C Range C19      -C24'           0.17 Ang.
PLAT335_ALERT_2_G Check Large C6 Ring C-C Range C19      -C24X           0.20 Ang.
PLAT720_ALERT_4_G Number of Unusual/Non-Standard Labels ..........         42 Note
```

```
PLAT764_ALERT_4_G Overcomplete CIF Bond List Detected (Rep/Expd) .       2.93 Ratio
PLAT779_ALERT_4_G Suspect or Irrelevant (Bond) Angle(s) in CIF . #         52 Check
              NA1A -S1   -NA2B    2.767   1.555   2.667             37.52 Deg.
PLAT779_ALERT_4_G Suspect or Irrelevant (Bond) Angle(s) in CIF . #         55 Check
              O5   -S1   -NA1B    1.555   1.555   1.455             41.10 Deg.
PLAT779_ALERT_4_G Suspect or Irrelevant (Bond) Angle(s) in CIF . #         65 Check
              NA1A -O5   -NA1C    1.455   1.555   1.455             25.92 Deg.
PLAT779_ALERT_4_G Suspect or Irrelevant (Bond) Angle(s) in CIF . #         68 Check
              NA1C -O5   -NA1B    1.455   1.555   1.455             30.49 Deg.
PLAT779_ALERT_4_G Suspect or Irrelevant (Bond) Angle(s) in CIF . #        109 Check
              O7   -S2   -NA2C    1.555   1.555   1.655             24.90 Deg.
PLAT779_ALERT_4_G Suspect or Irrelevant (Bond) Angle(s) in CIF . #        114 Check
              O8   -S2   -NA1C    1.555   1.555   2.767             30.33 Deg.
PLAT779_ALERT_4_G Suspect or Irrelevant (Bond) Angle(s) in CIF . #        118 Check
              NA1B -S2   -NA1C    2.767   1.555   2.767             16.15 Deg.
PLAT779_ALERT_4_G Suspect or Irrelevant (Bond) Angle(s) in CIF . #        121 Check
              O8   -S2   -NA1B    1.555   1.555   1.555             38.52 Deg.
PLAT779_ALERT_4_G Suspect or Irrelevant (Bond) Angle(s) in CIF . #        126 Check
              NA1C -S2   -NA1B    1.555   1.555   1.555             19.79 Deg.
PLAT779_ALERT_4_G Suspect or Irrelevant (Bond) Angle(s) in CIF . #        130 Check
              O7   -S2   -NA1A    1.555   1.555   1.555             41.10 Deg.
PLAT779_ALERT_4_G Suspect or Irrelevant (Bond) Angle(s) in CIF . #        134 Check
              NA1C -S2   -NA1A    1.555   1.555   1.555             13.73 Deg.
PLAT779_ALERT_4_G Suspect or Irrelevant (Bond) Angle(s) in CIF . #        135 Check
              NA2C -S2   -NA1A    1.655   1.555   1.555             38.40 Deg.
PLAT779_ALERT_4_G Suspect or Irrelevant (Bond) Angle(s) in CIF . #        137 Check
              NA1B -S2   -NA1A    1.555   1.555   1.555             32.87 Deg.
PLAT779_ALERT_4_G Suspect or Irrelevant (Bond) Angle(s) in CIF . #        145 Check
              NA2C -O7   -NA2B    1.655   1.555   1.655             27.81 Deg.
PLAT779_ALERT_4_G Suspect or Irrelevant (Bond) Angle(s) in CIF . #        149 Check
              NA1C -O7   -NA1A    1.555   1.555   1.555             23.61 Deg.
PLAT779_ALERT_4_G Suspect or Irrelevant (Bond) Angle(s) in CIF . #        153 Check
              NA1B -O8   -NA1C    2.767   1.555   2.767             33.43 Deg.
PLAT779_ALERT_4_G Suspect or Irrelevant (Bond) Angle(s) in CIF . #        160 Check
              NA1C -O8   -NA1B    1.555   1.555   1.555             29.98 Deg.
PLAT779_ALERT_4_G Suspect or Irrelevant (Bond) Angle(s) in CIF . #        163 Check
              NA1C -O8   -NA1A    2.767   1.555   2.767             23.85 Deg.
PLAT779_ALERT_4_G Suspect or Irrelevant (Bond) Angle(s) in CIF . #        192 Check
              O2'  -S1'  -NA1A    1.555   1.555   1.455             24.76 Deg.
PLAT779_ALERT_4_G Suspect or Irrelevant (Bond) Angle(s) in CIF . #        197 Check
              O2'  -S1'  -NA1C    1.555   1.555   1.455             23.86 Deg.
PLAT779_ALERT_4_G Suspect or Irrelevant (Bond) Angle(s) in CIF . #        201 Check
              NA1A -S1'  -NA1C    1.455   1.555   1.455             18.70 Deg.
PLAT779_ALERT_4_G Suspect or Irrelevant (Bond) Angle(s) in CIF . #        202 Check
              O3'  -S1'  -NA1C    1.555   1.555   2.767             38.55 Deg.
PLAT779_ALERT_4_G Suspect or Irrelevant (Bond) Angle(s) in CIF . #        206 Check
              NA1A -S1'  -NA1C    2.767   1.555   2.767             13.48 Deg.
PLAT779_ALERT_4_G Suspect or Irrelevant (Bond) Angle(s) in CIF . #        214 Check
              NA1A -S1'  -NA1B    1.455   1.555   1.455             36.22 Deg.
PLAT779_ALERT_4_G Suspect or Irrelevant (Bond) Angle(s) in CIF . #        215 Check
              NA1C -S1'  -NA1B    1.455   1.555   1.455             22.22 Deg.
PLAT779_ALERT_4_G Suspect or Irrelevant (Bond) Angle(s) in CIF . #        219 Check
              O1'  -S1'  -NA2B    1.555   1.555   2.667             40.09 Deg.
PLAT779_ALERT_4_G Suspect or Irrelevant (Bond) Angle(s) in CIF . #        221 Check
              NA1A -S1'  -NA2B    2.767   1.555   2.667             34.95 Deg.
PLAT779_ALERT_4_G Suspect or Irrelevant (Bond) Angle(s) in CIF . #        234 Check
              NA1A -O2'  -NA1C    1.455   1.555   1.455             32.34 Deg.
PLAT779_ALERT_4_G Suspect or Irrelevant (Bond) Angle(s) in CIF . #        241 Check
              NA2C -O2'  -NA2B    1.555   1.555   1.555             27.45 Deg.
PLAT779_ALERT_4_G Suspect or Irrelevant (Bond) Angle(s) in CIF . #        244 Check
              NA1C -O2'  -NA1B    1.455   1.555   1.455             29.77 Deg.
PLAT779_ALERT_4_G Suspect or Irrelevant (Bond) Angle(s) in CIF . #        255 Check
```

```
              NA1A -O3'  -NA1C    2.767   1.555   2.767             27.02 Deg.
PLAT779_ALERT_4_G Suspect or Irrelevant (Bond) Angle(s) in CIF . #        258 Check
              NA1C -O3'  -NA1B    2.767   1.555   2.767             30.76 Deg.
PLAT779_ALERT_4_G Suspect or Irrelevant (Bond) Angle(s) in CIF . #        285 Check
              O7'  -S2'  -NA2C    1.555   1.555   1.655             41.00 Deg.
PLAT779_ALERT_4_G Suspect or Irrelevant (Bond) Angle(s) in CIF . #        290 Check
              O7'  -S2'  -NA1C    1.555   1.555   1.555              4.70 Deg.
PLAT779_ALERT_4_G Suspect or Irrelevant (Bond) Angle(s) in CIF . #        294 Check
              NA2C -S2'  -NA1C    1.655   1.555   1.555             44.22 Deg.
PLAT779_ALERT_4_G Suspect or Irrelevant (Bond) Angle(s) in CIF . #        299 Check
              NA2B -O6'  -NA2C    1.555   1.555   1.555             20.50 Deg.
PLAT779_ALERT_4_G Suspect or Irrelevant (Bond) Angle(s) in CIF . #        303 Check
              NA1C -O7'  -NA1A    1.555   1.555   1.555             24.09 Deg.
PLAT779_ALERT_4_G Suspect or Irrelevant (Bond) Angle(s) in CIF . #        314 Check
              NA2C -O7'  -NA2B    1.655   1.555   1.655             24.29 Deg.
PLAT779_ALERT_4_G Suspect or Irrelevant (Bond) Angle(s) in CIF . #        317 Check
              NA1C -O7'  -NA1B    1.555   1.555   1.555             19.74 Deg.
PLAT779_ALERT_4_G Suspect or Irrelevant (Bond) Angle(s) in CIF . #        318 Check
              NA1A -O7'  -NA1B    1.555   1.555   1.555             42.91 Deg.
PLAT779_ALERT_4_G Suspect or Irrelevant (Bond) Angle(s) in CIF . #        376 Check
              O1A' -S1A' -NA1A    1.555   1.555   2.767             31.70 Deg.
PLAT779_ALERT_4_G Suspect or Irrelevant (Bond) Angle(s) in CIF . #        378 Check
              O3A' -S1A' -NA1B    1.555   1.555   1.455             21.90 Deg.
PLAT779_ALERT_4_G Suspect or Irrelevant (Bond) Angle(s) in CIF . #        387 Check
              NA1A -S1A' -NA2B    2.767   1.555   2.667             36.16 Deg.
PLAT779_ALERT_4_G Suspect or Irrelevant (Bond) Angle(s) in CIF . #        393 Check
              NA1A -O1A' -NA1C    2.767   1.555   2.767             19.73 Deg.
PLAT779_ALERT_4_G Suspect or Irrelevant (Bond) Angle(s) in CIF . #        398 Check
              NA1B -O3A' -NA1C    1.455   1.555   1.455             35.13 Deg.
PLAT779_ALERT_4_G Suspect or Irrelevant (Bond) Angle(s) in CIF . #        401 Check
              NA1C -O3A' -NA1A    1.455   1.555   1.455             26.02 Deg.
PLAT779_ALERT_4_G Suspect or Irrelevant (Bond) Angle(s) in CIF . #        412 Check
              O7A' -S2A' -NA1B    1.555   1.555   2.767             33.56 Deg.
PLAT779_ALERT_4_G Suspect or Irrelevant (Bond) Angle(s) in CIF . #        417 Check
              O6A' -S2A' -NA2B    1.555   1.555   1.555             11.60 Deg.
PLAT779_ALERT_4_G Suspect or Irrelevant (Bond) Angle(s) in CIF . #        422 Check
              O6A' -S2A' -NA2C    1.555   1.555   1.555             17.74 Deg.
PLAT779_ALERT_4_G Suspect or Irrelevant (Bond) Angle(s) in CIF . #        426 Check
              NA2B -S2A' -NA2C    1.555   1.555   1.555             20.39 Deg.
PLAT779_ALERT_4_G Suspect or Irrelevant (Bond) Angle(s) in CIF . #        428 Check
              NA2B -O6A' -NA2C    1.555   1.555   1.555             38.40 Deg.
PLAT779_ALERT_4_G Suspect or Irrelevant (Bond) Angle(s) in CIF . #        441 Check
              NA1B -O7A' -NA1C    2.767   1.555   2.767             24.60 Deg.
PLAT779_ALERT_4_G Suspect or Irrelevant (Bond) Angle(s) in CIF . #        445 Check
              NA1C -O7A' -NA1B    1.555   1.555   1.555             25.68 Deg.
PLAT779_ALERT_4_G Suspect or Irrelevant (Bond) Angle(s) in CIF . #        448 Check
              NA1B -O7A' -NA1A    2.767   1.555   2.767             39.12 Deg.
PLAT779_ALERT_4_G Suspect or Irrelevant (Bond) Angle(s) in CIF . #        450 Check
              NA1C -O7A' -NA1A    2.767   1.555   2.767             19.90 Deg.
PLAT779_ALERT_4_G Suspect or Irrelevant (Bond) Angle(s) in CIF . #        454 Check
              NA2A -O8A' -NA2D    1.555   1.555   1.555             36.03 Deg.
PLAT779_ALERT_4_G Suspect or Irrelevant (Bond) Angle(s) in CIF . #        714 Check
              NA2B -O3WA -NA2C    1.555   1.555   1.555             25.54 Deg.
PLAT779_ALERT_4_G Suspect or Irrelevant (Bond) Angle(s) in CIF . #        717 Check
              NA2A -O3WA -NA2D    2.677   1.555   2.677             36.77 Deg.
PLAT779_ALERT_4_G Suspect or Irrelevant (Bond) Angle(s) in CIF . #        723 Check
              NA2B -O3WA -H3WB    1.555   1.555   1.555              5.00 Deg.
PLAT779_ALERT_4_G Suspect or Irrelevant (Bond) Angle(s) in CIF . #        725 Check
              NA2C -O3WA -H3WB    1.555   1.555   1.555             24.00 Deg.
PLAT779_ALERT_4_G Suspect or Irrelevant (Bond) Angle(s) in CIF . #        733 Check
              NA2D -O3WB -NA2A    1.655   1.555   1.655             36.85 Deg.
PLAT779_ALERT_4_G Suspect or Irrelevant (Bond) Angle(s) in CIF . #        744 Check
```

```
              NA2D -O3WC -NA2D    2.777   1.555   1.655             40.90 Deg.
PLAT779_ALERT_4_G Suspect or Irrelevant (Bond) Angle(s) in CIF . #        760 Check
              NA2B -O4WB -NA2C    1.655   1.555   1.655             21.52 Deg.
PLAT779_ALERT_4_G Suspect or Irrelevant (Bond) Angle(s) in CIF . #        801 Check
              NA1B -O11B -NA1C    1.455   1.555   1.455             27.45 Deg.
PLAT779_ALERT_4_G Suspect or Irrelevant (Bond) Angle(s) in CIF . #        811 Check
              NA2D -O11C -H11M    2.667   1.555   1.555             11.00 Deg.
PLAT780_ALERT_1_G Coordinates do not Form a Properly Connected Set     Please Do !
PLAT804_ALERT_5_G Number of ARU-Code Packing Problem(s) in PLATON          2 Info
PLAT811_ALERT_5_G No ADDSYM Analysis: Too Many Excluded Atoms ....         ! Info
PLAT860_ALERT_3_G Number of Least-Squares Restraints .............      1914 Note
PLAT883_ALERT_1_G No Info/Value for _atom_sites_solution_primary .     Please Do !
PLAT910_ALERT_3_G Missing # of FCF Reflection(s) Below Theta(Min).         1 Note
PLAT912_ALERT_4_G Missing # of FCF Reflections Above STh/L=  0.600       602 Note
PLAT933_ALERT_2_G Number of OMIT Records in Embedded .res File ...         3 Note
PLAT941_ALERT_3_G Average HKL Measurement Multiplicity ...........       3.0 Low
PLAT978_ALERT_2_G Number C-C Bonds with Positive Residual Density.         5 Info
PLAT992_ALERT_5_G Repd & Actual _reflns_number_gt Values Differ by         5 Check
```

```
  0 ALERT level A = Most likely a serious problem - resolve or explain
  0 ALERT level B = A potentially serious problem, consider carefully
  8 ALERT level C = Check. Ensure it is not caused by an omission or oversight
145 ALERT level G = General information/check it is not something unexpected

  3 ALERT type 1 CIF construction/syntax error, inconsistent or missing data
 14 ALERT type 2 Indicator that the structure model may be wrong or deficient
  7 ALERT type 3 Indicator that the structure quality may be low
124 ALERT type 4 Improvement, methodology, query or suggestion
  5 ALERT type 5 Informative message, check
```

Datablock: CuL5

```
Bond precision:  C-C = 0.0020 A         Wavelength=0.71073

Cell:           a=7.5982(3)    b=17.2572(6)        c=24.3433(9)
                alpha=90       beta=98.6877(10)     gamma=90
Temperature:    100 K
```

	Calculated	Reported
Volume	3155.4(2)	3155.4(2)
Space group	P 21/c	P2(1)/c
Hall group	-P 2ybc	-P 2ybc
Moiety formula	C24 H24 Cu N4 Na2 O13 S2, 3(H2 O)	?
Sum formula	C24 H30 Cu N4 Na2 O16 S2	C24 H30 Cu N4 Na2 O16 S2
Mr	804.17	804.16
Dx,g cm-3	1.693	1.693
Z	4	4
Mu (mm-1)	0.934	0.934
F000	1652.0	1652.0
F000'	1655.35	
h,k,lmax	12,27,39	12,27,38
Nref	13894	13011
Tmin,Tmax	0.894,0.954	0.798,0.955
Tmin'	0.688	

Correction method= # Reported T Limits: Tmin=0.798 Tmax=0.955
AbsCorr = MULTI-SCAN

Data completeness= 0.936   Theta(max)= 34.993

R(reflections)= 0.0423(10733)   wR2(reflections)= 0.0978(13011)

S = 1.073   Npar= 506

The following ALERTS were generated. Each ALERT has the format
test-name_ALERT_alert-type_alert-level.
Click on the hyperlinks for more details of the test.

Alert level C

```
PLAT222_ALERT_3_C NonSolvent Resd 1  H   Uiso(max)/Uiso(min) Range       4.9 Ratio
PLAT906_ALERT_3_C Large K Value in the Analysis of Variance ......     2.946 Check
PLAT911_ALERT_3_C Missing FCF Refl Between Thmin & STh/L=    0.600         4 Report
PLAT975_ALERT_2_C Check Calcd Resid. Dens.  0.76A   From O4W            0.49 eA-3
PLAT975_ALERT_2_C Check Calcd Resid. Dens.  0.81A   From O5             0.48 eA-3
```

Alert level G

```
PLAT004_ALERT_5_G Polymeric Structure Found with Maximum Dimension         2 Info
PLAT720_ALERT_4_G Number of Unusual/Non-Standard Labels ..........        16 Note
PLAT764_ALERT_4_G Overcomplete CIF Bond List Detected (Rep/Expd) .      1.13 Ratio
PLAT794_ALERT_5_G Tentative Bond Valency for Cu1       (I)       .      1.28 Info
PLAT883_ALERT_1_G No Info/Value for _atom_sites_solution_primary .    Please Do !
PLAT910_ALERT_3_G Missing # of FCF Reflection(s) Below Theta(Min).         2 Note
PLAT912_ALERT_4_G Missing # of FCF Reflections Above STh/L=  0.600       871 Note
PLAT933_ALERT_2_G Number of OMIT Records in Embedded .res File ...         1 Note
PLAT941_ALERT_3_G Average HKL Measurement Multiplicity ..........        3.0 Low
PLAT978_ALERT_2_G Number C-C Bonds with Positive Residual Density.        11 Info
```

---

0 **ALERT level A** = Most likely a serious problem - resolve or explain
0 **ALERT level B** = A potentially serious problem, consider carefully
5 **ALERT level C** = Check. Ensure it is not caused by an omission or oversight
10 **ALERT level G** = General information/check it is not something unexpected

1 ALERT type 1 CIF construction/syntax error, inconsistent or missing data
4 ALERT type 2 Indicator that the structure model may be wrong or deficient
5 ALERT type 3 Indicator that the structure quality may be low
3 ALERT type 4 Improvement, methodology, query or suggestion
2 ALERT type 5 Informative message, check

Datablock: CuL6

Bond precision: C-C = 0.0034 A Wavelength=0.71073

Cell:	a=11.3964(4)	b=16.5661(6)	c=18.8108(7)
	alpha=109.6735(9)	beta=106.3052(9)	gamma=90.1407(10)
Temperature:	100 K		

	Calculated	Reported
Volume	3191.0(2)	3191.0(2)
Space group	P -1	P -1
Hall group	-P 1	-P 1
Moiety formula	2(C58 H55 Cu2 N8 Na4 O19.50 S4), 3(H O0.50), 2(H0.50 O0.25), 2(	?
Sum formula	C116 H117 Cu4 N16 Na8 O42.50 S8	C29 H29.25 Cu N4 Na2 O10.62 S2
Mr	3109.86	777.45
Dx,g cm-3	1.618	1.618
Z	1	4
Mu (mm-1)	0.909	0.909
F000	1597.0	1597.0
F000’	1600.18	
h,k,lmax	17,24,28	17,24,28
Nref	22283	21472
Tmin,Tmax	0.964,0.973	0.662,0.746
Tmin’	0.964	

Correction method= # Reported T Limits: Tmin=0.662 Tmax=0.746
AbsCorr = MULTI-SCAN

Data completeness= 0.964 Theta(max)= 32.080

R(reflections)= 0.0511(17258) wR2(reflections)= 0.1384(21472)

S = 1.021 Npar= 1143

The following ALERTS were generated. Each ALERT has the format
test-name_ALERT_alert-type_alert-level.
Click on the hyperlinks for more details of the test.

Alert level B

```
PLAT420_ALERT_2_B D-H Without Acceptor       O1W      --H1WA     .     Please Check
```

Author Response: This structure contains several disordered atoms, so that a clear assignement of al water molecules with the corresponding hydrogen atoms was not possible.

```
PLAT420_ALERT_2_B D-H Without Acceptor       O8W      --H8WB     .     Please Check
```

Author Response: This structure contains several disordered atoms, so that a clear assignement of al water molecules with the corresponding hydrogen atoms was not possible.

Alert level C

```
ABSTY02_ALERT_1_C  An _exptl_absorpt_correction_type has been given without
            a literature citation. This should be contained in the
            _exptl_absorpt_process_details field.
            Absorption correction given as multi-scan
DIFMX02_ALERT_1_C  The maximum difference density is > 0.1*ZMAX*0.75
            The relevant atom site should be identified.
PLAT041_ALERT_1_C Calc. and Reported SumFormula    Strings  Differ     Please Check
PLAT077_ALERT_4_C Unitcell Contains Non-integer Number of Atoms ..     Please Check
PLAT097_ALERT_2_C Large Reported Max.  (Positive) Residual Density       2.49 eA-3
PLAT213_ALERT_2_C Atom O5A             has ADP max/min Ratio .....        3.1 prolat
PLAT213_ALERT_2_C Atom O4B'            has ADP max/min Ratio .....        3.6 prolat
PLAT220_ALERT_2_C NonSolvent   Resd 1  C   Ueq(max)/Ueq(min) Range        5.1 Ratio
PLAT220_ALERT_2_C NonSolvent   Resd 1  O   Ueq(max)/Ueq(min) Range        3.6 Ratio
PLAT222_ALERT_3_C NonSolvent Resd 1  H   Uiso(max)/Uiso(min) Range        5.6 Ratio
PLAT241_ALERT_2_C High   'MainMol' Ueq as Compared to Neighbors of        O1M Check
PLAT314_ALERT_2_C Small Angle for H2O: Metal-O2W      -H2WB      .      77.10 Degree
PLAT911_ALERT_3_C Missing FCF Refl Between Thmin & STh/L=    0.600         86 Report
PLAT918_ALERT_3_C Reflection(s) with I(obs) much Smaller I(calc) .          1 Check
PLAT971_ALERT_2_C Check Calcd Resid. Dens.  0.85A   From O1M             2.17 eA-3
PLAT971_ALERT_2_C Check Calcd Resid. Dens.  0.75A   From O4A             1.91 eA-3
PLAT971_ALERT_2_C Check Calcd Resid. Dens.  0.85A   From Cu1B            1.51 eA-3
PLAT975_ALERT_2_C Check Calcd Resid. Dens.  1.04A   From O1W             1.36 eA-3
PLAT977_ALERT_2_C Check Negative Difference Density on H1MX             -0.51 eA-3
PLAT977_ALERT_2_C Check Negative Difference Density on H1WA             -0.51 eA-3
```

Alert level G

```
PLAT002_ALERT_2_G Number of Distance or Angle Restraints on AtSite         38 Note
PLAT003_ALERT_2_G Number of Uiso or Uij Restrained non-H Atoms ...        121 Report
PLAT004_ALERT_5_G Polymeric Structure Found with Maximum Dimension          2 Info
PLAT007_ALERT_5_G Number of Unrefined Donor-H Atoms .............           5 Report
PLAT045_ALERT_1_G Calculated and Reported Z Differ by a Factor ...       0.25 Check
PLAT083_ALERT_2_G SHELXL Second Parameter in WGHT  Unusually Large       5.47 Why ?
PLAT171_ALERT_4_G The CIF-Embedded .res File Contains EADP Records          1 Report
PLAT172_ALERT_4_G The CIF-Embedded .res File Contains DFIX Records         22 Report
```

```
PLAT175_ALERT_4_G The CIF-Embedded .res File Contains SAME Records          2 Report
PLAT177_ALERT_4_G The CIF-Embedded .res File Contains DELU Records          4 Report
PLAT178_ALERT_4_G The CIF-Embedded .res File Contains SIMU Records          4 Report
PLAT186_ALERT_4_G The CIF-Embedded .res File Contains ISOR Records          9 Report
PLAT300_ALERT_4_G Atom Site Occupancy of O1W        Constrained at       0.75 Check
PLAT300_ALERT_4_G Atom Site Occupancy of O2W        Constrained at       0.25 Check
PLAT300_ALERT_4_G Atom Site Occupancy of O3W        Constrained at       0.25 Check
PLAT300_ALERT_4_G Atom Site Occupancy of O5W        Constrained at       0.25 Check
PLAT300_ALERT_4_G Atom Site Occupancy of H1WA       Constrained at       0.75 Check
PLAT300_ALERT_4_G Atom Site Occupancy of H1WB       Constrained at       0.75 Check
PLAT300_ALERT_4_G Atom Site Occupancy of H2WA       Constrained at       0.25 Check
PLAT300_ALERT_4_G Atom Site Occupancy of H2WB       Constrained at       0.25 Check
PLAT300_ALERT_4_G Atom Site Occupancy of H3WA       Constrained at       0.25 Check
PLAT300_ALERT_4_G Atom Site Occupancy of H3WB       Constrained at       0.25 Check
PLAT300_ALERT_4_G Atom Site Occupancy of H5WA       Constrained at       0.25 Check
PLAT300_ALERT_4_G Atom Site Occupancy of H5WB       Constrained at       0.25 Check
PLAT300_ALERT_4_G Atom Site Occupancy of O8W        Constrained at        0.5 Check
PLAT300_ALERT_4_G Atom Site Occupancy of H8WA       Constrained at        0.5 Check
PLAT300_ALERT_4_G Atom Site Occupancy of H8WB       Constrained at        0.5 Check
PLAT300_ALERT_4_G Atom Site Occupancy of O9W        Constrained at        0.5 Check
PLAT300_ALERT_4_G Atom Site Occupancy of H9WA       Constrained at        0.5 Check
PLAT300_ALERT_4_G Atom Site Occupancy of H9WB       Constrained at        0.5 Check
PLAT300_ALERT_4_G Atom Site Occupancy of O4W        Constrained at       0.25 Check
PLAT300_ALERT_4_G Atom Site Occupancy of H4WA       Constrained at       0.25 Check
PLAT300_ALERT_4_G Atom Site Occupancy of H4WB       Constrained at       0.25 Check
PLAT300_ALERT_4_G Atom Site Occupancy of O6W        Constrained at       0.25 Check
PLAT300_ALERT_4_G Atom Site Occupancy of H6WA       Constrained at       0.25 Check
PLAT300_ALERT_4_G Atom Site Occupancy of O1W'       Constrained at       0.25 Check
PLAT300_ALERT_4_G Atom Site Occupancy of H1WC       Constrained at       0.25 Check
PLAT300_ALERT_4_G Atom Site Occupancy of H1WD       Constrained at       0.25 Check
PLAT300_ALERT_4_G Atom Site Occupancy of H6WB       Constrained at       0.25 Check
PLAT301_ALERT_3_G Main Residue  Disorder ..............(Resd  1  )        20% Note
PLAT302_ALERT_4_G Anion/Solvent/Minor-Residue Disorder (Resd  2  )       100% Note
PLAT302_ALERT_4_G Anion/Solvent/Minor-Residue Disorder (Resd  3  )       100% Note
PLAT302_ALERT_4_G Anion/Solvent/Minor-Residue Disorder (Resd  4  )       100% Note
PLAT302_ALERT_4_G Anion/Solvent/Minor-Residue Disorder (Resd  5  )       100% Note
PLAT302_ALERT_4_G Anion/Solvent/Minor-Residue Disorder (Resd  6  )       100% Note
PLAT303_ALERT_2_G Full Occupancy Atom H1MX      with # Connections       1.61 Check
PLAT720_ALERT_4_G Number of Unusual/Non-Standard Labels ..........         39 Note
PLAT764_ALERT_4_G Overcomplete CIF Bond List Detected (Rep/Expd) .       1.29 Ratio
PLAT778_ALERT_2_G Check O..H..X Bond in CIF: O6W      --H6WB             1.34 Ang.
PLAT779_ALERT_4_G Suspect or Irrelevant (Bond) Angle(s) in CIF . #        322 Check
              O7A  -S2A  -NA4'    1.555   1.555   1.445              34.47 Deg.
PLAT779_ALERT_4_G Suspect or Irrelevant (Bond) Angle(s) in CIF . #        326 Check
              O7A  -S2A  -NA1     1.555   1.555   2.667              38.47 Deg.
PLAT779_ALERT_4_G Suspect or Irrelevant (Bond) Angle(s) in CIF . #        463 Check
              O5B  -S1B  -NA1     1.555   1.555   1.555              33.14 Deg.
PLAT779_ALERT_4_G Suspect or Irrelevant (Bond) Angle(s) in CIF . #        477 Check
              O4B' -S1B' -NA1'    1.555   1.555   1.555              40.70 Deg.
PLAT779_ALERT_4_G Suspect or Irrelevant (Bond) Angle(s) in CIF . #        500 Check
              O8B  -S2B  -NA4     1.555   1.555   2.777              40.59 Deg.
PLAT779_ALERT_4_G Suspect or Irrelevant (Bond) Angle(s) in CIF . #        522 Check
              O8B' -S2B' -NA4'    1.555   1.555   2.777              40.20 Deg.
PLAT779_ALERT_4_G Suspect or Irrelevant (Bond) Angle(s) in CIF . #        653 Check
              NA1' -O1M  -H1MX    2.667   1.555   1.555              17.20 Deg.
PLAT779_ALERT_4_G Suspect or Irrelevant (Bond) Angle(s) in CIF . #        671 Check
              NA3  -O1W  -H1WA    1.665   1.555   1.555              11.00 Deg.
PLAT779_ALERT_4_G Suspect or Irrelevant (Bond) Angle(s) in CIF . #        685 Check
              NA1' -O3W  -H3WB    1.555   1.555   1.555              42.00 Deg.
PLAT780_ALERT_1_G Coordinates do not Form a Properly Connected Set     Please Do !
PLAT789_ALERT_4_G Atoms with Negative _atom_site_disorder_group  #          6 Check
PLAT860_ALERT_3_G Number of Least-Squares Restraints ............        1072 Note
```

```
PLAT883_ALERT_1_G No Info/Value for _atom_sites_solution_primary .     Please Do !
PLAT910_ALERT_3_G Missing # of FCF Reflection(s) Below Theta(Min).          2 Note
PLAT912_ALERT_4_G Missing # of FCF Reflections Above STh/L=  0.600        722 Note
PLAT913_ALERT_3_G Missing # of Very Strong Reflections in FCF ....          2 Note
PLAT933_ALERT_2_G Number of OMIT Records in Embedded .res File ...          1 Note
PLAT941_ALERT_3_G Average HKL Measurement Multiplicity ...........        2.5 Low
PLAT978_ALERT_2_G Number C-C Bonds with Positive Residual Density.          1 Info
```

```
   0 ALERT level A = Most likely a serious problem - resolve or explain
   2 ALERT level B = A potentially serious problem, consider carefully
  20 ALERT level C = Check. Ensure it is not caused by an omission or oversight
  68 ALERT level G = General information/check it is not something unexpected

   6 ALERT type 1 CIF construction/syntax error, inconsistent or missing data
  22 ALERT type 2 Indicator that the structure model may be wrong or deficient
   8 ALERT type 3 Indicator that the structure quality may be low
  52 ALERT type 4 Improvement, methodology, query or suggestion
   2 ALERT type 5 Informative message, check
```

Datablock: CuL7

Bond precision: C-C = 0.0048 A Wavelength=0.71073

Cell:	a=36.8856(14)	b=11.7527(5)	c=17.2070(6)
	alpha=90	beta=112.251(1)	gamma=90
Temperature:	100 K		

	Calculated	Reported
Volume	6903.9(5)	6903.9(5)
Space group	C 2/c	C 2/c
Hall group	-C 2yc	-C 2yc
Moiety formula	C32 H25.81 Cu N4 Na1.67 O11.90 S2, 1.598(H2 O), 0.328(Na)	?
Sum formula	C32 H29 Cu N4 Na2 O13.50 S2	C32 H29 Cu N4 Na2 O13.50 S2
Mr	859.26	859.23
Dx,g cm-3	1.653	1.653
Z	8	8
Mu (mm-1)	0.855	0.855
F000	3520.1	3520.0
F000’	3526.73	
h,k,lmax	54,17,25	53,17,25
Nref	11556	11052
Tmin,Tmax	0.814,0.991	0.669,0.746
Tmin’	0.774	

Correction method= # Reported T Limits: Tmin=0.669 Tmax=0.746
AbsCorr = MULTI-SCAN

```
Data completeness= 0.956          Theta(max)= 31.547

R(reflections)= 0.0635( 8612)     wR2(reflections)= 0.1935( 11052)

S = 1.041                   Npar= 564
```

The following ALERTS were generated. Each ALERT has the format
test-name_ALERT_alert-type_alert-level.
Click on the hyperlinks for more details of the test.

Alert level B

```
PLAT420_ALERT_2_B D-H Without Acceptor       O4W      --H4WA     .     Please Check
```

Author Response: In this structure one of the Na-atoms present and one water molecule are disordered in three positions. Due to this diffuse area of electron densities, some of the hydrogen atoms of water molecules are without acceptor.

Alert level C

```
DIFMX02_ALERT_1_C  The maximum difference density is > 0.1*ZMAX*0.75
            The relevant atom site should be identified.
PLAT097_ALERT_2_C Large Reported Max.  (Positive) Residual Density       2.21 eA-3
PLAT213_ALERT_2_C Atom O8              has ADP max/min Ratio .....        3.1 prolat
PLAT220_ALERT_2_C NonSolvent   Resd 1  O   Ueq(max)/Ueq(min) Range        3.9 Ratio
PLAT241_ALERT_2_C High   'MainMol' Ueq as Compared to Neighbors of        O6 Check
PLAT314_ALERT_2_C Small Angle for H2O: Metal-O2W      -H2WA      .      44.95 Degree
PLAT911_ALERT_3_C Missing FCF Refl Between Thmin & STh/L=    0.600         33 Report
PLAT971_ALERT_2_C Check Calcd Resid. Dens.  1.04A   From S1            1.93 eA-3
PLAT971_ALERT_2_C Check Calcd Resid. Dens.  0.64A   From S2            1.60 eA-3
PLAT976_ALERT_2_C Check Calcd Resid. Dens.  0.54A   From O3W          -1.14 eA-3
PLAT976_ALERT_2_C Check Calcd Resid. Dens.  0.53A   From O3W          -0.91 eA-3
PLAT976_ALERT_2_C Check Calcd Resid. Dens.  0.65A   From O2W          -0.76 eA-3
PLAT976_ALERT_2_C Check Calcd Resid. Dens.  0.86A   From O8           -0.65 eA-3
PLAT976_ALERT_2_C Check Calcd Resid. Dens.  0.73A   From O4W          -0.57 eA-3
PLAT976_ALERT_2_C Check Calcd Resid. Dens.  0.57A   From O4W          -0.48 eA-3
PLAT977_ALERT_2_C Check Negative Difference Density on H3WA           -0.76 eA-3
PLAT977_ALERT_2_C Check Negative Difference Density on H4WA           -0.47 eA-3
PLAT977_ALERT_2_C Check Negative Difference Density on H5WA           -0.38 eA-3
```

Alert level G

```
PLAT002_ALERT_2_G Number of Distance or Angle Restraints on AtSite         16 Note
PLAT003_ALERT_2_G Number of Uiso or Uij Restrained non-H Atoms ...          5 Report
PLAT004_ALERT_5_G Polymeric Structure Found with Maximum Dimension          2 Info
PLAT007_ALERT_5_G Number of Unrefined Donor-H Atoms ..............          6 Report
PLAT083_ALERT_2_G SHELXL Second Parameter in WGHT  Unusually Large      31.11 Why ?
PLAT128_ALERT_4_G Alternate Setting for Input Space Group     C2/c       I2/a Note
PLAT172_ALERT_4_G The CIF-Embedded .res File Contains DFIX Records         18 Report
PLAT186_ALERT_4_G The CIF-Embedded .res File Contains ISOR Records          3 Report
PLAT300_ALERT_4_G Atom Site Occupancy of H3WA       Constrained at        0.5 Check
PLAT300_ALERT_4_G Atom Site Occupancy of H3WB       Constrained at        0.5 Check
PLAT301_ALERT_3_G Main Residue  Disorder ..............(Resd  1  )         2% Note
PLAT302_ALERT_4_G Anion/Solvent/Minor-Residue Disorder (Resd  3  )       100% Note
```

```
PLAT302_ALERT_4_G Anion/Solvent/Minor-Residue Disorder (Resd  4  )       100% Note
PLAT302_ALERT_4_G Anion/Solvent/Minor-Residue Disorder (Resd  5  )       100% Note
PLAT303_ALERT_2_G Full Occupancy Atom H2WA      with # Connections       2.00 Check
PLAT303_ALERT_2_G Full Occupancy Atom H4WA      with # Connections       1.24 Check
PLAT720_ALERT_4_G Number of Unusual/Non-Standard Labels ..........         18 Note
PLAT764_ALERT_4_G Overcomplete CIF Bond List Detected (Rep/Expd) .       1.12 Ratio
PLAT779_ALERT_4_G Suspect or Irrelevant (Bond) Angle(s) in CIF . #         34 Check
              NA2' -O2   -NA2"    6.555   1.555   6.555             40.40 Deg.
PLAT780_ALERT_1_G Coordinates do not Form a Properly Connected Set     Please Do !
PLAT794_ALERT_5_G Tentative Bond Valency for Cu1       (I)       .       1.30 Info
PLAT860_ALERT_3_G Number of Least-Squares Restraints .............         50 Note
PLAT883_ALERT_1_G No Info/Value for _atom_sites_solution_primary .     Please Do !
PLAT910_ALERT_3_G Missing # of FCF Reflection(s) Below Theta(Min).          1 Note
PLAT912_ALERT_4_G Missing # of FCF Reflections Above STh/L=  0.600        467 Note
PLAT933_ALERT_2_G Number of OMIT Records in Embedded .res File ...          3 Note
PLAT941_ALERT_3_G Average HKL Measurement Multiplicity ...........        3.4 Low
PLAT965_ALERT_2_G The SHELXL WEIGHT Optimisation has not Converged     Please Check
PLAT978_ALERT_2_G Number C-C Bonds with Positive Residual Density.          2 Info
```

```
   0 ALERT level A = Most likely a serious problem - resolve or explain
   1 ALERT level B = A potentially serious problem, consider carefully
  18 ALERT level C = Check. Ensure it is not caused by an omission or oversight
  29 ALERT level G = General information/check it is not something unexpected

   3 ALERT type 1 CIF construction/syntax error, inconsistent or missing data
  25 ALERT type 2 Indicator that the structure model may be wrong or deficient
   5 ALERT type 3 Indicator that the structure quality may be low
  12 ALERT type 4 Improvement, methodology, query or suggestion
   3 ALERT type 5 Informative message, check
```

Datablock: H2L5

Bond precision:	C-C = 0.0041 A		Wavelength=0.71073
Cell:	a=14.4650(14)	b=8.5135(9)	c=15.7483(14)
	alpha=90	beta=109.155(3)	gamma=90
Temperature:	100 K		

	Calculated	Reported
Volume	1832.0(3)	1832.0(3)
Space group	P 21/c	P2(1)/c
Hall group	-P 2ybc	-P 2ybc
Moiety formula	C24 H16 N4 O8 S2, 2(C6 H16 N)	?
Sum formula	C36 H48 N6 O8 S2	C36 H48 N6 O8 S2
Mr	756.92	756.92
Dx,g cm-3	1.372	1.372
Z	2	2
Mu (mm-1)	0.206	0.206
F000	804.0	804.0
F000'	804.84	
h,k,lmax	17,10,18	17,10,18
Nref	3380	3343
Tmin,Tmax	0.988,0.990	0.541,0.990
Tmin'	0.940	

Correction method= # Reported T Limits: Tmin=0.541 Tmax=0.990
AbsCorr = MULTI-SCAN

Data completeness= 0.989 Theta(max)= 25.391

R(reflections)= 0.0493(2165) wR2(reflections)= 0.1269(3343)

S = 0.999 Npar= 238

The following ALERTS were generated. Each ALERT has the format
test-name_ALERT_alert-type_alert-level.
Click on the hyperlinks for more details of the test.

Alert level C

```
PLAT340_ALERT_3_C Low Bond Precision on  C-C Bonds ...............    0.00413 Ang.
PLAT911_ALERT_3_C Missing FCF Refl Between Thmin & STh/L=    0.600         34 Report
```

Alert level G

```
PLAT007_ALERT_5_G Number of Unrefined Donor-H Atoms ..............          2 Report
PLAT720_ALERT_4_G Number of Unusual/Non-Standard Labels ..........         15 Note
PLAT883_ALERT_1_G No Info/Value for _atom_sites_solution_primary .     Please Do !
PLAT910_ALERT_3_G Missing # of FCF Reflection(s) Below Theta(Min).          1 Note
PLAT912_ALERT_4_G Missing # of FCF Reflections Above STh/L=  0.600          3 Note
PLAT941_ALERT_3_G Average HKL Measurement Multiplicity ...........        4.1 Low
PLAT978_ALERT_2_G Number C-C Bonds with Positive Residual Density.          0 Info
```

---

0 **ALERT level A** = Most likely a serious problem - resolve or explain
0 **ALERT level B** = A potentially serious problem, consider carefully
2 **ALERT level C** = Check. Ensure it is not caused by an omission or oversight
7 **ALERT level G** = General information/check it is not something unexpected

1 ALERT type 1 CIF construction/syntax error, inconsistent or missing data

```
  1 ALERT type 2 Indicator that the structure model may be wrong or deficient
  4 ALERT type 3 Indicator that the structure quality may be low
  2 ALERT type 4 Improvement, methodology, query or suggestion
  1 ALERT type 5 Informative message, check
```

It is advisable to attempt to resolve as many as possible of the alerts in all categories. Often the minor alerts point to easily fixed oversights, errors and omissions in your CIF or refinement strategy, so attention to these fine details can be worthwhile. In order to resolve some of the more serious problems it may be necessary to carry out additional measurements or structure refinements. However, the purpose of your study may justify the reported deviations and the more serious of these should normally be commented upon in the discussion or experimental section of a paper or in the "special_details" fields of the CIF. checkCIF was carefully designed to identify outliers and unusual parameters, but every test has its limitations and alerts that are not important in a particular case may appear. Conversely, the absence of alerts does not guarantee there are no aspects of the results needing attention. It is up to the individual to critically assess their own results and, if necessary, seek expert advice.

Publication of your CIF in IUCr journals

A basic structural check has been run on your CIF. These basic checks will be run on all CIFs submitted for publication in IUCr journals (*Acta Crystallographica*, *Journal of Applied Crystallography*, *Journal of Synchrotron Radiation*); however, if you intend to submit to *Acta Crystallographica Section C* or *E* or *IUCrData*, you should make sure that full publication checks are run on the final version of your CIF prior to submission.

Publication of your CIF in other journals

Please refer to the *Notes for Authors* of the relevant journal for any special instructions relating to CIF submission.

PLATON version of 10/08/2020; check.def file version of 06/08/2020

Datablock CuL1 - ellipsoid plot

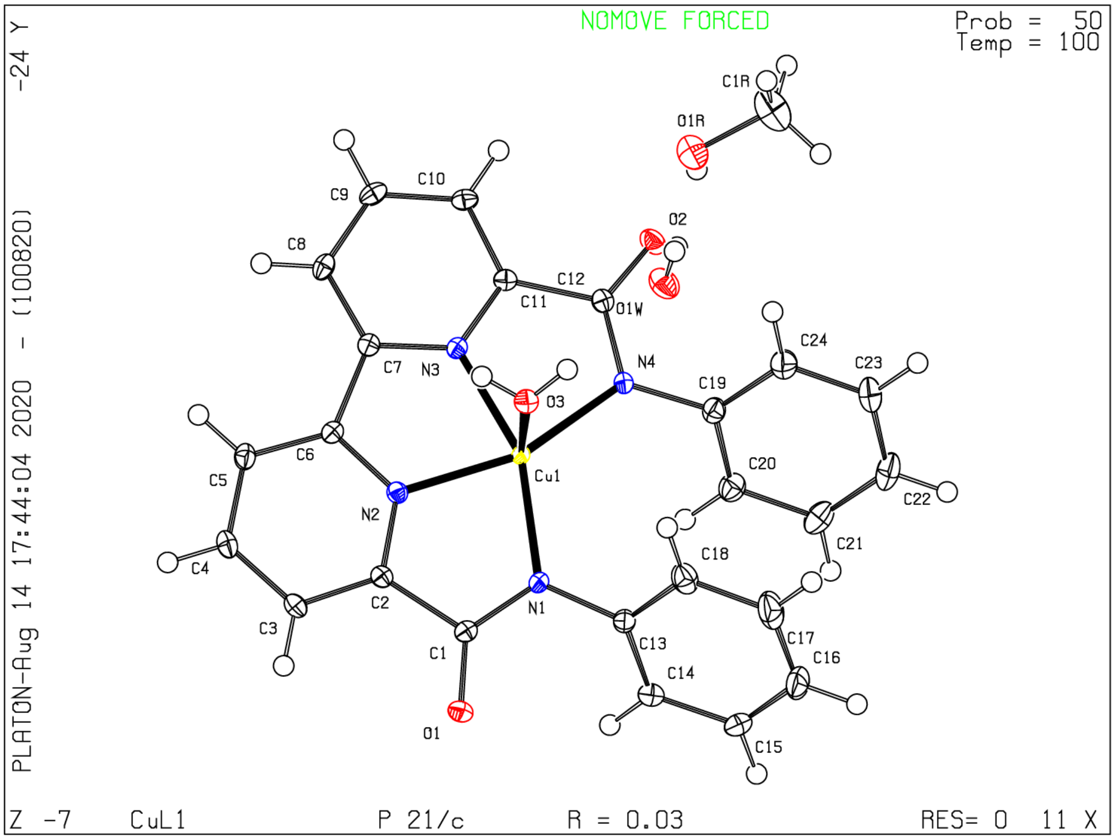

Datablock CuL2 - ellipsoid plot

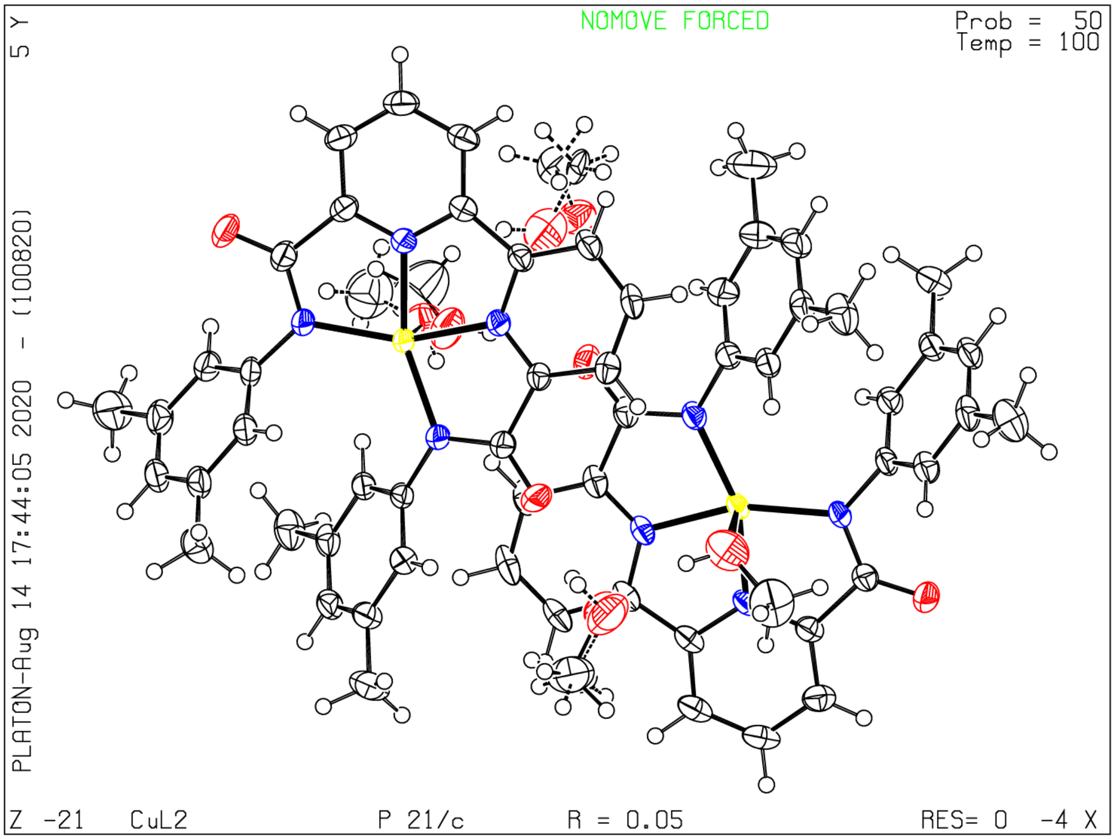

Datablock CuL3 - ellipsoid plot

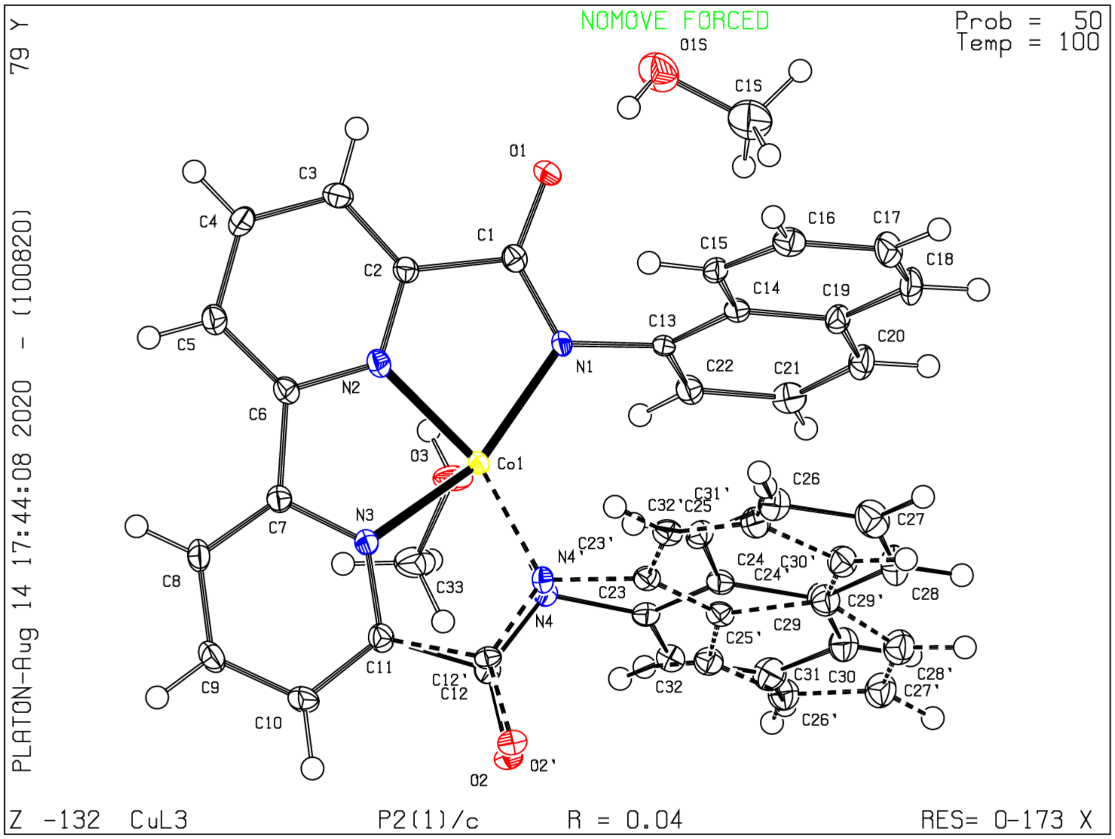

Datablock CuL4 - ellipsoid plot

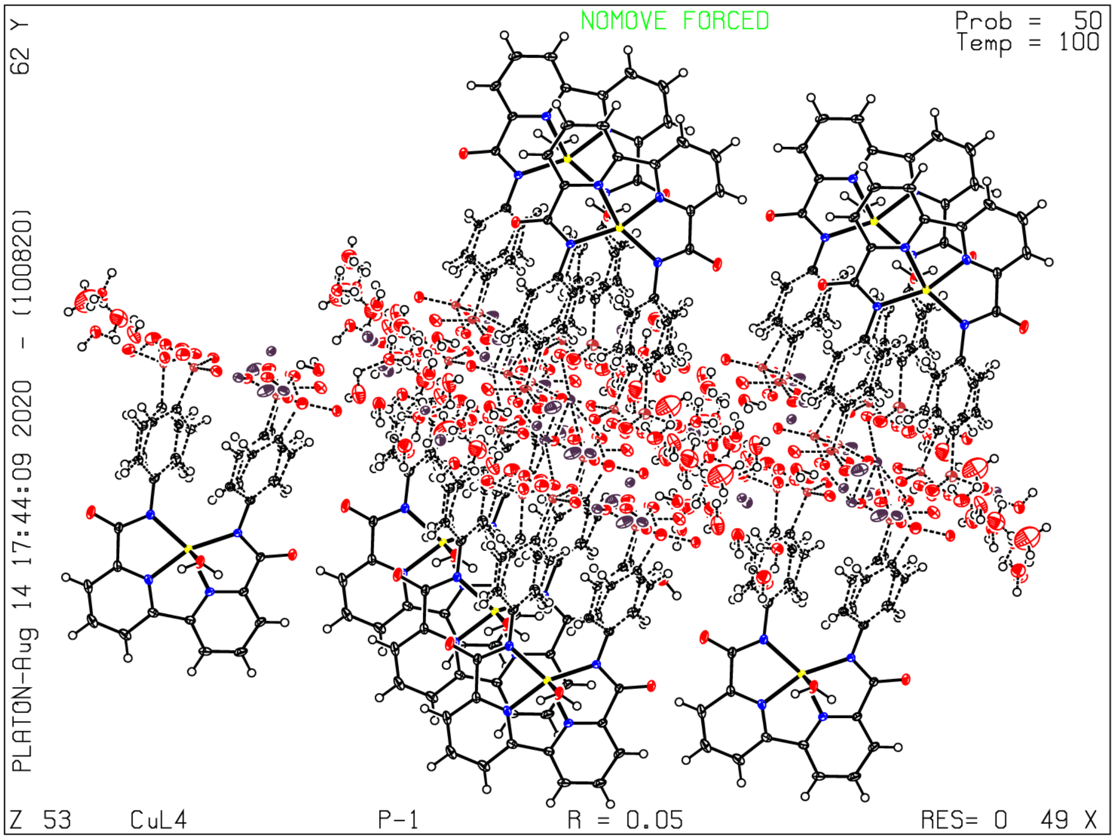

Datablock CuL5 - ellipsoid plot

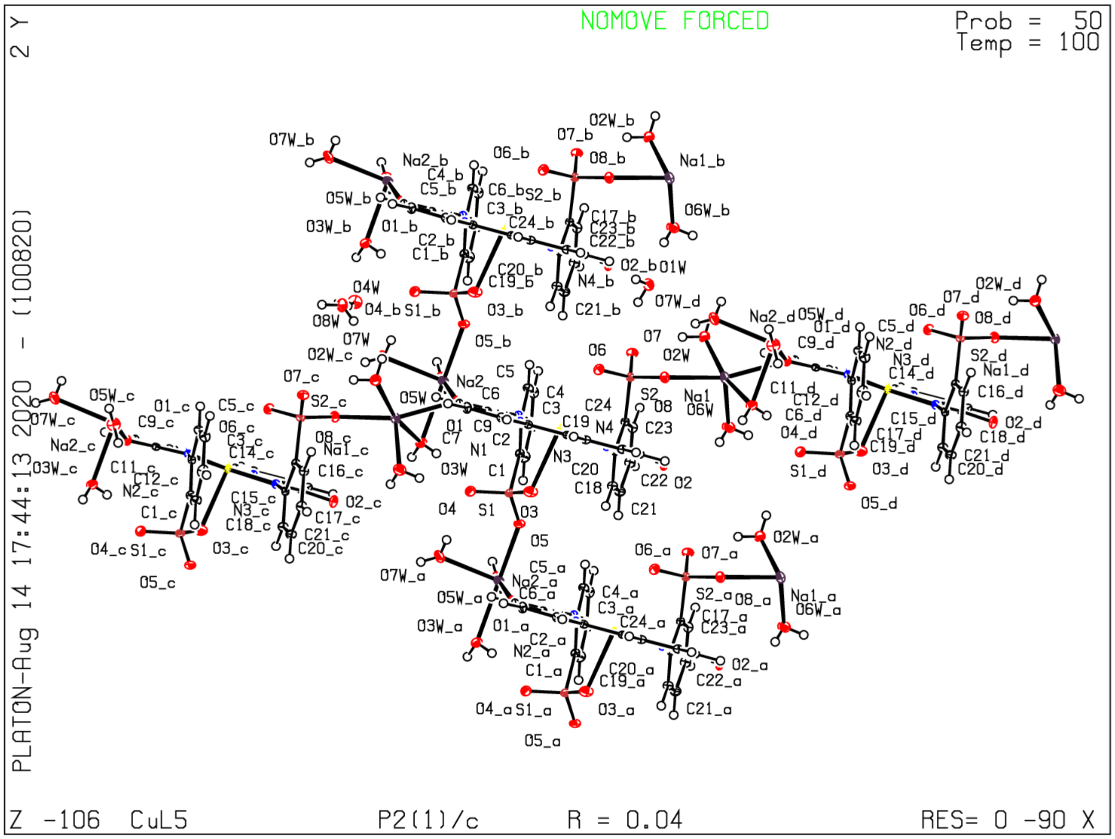

Datablock CuL6 - ellipsoid plot

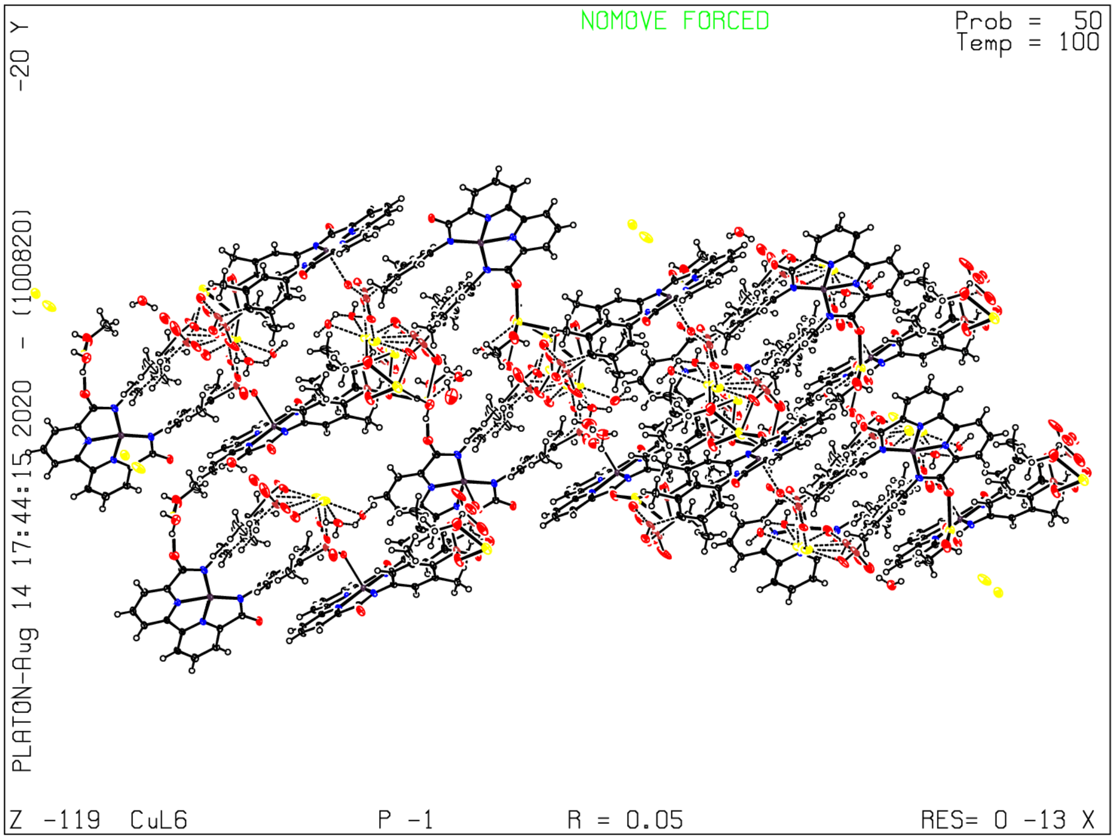

Datablock CuL7 - ellipsoid plot

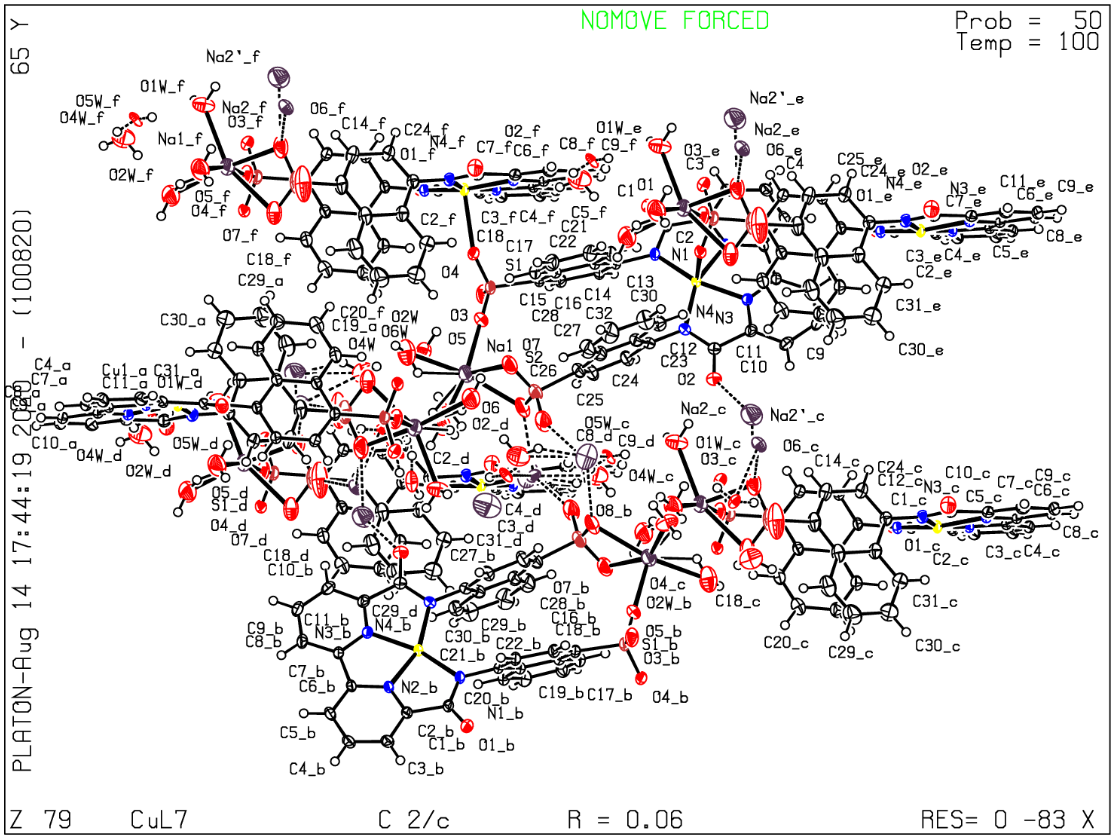

Datablock H2L5 - ellipsoid plot

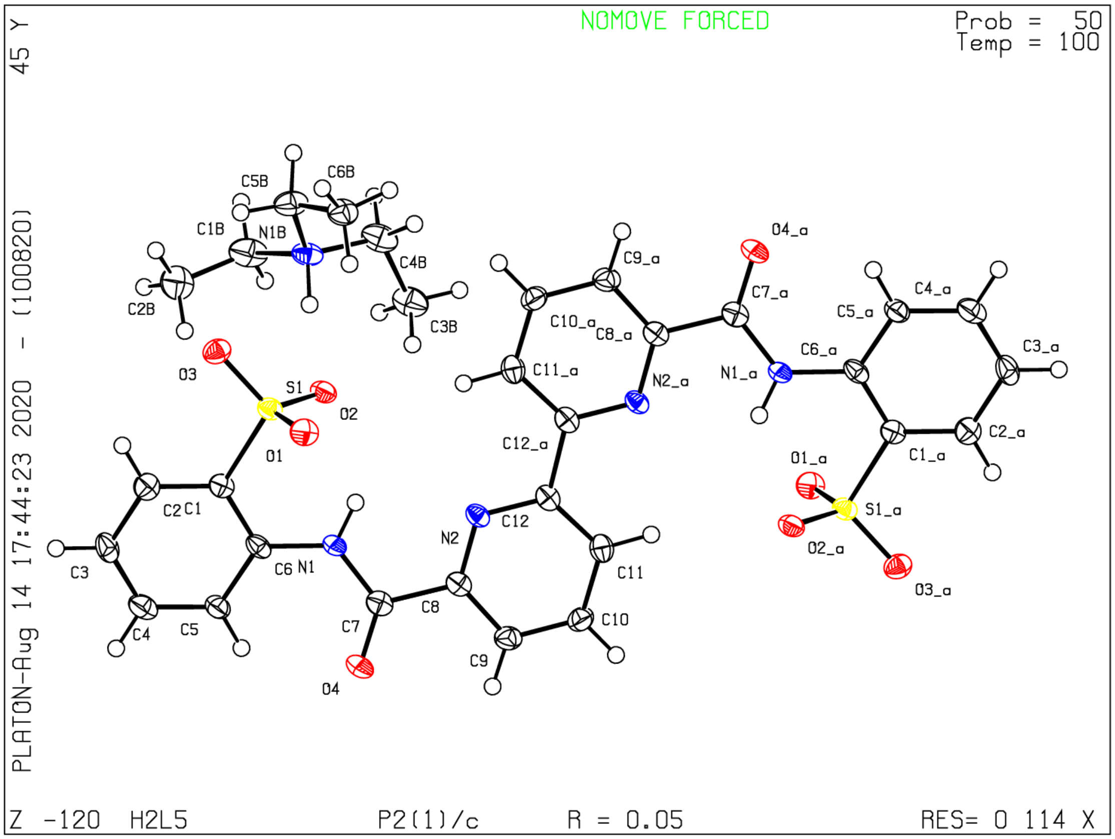

Supplement: Supplementary file 1 — Supporting Information [file ANIE-60-18639-s001.zip › checkcif_CuL.pdf]
