# Supplementary material for: Consecutive Ligand‐Based Electron Transfer in New Molecular Copper‐Based Water Oxidation Catalysts
Source: Angew Chem Int Ed Engl. 2021 Jul 16;60(34):18639–44. doi: 10.1002/anie.202104020 (PMC8456863; doi:10.1002/anie.202104020)
Supplement: Supplementary file 2 — Supporting Information [file ANIE-60-18639-s002.pdf]

Angewandte Chemie
Eine Zeitschrift der Gesellschaft Deutscher Chemiker

Supporting Information

Consecutive Ligand-Based Electron Transfer in New Molecular Copper-Based Water Oxidation Catalysts

*Marcos Gil-Sepulcre+, Pablo Garrido-Barros+, Jan Oldengott, Ignacio Funes-Ardoiz, Roger Bofill, Xavier Sala, Jordi Benet-Buchholz, and Antoni Llobet**

anie_202104020_sm_miscellaneous_information.pdf

Supporting Information

Table of Contents

Experimental Procedures....2

- Materials....2
- Instrumentation and measurements....2
- Electrochemical measurements....2
- O_2 evolution experiments....3
- Foot of the Wave Analysis (FOWA). k_{obs} Calculation....3
- TON Calculation....3
- Synthesis of the ligands....3
- Synthesis of Cu-complexes....7
- Synthesis of complex [(L4)Zn](Na)$_2$·4H_2O.....8

Spectroscopic Characterization....9

- NMR Spectroscopy....9
- Mass-spectrometry....31
- UV-Vis spectroscopy....39

X-Ray Crystallography....40

- Crystal preparation:....40

Electron Paramagnetic Resonance (EPR)....45

Electrochemical behavior in organic solvents....46

Electrochemical behavior in water....47

Determination of the kinetic constant. Foot of the Wave Analysis (FOWA).....53

O_2 Evolution experiments.....56

Scanning Electron Microscopy....58

Computational studies....60

Cartesian coordinates (Å) and calculated potential energies (atomic units)....71

References....91

Experimental Procedures

Materials

All general reagents and chemicals were used as purchased from Sigma-Aldrich, Fluka and Merck chemical companies without further purification unless otherwise stated. The ligand precursor ([2,2'-bipyridine]-6,6'-dicarboxylic acid) and 5-amino-*m*-xylol-4-sulfonic acid were prepared according to the experimental procedure reported in the literature.[1,2] Air and moisture sensitive reactions were carried out under N_2 or Ar in oven-dried (120 °C) glassware. Evaporation of solvents *in vacuo* was done with a *Büchi Rotevapor R-200* at 40 °C.

Instrumentation and measurements

NMR spectra were measured on a *Bruker AV-500, Bruker AV-400,* and *Bruker 300 MHz* spectrometers. All NMR experiments were performed at room temperature in corresponding deuterated solvents and using solvent signals as reference. UV-Vis spectra were measured on a *Cary 50 UV-vis* spectrometer by *Varian Inc*. Electrospray ionization mass spectra (ESI-MS) were performed on an *Agilent Technologies 6130-Quadrupole LC/MS* connected to an *Agilent Technologies HPLC-1200 series*. Samples were dissolved in MeOH or H_2O and injected directly with an auto-sampler. HRMS samples were measured on a Bruker HPLC-QqTOF Maxis impact device. Elemental Analysis of the samples was carried out in a Thermo Finnigan elemental analyzer Flash 1112 model. Electron Paramagnetic Resonance was performed in an EMX Micro X-band EPR spectrometer (Bruker) at 77 K using a liquid N2 finger dewar. Data acquisition: perpendicular mode, modulation frequency of 100 KHz, microwave frequency of 9.38 GHz, modulation amplitude of 4 G, a 0.01 ms time constant and 4.12 ms conversion time with a microwave power of 0.556 mW.

Electrochemical measurements

Differential pulse voltammetry (DPV) and cyclic voltammetry (CV) were measured on a CHI660D potentiostat using a three-electrode cell. Glassy carbon (GC), Carbon paper (C-paper, SGL Carbon, Sigracet 39 AA) or boron-doped diamond (BDD) (d = 3 mm) working electrodes were employed while a Pt rod/mesh was used as counter electrode and a Hg/$HgSO_4$ (K_2SO_4 sat.) or Ag/AgCl (KCl sat.) electrode were used as a reference electrode. Working electrodes were polished with 1 and 0.05 micron alumina paste, washed with distilled water and acetone, and sonicated in acetone for 5 minutes before each measurement. DMF employed for electrochemical measurements was prepared containing the necessary amount of *n*-Bu_4NPF_6 ($TBAPF_6$) as supporting electrolyte to yield a solution of 0.1 M ionic strength. CVs were typically recorded at different scan rates from 25 to 1000 mV/s. DPVs were recorded with the following parameters: amplitude = 50 mV, step height = 4 mV, pulse width = 0.05 s. All redox potentials in the present work are reported versus NHE by adding 0.648 V to the measured potential.

O_2 evolution experiments

Controlled Potential Electrolysis (CPE) experiments were performed at different potentials and different pH values to catalyze the water oxidation reaction by the complexes by using a two-compartment cell closed with a septum. As working electrode large surface BDD electrodes (rectangular shape with 1.5 cm^2 surface) were used together with a silver/silver chloride (KCl sat.) as a reference electrode. These ones were placed in one of the compartments that was filled with a 1.5 mM solution of the complex (phosphate buffer pH 7, borate buffer pH 9 or phosphate buffer pH 11.6, of 0.1 M ionic strength). In the other compartment, containing only the buffer solution, a mesh platinum counter electrode was used.
The oxygen evolution was monitored with an OXNP type Clark electrode in gas phase (from Unisense Company). The CPE was carried out using an IJ-Cambria CHI-660 potentiostat and was started as soon as the oxygen sensor signal was stable under air atmosphere. During the experiment, solutions of both compartments were vigorously stirred. Calibration of the oxygen sensor was performed after each experiment by adding known amounts of pure oxygen into the cell using a Hamilton syringe. The results of the water oxidation catalysis with the complexes were compared with blank experiments under the same conditions but in the absence of the complex. The Faradaic efficiency was determined according to the total charge passed during the CPE and the total amount of generated oxygen by considering that water oxidation is a $4e^-$ oxidation process.

Foot of the Wave Analysis (FOWA). k_{obs} Calculation

Under catalytic conditions FOWA equation is operative.

$$\frac{i}{i_p} = \frac{n \cdot 2.24 \cdot \sqrt{\frac{R \cdot T}{F \cdot v}} \cdot k_{obs}}{1+\exp[\frac{F}{RT}(E^o{}_{cat} - E)]} \qquad \text{Equation (1)}$$

where k_{obs} is the apparent WNA pseudo-rate constant (k[H_2O]), E^0_{cat} corresponds to the standard potential for the catalytic wave (E^0_{cat} according to the DPVs shown in Figure S46), i is the current in the presence of substrate, i_p corresponds to the peak current of one-electron redox process of the catalyst (extracted from the Cu^{II}/Cu^{I} couple when available), F is the faradaic constant (96485 C mol^{-1}), T is the temperature (298 K), v is the scan rate (100 mV s^{-1}) and R is 8.314 J mol^{-1} K^{-1}.[3]

TON Calculation

The total TON values can be obtained from the oxygen evolution experiment taking in account the total amount of catalyst present in the solution by using equation (2). However, since only the catalyst present in the layer of the solution in contact with the electrode is involved in the water oxidation reaction, this TON value is underestimated. Lin and co-workers adapted it to the formula (3) based on the previous methodology developed by Savéant and co-workers, which gives a more realistic TON value based on the amount catalyst in contact with the electrode.[4]

$$TON = \frac{\mu mol\ produced\ O_2}{\mu mol\ cat.} \qquad \text{Equation (2)}$$

$$TON = \frac{k_{obs} t}{1+\exp[\frac{F}{RT}(E^o{}_{cat} - E)]} \qquad \text{Equation (3)}$$

Synthesis of the ligands

The general procedure for the synthesis of ligands, [H_2L1], [H_2L3], [H_2L4]$^{2-}$ and [H_2L5]$^{2-}$ was adapted from the literature[5] as follows: 500 mg (2.05 mmol) of [2,2'-bipyridine]-6,6'-dicarboxylic acid were suspended in 20 mL of $SOCl_2$ and the mixture was refluxed at 85 °C under a nitrogen atmosphere during 6 hours. After complete dissolution of the reactant, $SOCl_2$ was completely removed under vacuum, yielding a white powder corresponding to the acyl chloride derivative. The white solid was re-suspended in 40 mL of dry DCM and the temperature was decreased until 0 °C using an ice bath. Then, 4 eq. of NEt_3 were added dropwise and stirred for 10 minutes. Finally, a previously prepared dispersion of the corresponding phenylamine or naphthylamine (4.1 mmol, 2.0 eq.) in 40 mL of dry DCM were added dropwise to the reaction volume and the mixture was vigorous stirred for 72 h at room temperature. The appearing solid consisted in the corresponding ligand ([H_2L1], [H_2L3], [H_2L4]$^{2-}$ and [H_2L5]$^{2-}$), which was filtered and washed with DCM and Et_2O, yielding the desired product without further purification.
In case of ligands [H_2L2], [H_2L6]$^{2-}$, [H_2L7]$^{2-}$ and [H_2L8]$^{2-}$, the general procedure was adapted as follows. The acyl chloride derivative was re-dissolved in 20 mL of dry DMA and added dropwise to a dispersion of the corresponding amine (4 mmol, 2 eq.) in 8 mL of dry DMA containing 4 eq. of Et_3N. This solution was stirred under a nitrogen atmosphere for 15 h at 50 °C. The appearing solid consisted in the corresponding ligand ([H_2L6]$^{2-}$, [H_2L7]$^{2-}$ and [H_2L8]$^{2-}$) which was filtered and washed with acetone and Et_2O, yielding the desired product

without further purification. In case of [H_2L2], the reaction mixture showed no precipitate after 4 days of reaction. The clean solution was mixed with 0.1 M Na_2CO_3 solution, extracted with DCM, dried over $MgSO_4$, and evaporated to yield [H_2L2] as a white powder.

[H₂L1]*·0.8 H₂O*

Yield: 695 mg, 1.75 mmol, 86 %. ^{1}H NMR (DMSO-d$_6$): *δ* [ppm] = 10.66 (H_4, s, 2H), 9.22 (H_1, dd, *J* = 6.9, 2.1 Hz, 2H), 8.29 ($H_{2\text{-}3}$, m, 4H), 7.93 (H_5, dd, *J* = 8.71, 1.2 Hz, 4H), 7.4 (H_6, dd, *J* = 8.7 Hz, 4H), 7.16 (H_7, tt, *J* = 7.4, 1.2 Hz, 2H). ^{13}C NMR (DMSO-d$_6$): 162.9 (C_6), 153.9 (C_5), 150.2 (C_1), 139.7 (C_4), 138.6 (C_7), 129.1 (C_9), 125.3 (C_2), 124.7 (C_{10}), 123.6 (C_3), 121.6 (C_8). ESI-MS (MeOH) m/z positive mode: 417.0 [H_2L1+Na$^+$]$^+$. Elemental analysis (% found): C, 70.36; H, 4.86; N, 13.79. Calcd. for $C_{24}H_{18}N_4O_2$·0.8 H_2O: C, 70.51; H, 4.83; N, 13.70. IR ($\tilde{\nu}$ / cm^{-1}): 3421m, 3347s, 1663m, 1598m, 1530s, 1498w, 1448m, 14312m, 1325m, 1244w, 1077m, 994w, 756m, 687m, 664m, 494w.

[H₂L2]*·0.3 H₂O*

Yield: 453 mg, 1.00 mmol, 49%. ^{1}H NMR (500 MHz, $CDCl_3$): *δ* [ppm] = 9.92 (H_4, s, 2H), 8.62 (H_1, dd, *J* = 7.9, 1.0 Hz, 2H), 8.42 (H_3, d, *J* = 7.7, 1.0 Hz, 2H), 8.16 (H_2, t, *J* = 7.8 Hz, 2H), 7.46 (H_5, s, 4H), 6.84 (H_7, s, 2H), 2.38 (H_6, s, 12H). ^{13}C NMR (126 MHz, $CDCl_3$): *δ* [ppm] = 161.7 (C_6), 153.7 (C_1), 149.9 (C_5), 139.1 (C_3, C_9), 137.5 (C_7), 126.6 (C_{11}), 123.9 (C_2), 123.2 (C_4), 117.8 (C_8), 21.6 (C_{10}). ESI-HRMS ($CHCl_3$/MeOH) m/z positive mode: calcd for [H_2L2-H$^+$]$^-$, ($C_{28}H_{25}N_4O_2$) :449.1983, found: 449.1981. Elemental analysis (% found): C, 73.39; H, 5.54; N, 12.55. Calcd for $C_{28}H_{26}N_4O_2$·0.3 H_2O: C: 73.76; H: 5.88; N: 12.29. IR ($\tilde{\nu}$ / cm^{-1}): 3352s, 3095w, 3015w, 2914m, 2856w, 1683s, 1611m, 1581m, 1553s, 1417s, 1237w, 1178w, 1075w, 996w, 834m, 758m, 685m, 633m.

[H₂L3]*·0.5 H₂O*

Yield: 1.40 g, 1.42 mmol, 71%. ^{1}H NMR (400MHz, $CDCl_3$): *δ* [ppm] = 10.74 (H_4, s, 2H), 8.78 (H_1, dd, *J* = 7.9, 1.0 Hz, 2H), 8.52 (H_3, dd, *J* = 7.7, 1.0 Hz, 2H), 8.40 (d, *J* = 6.8 Hz, 2H), 8.23 (H_2, t, *J* = 7.8 Hz, 2H), 8.13 (d, *J* = 8.5 Hz, 2H), 7.95 (d, *J* = 8.7 Hz, 2H), 7.77 (d, *J* = 8.2 Hz, 2H), 7.66 – 7.53 ($H_{6,9,10}$, m, 6H). HRMS-ESI: Calcd for [H_2L3+Na$^+$]$^+$, ($C_{32}H_{22}N_4O_2$Na): 517.1635, found: 517.1625. Elemental analysis (% found): C, 76.2; H, 4.5; N, 11.1. Calcd for $C_{32}H_{22}N_4O_2$·0.5 H_2O: C, 76.3; H, 4.6; N, 11.1. IR ($\tilde{\nu}$ / cm^{-1}): 3370s, 3356s, 3055m, 3012w, 1685s, 1584m, 1536s, 1496s, 1430m, 1405m, 1344m, 1255m, 1133m, 1068m, 996w, 792m, 770m, 735m, 631m.

[H₂L4]*(Et₃NH)₂·2 H₂O*

Yield: 931 mg, 1.68 mmol, 61%. ^{1}H NMR (DMSO-d$_6$): *δ* [ppm] = 10.70 (H_4, s, 2H), 9.24 (H_1, dd, *J* = 7.3, 1.6 Hz, 2H), 8.30 ($H_{2\text{-}3}$, m, 4H), 7.90 (H_5, d, *J* = 8.7 Hz, 4H), 7.65 (H_6, d, *J* = 8.7 Hz, 4H). ^{13}C NMR (DMSO-d$_6$): 162.9 (C_6), 153.9 (C_5), 150.0 (C_1), 144.7 (C_{10}), 139.8 (C_4), 138.6 (C_7), 126.5 (C_9), 125.3 (C_2), 123.7 (C_3), 120.4 (C_8). ESI-MS (MeOH) m/z negative mode: 553.0 [H_2L4+H$^+$]$^-$, 276.1 [H_2L4]$^{2-}$. Elemental analysis (% found): C, 54.6; H, 6.7; N, 10.6. Calcd for $C_{24}H_{16}N_4O_8S_2^{2-}$·2 Et_3NH^+·2 H_2O: C, 54.5; H, 6.6; N, 10.6. IR ($\tilde{\nu}$ / cm^{-1}): 3333m, 3316m, 3055w, 2933w, 1690s, 1583m, 1522s, 1434m, 1398m, 1275m, 1230w, 1166m, 1126m, 1073m, 893w, 826m, 681m, 548m.

[H₂L5]*(Et₃NH)₂·1.3 H₂O*

Yield: 965 mg, 1.74 mmol, 64%. ^{1}H NMR (DMSO-d$_6$): *δ* [ppm] = 12.55 (H_4, s, 2H), 9.51 (H_1, dd, *J* = 7.3, 1.7 Hz, 2H), 8.67 (H_5, dd, *J* = 8.2, 1.1 Hz, 2H), 8.26 ($H_{2\text{-}3}$, m, 4H), 7.82 (H_8, dd, *J* = 7.7, 1.7 Hz, 2H), 7.43 (H_6, t, *J* = 1.2 Hz, 2H), 7.14 (H_7, td, *J* = 7.5, 1.2 Hz, 2H). ^{13}C NMR (DMSO-d$_6$): 162,4 (C_6), 153.9 (C_5), 149.6 (C_1), 139.9 (C_4), 136.8 (C_{12}), 135.3 (C_7), 130.1 (C_9), 127.6 (C_8), 125.4 (C_2), 123.2 ($C_{1\text{-}2}$), 120.2 ($C_{3\text{-}7}$). ESI-MS (MeOH) m/z negative mode: 553.0 [H_2L5+H$^+$]$^-$, 276.1 [H_2L5]$^{2-}$. Elemental analysis (% found): C, 55.3; H, 6.0; N, 10.6. Calcd for $C_{24}H_{16}N_4O_8S_2^{2-}$·2 Et_3NH^+·1.3 H_2O: C, 55.4; H, 6.5; N, 10.8. IR ($\tilde{\nu}$ / cm^{-1}): 3422m, 3344m, 3276m, 3001m, 2702m, 2502w, 1682m, 1580s, 1524s, 1425s, 1398w, 1245s, 1222m, 1165s, 1149m, 1124m, 1008m, 904w, 785w, 613m.

[H₂L6]*(Et₃NH)₂*

Yield: 1.29 g, 1.48 mmol, 74 %. ^{1}H NMR (DMSO-d$_6$): *δ* [ppm] = 10.48 (H_4, s, 2H), 9.21 (H_1, d, *J* = 7.9, 1.0 Hz, 2H), 8.87 ($(CH_3CH_2)_3NH$, s, 2H), 8.25-8.32 ($H_{2\text{-}3}$, m, 4H), 7.56 (H_5, s, 4H), 3.09 ($(CH_3CH_2)_3NH$, q, *J* = 6.9 Hz, 12H), 2.60 (H_6, s, 12H), 1.17 ($(CH_3CH_2)_3NH$, tr, *J* = 7.2 Hz, 18H). ^{13}C NMR (DMSO-d$_6$): 162.4 (C_6), 153.9 (C_5), 149.6 (C_1), 139.9 (C_4), 136.8 (C_{12}), 135.3 (C_7), 130.1 (C_9), 127.6 (C_8), 125.4 (C_2), 123.2 ($C_{1\text{-}2}$), 120.2 ($C_{3\text{-}7}$), 62.0 ($(CH_3CH_2)_3NH$), 25.5 ($(CH_3CH_2)_3NH$). HRMS-ESI(MeOH): calcd for [H_2L6]$^{2-}$, ($C_{28}H_{24}N_4O_8S_2$): 304.0523, found 304.0535. Elemental analysis (% found): C, 57.8; H, 6.3; N, 10.2. Calcd for $C_{24}H_{26}N_4O_8S_2^{2-}$·2$Et_3NH^+$: C, 58.1; H, 7.0; N, 10.2. IR ($\tilde{\nu}$ / cm^{-1}): 3441br, 3330w, 3084w, 2984m, 1681s, 1644m, 1581m, 1530s, 1436m, 1399m, 1169s, 1082s, 1011s, 681m.

***[H₂L7]**(Et₃NH)₂·2 H₂O*

Yield: 500 mg, 0.58 mmol, 29 %. ^{1}H NMR (400 MHz, DMSO-d$_6$): δ [ppm] = 11.13 (H_4, s, 2H), 9.37 (H_1, dd, J = 7.1, 1.8 Hz, 2H), 8.95 (H_7, m, 2H, H12), 8.30 ($H_{2,3}$, m, 4H), 8.05 ($H_{6,10}$, m, 4H), 7.72 (H_5, d, J = 7.7 Hz, 2H), 7.59 ($H_{8,9}$, m, 4H, H13, H14), 3.09 (($CH_3CH_2)_3NH$, q, 7.3 Hz, 12H), 1.17 (($CH_3CH_2)_3NH$, tr, J = 7.2 Hz, 18H). ^{13}C NMR (101 MHz, DMSO-d$_6$): δ [ppm] = 163.3 (C_6), 153.7(C_1), 149.5 (C_5), 142.4 (C_{10}), 139.3 (C_3), 134.5 (C_{11}), 129.8 (C_7), 129.3 (C_{16}), 128.1 (C_{12}), 125.8 (C_{13+14}), 124.8 (C_2), 124.2 (C_9), 123.2 (C_4), 122.8 (C_{15}), 121.8 (C_8), 62.0 (($CH_3CH_2)_3NH$), 25.5 (($CH_3CH_2)_3NH$). HRMS-ESI (MeOH): Calcd. for $[H_2L7]^{2-}$, ($C_{32}H_{22}N_4O_8S_2$): 326.0367, found 326.0373. Elemental analysis (% found): C, 59.25; H, 6.72; N, 9.47. Calcd for $C_{32}H_{22}N_4O_8S_2^{2-}\cdot 2Et_3NH^{+}\cdot 2H_2O$: C, 59.18; H, 6.32; N, 9.41. IR ($\tilde{\nu}$ / cm^{-1}): 3398s, 3206s, 3088s, 1685s, 1610m, 1532s, 1508s, 1438w, 1147s, 1041s, 750m, 681m.

***[H₂L8]**(Et₃NH)₂·1.5H₂O*

Yield: 774 mg, 1.81 mmol, 88%. ^{1}H NMR (400 MHz, DMSO-d$_6$): δ [ppm] = 10.88 (H_4, s, 2H), 9.29 (H_1, dd, J = 6.3, 2.7 Hz, 2H), 8.54 (H_{10}, s, 2H), 8.40 – 8.29 ($H_{2,3}$, m, 4H), 8.14 (H_7, s, 2H), 8.04 ($H_{5,6}$, m, 4H), 7.86 (H_9, d, J = 8.7 Hz, 2H), 7.72 (H_8, dd, J = 8.5, 1.5 Hz, 2H), 3.09 (($CH_3CH_2)_3NH$, m, 12H), 1.17 (($CH_3CH_2)_3NH$, tr, J = 7.2 Hz, 18H). ^{13}C NMR (101 MHz, DMSO) δ 162.7 (C_6), 153.5 (C_1), 149.6 (C_5), 144.7 (C_{12}), 139.4 (C_3), 136.3 (C_7), 133.1 (C_{15}), 129.3 (C_{10}), 128.9 (C_9), 127.0 (C_{14}), 124.9 (C_2), 124.5 (C_{13}), 123.9 (C_{11}), 123.2 (C_4), 121.9 (C_8), 117.3 (C_{16}), 62.0 (($CH_3CH_2)_3NH$), 25.5 (($CH_3CH_2)_3NH$). HRMS-ESI (MeOH): calcd for $[H_2L8]^{2-}$, ($C_{32}H_{22}N_4O_8S_2$): 326.0367, found 326.0362. Elemental analysis (% found): C, 59.6; H, 5.9; N, 9.5. Calcd. for $C_{32}H_{22}N_4O_8S_2^{2-}\cdot 2Et_3NH^{+}\cdot 1.5H_2O$: C, 59.8; H, 6.3; N, 9.5. IR ($\tilde{\nu}$ / cm^{-1}): 3310s, 3021s, 2860s,2776s, 2501m, 1682s, 1580m, 1536s, 1493m,1438m, 1393m, 1340w, 1241s, 1159s, 1090s, 1019s, 823m, 670m.

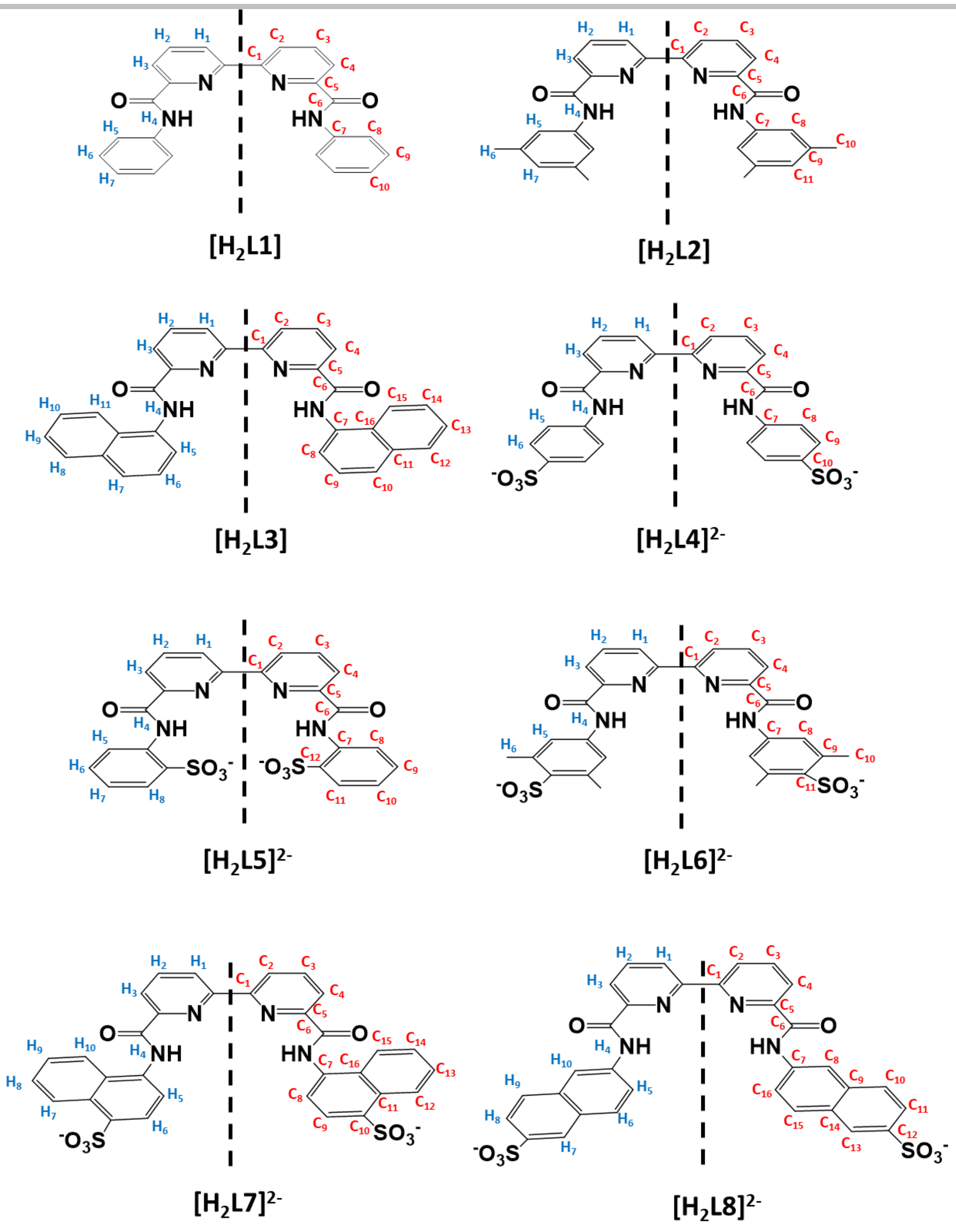

Figure S1. Schematic drawing of the ligands synthesized in this work and their ^{1}H NMR and ^{13}C NMR assignments.

Synthesis of Cu-complexes

The general procedure for the synthesis of the copper complexes was adapted from a previous published work.[6] Typically, 0.2 mmol of the corresponding ligand were suspended in 8 mL of MeOH and stirred during 15 minutes. Afterwards, 7.2 mL (4 eq.) of 0.1 M $NaOH_{aq.}$ were added to the reaction mixture and vigorously stirred during 30 minutes at room temperature. After complete dissolution of the ligand, 0.2 mmol of copper perchlorate hexahydrate dissolved in 8 mL of MeOH were added dropwise to the mixture, which were allowed to react overnight (16 h) at room temperature. Then, the reaction mixture was filtrated and MeOH was evaporated under vacuum, and the remaining solution was diffused with the corresponding solvent (further details about crystallization are explained in Table S1), yielding the corresponding pure copper complexes.

***[(L1)Cu]**·1.5 H_2O·MeOH*

Yield: 38 mg, 0.08 mmol, 42% ESI-MS (MeOH) m/z positive mode: 456.0 $[(L1)Cu+H]^-$, 494.1 $[(L1)Cu+K]^-$. Elemental analysis (% found): C, 54.6; H, 3.2; N, 10.5. Calcd. for $C_{24}H_{16}CuN_4O_2$·1.5 H_2O ·MeOH: C, 54.8; H, 3.3; N, 10.6. IR ($\tilde{\nu}$ / cm^{-1}): 3396s, 3055w, 2922w, 2837w, 1625m, 1614m, 1577s, 1487m, 1468m, 1354m, 1337m, 1283m, 1142m, 1028m, 747m, 689m, 485w.

***[(L2)Cu]**·2 H_2O·MeOH*

Yield: 41 mg, 0.08 mmol, 42% ESI-MS (MeOH) m/z positive mode: 512.1 $[(L2)Cu+Na]^+$, 1023.0 $[2(L2)Cu+H]^+$. HRMS-ESI($CHCl_3$/MeOH): calcd for $[(L3)Cu+H]^+$, (C28H25N4O2Cu) : 512.1259, found 512.1268. Elemental analysis (% found): C, 58.5; H, 6.1; N, 9.5. Calcd for $C_{28}H_{24}CuN_4NO_2$·2 H_2O ·MeOH: C, 58.2; H, 5.7; N, 9.4. IR ($\tilde{\nu}$ / cm^{-1}): 3355m, 3031w, 2999w, 2911m, 2860w, 1623m, 1570s, 169m, 1353s, 1262m, 1076m, 1027m, 826m, 767m, 688m, 579w.

***[(L3)Cu]**·1.5MeOH*

Yield: 45 mg, 0.08 mmol, 40% ESI-MS (MeOH) m/z positive mode: 556.1 $[(L3)Cu+H]^+$, 578.1 $[(L3)Cu+Na]^+$ 1111.1 $[2·(L3)Cu+H]^+$. HRMS-ESI(+ve): calcd for $[(L3)Cu+H]^+$, ($C_{32}H_{21}CuN_4O_2$): 556.0955, found: 556.0948. Elemental analysis (% found): C, 66.3; H, 3.9; N, 9.6. Calcd for $C_{32}H_{20}CuN_4Na_2O_8S_2$·1.5 MeOH: C, 66.6; H, 4.3; N, 9.3. IR ($\tilde{\nu}$ / cm^{-1}): 3655w, 3381m, 3192m, 3051m, 1624m, 1578s, 1565s, 1504m, 1472w, 1392m, 1338m, 1285m, 1037m, 790m, 763m, 705w.

***[(L4)Cu]**Na_2 ·3H_2O*

Yield: 19 mg, 0.03 mmol, 32% ESI-MS (MeOH) m/z negative mode: 636.0 $[(L4)Cu+Na^+]^-$, 614.0 $[(L4)Cu+H]^-$. Elemental analysis (% found): C, 40.2; H, 2.5; N, 7.7. Calcd for $C_{24.}H_{14}CuN_4Na_2O_8S_2$·3 H_2O: C, 40.4; H, 2.8; N, 7.8. IR ($\tilde{\nu}$ / cm^{-1}): 3585s, 3513s, 3460s, 3382m, 3227m, 1621m, 1597s, 1574m, 1497m, 1474m, 1358m, 1199m, 1120s, 1032s, 1007m, 820w, 693m, 570m.

***[(L5)Cu]**Na_2·4 H_2O·2 MeOH*

Yield: 15 mg, 0.02 mmol, 25%. ESI-MS (MeOH) m/z negative mode: 636.0 $[(L5)Cu+Na^+]^-$, 614.0 $[(L5)Cu+H]^-$. Elemental analysis (% found): C, 39.2; H, 3.9; N, 6.5. Calcd for $C_{24}H_{14}CuN_4Na_2O_8S_2$·4 H_2O ·2 MeOH: C, 39.2; H, 3.8; N, 7.0. IR ($\tilde{\nu}$ / cm^{-1}): 3585s, 3513s, 3459m, 3417s, 3331m, 3226m, 3097w, 1620w, 1593m, 1574s, 1472m, 1388m, 1293w, 1177m, 1084s, 1013m, 761w, 161m, 569m.

***[(L6)Cu]**Na_2·2.4H_2O*

Yield: 42 mg, 0.06 mmol, 72% ESI-MS (MeOH) m/z negative mode: 609.0 $[(L6)Cu+H^+]^-$. Elemental analysis (% found): C, 44.4; H, 3.9; N, 7.4. Calcd for $C_{28}H_{22}CuN_4Na_2O_8S_2$·2.4 H_2O: C, 44.3; H, 3.6; N, 7.4. IR ($\tilde{\nu}$ / cm^{-1}): 3404s, 3076w, 2974w, 2937w, 1612w, 1591m, 1557s, 1472m, 1368m, 1305w, 1170s, 1084s, 1016m, 765m, 690m, 648m.

***[(L7)Cu]**Na_2·7H_2O*

Yield: 59 mg, 0.08 mmol, 49% ESI-MS ($CHCl_3$/MeOH) m/z negative mode: 713.8 $[(L7)Cu+H^+]^-$, 735.8 $[(L7)Cu+Na^+]^-$. HRMS-ESI ($CHCl_3$/MeOH): calcd for $[(L7)Cu]^{2-}$, ($C_{32}H_{18}CuN_4O_8S_2$) : 356.4937, found 356.4933. Elemental analysis (% found): C, 43.4; H, 3.2; N, 6.2. Calcd for $C_{32}H_{18}CuN_4Na_2O_8S_2$·7 H_2O: C, 43.4; H, 3.6; N, 6.3. IR ($\tilde{\nu}$ / cm^{-1}): 3626m, 3358s, 3078w, 1614w, 1575m, 1552s, 1506m, 1420w, 1390s, 176m, 1285w, 1214w, 1182s, 1156m, 1045s, 1156m, 1045s, 1023m, 759m, 688m, 612w.

***[(L8)Cu]**Na$_2$·6H$_2$O*

Yield: 12 mg, 16 mmol, 20% ESI-MS (MeOH) m/z negative mode: 735.8 [(L8)Cu+Na$^+$]$^-$, 713.8 [(L8)Cu+H$^+$]$^-$. HRMS-ESI(-ve, MeOH): calcd for [(L8)Cu+Na]$^-$, ($C_{32}H_{18}CuN_4O_8S_2Na$): 735.9765, found 735.9763. Elemental analysis (% found): C, 44.2; H, 3.3; N, 6.5. Calcd for $C_{32}H_{18}CuN_4Na_2O_8S_2$ 6 H_2O: C, 44.3; H, 3.5; N, 6.4. IR ($\tilde{\nu}$ / cm^{-1}): 3427s, 3081m, 1673w, 1626m, 1577s, 1530m, 1494m, 1470m, 1377m, 1181s, 1096s, 1035s, 819m, 674m, 623m.

Synthesis of complex [(L4)Zn](Na)$_2$·4H$_2$O.

The procedure for the synthesis of the zinc complex was similar to the synthesis of the copper complexes. To a suspension of [H$_2$L4]$^{2-}$ (200 mg (0.36 mmol) in 16 mL of MeOH), 14.4 mL (4 eq.) of 0.1 M NaOH$_{aq.}$ were added to the reaction mixture and vigorously stirred during 30 minutes at room temperature. After complete dissolution of the ligand, 131 mg (0.36 mmol) of zinc trifluoromethanesulfonate dissolved in 4 mL of MeOH was added dropwise to the mixture, which was allowed to react overnight (16 h) at room temperature. Afterwards, a white precipitate appeared, which was filtrated and washed with H_2O (2 x 2 mL) and dried over vacuum yielding **[(L4)Zn]**Na$_2$·4 H_2O as a white solid.

***[(L4)Zn]**Na$_2$·4 H$_2$O*

Yield: 193 mg, 0.29 mmol, 81%. ^{1}H-NMR (D_2O-d$_2$): δ [ppm] = 8.04 (H$_{1-2}$, m, 4H), 7.67 (H$_3$, dd, J= 7.5, 1.3 Hz, 2H), 7.20 (H$_4$, d, = 8.5 Hz, 4H), 6.36 (H$_5$, d, J= 8.5 Hz, 4H). Elemental analysis (% found): C, 39.8; H, 3.4; N, 7.6. Calcd for $C_{24}H_{14}CuN_4Na_2O_8S_2Zn$·4 H_2O: C, 39.3; H, 3.0; N, 7.6.

Spectroscopic Characterization

NMR Spectroscopy

A

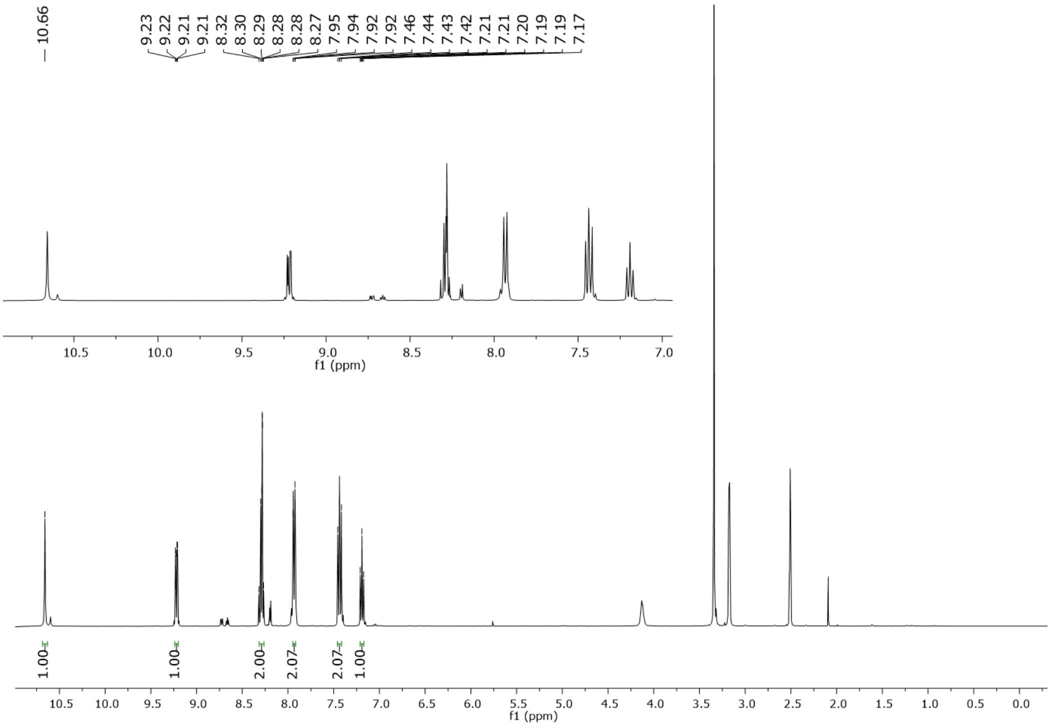

B

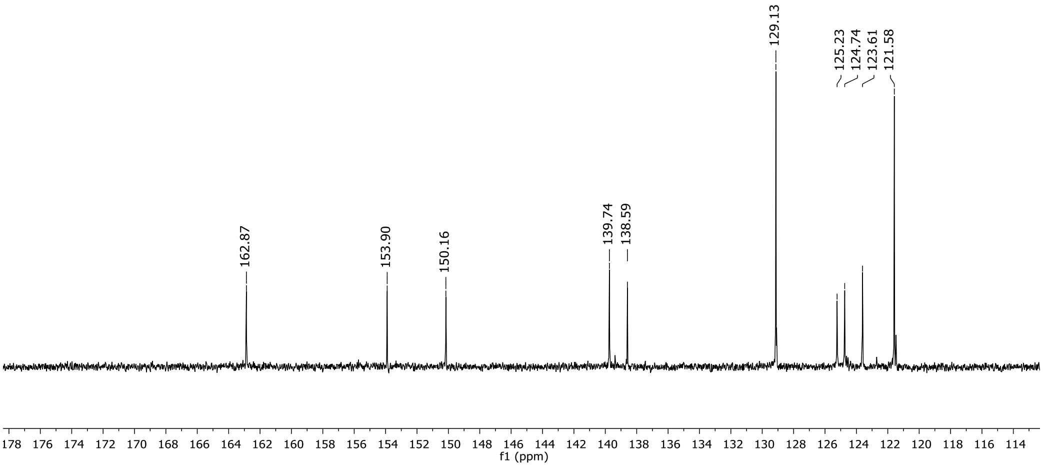

C

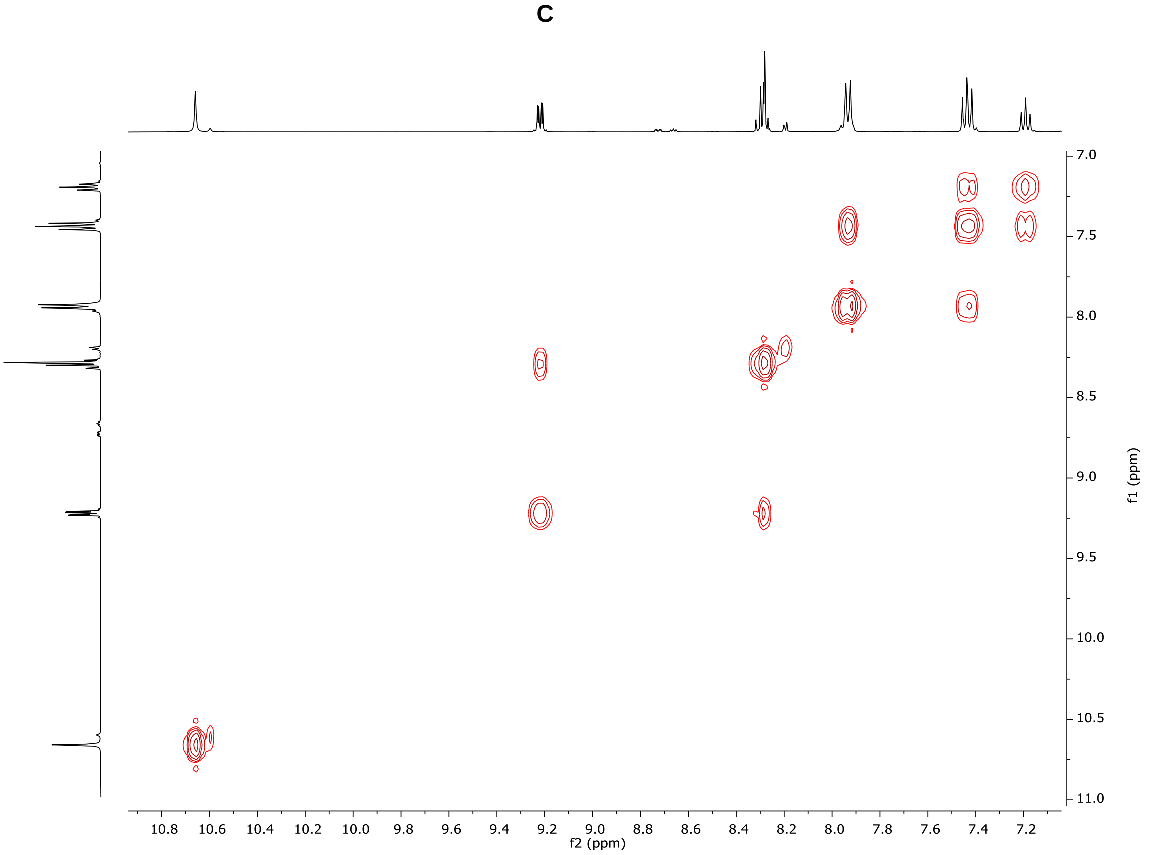
C
7.0
7.5
8.0
8.5
9.0
9.5
10.0
10.5
11.0
f1 (ppm)
10.8 10.6 10.4 10.2 10.0 9.8 9.6 9.4 9.2 9.0 8.8 8.6 8.4 8.2 8.0 7.8 7.6 7.4 7.2
f2 (ppm)

D

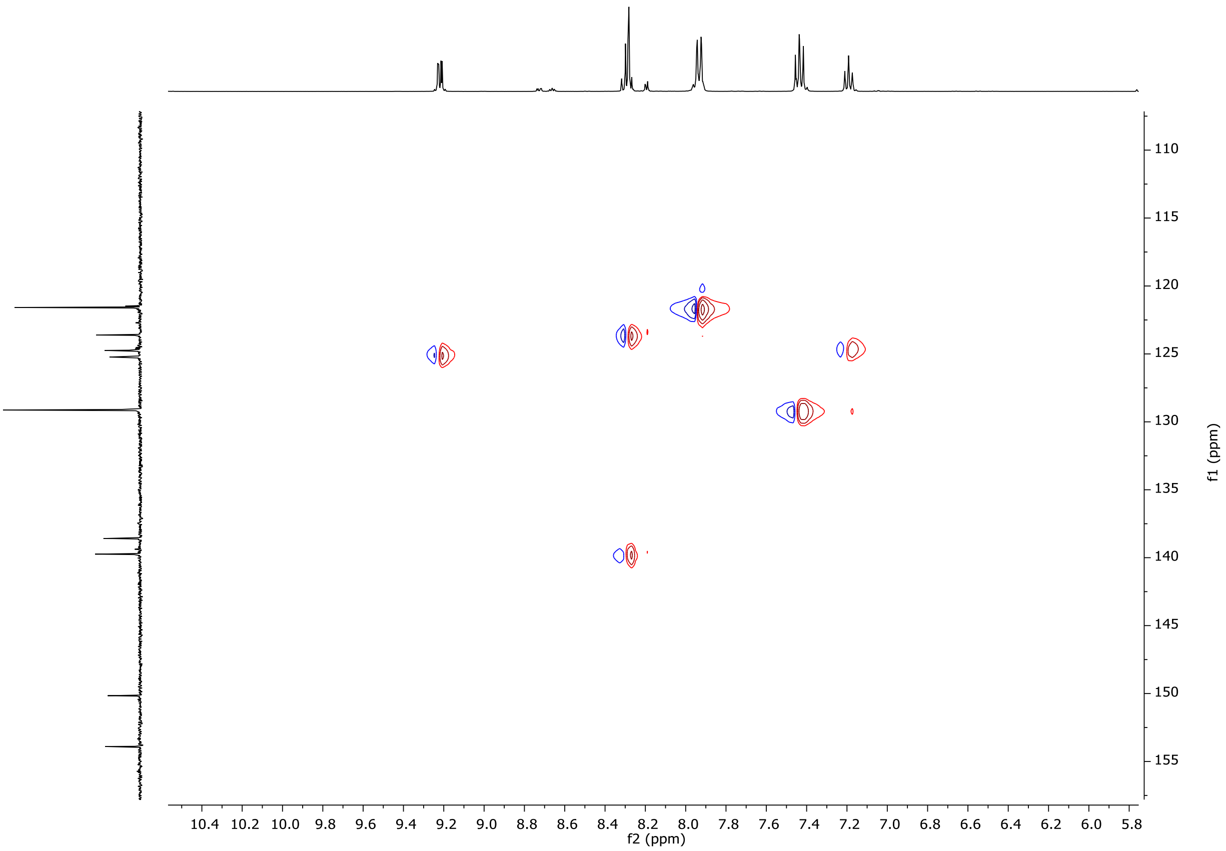
110
115
120
125
130
135
140
145
150
155
f1 (ppm)
10.4 10.2 10.0 9.8 9.6 9.4 9.2 9.0 8.8 8.6 8.4 8.2 8.0 7.8 7.6 7.4 7.2 7.0 6.8 6.6 6.4 6.2 6.0 5.8
f2 (ppm)

E

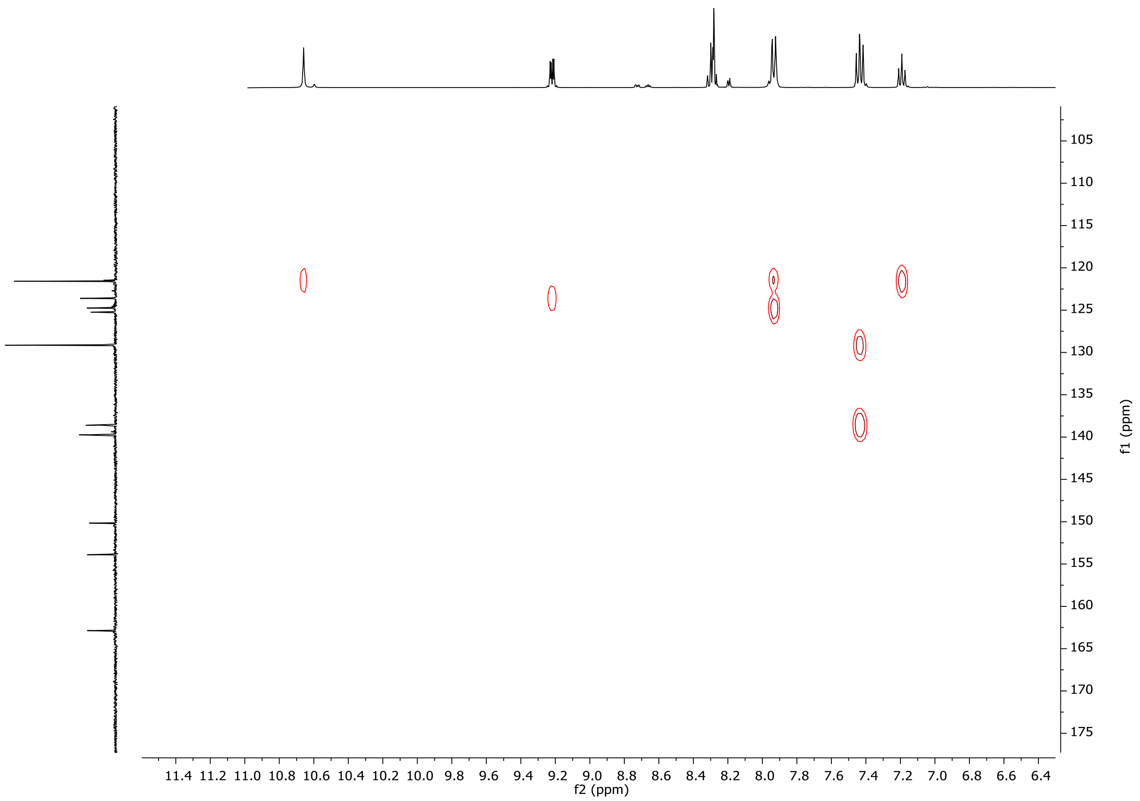

Figure S2. NMR spectra (DMSO-d_6, 500 MHz, 25 °C) for **H_2L1**. (A) ^{1}H-NMR, (B) ^{13}C NMR, (C) COSY, (D) HSQC and (E) HMBC.

A

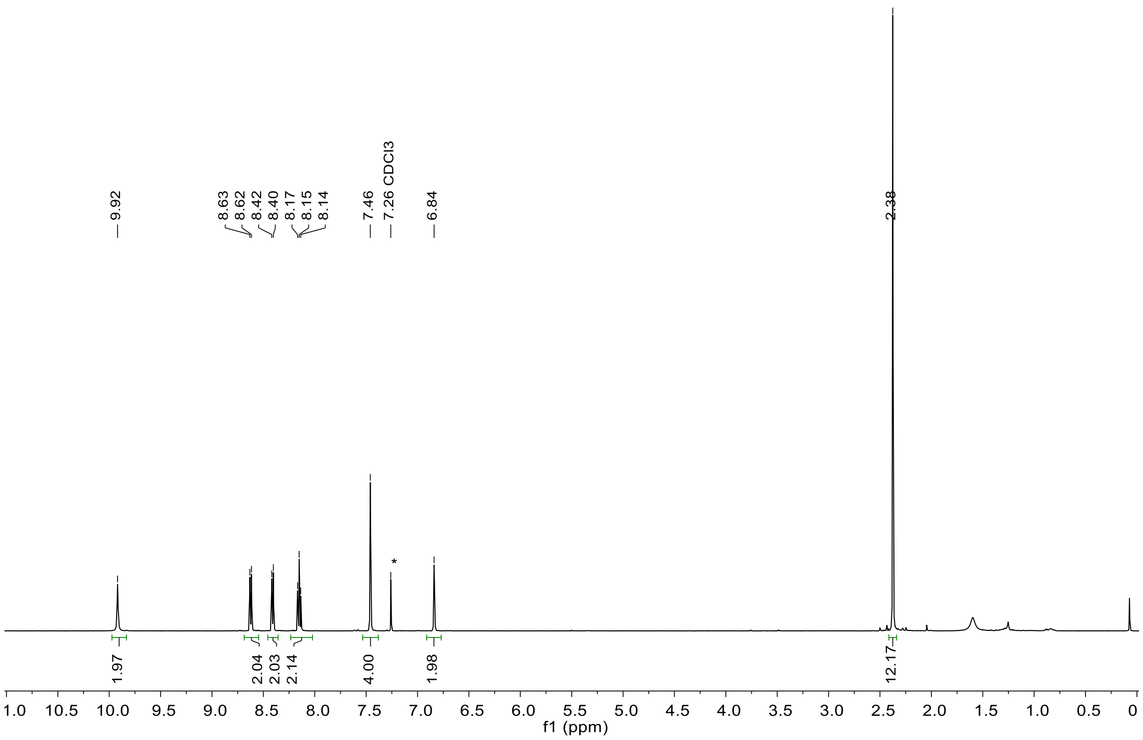
9.92
8.63
8.62
8.42
8.40
8.17
8.15
8.14
7.46
7.26 CDCl3
6.84
2.38
*
1.97
2.04
2.03
2.14
4.00
1.98
12.17
1.0 10.5 10.0 9.5 9.0 8.5 8.0 7.5 7.0 6.5 6.0 5.5 5.0 4.5 4.0 3.5 3.0 2.5 2.0 1.5 1.0 0.5 0
f1 (ppm)

B

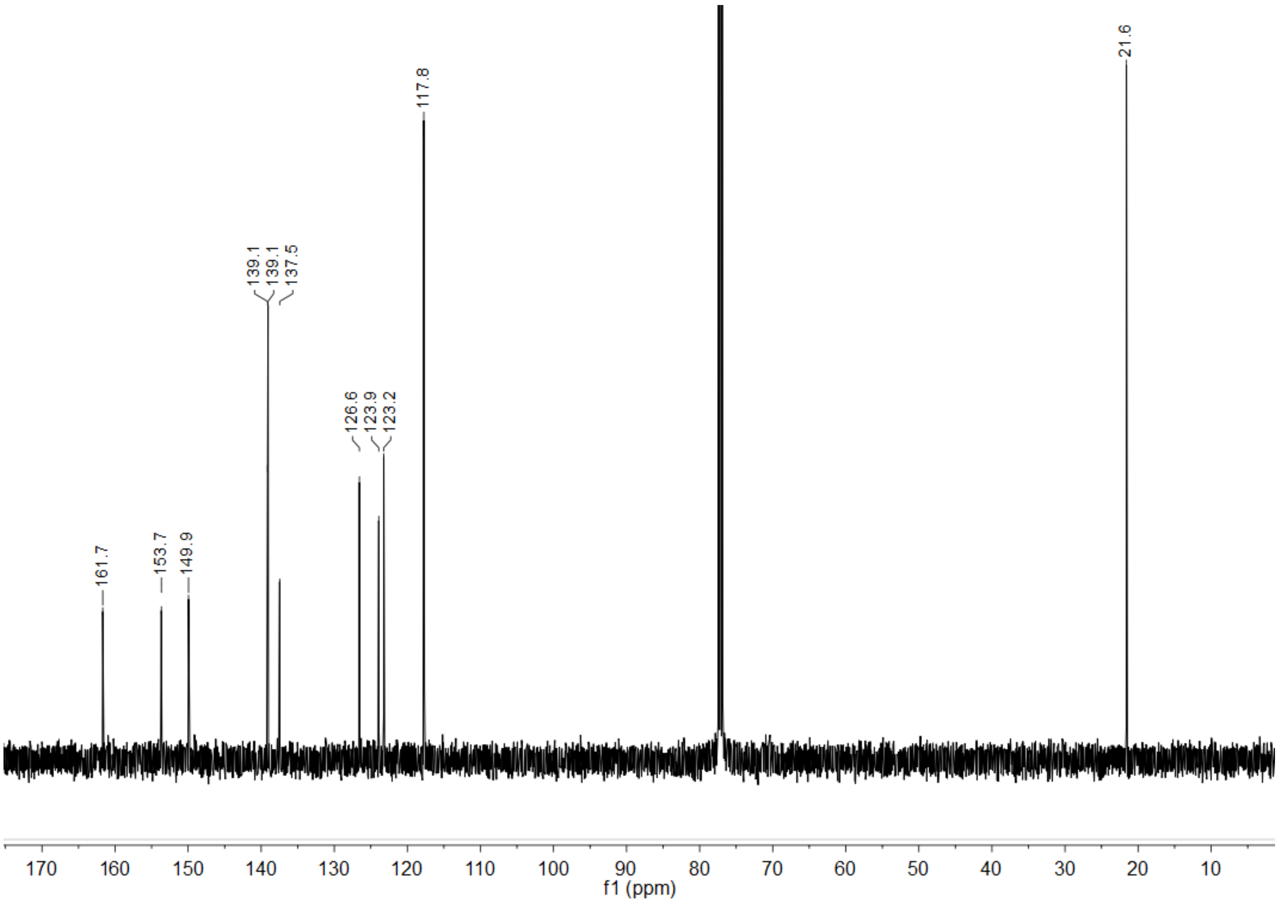
161.7
153.7
149.9
139.1
139.1
137.5
126.6
123.9
123.2
117.8
21.6
170 160 150 140 130 120 110 100 90 80 70 60 50 40 30 20 10
f1 (ppm)

C

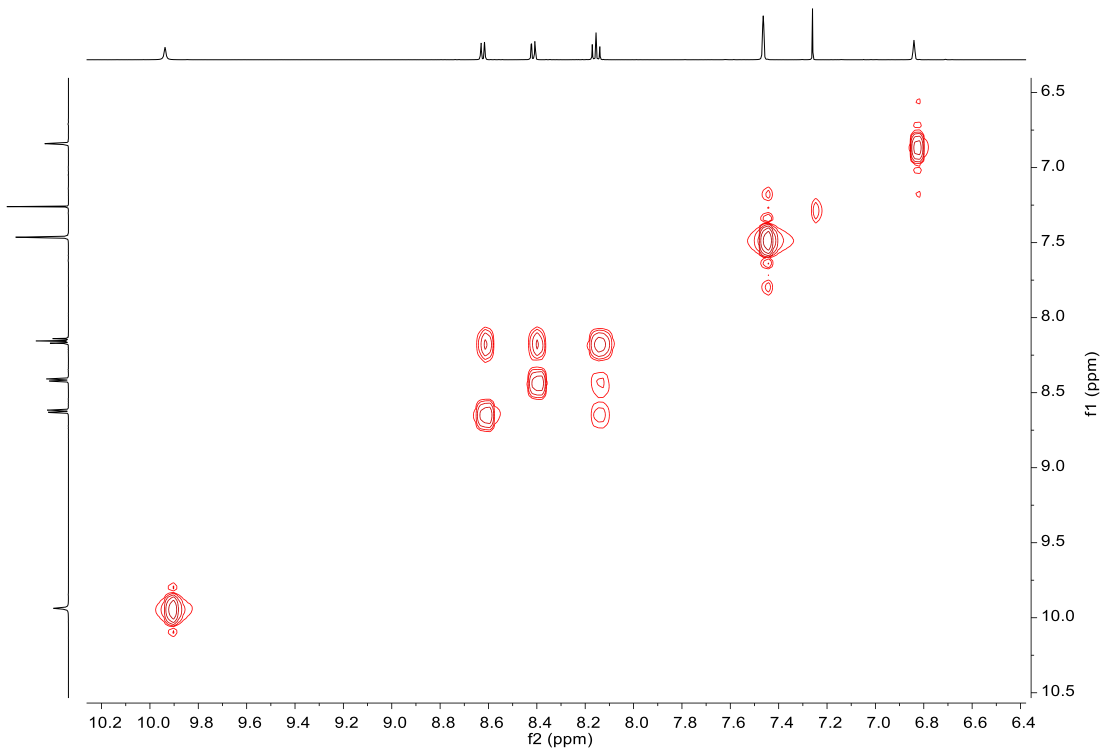
10.2 10.0 9.8 9.6 9.4 9.2 9.0 8.8 8.6 8.4 8.2 8.0 7.8 7.6 7.4 7.2 7.0 6.8 6.6 6.4
f2 (ppm)
6.5 7.0 7.5 8.0 8.5 9.0 9.5 10.0 10.5
f1 (ppm)

D

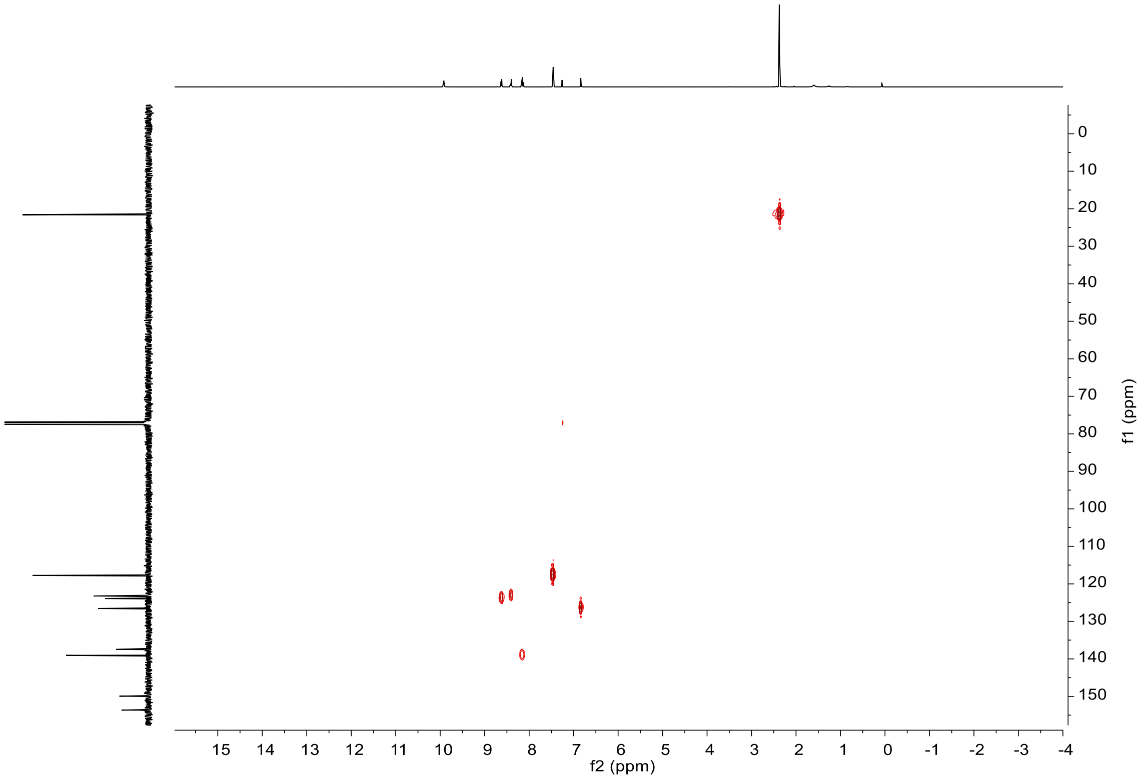
15 14 13 12 11 10 9 8 7 6 5 4 3 2 1 0 -1 -2 -3 -4
f2 (ppm)
0 10 20 30 40 50 60 70 80 90 100 110 120 130 140 150
f1 (ppm)

E

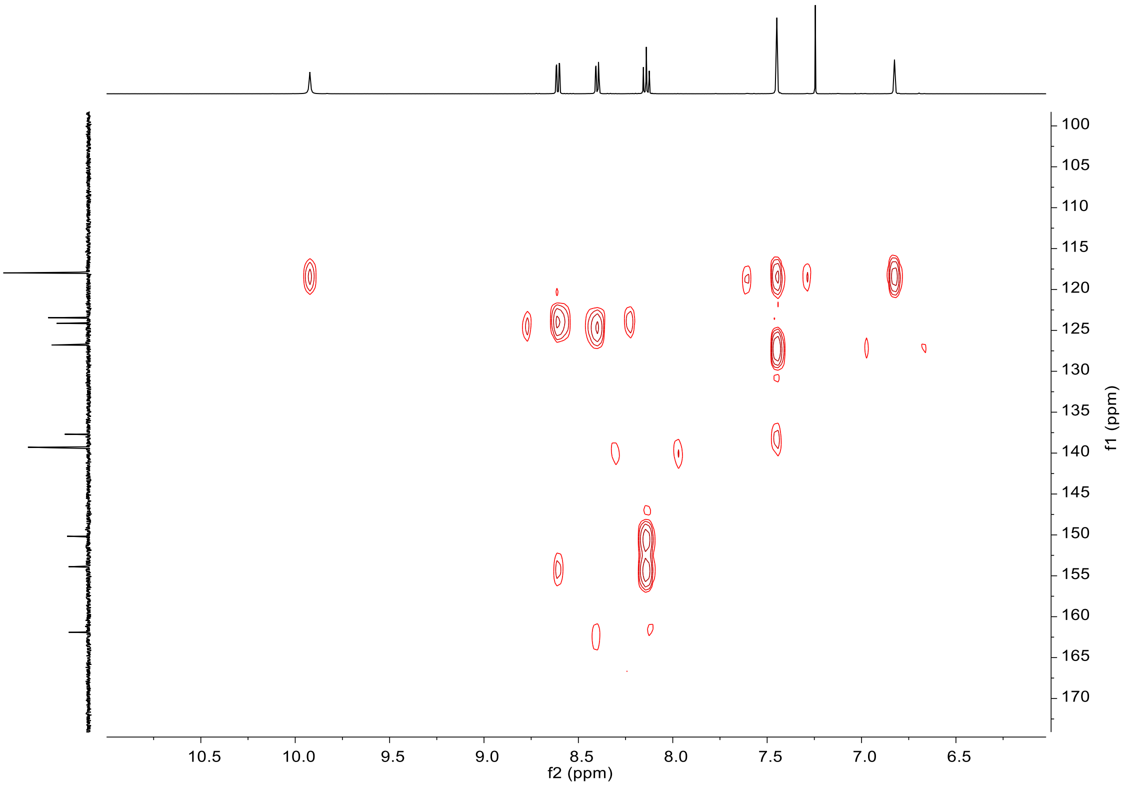

Figure S3. NMR spectra (DMSO-d_6, 500 MHz, 25 °C) for **H_2L2**. (A) ^{1}H-NMR, (B) ^{13}C NMR, (C) COSY, (D) HSQC and (E) HMBC.

A

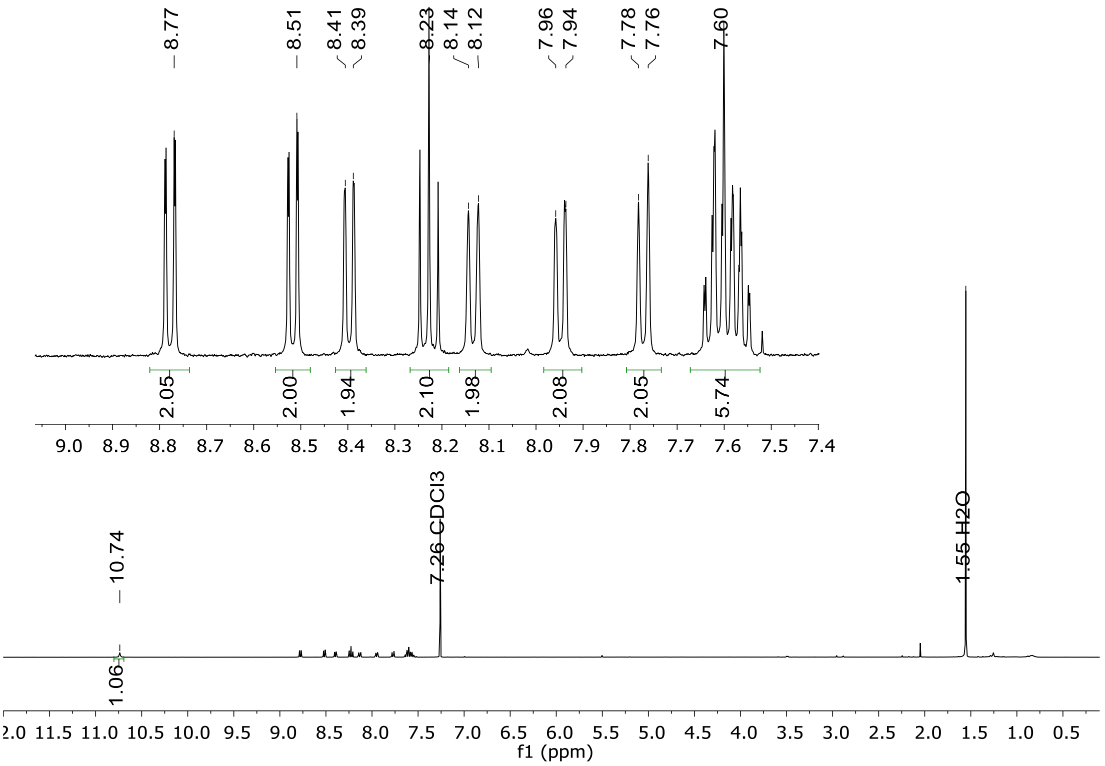

B

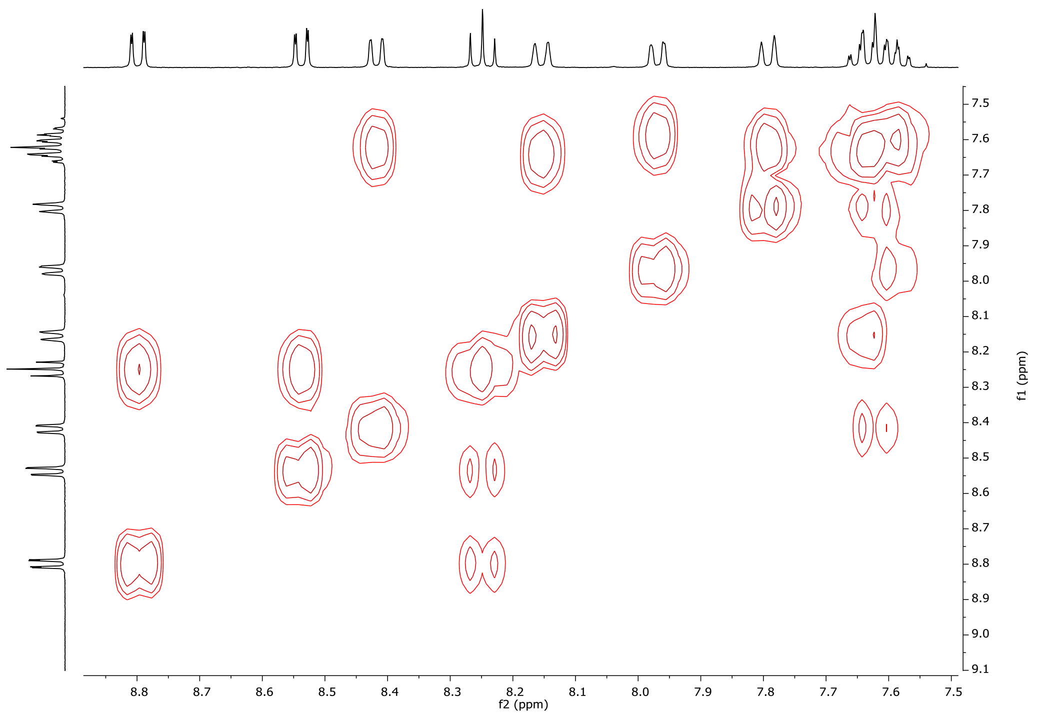

Figure S4. NMR spectra (DMSO-d_6, 500 MHz, 25 °C) for **H_2L3**. (A) ^{1}H-NMR, (B) COSY. ***Note***: Due to low solubility of the compound, ^{13}C-NMR was not measurable.

A

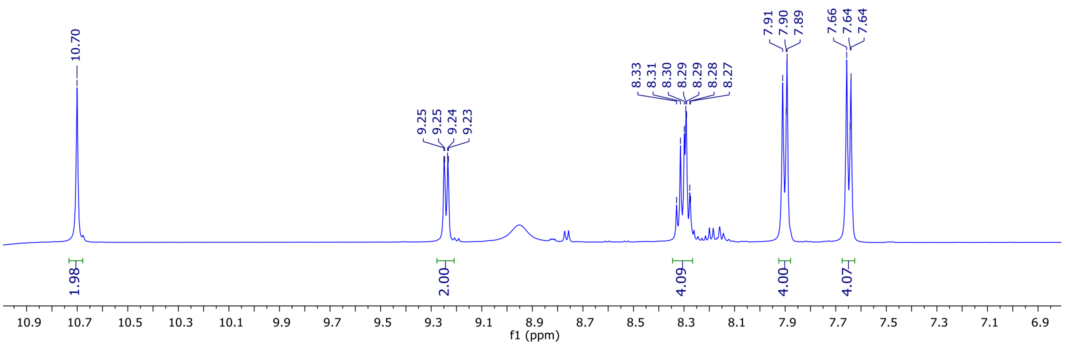
10.70
9.25
9.25
9.24
9.23
8.33
8.31
8.30
8.29
8.29
8.28
8.27
7.91
7.90
7.89
7.66
7.64
7.64
1.98
2.00
4.09
4.00
4.07
10.9 10.7 10.5 10.3 10.1 9.9 9.7 9.5 9.3 9.1 8.9 8.7 8.5 8.3 8.1 7.9 7.7 7.5 7.3 7.1 6.9
f1 (ppm)

B

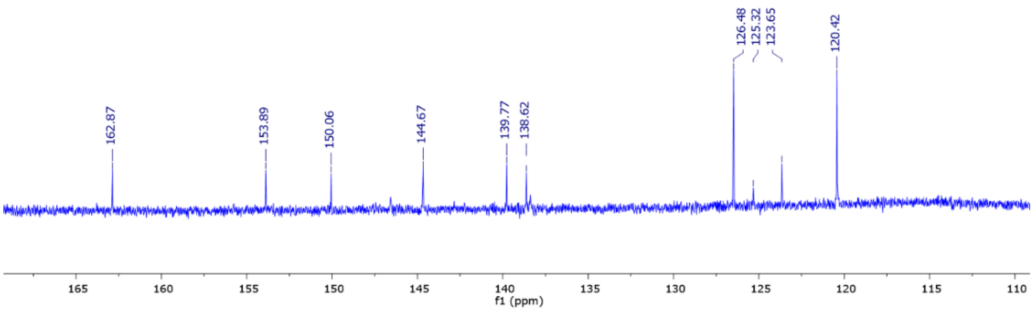
162.87
153.89
150.06
144.67
139.77
138.62
126.48
125.32
123.65
120.42
165 160 155 150 145 140 135 130 125 120 115 110
f1 (ppm)

C

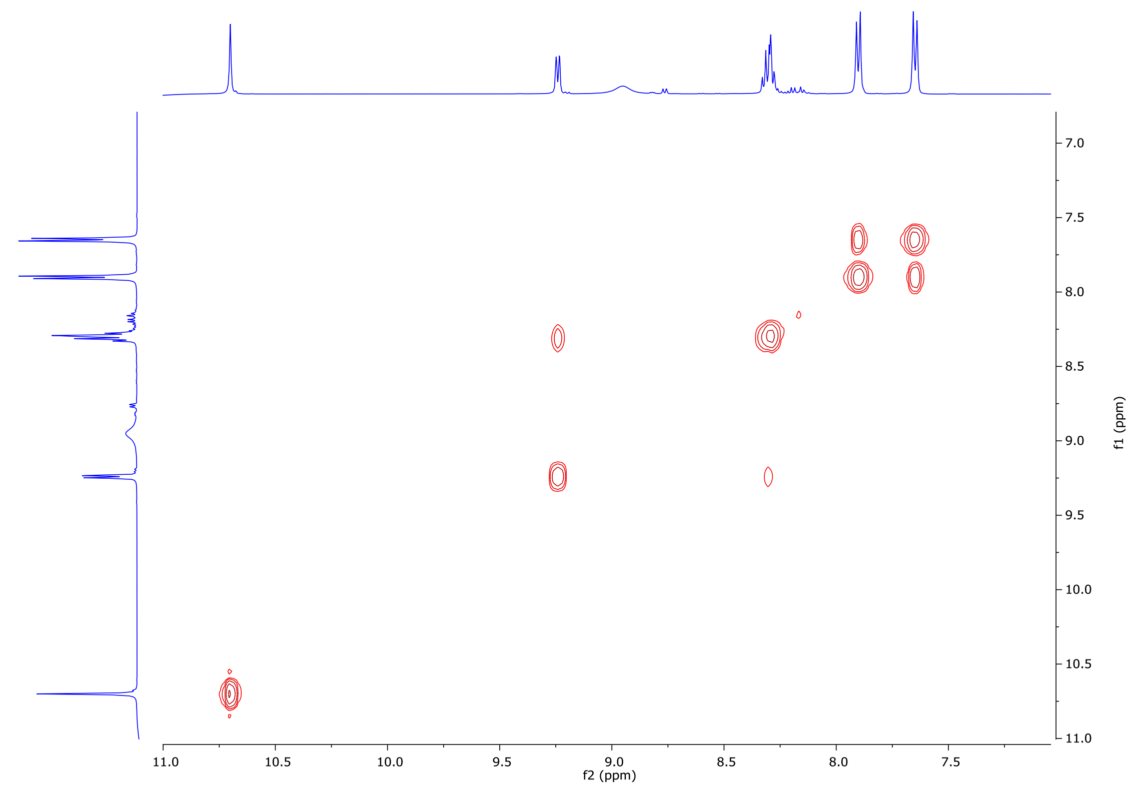
7.0
7.5
8.0
8.5
9.0
9.5
10.0
10.5
11.0
f1 (ppm)
11.0 10.5 10.0 9.5 9.0 8.5 8.0 7.5
f2 (ppm)

D

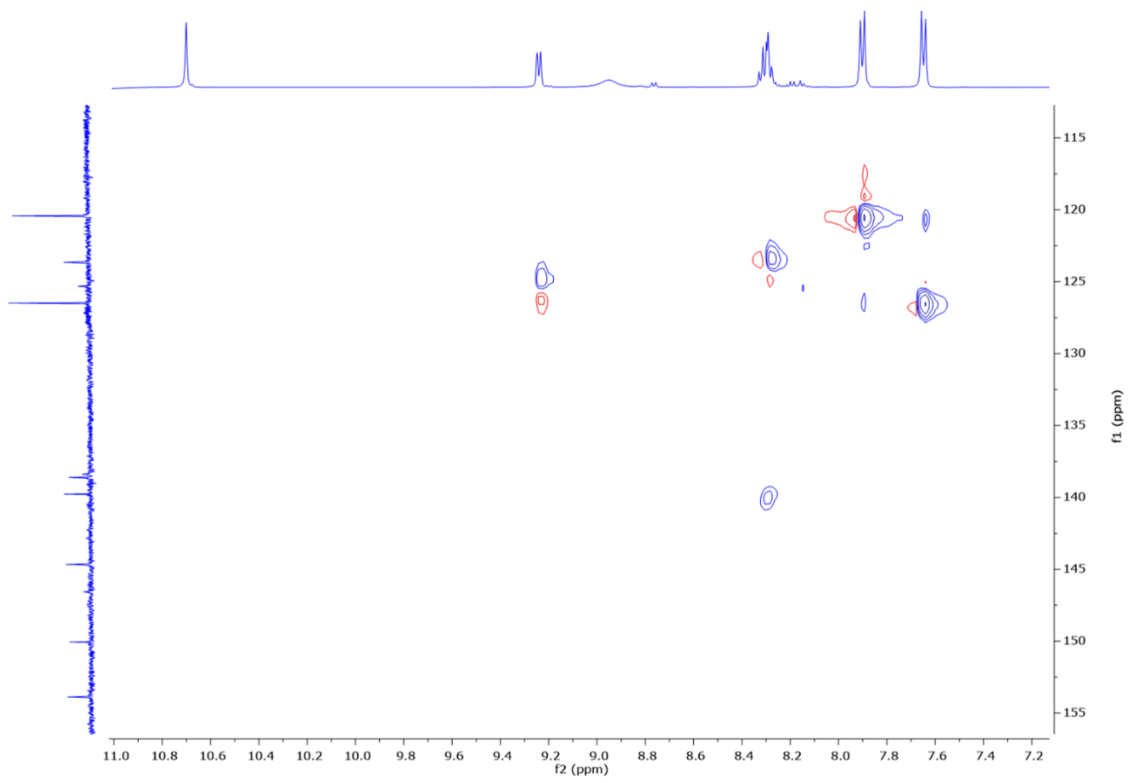
115
120
125
130
135
140
145
150
155
f1 (ppm)
11.0 10.8 10.6 10.4 10.2 10.0 9.8 9.6 9.4 9.2 9.0 8.8 8.6 8.4 8.2 8.0 7.8 7.6 7.4 7.2
f2 (ppm)

E

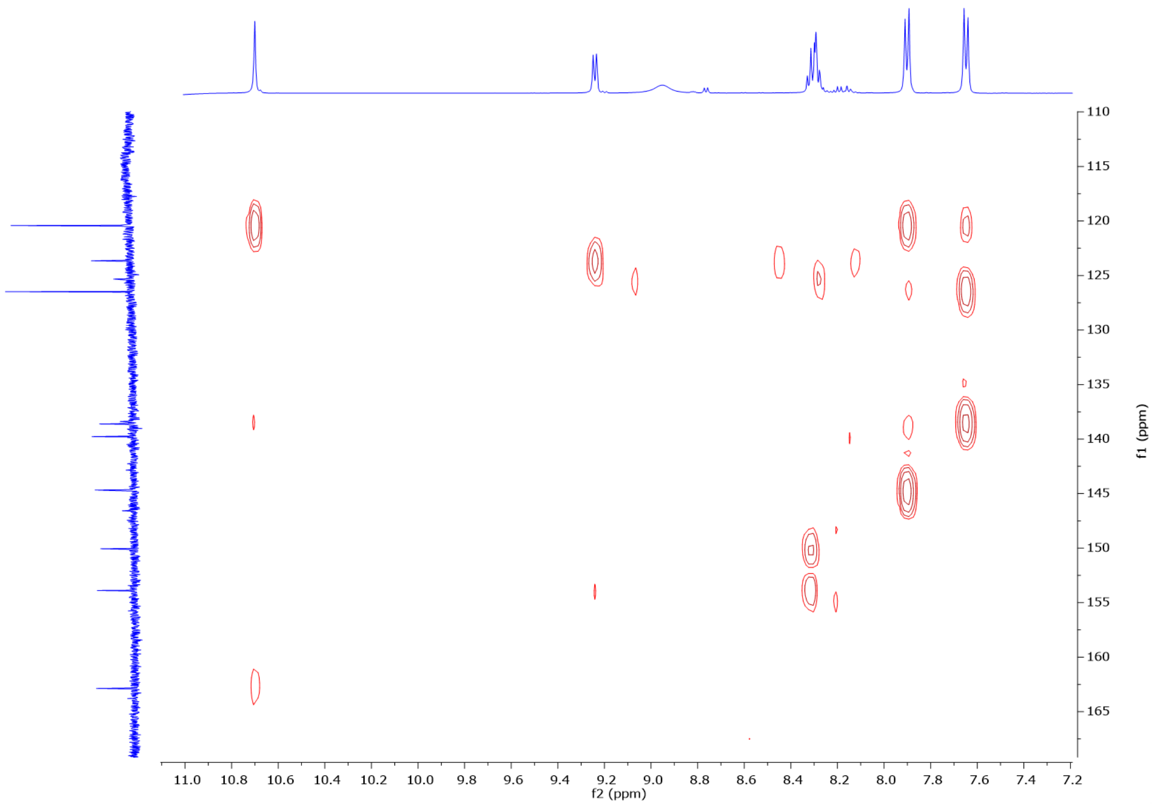

Figure S5. NMR spectra (DMSO-d_6, 500 MHz, 25 °C) for **H_2L4^{2-}**. (A) ^{1}H-NMR, (B) ^{13}C NMR, (C) COSY, (D) HSQC and (E) HMBC.

A

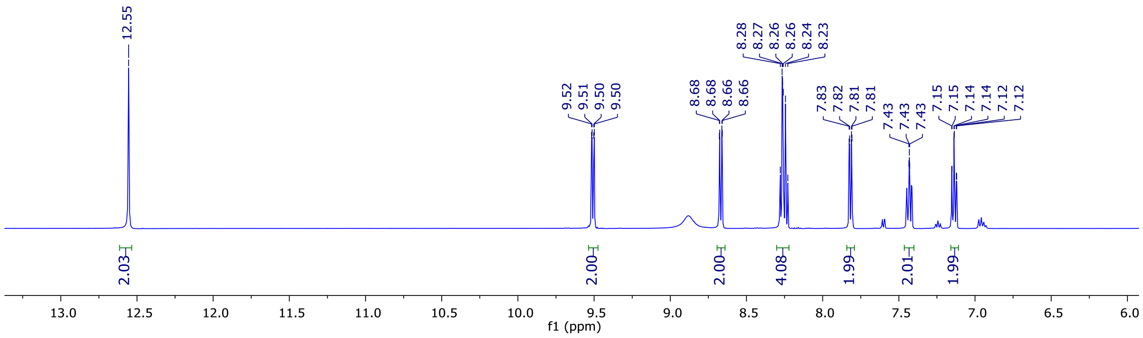
12.55
9.52
9.51
9.50
9.50
8.68
8.68
8.66
8.66
8.28
8.27
8.26
8.26
8.24
8.23
7.83
7.82
7.81
7.81
7.43
7.43
7.43
7.15
7.15
7.14
7.14
7.12
7.12
2.03
2.00
2.00
4.08
1.99
2.01
1.99
13.0 12.5 12.0 11.5 11.0 10.5 10.0 9.5 9.0 8.5 8.0 7.5 7.0 6.5 6.0
f1 (ppm)

B

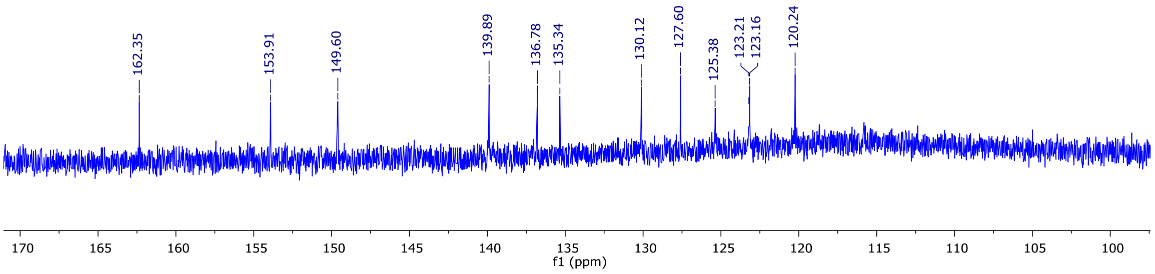
162.35
153.91
149.60
139.89
136.78
135.34
130.12
127.60
125.38
123.21
123.16
120.24
170 165 160 155 150 145 140 135 130 125 120 115 110 105 100
f1 (ppm)

C

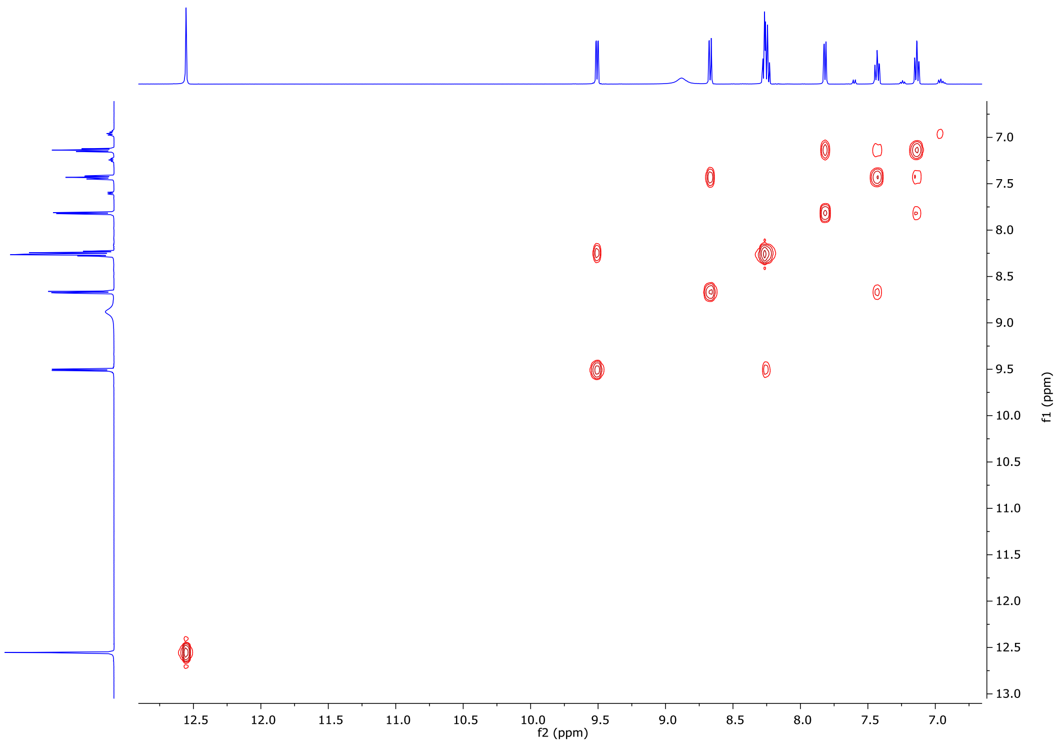
12.5
12.0
11.5
11.0
10.5
10.0
9.5
9.0
8.5
8.0
7.5
7.0
f2 (ppm)
7.0
7.5
8.0
8.5
9.0
9.5
10.0
10.5
11.0
11.5
12.0
12.5
13.0
f1 (ppm)

D

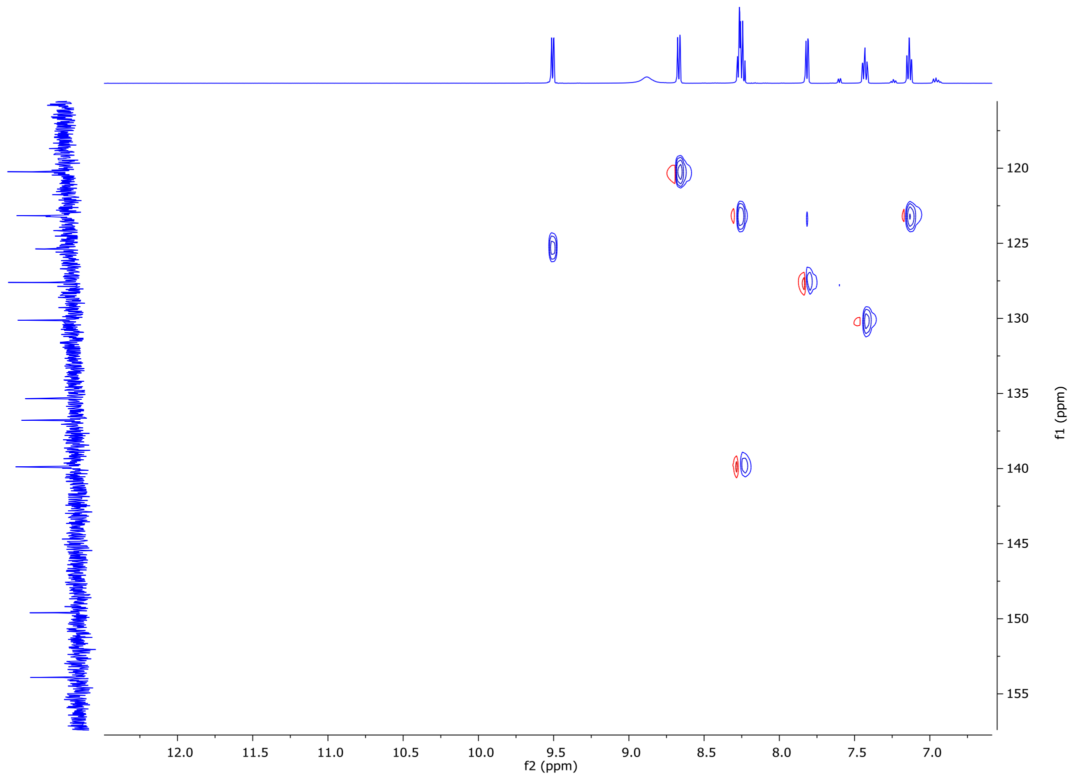
12.0
11.5
11.0
10.5
10.0
9.5
9.0
8.5
8.0
7.5
7.0
f2 (ppm)
120
125
130
135
140
145
150
155
f1 (ppm)

E

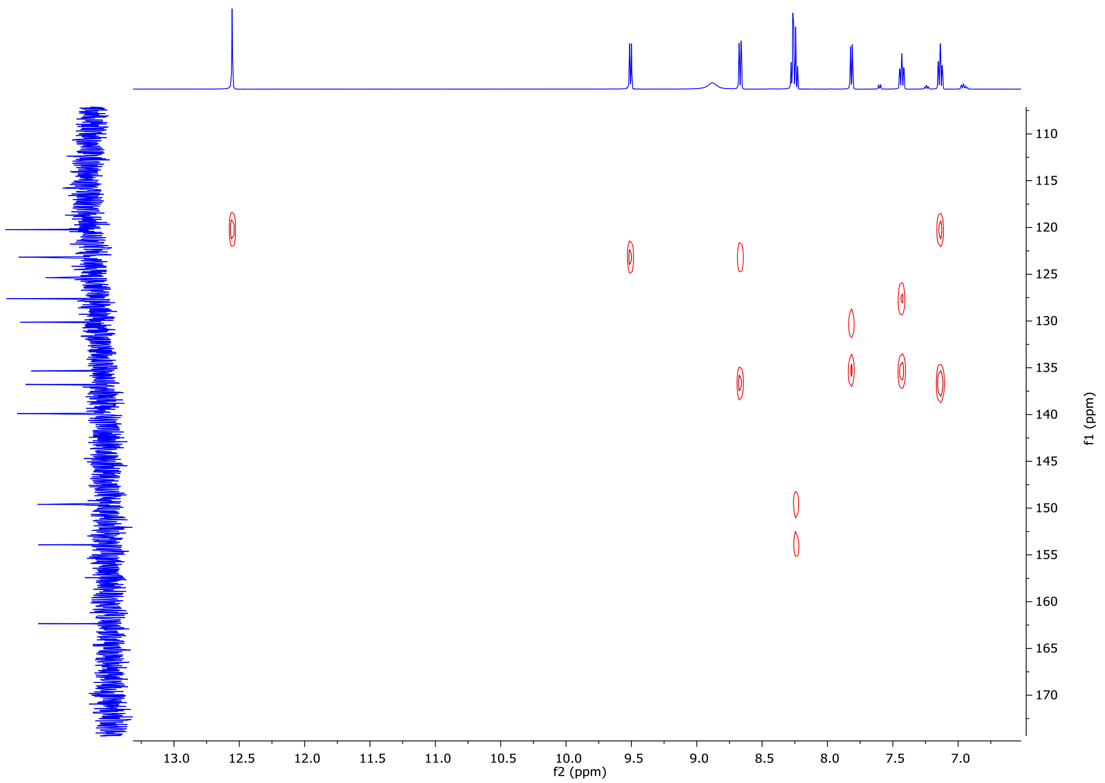

Figure S6. NMR spectra (DMSO-d_6, 500 MHz, 25 °C) for **H_2L5^{2-}**. (A) ^{1}H-NMR, (B) ^{13}C NMR, (C) COSY, (D) HSQC and (E) HMBC.

A

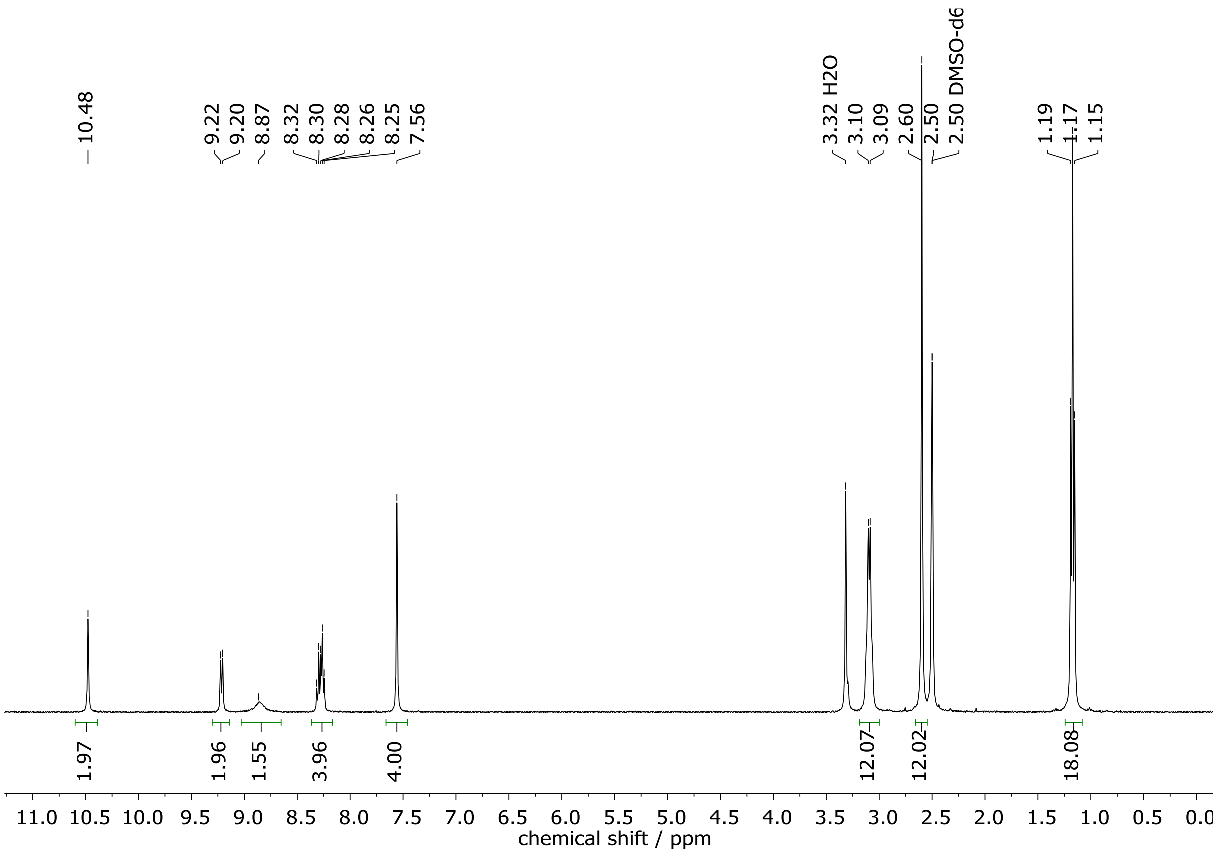
10.48
9.22
9.20
8.87
8.32
8.30
8.28
8.26
8.25
7.56
3.32 H2O
3.10
3.09
2.60
2.50
2.50 DMSO-d6
1.19
1.17
1.15
1.97
1.96
1.55
3.96
4.00
12.07
12.02
18.08
11.0 10.5 10.0 9.5 9.0 8.5 8.0 7.5 7.0 6.5 6.0 5.5 5.0 4.5 4.0 3.5 3.0 2.5 2.0 1.5 1.0 0.5 0.0
chemical shift / ppm

B

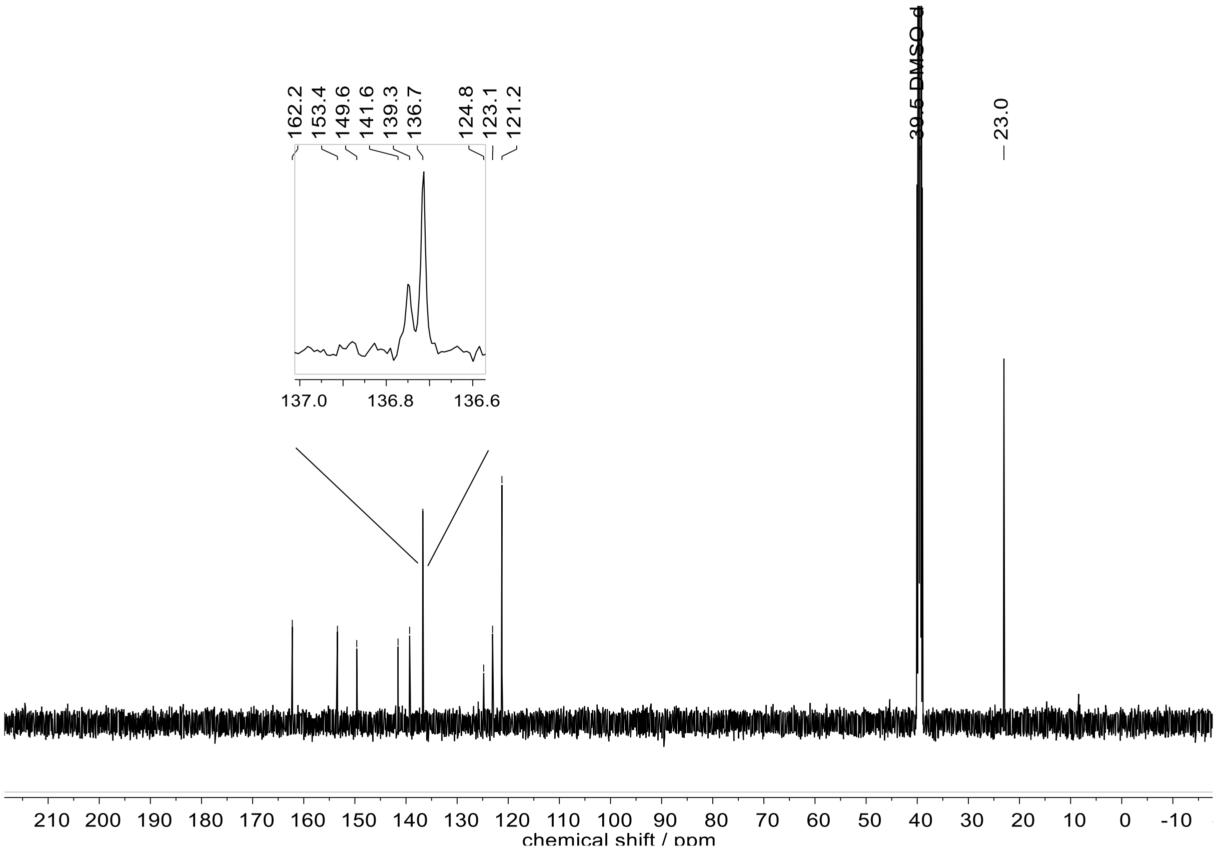
162.2
153.4
149.6
141.6
139.3
136.7
124.8
123.1
121.2
39.5 DMSO-d
23.0
137.0 136.8 136.6
210 200 190 180 170 160 150 140 130 120 110 100 90 80 70 60 50 40 30 20 10 0 -10
chemical shift / ppm

C

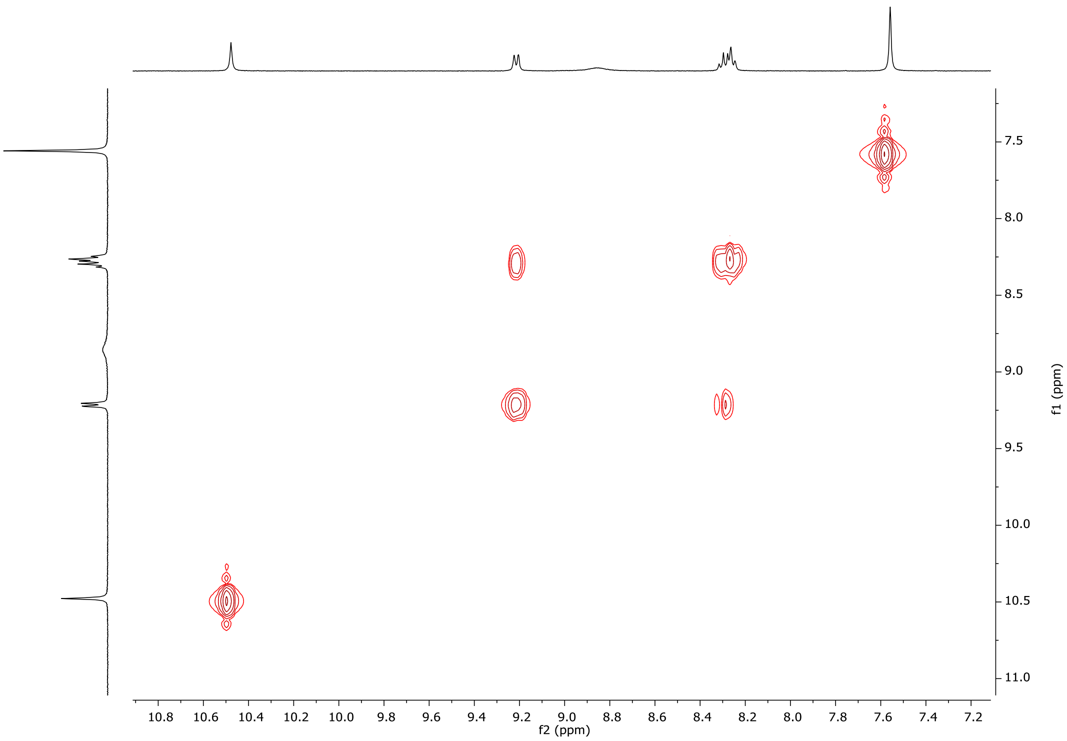

D

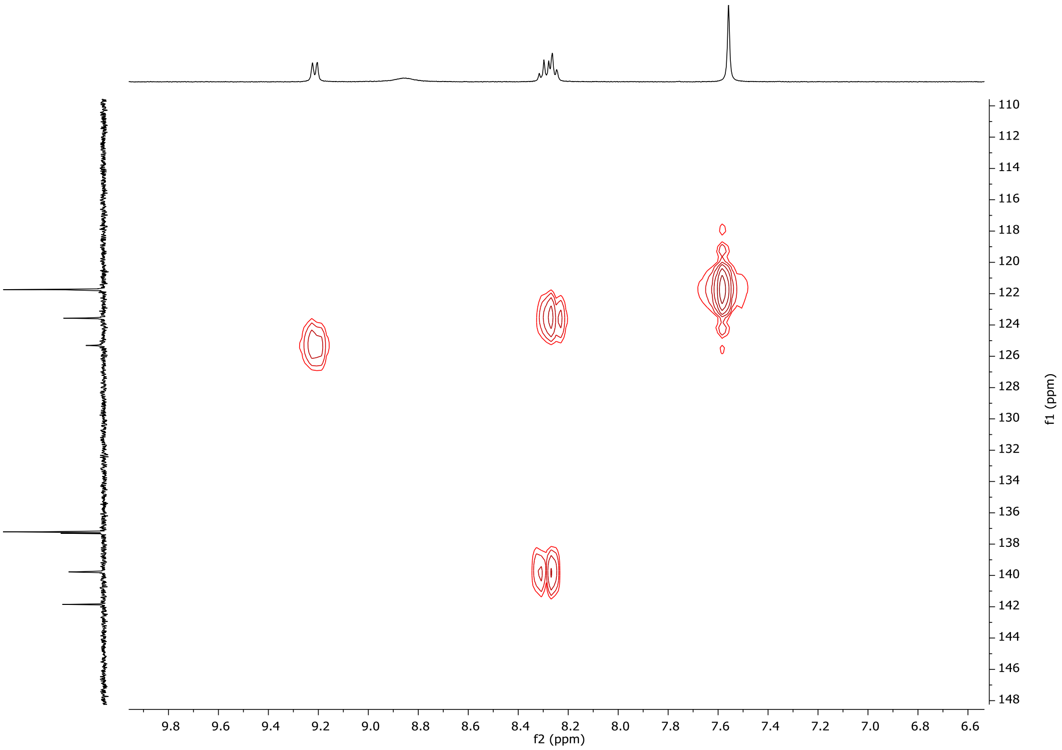

E

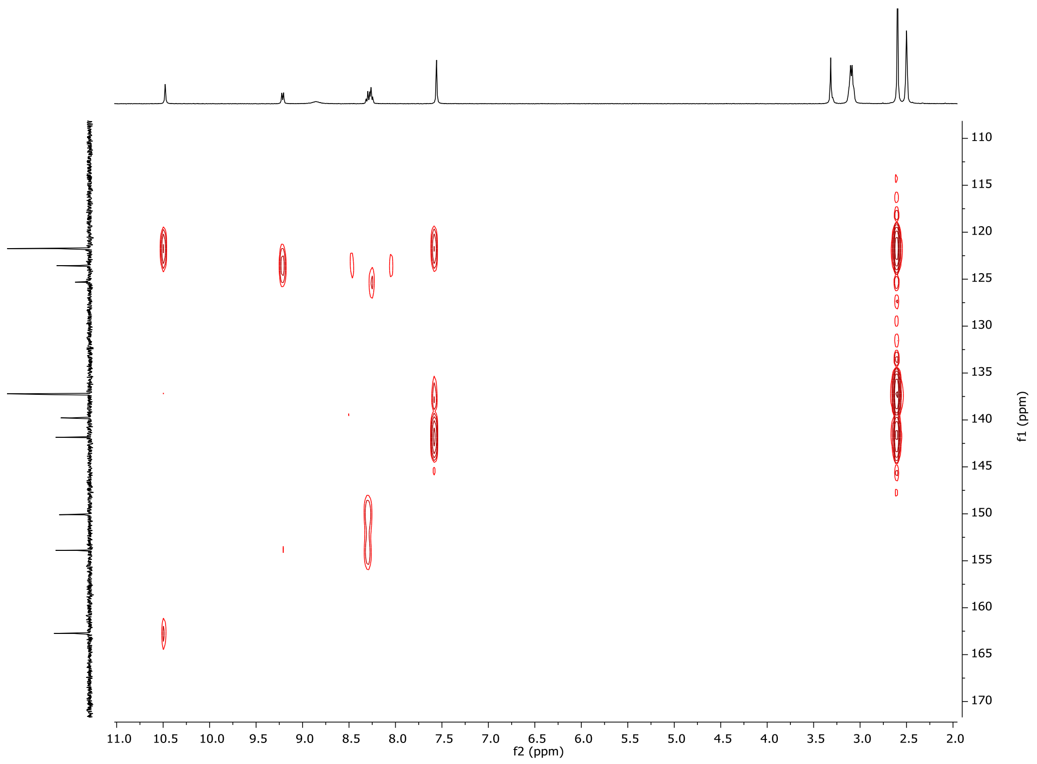

Figure S7. NMR spectra (DMSO-d_6, 500 MHz, 25 °C) for $\mathbf{H_2L6^{2-}}$. (A) ^{1}H-NMR, (B) ^{13}C NMR, (C) COSY, (D) HSQC and (E) HMBC.

A

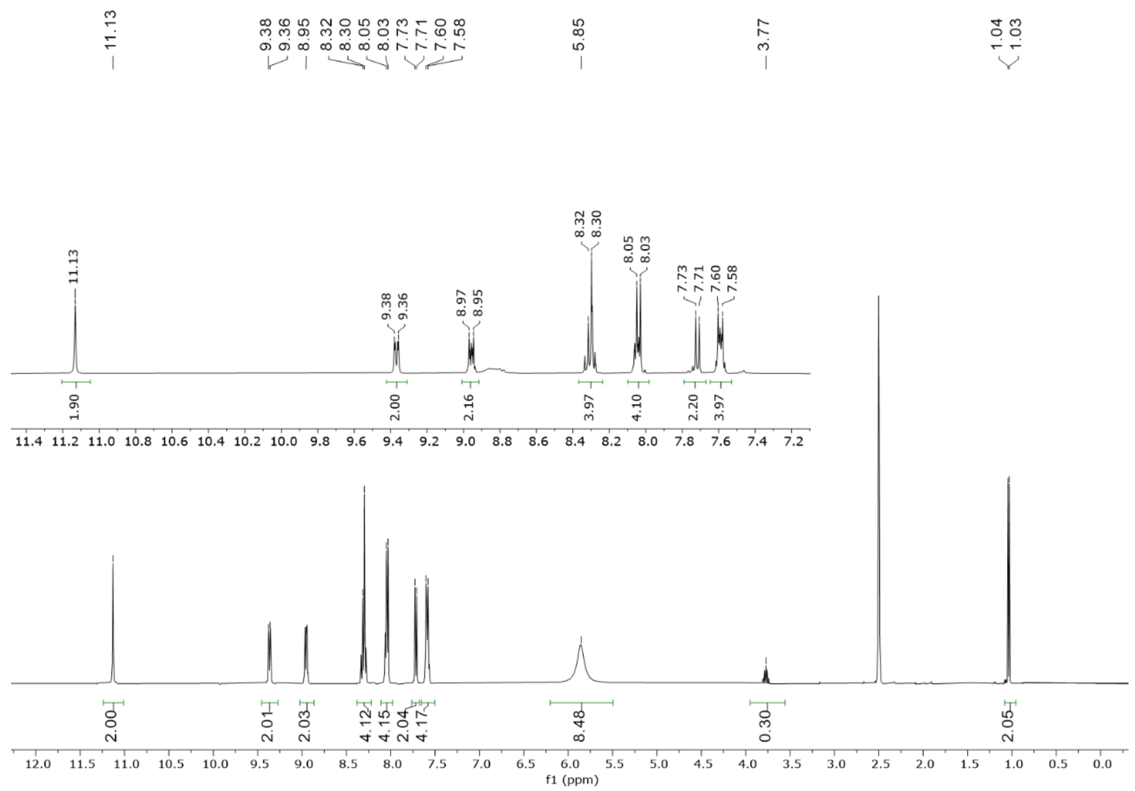
11.13
9.38
9.36
8.95
8.32
8.30
8.05
8.03
7.73
7.71
7.60
7.58
5.85
3.77
1.04
1.03
f1 (ppm)

B

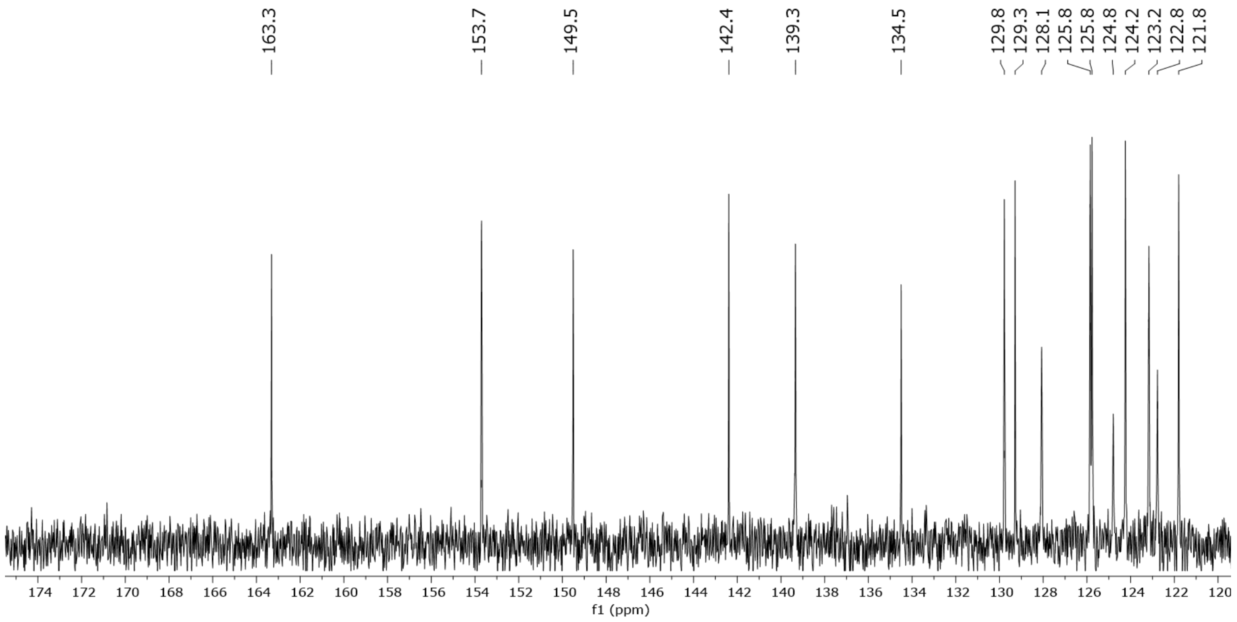
163.3
153.7
149.5
142.4
139.3
134.5
129.8
129.3
128.1
125.8
125.8
124.8
124.2
123.2
122.8
121.8
f1 (ppm)

C

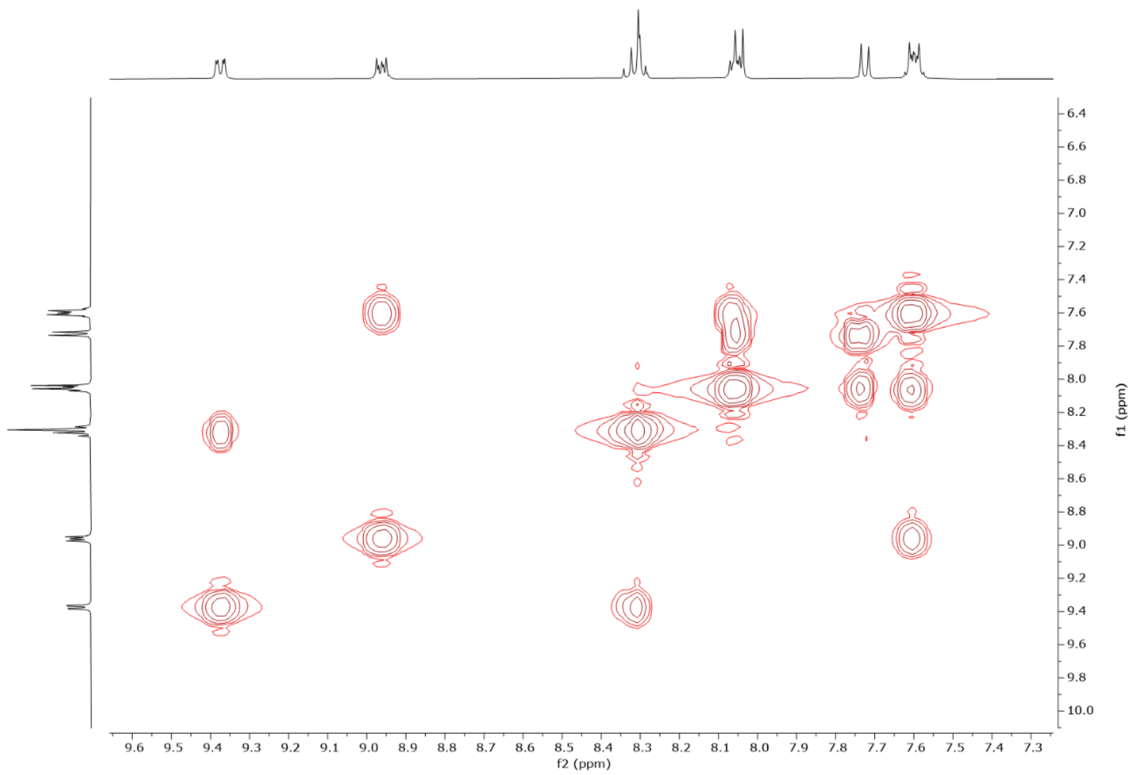
f1 (ppm)
f2 (ppm)
6.4
6.6
6.8
7.0
7.2
7.4
7.6
7.8
8.0
8.2
8.4
8.6
8.8
9.0
9.2
9.4
9.6
9.8
10.0
9.6 9.5 9.4 9.3 9.2 9.1 9.0 8.9 8.8 8.7 8.6 8.5 8.4 8.3 8.2 8.1 8.0 7.9 7.8 7.7 7.6 7.5 7.4 7.3

D

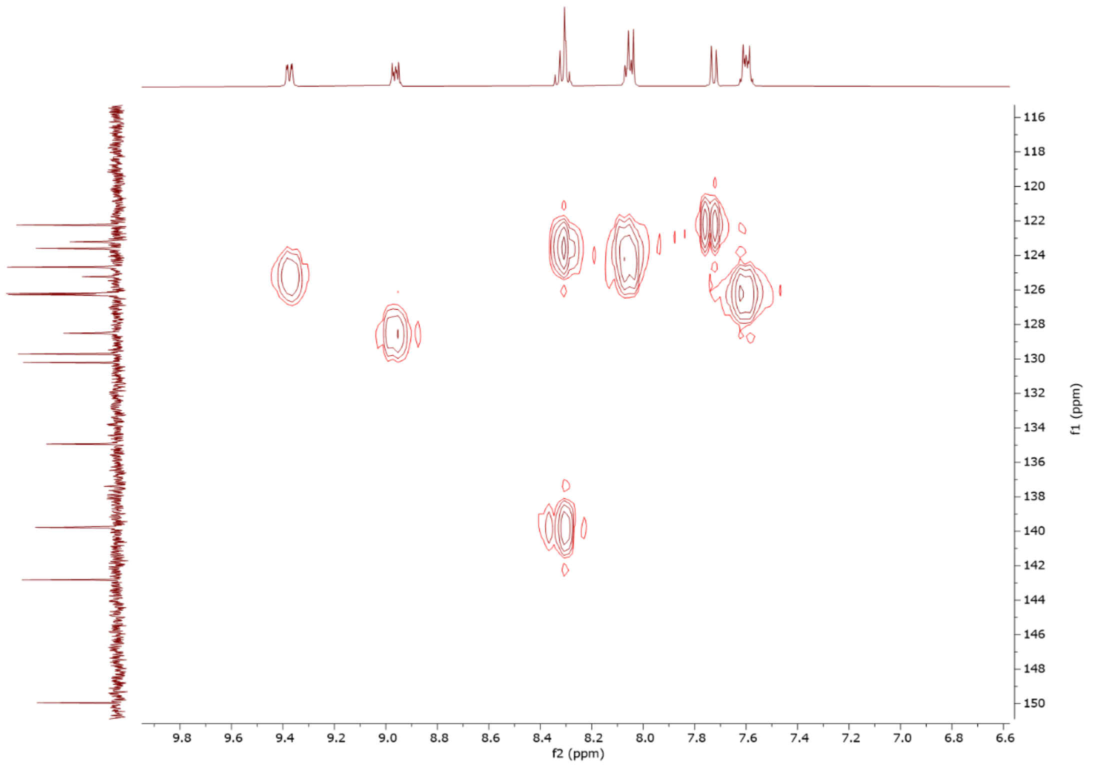
f1 (ppm)
f2 (ppm)
116
118
120
122
124
126
128
130
132
134
136
138
140
142
144
146
148
150
9.8 9.6 9.4 9.2 9.0 8.8 8.6 8.4 8.2 8.0 7.8 7.6 7.4 7.2 7.0 6.8 6.6

E

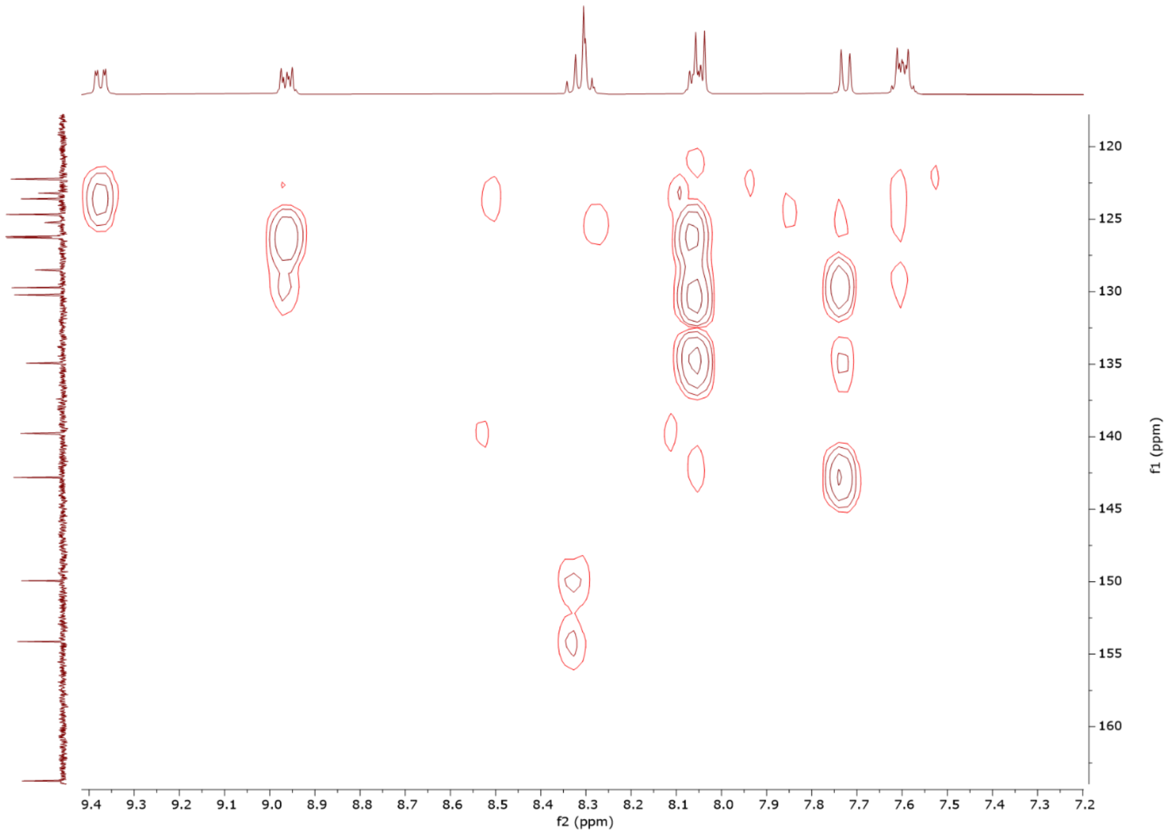

Figure S8. NMR spectra (DMSO-d_6, 500 MHz, 25 °C) for **H_2L7^{2-}**. (A) ^{1}H-NMR, (B) ^{13}C NMR, (C) COSY, (D) HSQC and (E) HMBC.

A

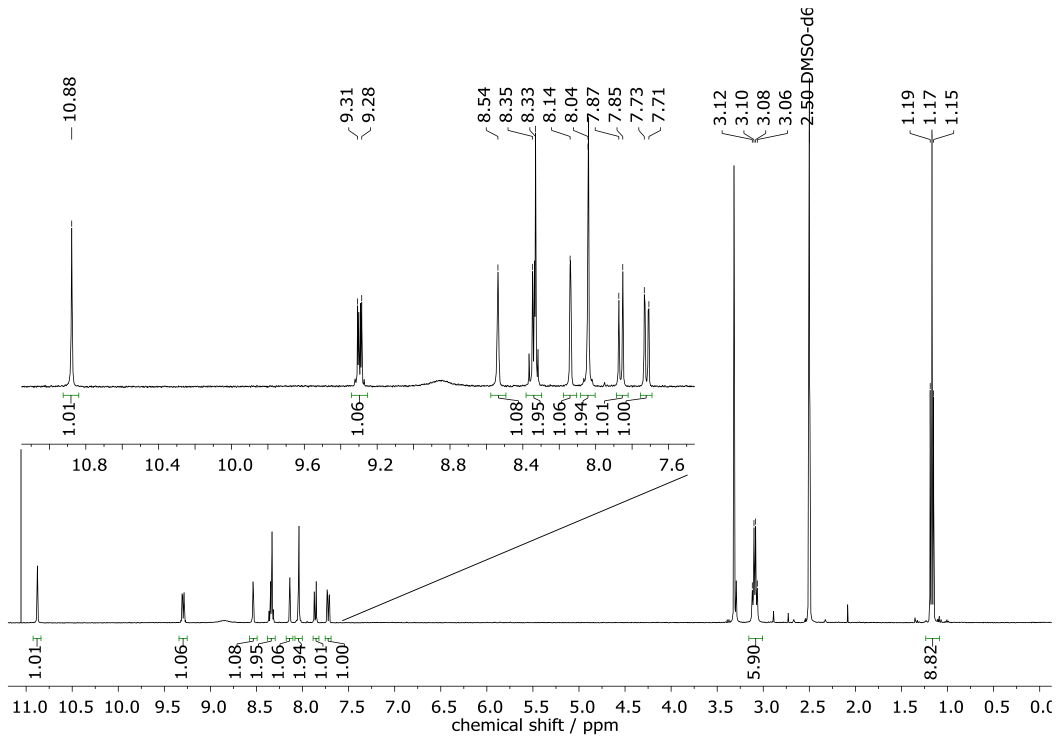
10.88
9.31
9.28
8.54
8.35
8.33
8.14
8.04
7.87
7.85
7.73
7.71
3.12
3.10
3.08
3.06
2.50 DMSO-d6
1.19
1.17
1.15
1.01
1.06
1.08
1.95
1.06
1.94
1.01
1.00
5.90
8.82
chemical shift / ppm

B

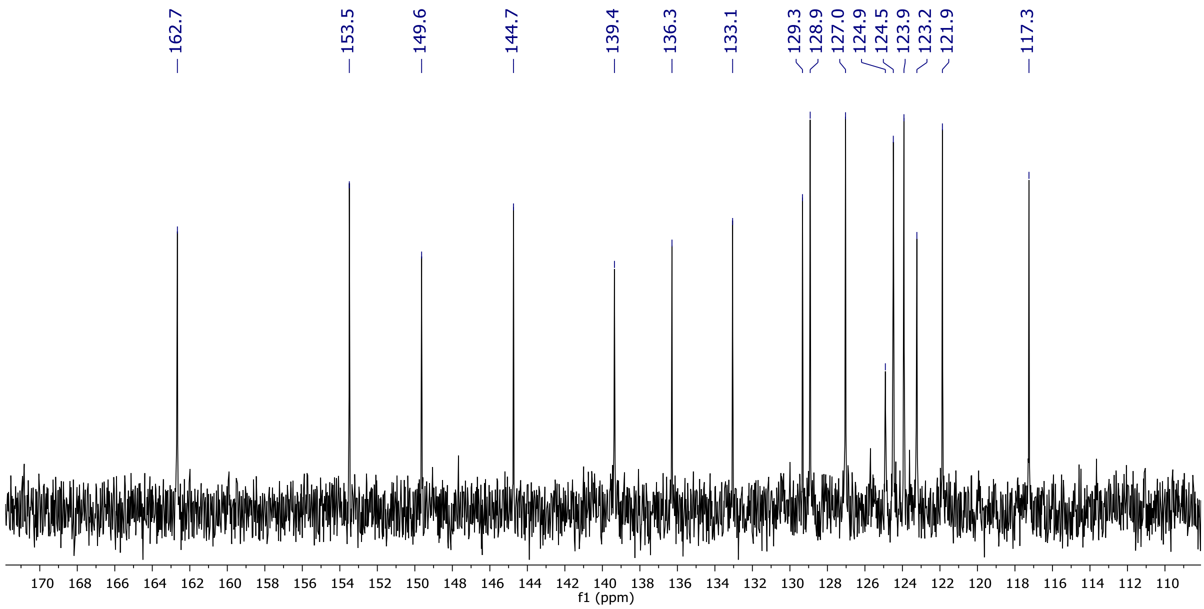
162.7
153.5
149.6
144.7
139.4
136.3
133.1
129.3
128.9
127.0
124.9
124.5
123.9
123.2
121.9
117.3
f1 (ppm)

C

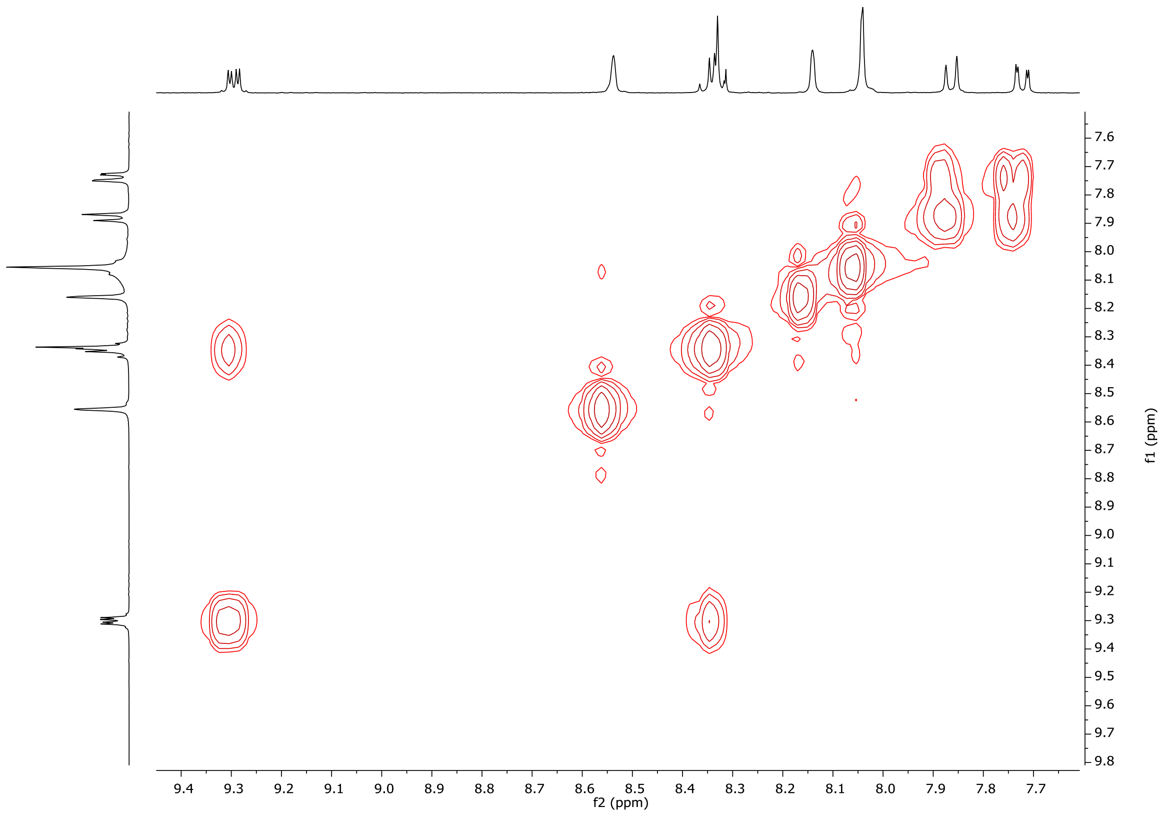

D

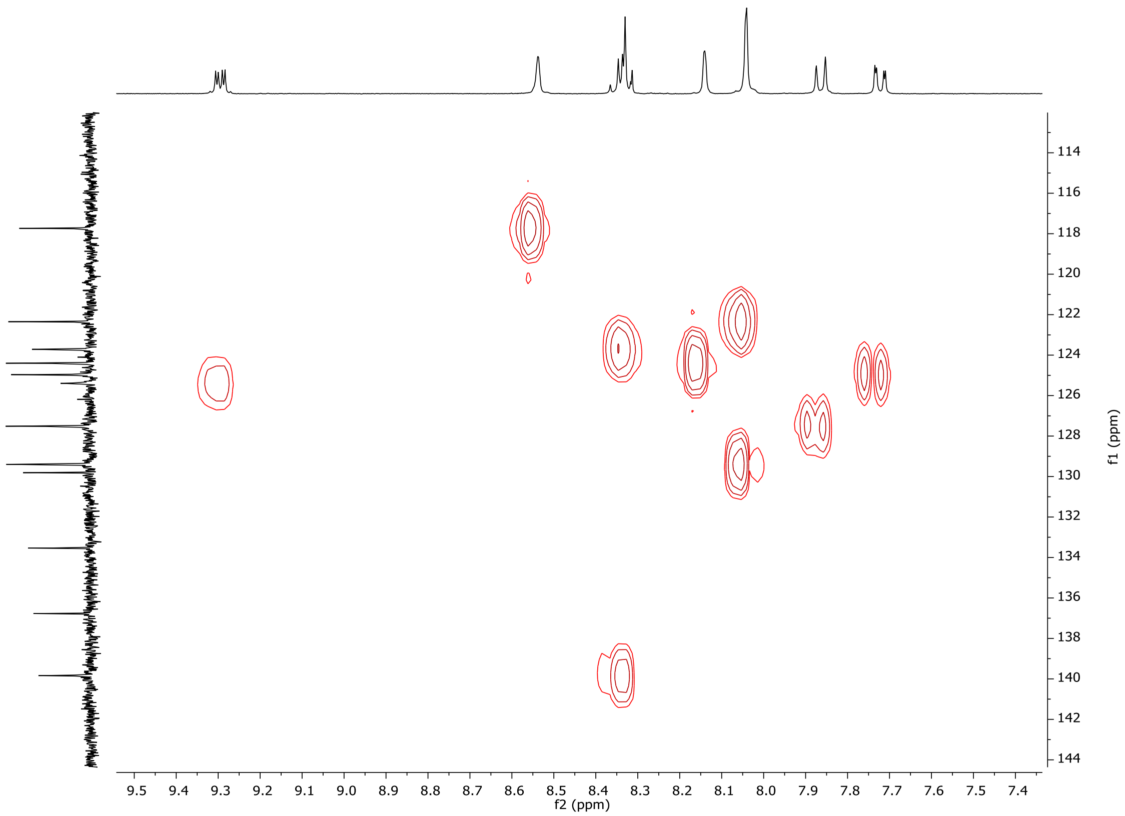

E

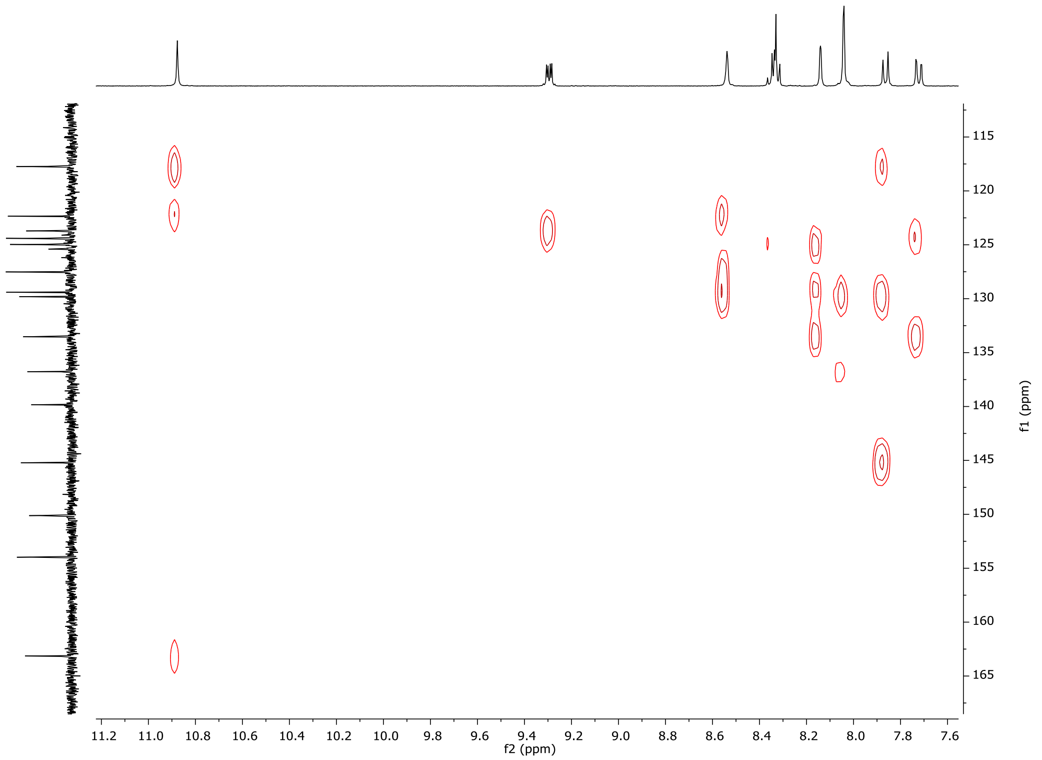

Figure S9. NMR spectra (DMSO-d_6, 500 MHz, 25 °C) for **H_2L8^{2-}**. (A) ^{1}H-NMR, (B) ^{13}C NMR, (C) COSY, (D) HSQC and (E) HMBC.

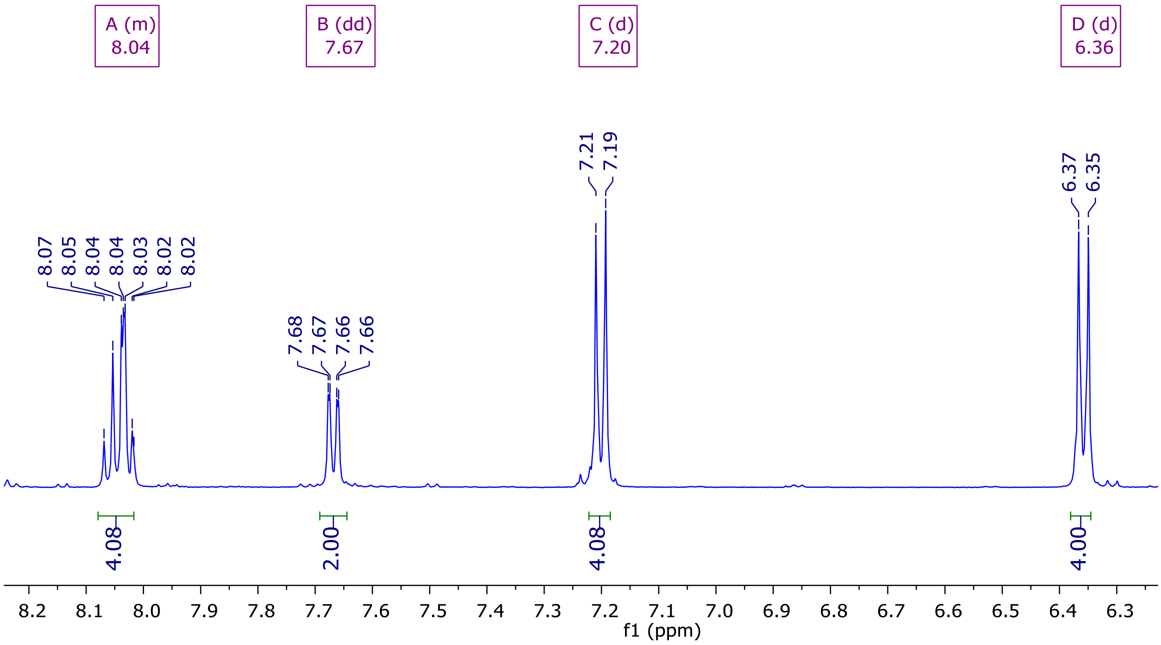

Figure S10. ^{1}H-NMR spectrum of the complex **$[(L4)Zn]^{2-}$** (D_2O-d_2, 500 MHz, 25 °C).

Mass-spectrometry

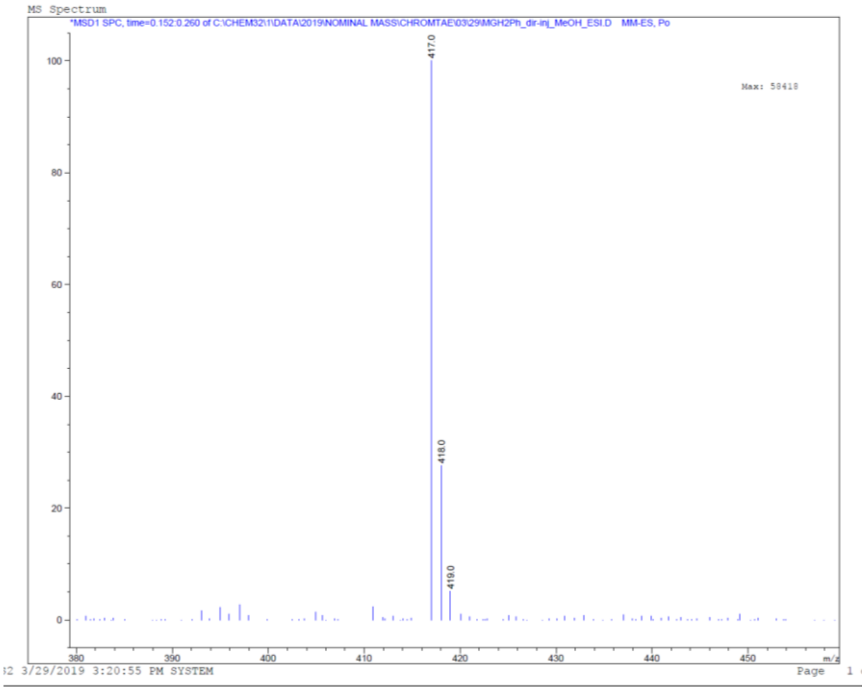

Figure S11. (+)-ESI-MS for **H_2L1**. m/z: 417.0 [H_2L1+Na$^+$]$^+$

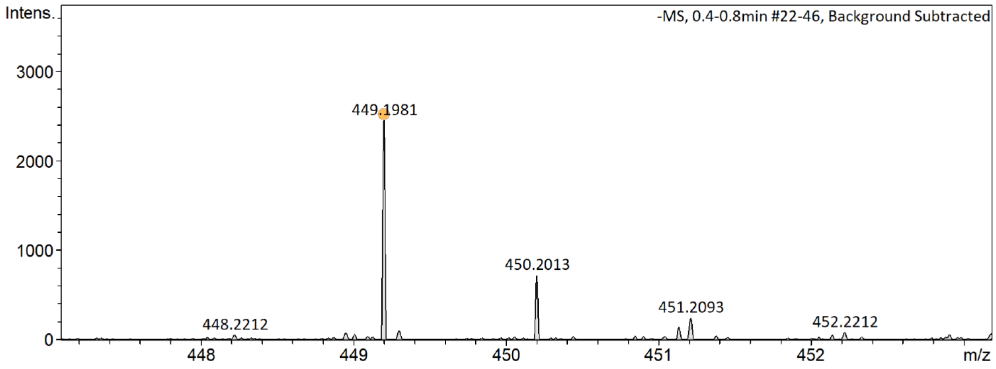

Figure S12. (+)-ESI-HRMS for **H_2L2**. Calcd for [H_2L2-H$^+$]$^+$, ($C_{28}H_{25}N_4O_2$) :449.1983, found: 449.1981.

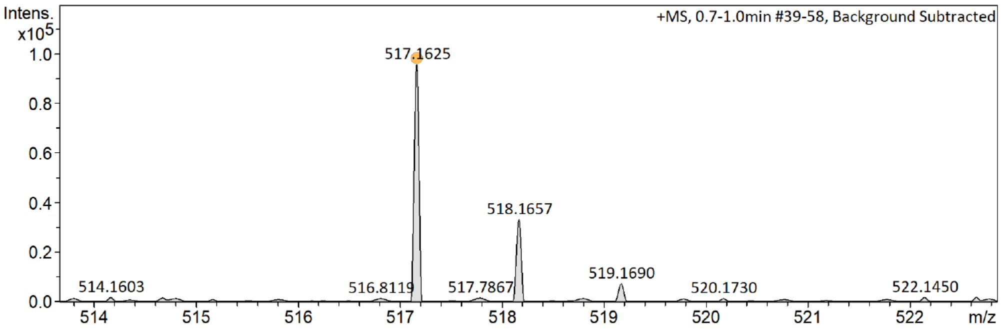

Figure S13. (+)-ESI-HRMS for **H_2L3**. Calcd for [H_2L3+Na$^+$]$^+$, ($C_{32}H_{22}N_4O_2$Na): 517.1635, found: 517.1625.

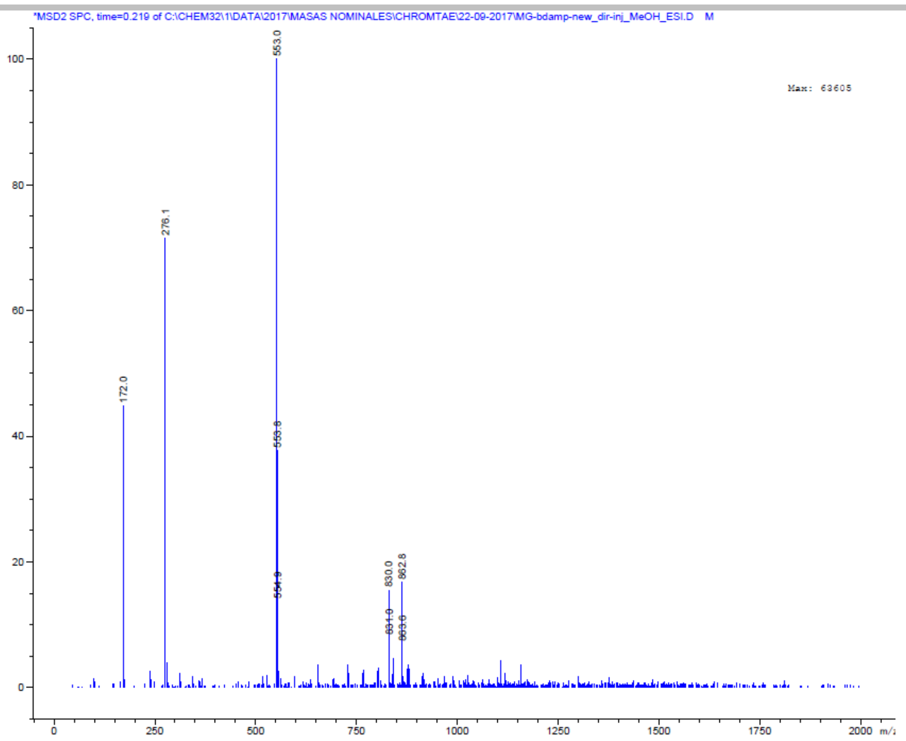

Figure S14. (-)-ESI-MS spectrum for $\mathbf{H_2L4^{2-}}$. m/z: 553.0 $[H_2L4+H^+]^-$, 276.1 $[H_2L4]^{2-}$.

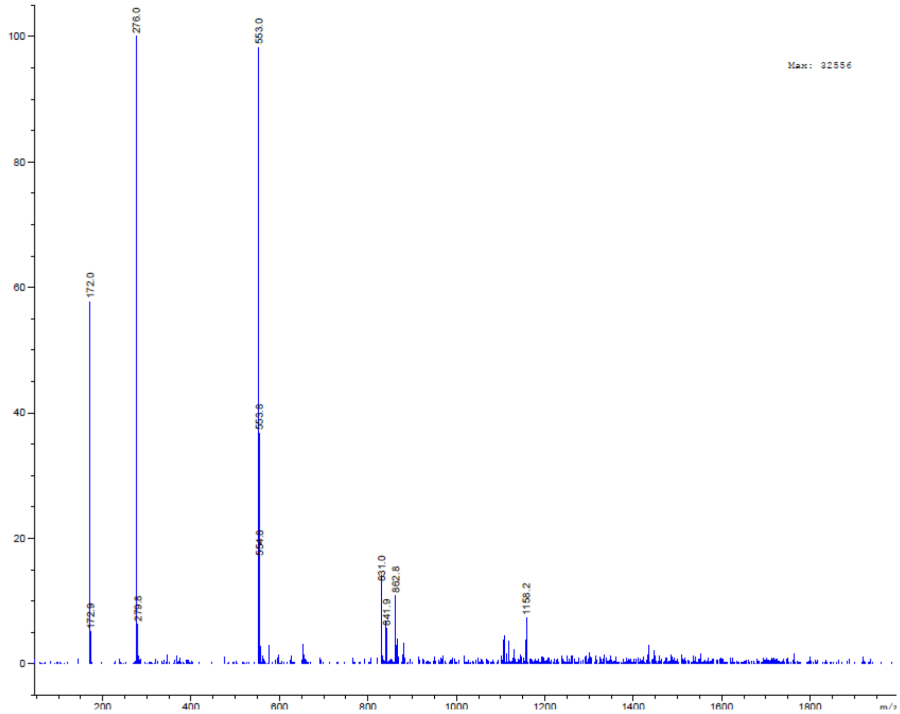

Figure S15. (-)-ESI-MS spectrum for the ligand $\mathbf{H_2L5^{2-}}$. m/z: 553.0 $[H_2L5+H^+]^-$, 276.1 $[H_2L5]^{2-}$

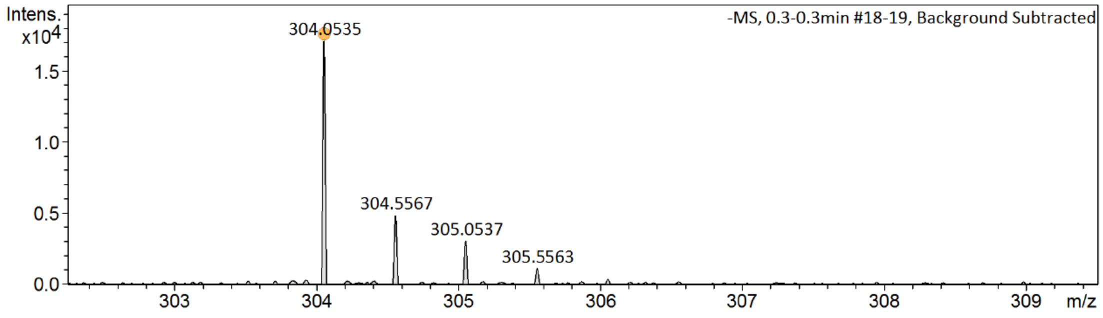

Figure S16. (-)-ESI-HRMS for **H_2L6^{2-}**. Calcd for $[H_2L6]^{2-}$, ($C_{28}H_{24}N_4O_8S_2$) : 304.0523, found 304.0535.

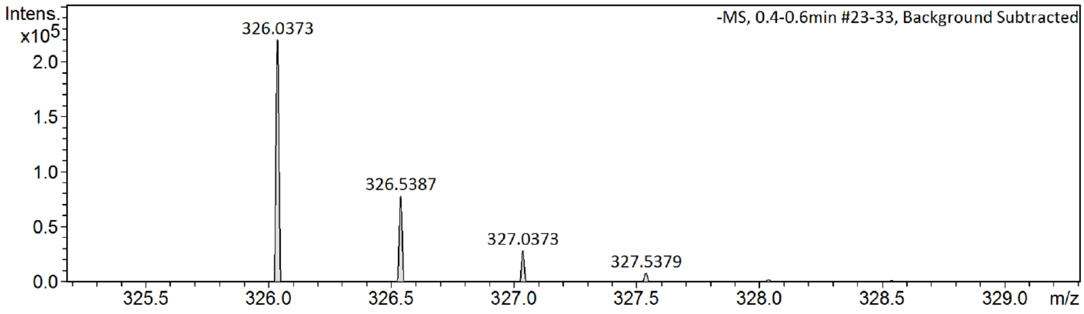

Figure S17. (-)-ESI-MS for **H_2L7^{2-}**. Calcd for $[H_2L7]^{2-}$, ($C_{32}H_{22}N_4O_8S_2$) : 326.0367, found 326.0373.

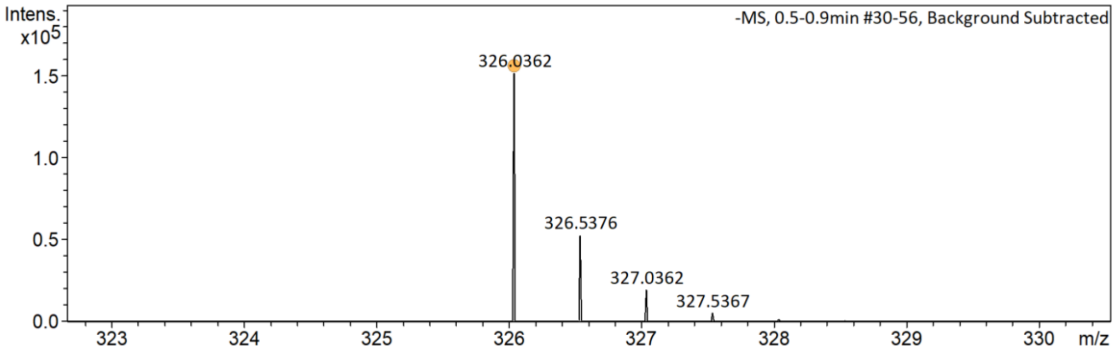

Figure S18. (-)-ESI-MS for **H_2L8^{2-}**. Calcd for $[H_2L8]^{2-}$, ($C_{32}H_{22}N_4O_8S_2$) : 326.0367, found 326.0362.

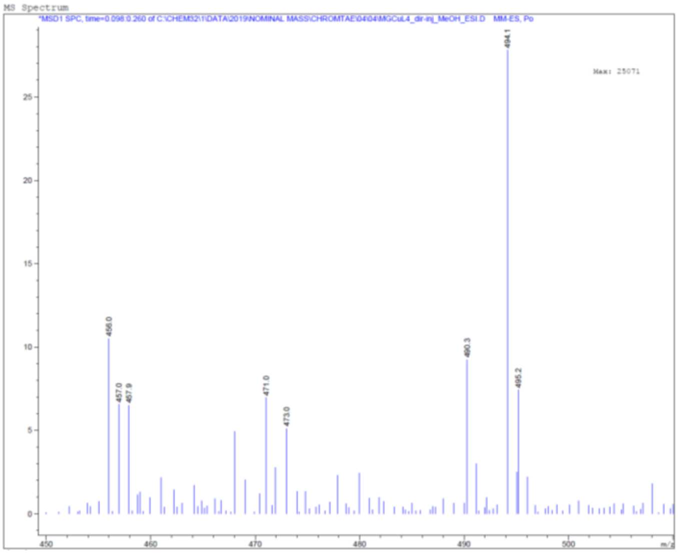

Figure S19. (+)-ESI-MS for **[(L1)Cu]**. m/z = 456.0 [(L1)Cu-H$^+$]$^+$; m/z = 494.1[(L1)Cu+K$^+$]$^+$.

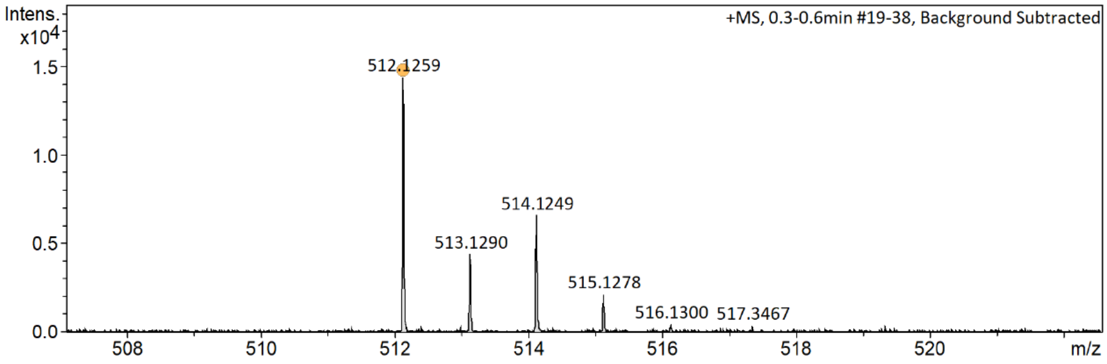

Figure S20. (+)-ESI-HRMS for **[(L2)Cu]**. Calcd for [(L2)Cu+H$^+$]$^+$, (C28H25N4O2Cu) : 512.1259, found 512.1268.

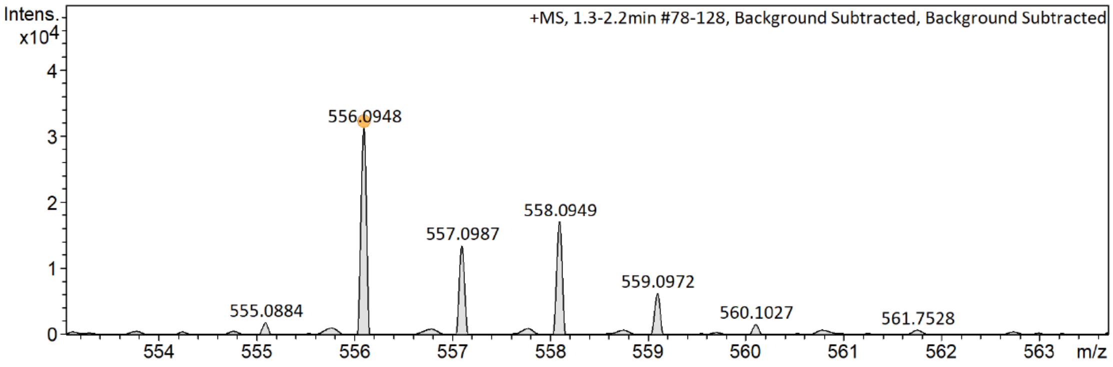

Figure S21. (-)-ESI-HRMS for **[(L3)Cu]**. Calcd for [(L3)Cu+H$^+$]$^+$, ($C_{32}H_{21}CuN_4O_2$): 556.0955, found: 556.0948.

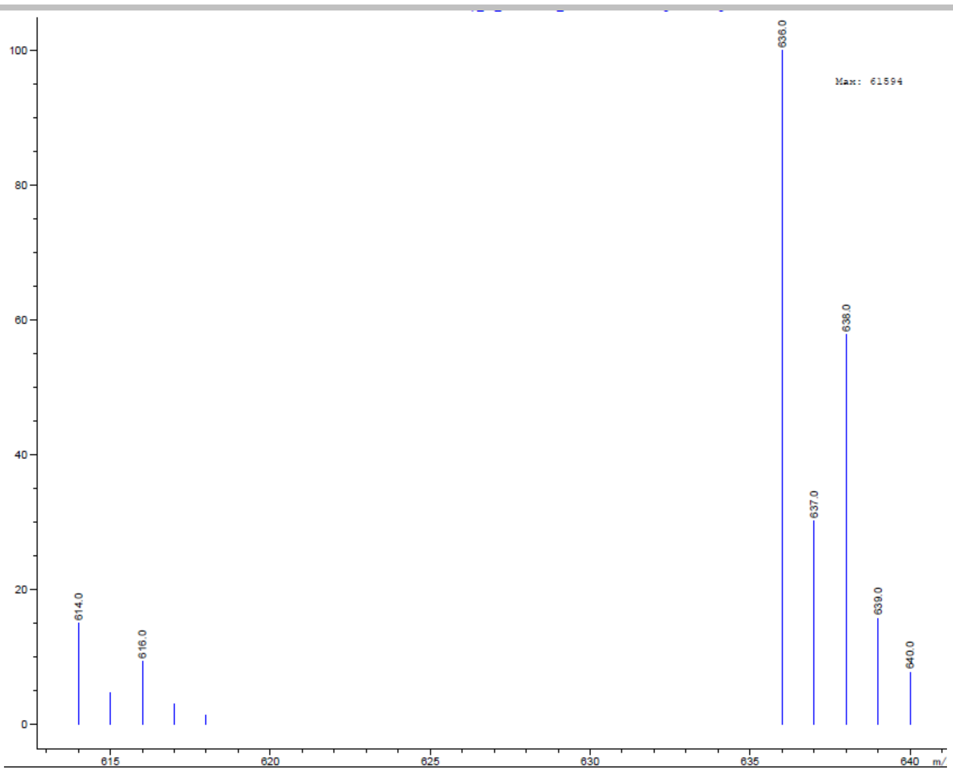

Figure S22. (-)-ESI-MS for **$[(L4)Cu]^{2-}$**. m/z: 636.0 $[(L4)Cu+Na^{+}]^{-}$, 614.0 $[(L4)Cu+H^{+}]^{-}$.

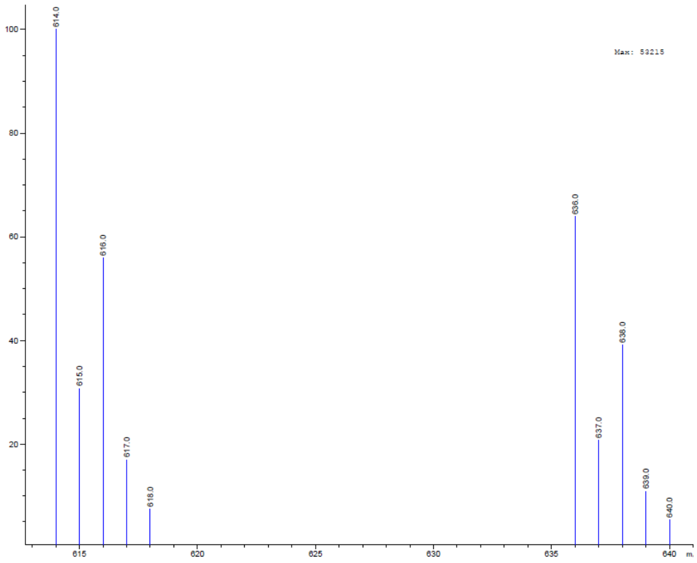

Figure S23. (-)-ESI-MS for **$[(L5)Cu]^{2-}$**. m/z: 636.0 $[(L5)Cu+Na^{+}]^{-}$, 614.0 $[(L5)Cu+H^{+}]^{-}$.

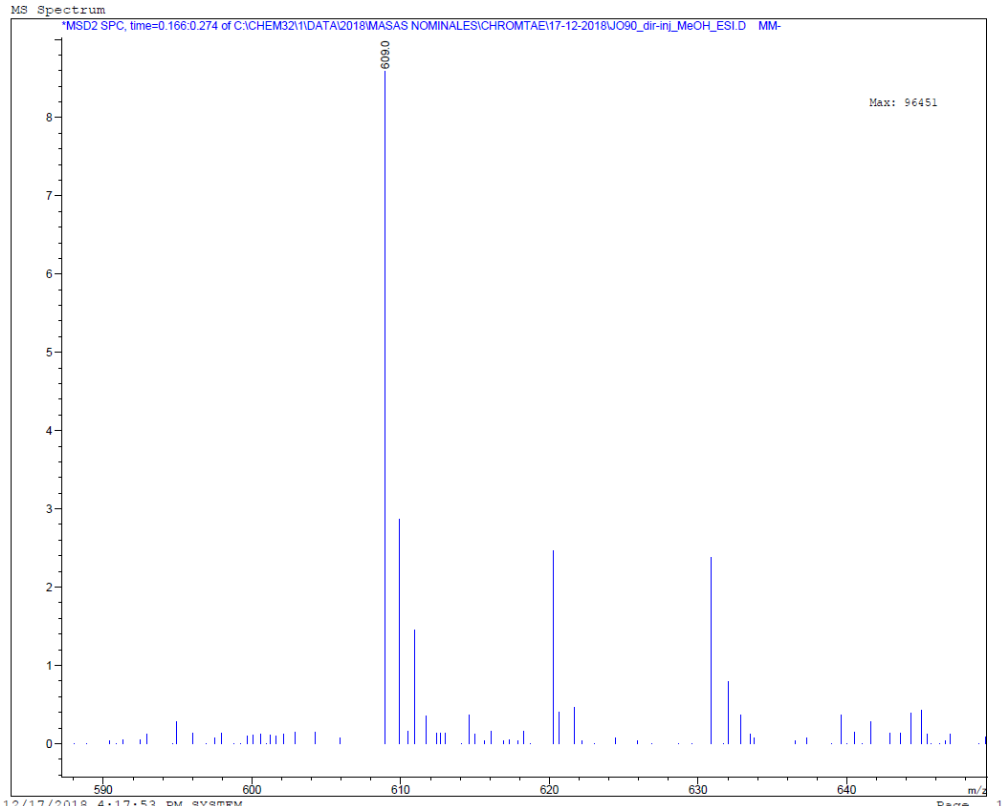

Figure S24. (-)-ESI-MS for **[(L6)Cu]$^{2-}$**. m/z = 609.0 [(L6)Cu-Na^{+}]$^{-}$.

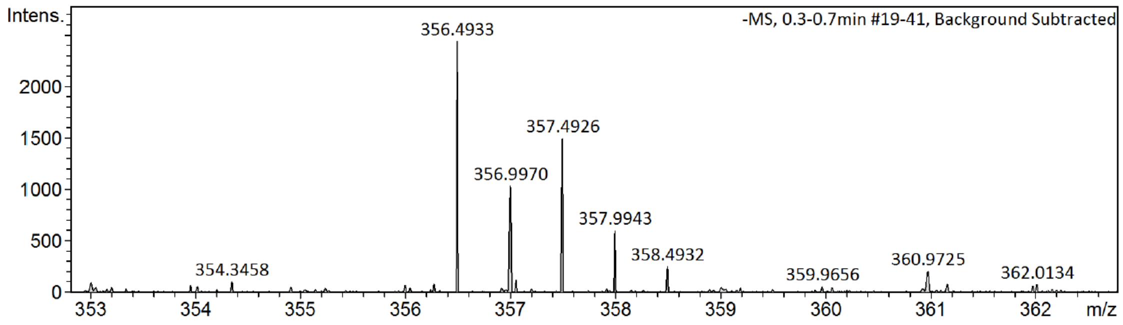

Figure S25. (-)-ESI-HRMS for **[(L7)Cu]$^{2-}$**. Calcd for [(L7)Cu]$^{2-}$, ($C_{32}H_{18}CuN_4O_8S_2$) : 356.4937, found 356.4933.

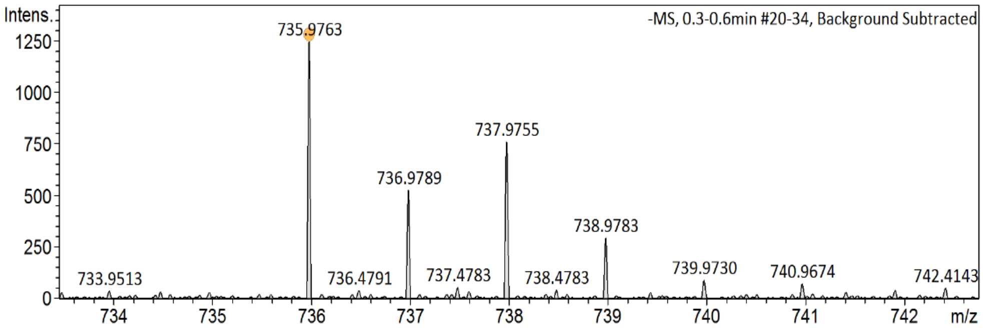

Figure S26. (-)-ESI-HRMS for **[(L8)Cu]$^{2-}$**. Calcd for [(L8)Cu+Na^{+}]$^{-}$, ($C_{32}H_{18}CuN_4O_8S_2Na$) : 735.9765, found 735.9763.

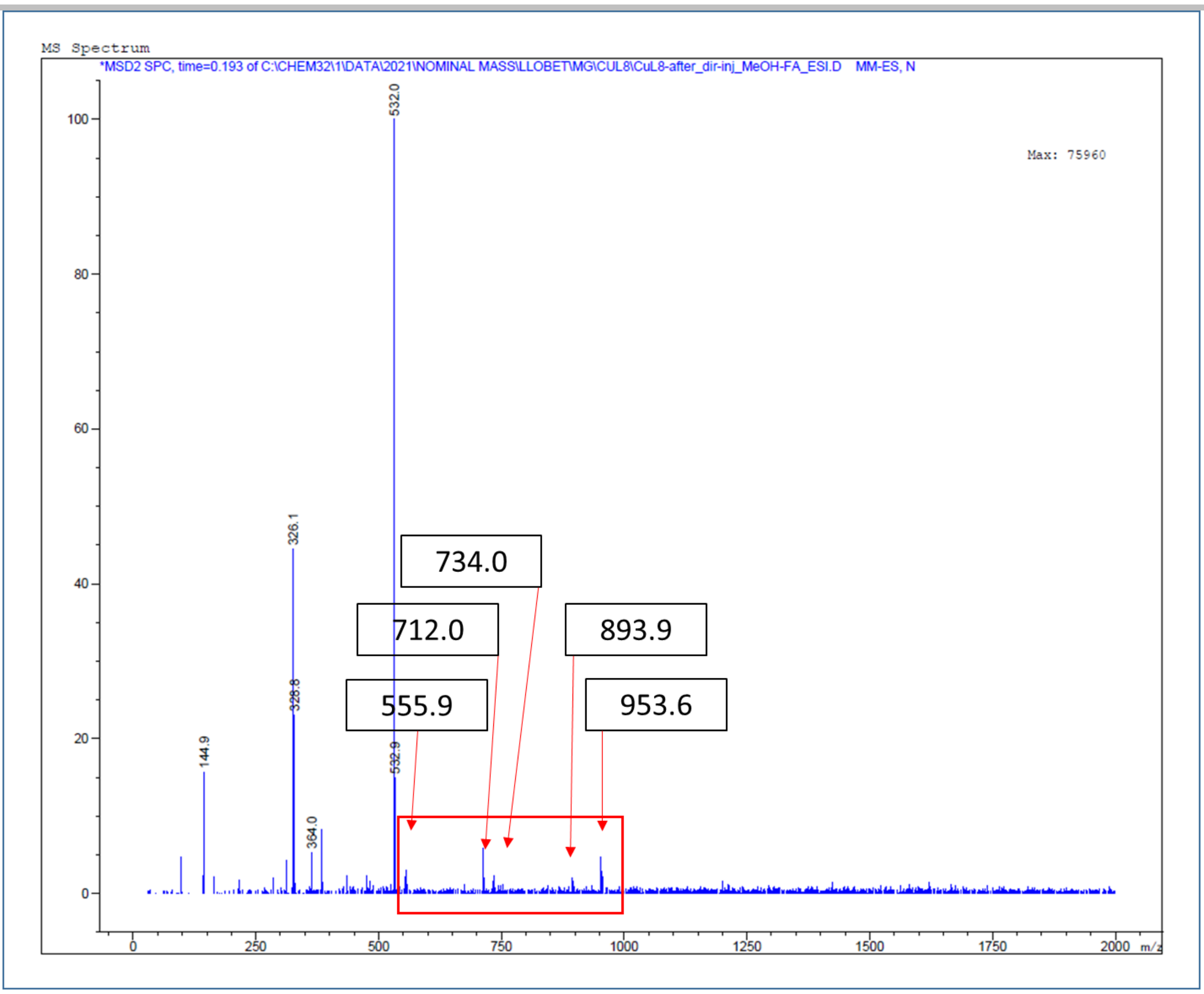

Figure S27. (-)-ESI-MS spectrum for **[(L8)Cu]**$^{2-}$ after a CPE experiment at 1.45 V vs. NHE in 0.01 M phosphate buffer pH 11.6 during 12 h. The spectrum shows peaks at m/z= 555.9, 712.0, 734.0, 893.9 and 953.6 can be potentially assigned to molecular Cu degradation products based on the isotopic cracking pattern. The peak at m/z= 712.0 is assigned in Figure S28.

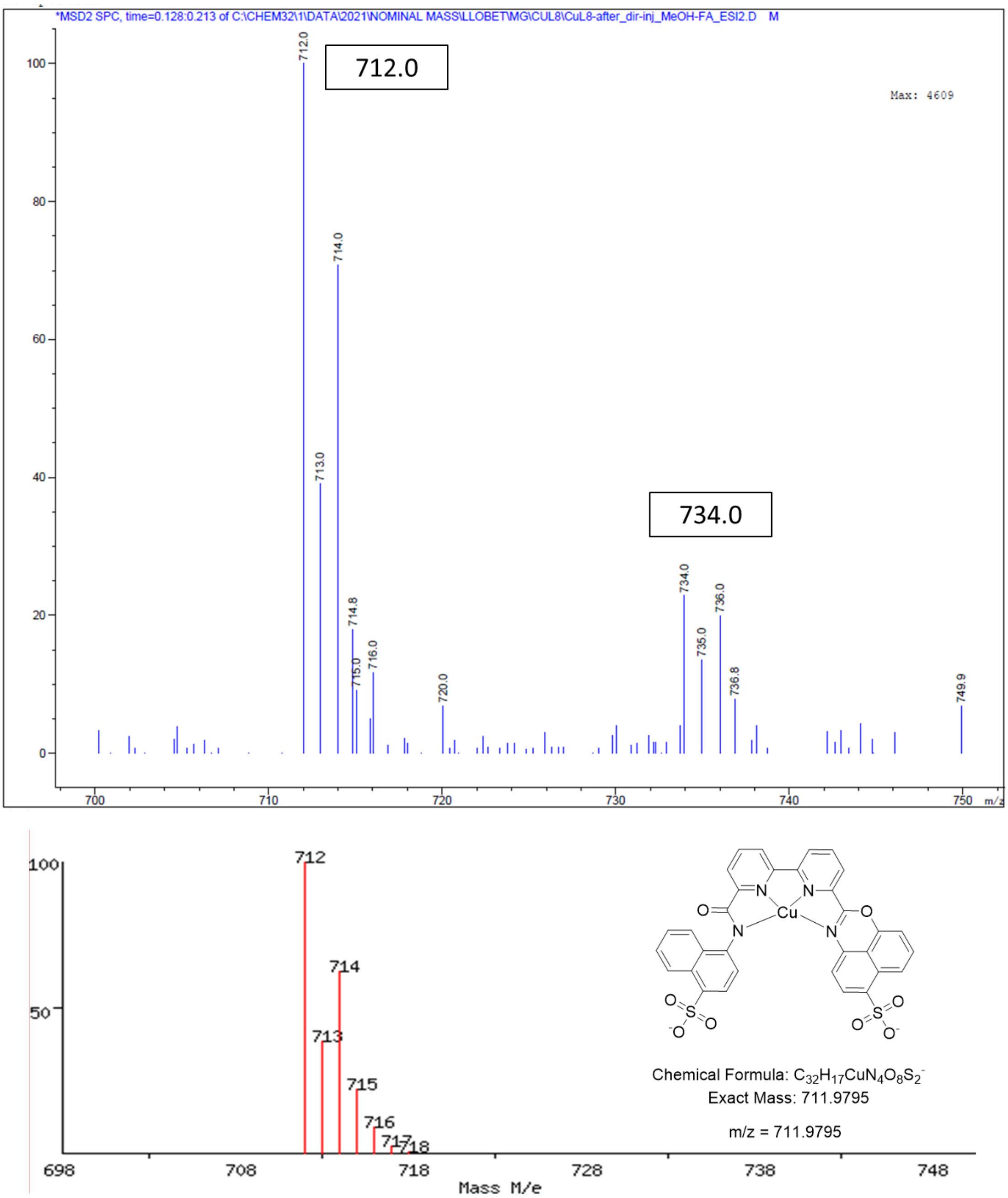

Figure 28. Top, zoom in the m/z= 700-750 region of (-)-ESI-MS spectrum from Figure S27. Bottom, simulation and molecular assignment of the m/z =712 peak.

UV-Vis spectroscopy

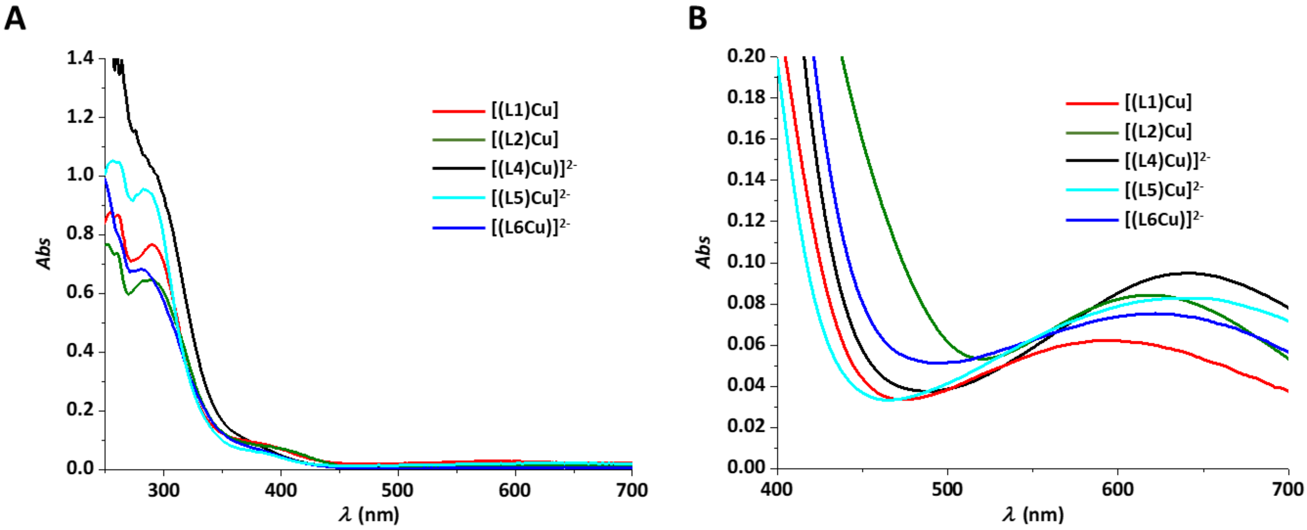

Figure S29. (**A**) UV-vis spectra of **[(L1)Cu]** (red line), **[(L2)Cu]** (green line), **[(L4)Cu]$^{2-}$** (black line), **[(L5)Cu]$^{2-}$** (light blue line) and **[(L6)Cu]$^{2-}$** (blue line). (**B**) UV-vis spectra enlargement of the visible region. Conditions: [**Complex**] = ~0.1 mM in 0.1 M phosphate buffer (pH 11.6). ***Note:*** in order to solve low solubility issues with **[(L1)Cu]** and **[(L2)Cu]**, 40% of TFE was added to the mixture used for UV-Vis measurements.

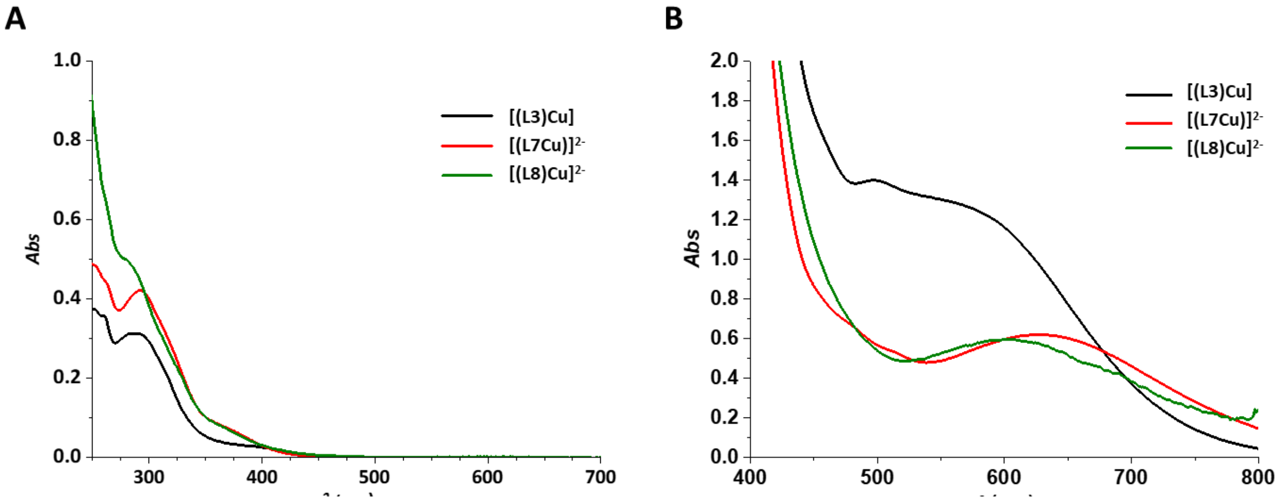

Figure S30. (**A**) UV-vis spectra of **[(L3)Cu]** (black line), **[(L7)Cu]$^{2-}$** (red line) and **[(L8)Cu]$^{2-}$** (green line). (**B**) UV-vis spectra enlargement of the visible region. Conditions: **[Complex]** = 0.04 mM (A, *d*= 1 mm) and 1 mM (B, *d*= 10 mm) in 0.1 M phosphate buffer (pH 11.6). ***Note:*** in order to solve low solubility issues with **[(L3)Cu]** 40% of TFE was added to the mixture used for UV-Vis measurements.

X-Ray Crystallography

Crystal preparation:

Table S1. Summary of the conditions employed for the crystallization of the complexes studied in this work.

Compound	Methodology
**H_2L5^{2-}**	Crystals were grown in water by slow diffusion of EtOH.
**[(L1)Cu]**	Crystals were grown by slow evaporation in MeOH/Toluene.
**[(L2)Cu]**	Crystals were grown in MeOH by slow diffusion of Et_2O.
**[(L3)Cu]**	Crystals were grown by slow evaporation in MeOH/Toluene.
**$[(L4)Cu]^{2-}$**	Crystals were grown in water by slow diffusion of acetone.
**$[(L5)Cu]^{2-}$**	Crystals were grown in water by slow diffusion of acetone.
**$[(L6)Cu]^{2-}$**	Crystals were grown in MeOH by slow diffusion of layered isopropanol.
**$[(L7)Cu]^{2-}$**	Crystals were grown in water by slow diffusion of acetone.

The crystals were selected using a Zeiss stereomicroscope using polarized light and prepared under inert conditions immersed in perfluoropolyether as protecting oil for manipulation.

Data collection: Crystal structure determinations for samples were carried out using an Apex DUO Kappa 4-axis goniometer equipped with an APPEX 2 4K CCD area detector, a Microfocus Source E025 IuS using MoK_α radiation, a Quazar MX multilayer Optics as monochromator and an Oxford Cryosystems low temperature device Cryostream 700 plus (T = -173 °C). Full-sphere data collection was used with ω and φ scans. *Programs used:* Bruker Device: Data collection APEX-2,[7] data reduction Bruker Saint[8] V/.60A and absorption correction SADABS.[9]

Structure Solution and Refinement: Crystal structure solution was achieved using the computer program SHELXT.[10] Visualization was performed with the program SHELXle.[11] Missing atoms were subsequently located from difference Fourier synthesis and added to the atom list. Least-squares refinement on F^2 using all measured intensities was carried out using the program SHELXL 2015.[12] All non-hydrogen atoms were refined including anisotropic displacement parameters.

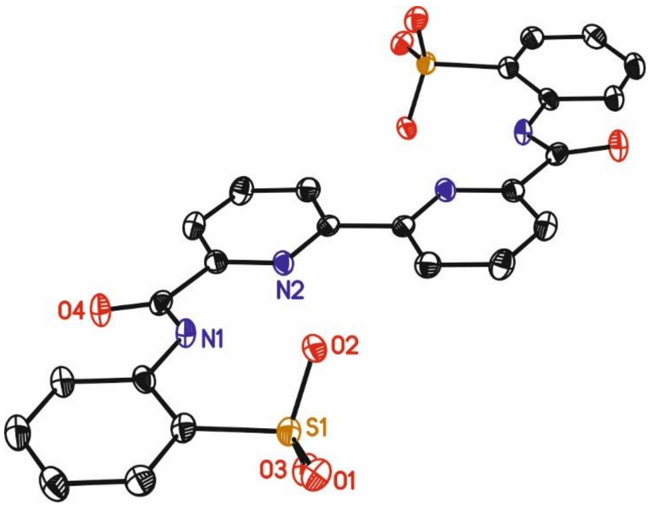

Figure S31. ORTEP representation of **H_2L5^{2-}** at 50% probability level. The counter ions, solvent molecules and hydrogen atoms have been omitted for clarity. Color code: C, black; N, blue; O, red; S, yellow.

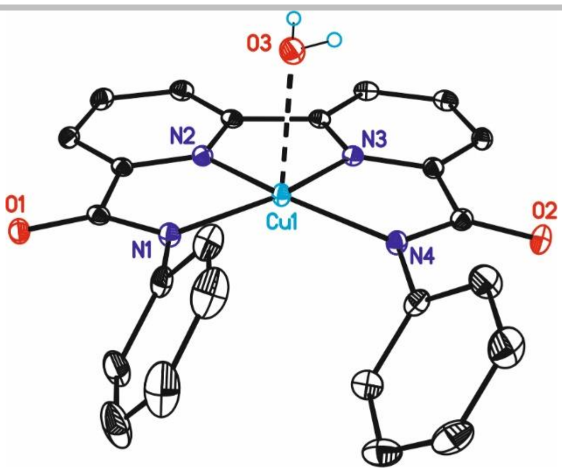

Figure S32. ORTEP representation of **[(L1)Cu]** at 50% probability level. The counter ions, solvent molecules and hydrogen atoms have been omitted for clarity. Color code: C, black; N, blue; O, red; Cu, light blue. The ORTEP drawing shows a contact (2.38 Å) with an oxygen from a H_2O molecule in the apical position.

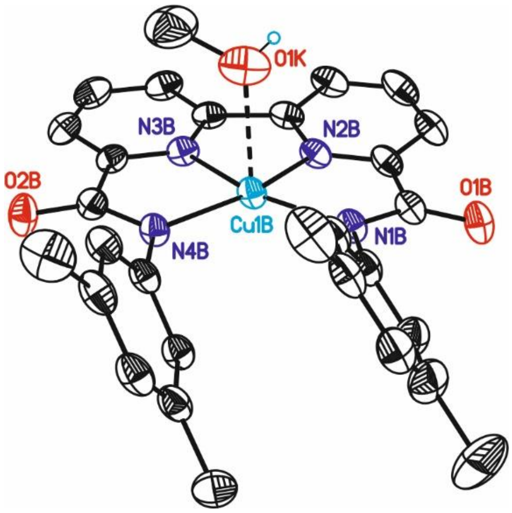

Figure S33. ORTEP representation of **[(L2)Cu]** at 50% probability level. The counter ions, solvent molecules and hydrogen atoms have been omitted for clarity. Color code: C, black; N, blue; O, red; Cu, light blue. The ORTEP drawing shows a contact (2.47 Å) with an oxygen from a MeOH molecule in the apical position.

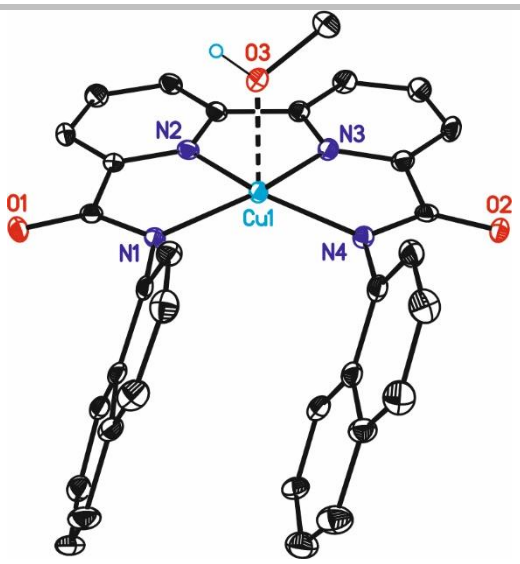

Figure S34. ORTEP representation of **[(L3)Cu]** at 50% probability level. The counter ions, solvent molecules and hydrogen atoms have been omitted for clarity. Color code: C, black; N, blue; O, red; Cu, light blue. The ORTEP drawing shows a contact (2.25 Å) with an oxygen from a MeOH molecule in the apical position.

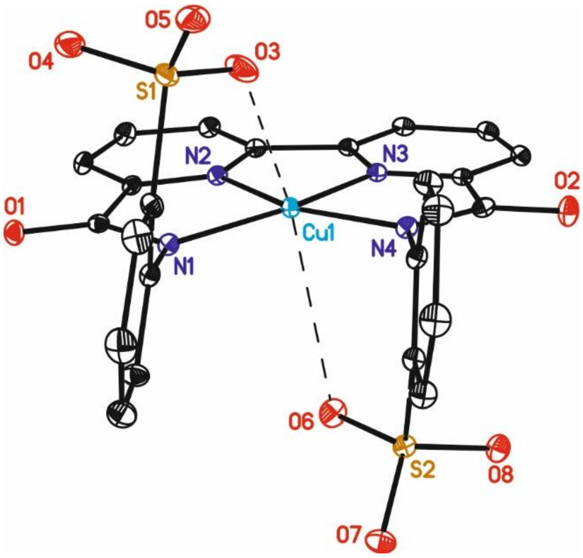

Figure S35. ORTEP representation of **[(L5)Cu]**$^{2-}$ at 50% probability level. The counter ions, solvent molecules and hydrogen atoms have been omitted for clarity. Color code: C, black; N, blue; O, red; Cu, light blue; S, yellow. The ORTEP drawing shows a contact (2.61 Å) between an oxygen from the sulfonate moiety (O3) and the Cu center in the apical position.

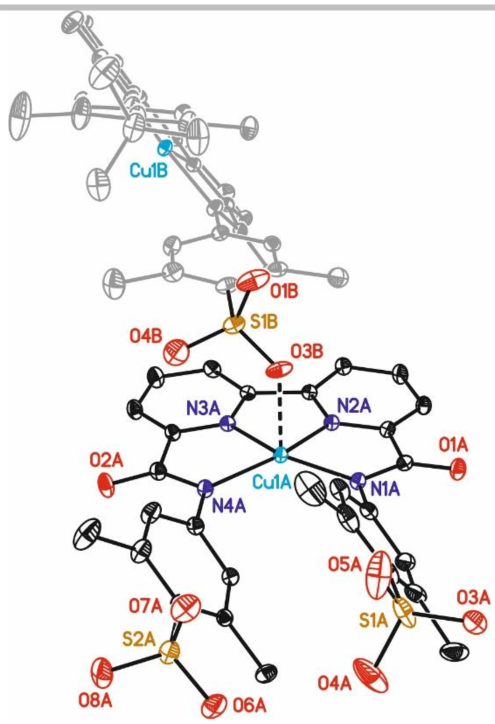

Figure S36. ORTEP representation of **[(L6)Cu]**$^{2-}$ at 50% probability level. The counter ions, solvent molecules and hydrogen atoms have been omitted for clarity. Color code: C, black; N, blue; O, red; Cu, light blue; S, yellow. The ORTEP drawing shows a contact (2.33 Å) with a sulfonate moiety from another **[(L6)Cu]**$^{2-}$ unit in the apical position (grey).

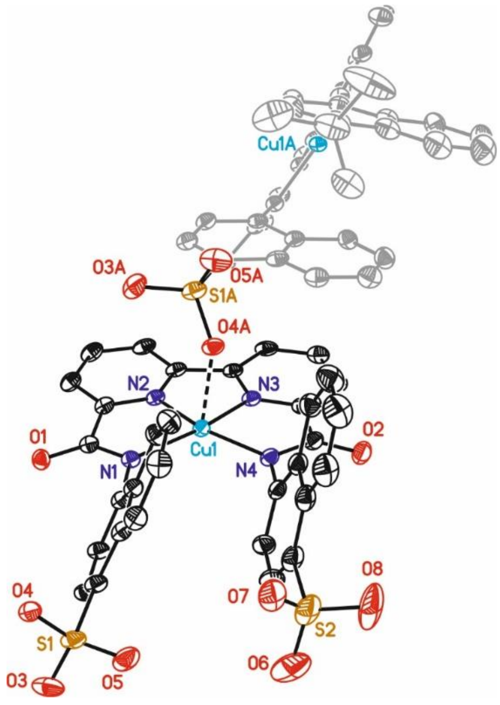

Figure S37. ORTEP representation of **[(L7)Cu]**$^{2-}$ at 50% probability level. The counter ions, solvent molecules and hydrogen atoms have been omitted for clarity. Color code: C, grey; N, blue; O, red; Cu, light blue; S, yellow. The ORTEP drawing shows a contact (2.38 Å) with a sulfonate moiety from another **[(L7)Cu]**$^{2-}$ unit in the apical position (grey).

Table S2. Selected interatomic bond distances.

	Cu-N_{bpy1}	Cu-N_{bpy2}	Cu-N_{amide1}	Cu-N_{amide2}	Cu-X_{apical}	N_{amide1}-C_{ar1}	N_{amide2}-C_{ar2}
[(L1)Cu]	1.9498(13)	1.9573(13)	1.9833(13)	2.0158(13)	2.3831(14)	1.422(2)	1.433(2)
[(L2)Cu]	1.9522(17)	1.9546(17)	1.9855(17)	1.9913(17)	2.423(3)	1.425(3)	1.423(2)
	1.9475(17)	1.9517(17)	1.9759(18)	1.9828(17)	2.475(2)	1.429(2)	1.421(2)
[(L3)Cu]	1.950(2)	1.9597(19)	1.9920(19)	2.012(5)	2.2455(18)	1.434(3)	1.421(6)
$[(L4)Cu]^{2-}$	1.9569(14)	1.9508(14)	2.0085(14)	2.0152(14)	2.3069(14)	1.415(2)	1.418(2)
$[(L5)Cu]^{2-}$	1.9515(14)	1.9473(13)	1.9632(14)	1.9868(14)	2.6110(13)	1.418(2)	1.424(2)
$[(L6)Cu]^{2-}$	1.9674(17)	1.9602(17)	1.9961(17)	2.0163(17)	2.324(6)	1.419(3)	1.414(3)
	1.9615(17)	1.9554(18)	1.9848(18)	2.0058(18)	2.339(3)	1.420(3)	1.421(3)
$[(L7)Cu]^{2-}$	1.943(2)	1.964(2)	2.012(2)	1.979(2)	2.382(2)	1.431(3)	1.421(4)

Table S3. Selected interatomic angles.

	N_{bpy1}-Cu-N_{bpy2}	N_{amide1}-Cu-N_{bpy1}	N_{bpy2}-Cu-N_{amide2}	N_{amide1}-Cu-N_{amide2}	N_{bpy1}-Cu-N_{amide2}	N_{bpy2}-Cu-N_{amide1}
[(L1)Cu]	78.02(5)	80.34(5)	80.94(5)	118.99(5)	156.79(6)	157.46(6)
[(L2)Cu]	77.41(7)	80.82(7)	81.09(6)	119.66(7)	158.19(7)	156.42(7)
	77.96(7)	81.09(7)	80.83(7)	119.73(7)	157.90(7)	158.95(7)
[(L3)Cu]	77.99(8)	80.74(8)	80.77(15)	118.44(14)	157.09(19)	156.92(8)
$[(L4)Cu]^{2-}$	78.27(6)	80.12(6)	80.74(6)	119.69(6)	158.81(6)	155.51(6)
$[(L5)Cu]^{2-}$	78.49(6)	80.16(6)	80.56(6)	120.78(6)	159.03(6)	158.00(6)
$[(L6)Cu]^{2-}$	77.43(7)	80.58(7)	80.53(7)	120.23(7)	156.08(7)	157.57(7)
	77.76(7)	80.80(7)	80.58(7)	119.75(7)	156.63(8)	158.13(7)
$[(L7)Cu]^{2-}$	77.78(10)	81.30(10)	80.62(10)	118.57(10)	157.37(10)	157.09(10)

Electron Paramagnetic Resonance (EPR)

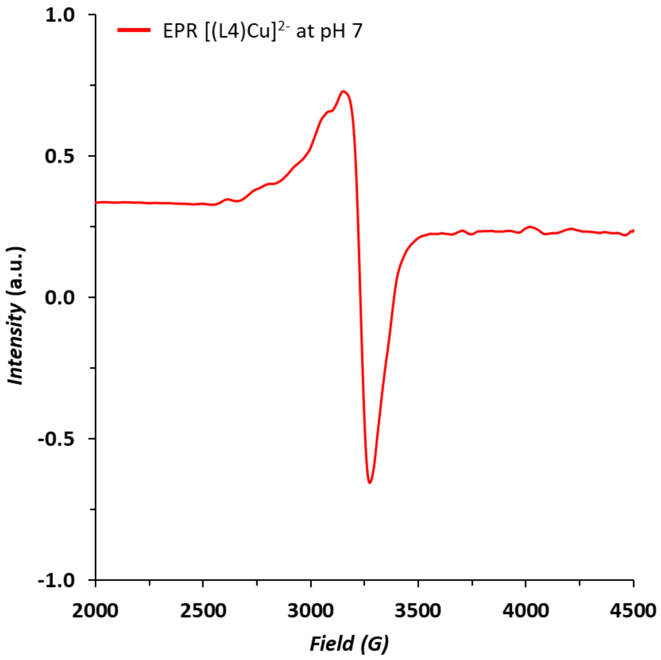

Figure 38. EPR spectra measured for **[(L4)Cu]$^{2-}$** in 0.1 M phosphate buffer (pH 7). The spectrum shows the characteristic EPR signal for a Cu^{II} complex with g_{II} = 2.074.

Electrochemical behavior in organic solvents

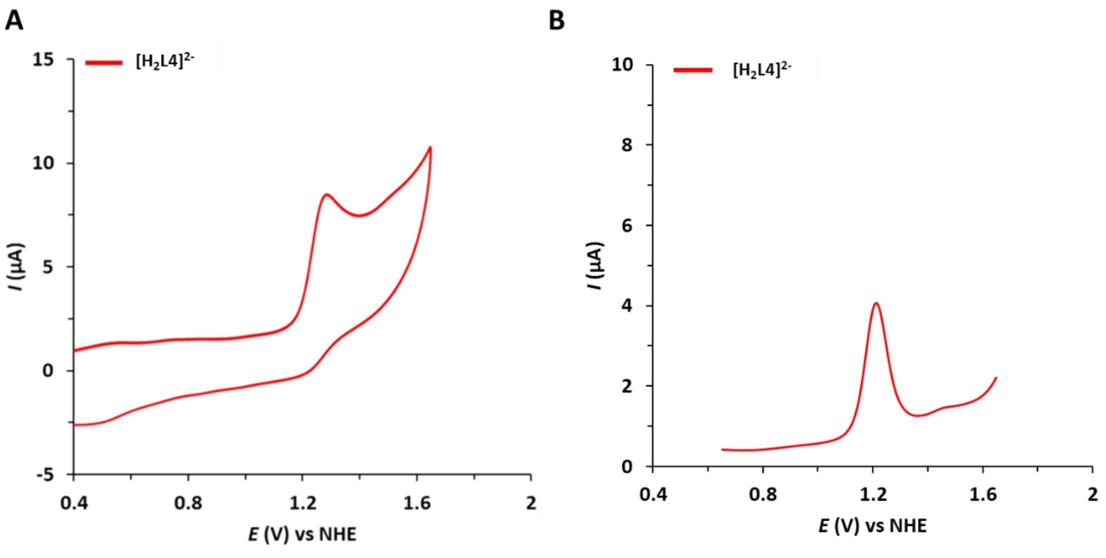

Figure S39. (**A**) Cyclic Voltammogram of a 1 mM solution of H_2L4^{2-} in DMF containing 0.1 M $TBAPF_6$ at a scan rate of 100 mV/s (**B**) DPV experiments of a 1 mM solution of H_2L4^{2-} in DMF containing 0.1 M $TBAPF_6$.

Electrochemical behavior in water

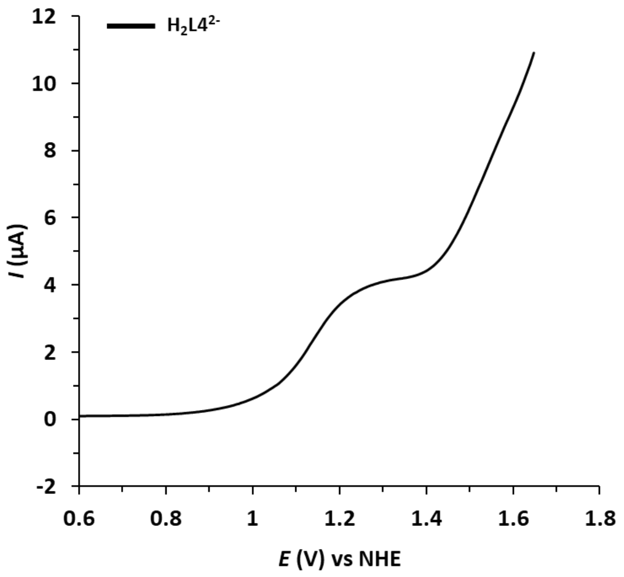

Figure S40. DPV experiment of a 1 mM solution of $[H_2L4]^{2-}$ in 0.1 M phosphate buffer pH 11.6. Conditions: scan rate of 100 mV/s, GC as working electrode.

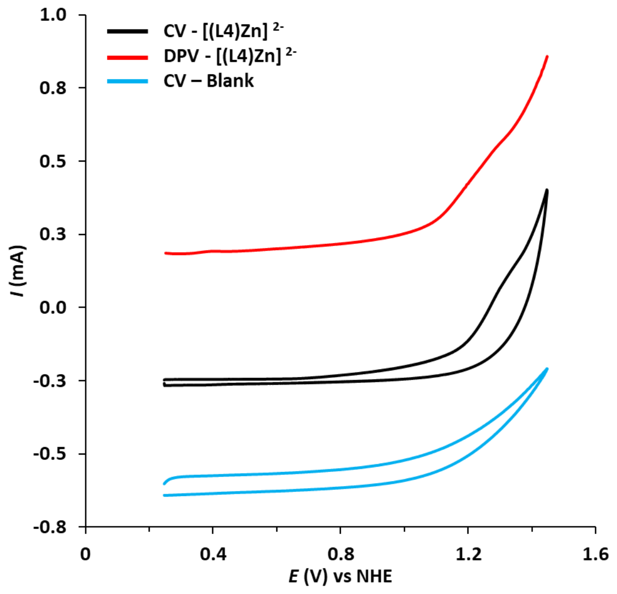

Figure S41. CV (black line) and DPV (red line) experiments of 1 mM solution of $\mathbf{[(L4)Zn]^{2-}}$ in a 0.1 M phosphate buffer pH 11.6. Blue line corresponds to a blank with no complex. Conditions: scan rate of 100 mV/s, GC-paper as working electrode.

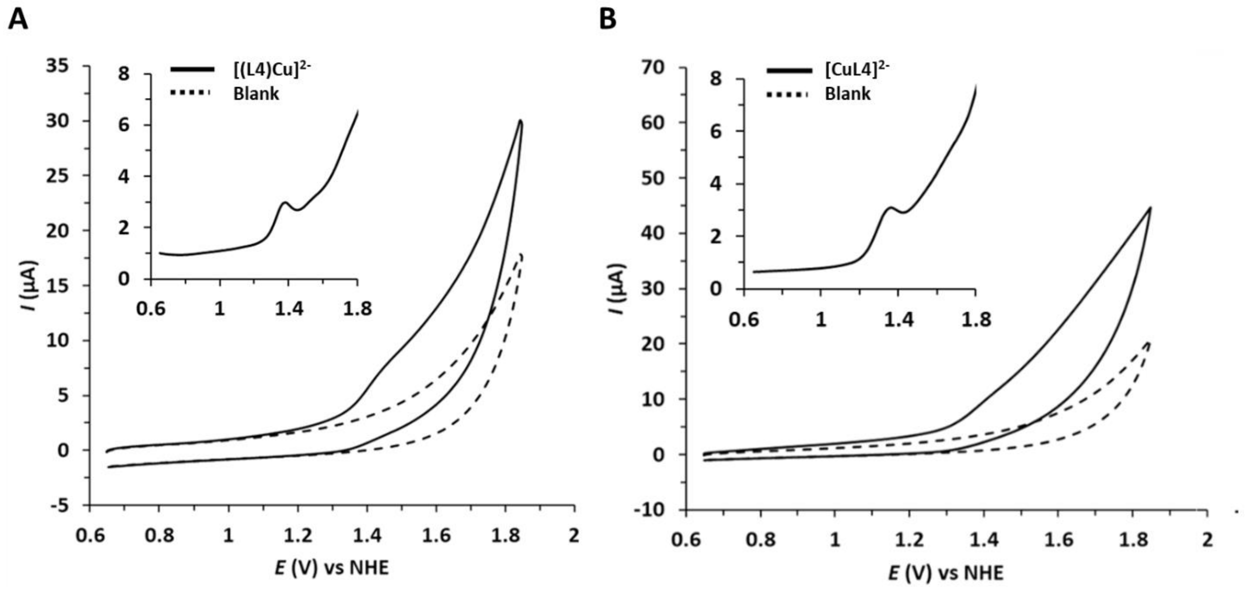

Figure S42. Cyclic Voltammograms of a 1 mM solution of **[(L4)Cu]$^{2-}$** at pH 7 (**A**) and 11.6 (**B**) in 0.1 M phosphate buffer solutions. *Inset:* Differential Pulse Voltammograms for **[(L4)Cu]$^{2-}$** at each pH value. Dashed black line corresponds to a blank with no catalyst. Conditions: scan rate of 100 mV/s, GC as working electrode.

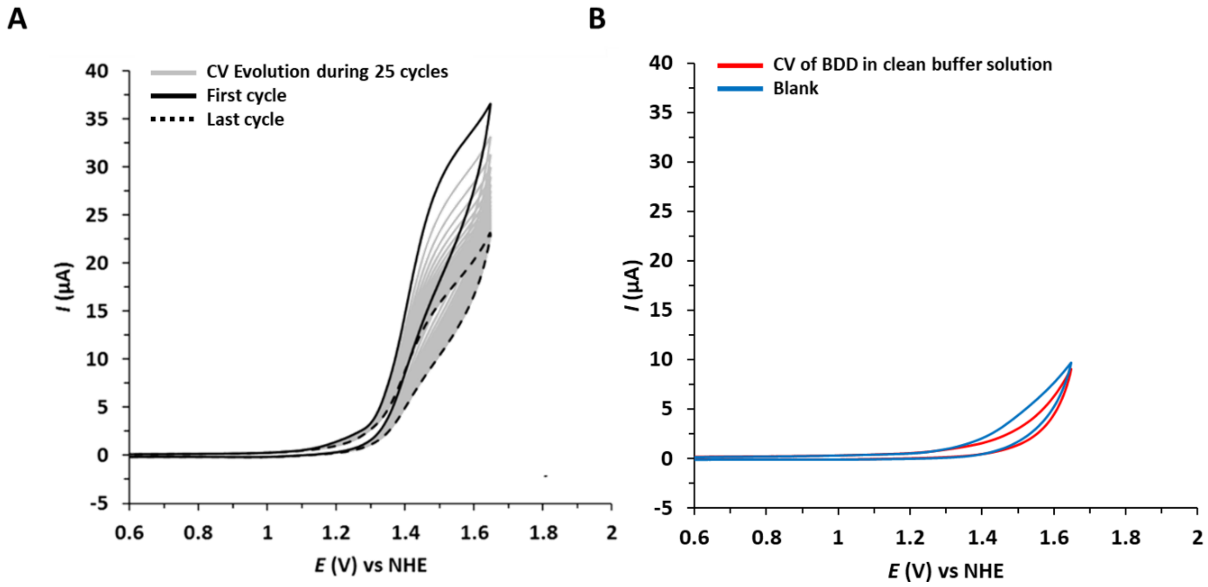

Figure S43. (**A**) Evolution of CV profile of a 1 mM solution of **[(L4)Cu]$^{2-}$** in 0.1 M phosphate buffer (pH 11.6) during 25 cycles. (**B**) CV measurement with a BDD electrode that has performed the previous 25 cycles of the complex (red line) or of a blank solution (blue line) immersed in a freshly-prepared 0.1 M phosphate buffer pH 11.6. Conditions: scan rate of 100 mV/s, BDD disk as working electrode.

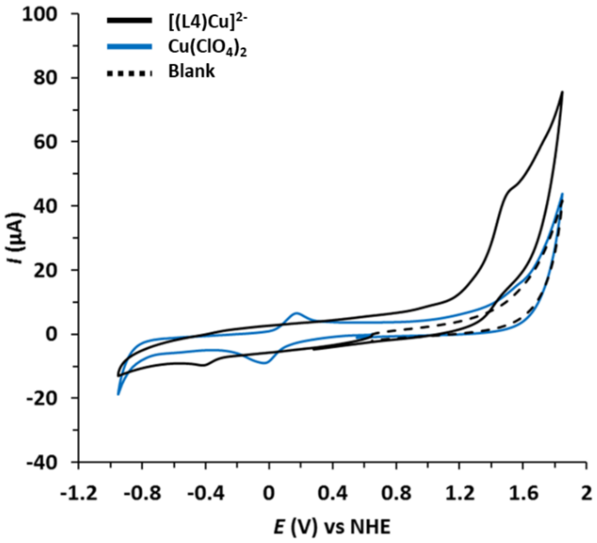

Figure S44. Cyclic Voltammograms of a 1 mM solution of **[(L4)Cu]$^{2-}$** in 0.1 M phosphate buffer pH 11.6, showing the electrocatalytic response of the complex (solid black line), of a 1 mM solution of $Cu(ClO_4)_2$ (solid blue line) and the blank (dashed line). Conditions: scan rate of 100 mV/s, BDD disk as working electrode.

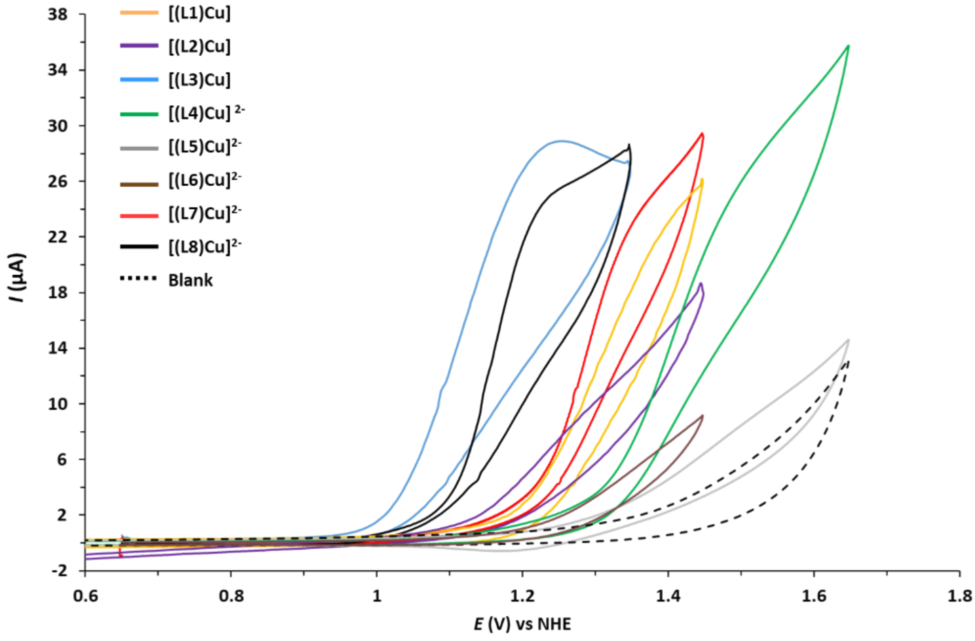

Figure S45. Cyclic Voltammograms for the complexes studied in this work in 0.1 M phosphate buffer pH 11.6. *Note:* In case of complexes **[(L1-3)Cu]** the experiments were performed in a mixture of 0.1 M phosphate buffer/TFE (6:4) to fully solubilize the complexes. [**Complex**] = ~1 mM. BDD disk as working electrode.

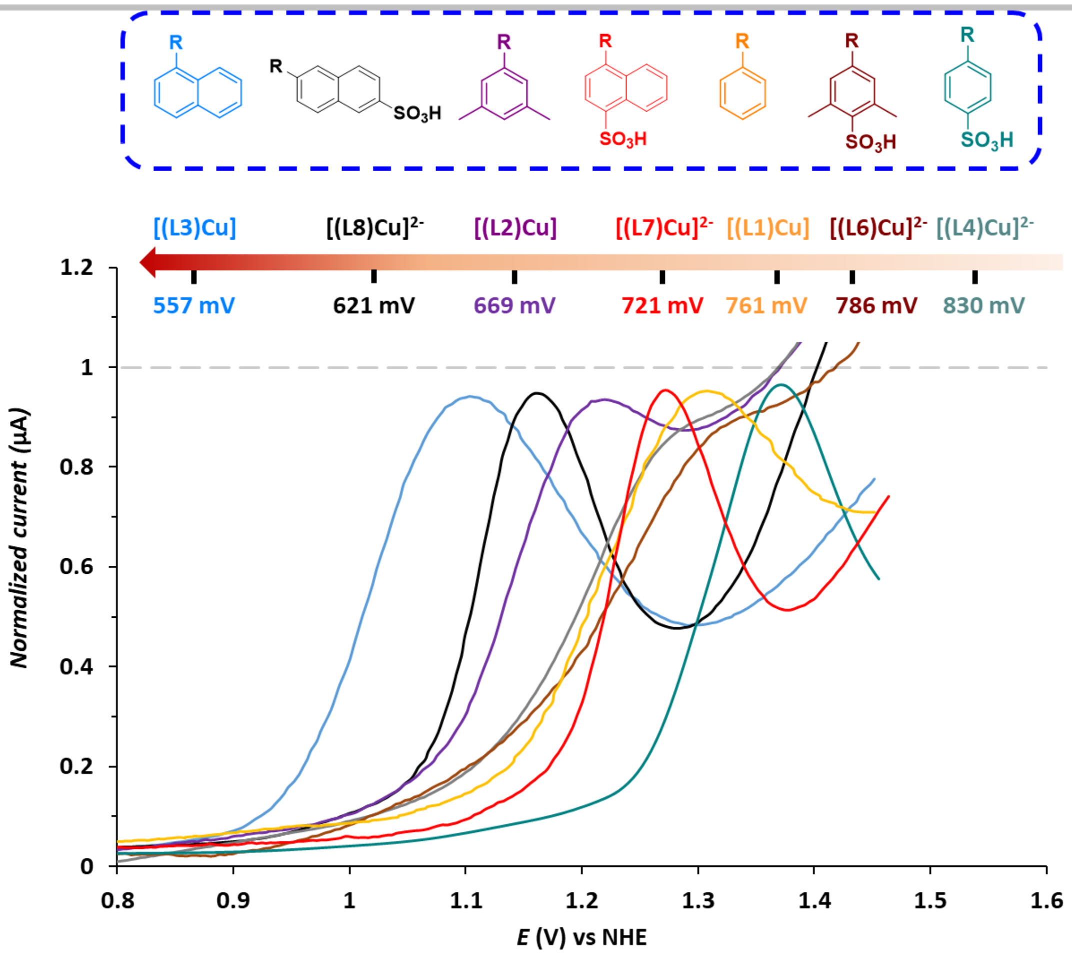

Figure S46. DPV experiments for the complexes studied in this work in 0.1 M phosphate buffer pH 11.6. The current (µA) has been normalized between 0 and 1 values for comparison purposes. *Note:* In case of complexes **[(L1-3)Cu]** the experiments were performed in a mixture of 0.1 M phosphate buffer/TFE (6:4) to fully solubilize the complexes. [**Complex**] = ~1 mM. BDD disk as working electrode. *Top inset:* Influence of the substituents in $[(LN)Cu]^{n-}$ complexes on the η (mV) of the water oxidation reaction.

A

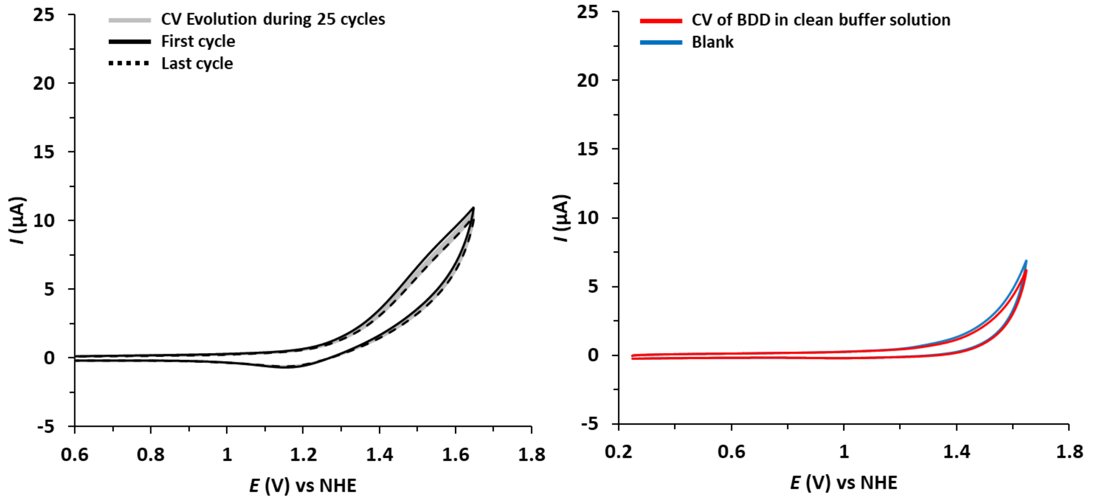
CV Evolution during 25 cycles
First cycle
Last cycle
I (µA)
E (V) vs NHE
CV of BDD in clean buffer solution
Blank
I (µA)
E (V) vs NHE

B

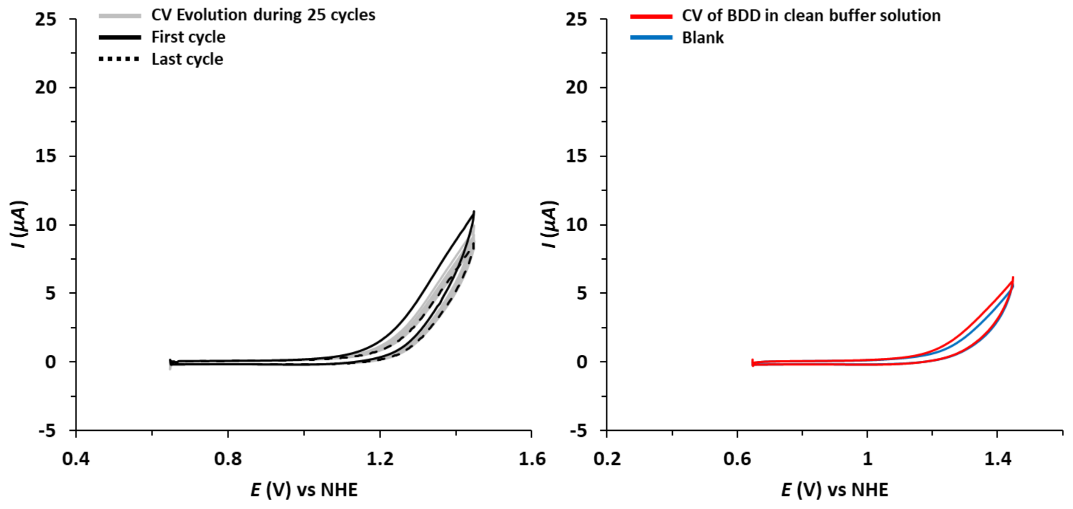
CV Evolution during 25 cycles
First cycle
Last cycle
I (µA)
E (V) vs NHE
CV of BDD in clean buffer solution
Blank
I (µA)
E (V) vs NHE

C

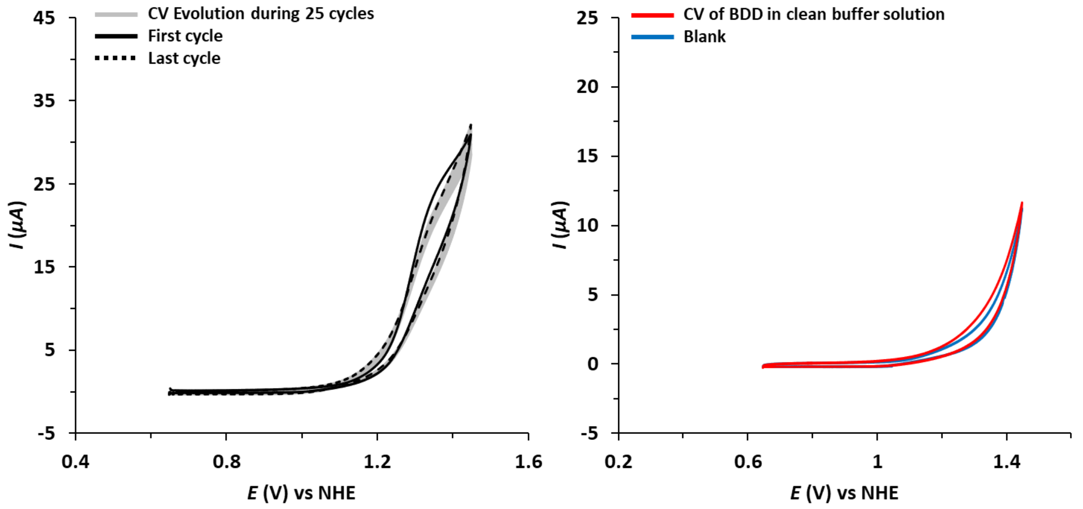
CV Evolution during 25 cycles
First cycle
Last cycle
I (µA)
E (V) vs NHE
CV of BDD in clean buffer solution
Blank
I (µA)
E (V) vs NHE

D

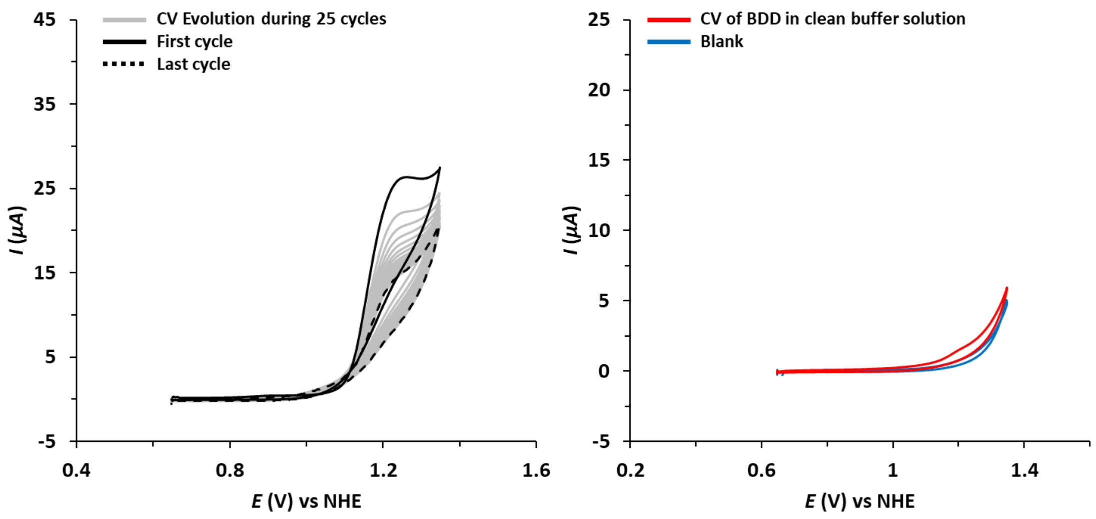

Figure S47. Evolution of CV profile for the complexes in 0.1 M phosphate buffer pH 11.6 after 25 cycles (Left) and CV measurement with a BDD electrode that has performed the previous 25 cycles of the complex (red line) or of a blank solution (blue line) immersed in a freshly-prepared catalyst-free 0.1 M phosphate buffer pH 11.6 (Right). Code: (**A**) **$[(L5)Cu]^{2-}$**,(**B**) **$[(L6)Cu]^{2-}$**,(**C**) **$[(L7)Cu]^{2-}$** and (**D**) **$[(L8)Cu]^{2-}$**. Conditions: scan rate of 100 mV/s, [**Complex**] = 1 mM. BDD as working electrode.

Determination of the kinetic constant. Foot of the Wave Analysis (FOWA).

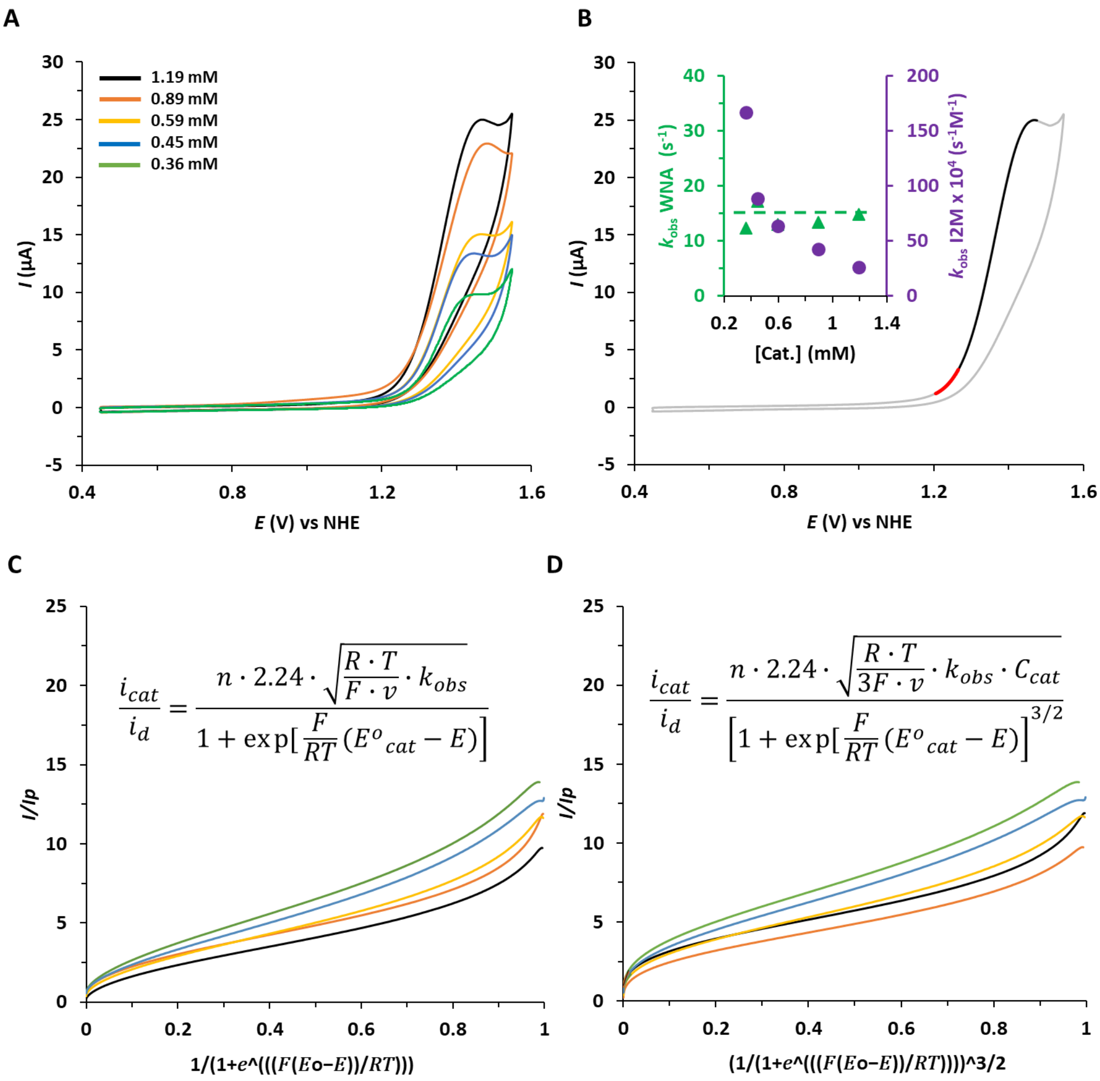

Figure S48. (**A**) CVs of [**(L4)Cu**]$^{2-}$ at different concentrations in 0.1 M phosphate buffer pH 11.6 at 100 mV/s scan rate. (**B**) CV of a 1.19 mM solution of [**(L4)Cu**]$^{2-}$ in 0.1 M phosphate buffer pH 11.6 at 100 mV/s scan rate (grey line), experimental data used for FOWA analysis (black line) and region used for the determination of k_{obs} (red line) by plotting i/i_p^0 vs. 1/{1+exp[(F/RT)(E^0_{PQ}−E)]} for a WNA mechanism and i/i_p^0 vs. (1/{1+exp[(F/RT)(E^0_{PQ}−E)]})$^{3/2}$ for an I2M mechanism. *Inset:* Plot of k_{obs} vs. [**(L4)Cu**]$^{2-}$ assuming a WNA mechanism (green trace) or an I2M mechanism (purple trace). The linear trend indicates that the electrochemical oxidation of water to dioxygen is following a WNA mechanism.[3] (**C**) FOWA region obtained by plotting i/i_p^0 vs. 1/{1+exp[(F/RT)(E^0_{PQ}−E)]} for a WNA mechanism and (**D**) FOWA region obtained by plotting i/i_p^0 vs. (1/{1+exp[(F/RT)(E^0_{PQ}−E)]})$^{3/2}$ for an I2M mechanism used for the calculation of k_{obs}.

A

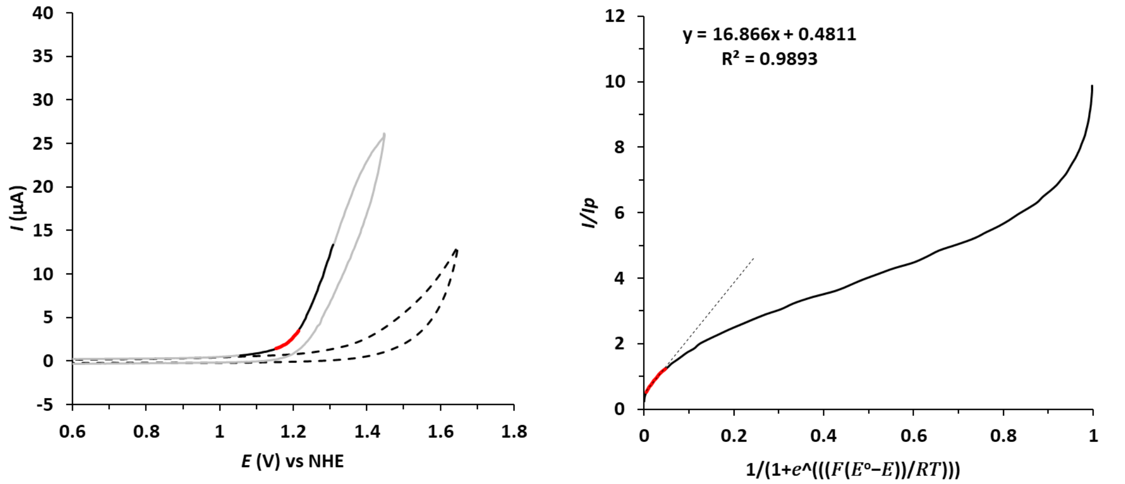
I (µA)
E (V) vs NHE
y = 16.866x + 0.4811
R² = 0.9893
I/Ip
1/(1+e^(((F(E°−E))/RT)))

B

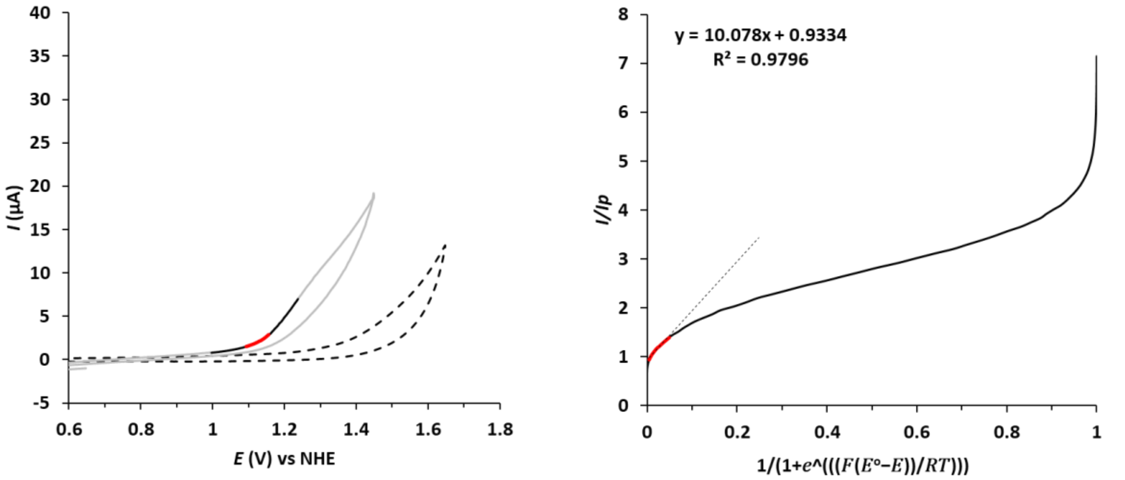
I (µA)
E (V) vs NHE
y = 10.078x + 0.9334
R² = 0.9796
I/Ip
1/(1+e^(((F(E°−E))/RT)))

C

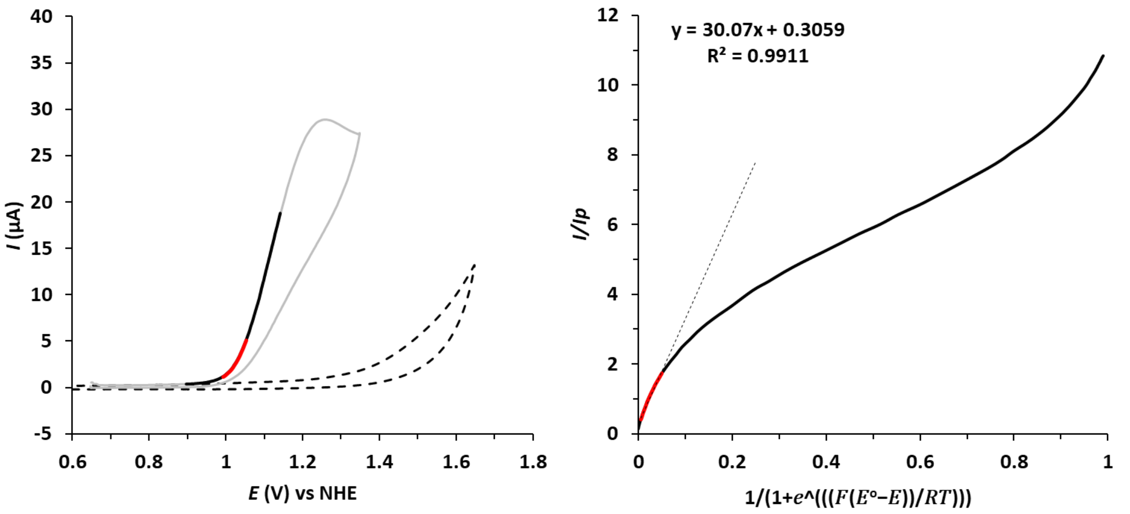
I (µA)
E (V) vs NHE
y = 30.07x + 0.3059
R² = 0.9911
I/Ip
1/(1+e^(((F(E°−E))/RT)))

D

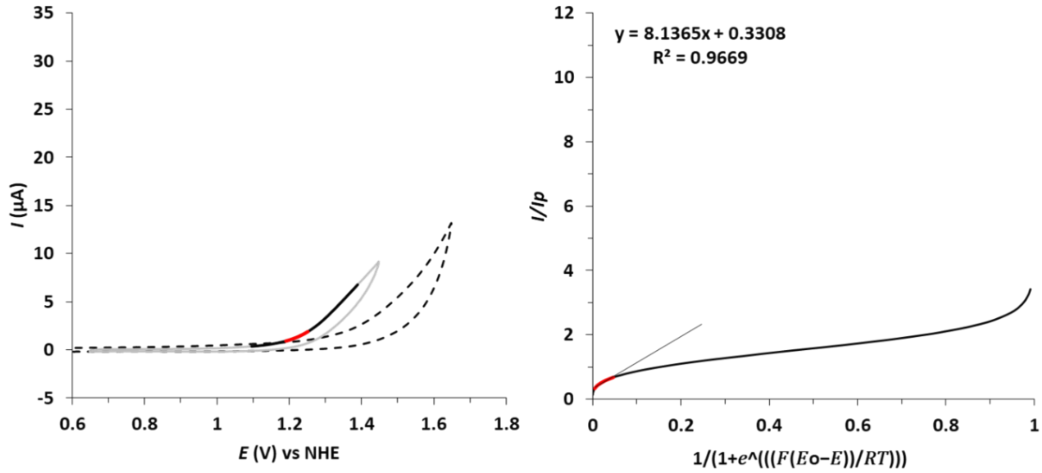

E

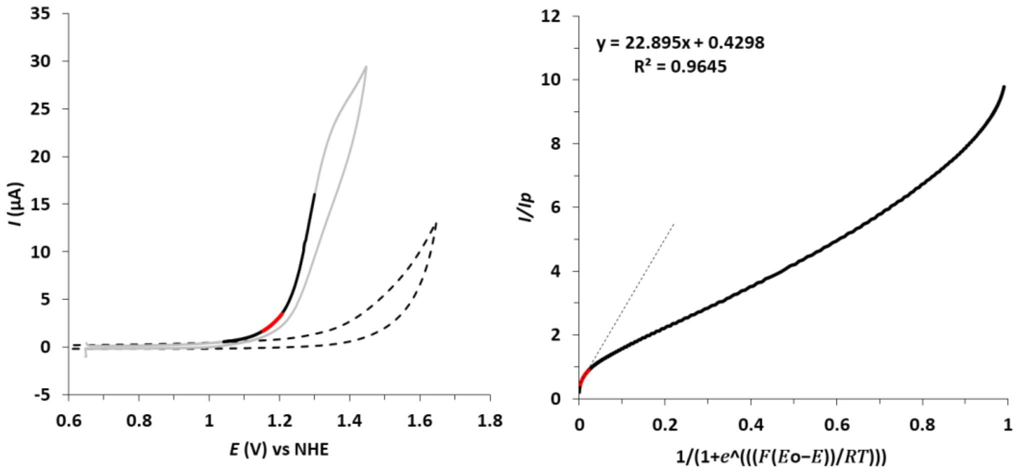

F

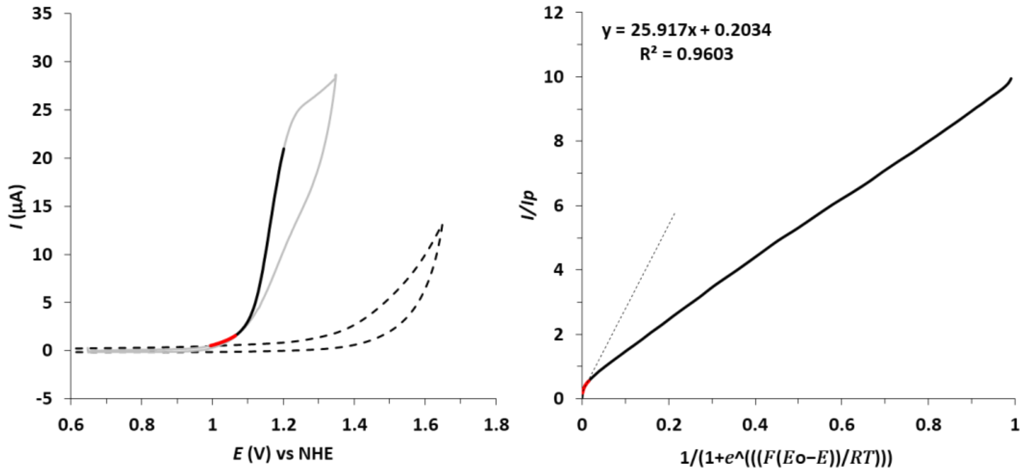

Figure S49. (Left) CVs of 1 mM solution of complexes in 0.1 M phosphate buffer pH 11.6 at 100 mV/s scan rate (grey line) and the blank (dashed line). Code: (**A**) **[(L1)Cu]**, (**B**) **[(L2)Cu]**,(**C**) **[(L3)Cu]**, (**D**) **[(L6)Cu]$^{2-}$**, (**E**) **[(L7)Cu]$^{2-}$**, (**F**) **[(L8)Cu]$^{2-}$**. Solid red line corresponds to the experimental data used for FOWA analysis and solid black line shows the region used for the determination of k_{obs}. (Right) FOWA obtained by plotting i/i_p^o vs. 1/{1+exp[(F/RT)($E^o_{PQ}-E$)]}. *Note:* In case of complexes **[(L1-3)Cu]** the experiments were performed in a mixture of 0.1 M phosphate buffer/TFE (6:4) to fully solubilize the complexes.

O_2 Evolution experiments.

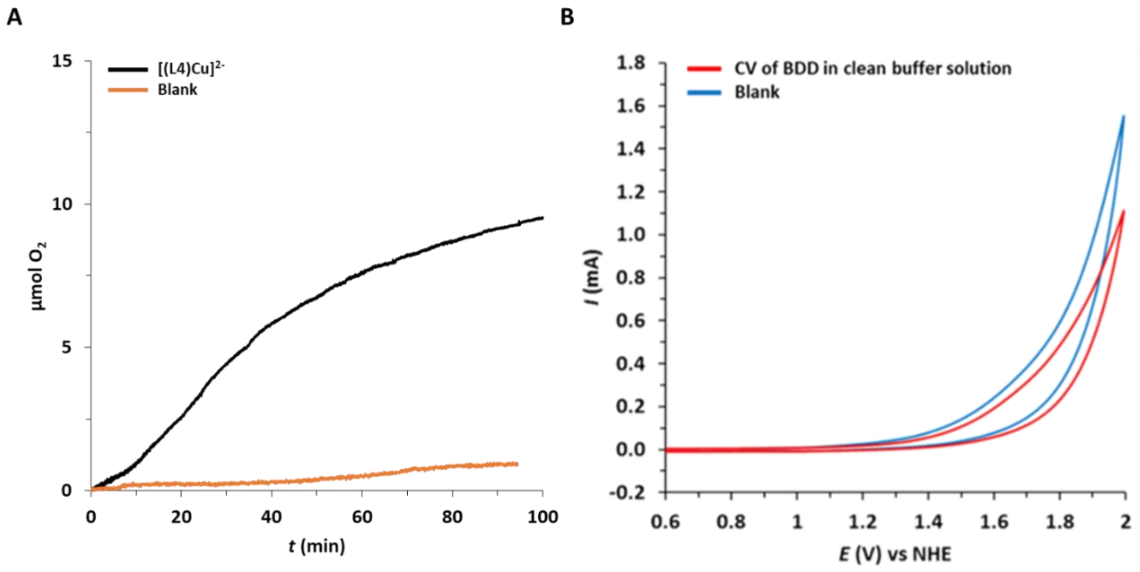

Figure S50. (**A**) Oxygen evolution measurements given in μmol O_2 vs. time (min) for complex **$[(L4)Cu]^{2-}$** at 1.5 mM concentration (V_{total}= 3 mL) using a Clark probe electrode during a CPE at 1.6 V vs. NHE in 0.1 M phosphate buffer pH 11.6. Blank data in the absence of complex is shown in orange. (**B**) Comparison of CVs of a blank solution after a CPE at 1.6 V during 95 mins and of the mechanically polished BDD electrode under a blank solution, showing no catalytic response due to the presence of heterogeneous materials deposited onto the surface of the electrode. Conditions: scan rate of 100 mV/s, BDD disk as working electrode, Pt mesh counter electrode and AgCl as reference electrode. Q = 4.58 C, moles e^-= 4.75 x 10^{-5} mol, FE= 76 %. TON= (μmol O_2)/(μmol cat.)= 1.86. A TON of 58993 was obtained using the methodology developed by Savéant and co-workers based on the electroactive catalyst (i.e. catalyst in contact with the electrode).[4]

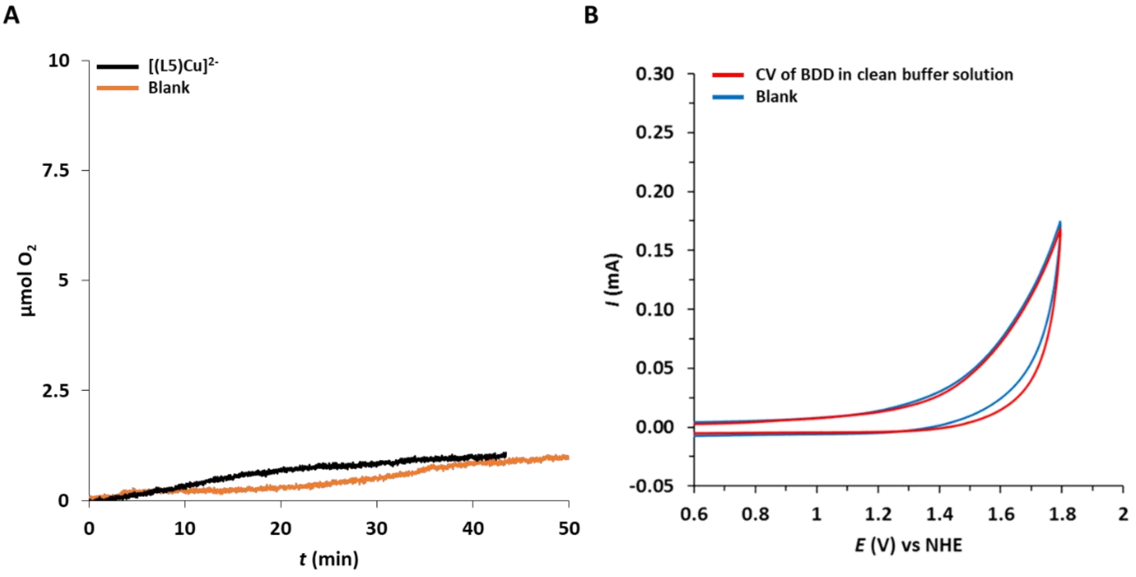

Figure S51. (**A**) Oxygen evolution measurements given in μmol O_2 vs. time (min) for complex **$[(L5)Cu]^{2-}$** at 1.5 mM concentration (V_{total}= 3 mL) using a Clark probe electrode during a CPE at 1.6 V vs. NHE in 0.1 M phosphate buffer pH 11.6. Blank data in the absence of complex is shown in orange. (**B**) Comparison of CVs of a blank solution after a CPE at 1.6 V during 43 min and of the mechanically polished BDD electrode under a blank solution, showing no catalytic response due to the presence of heterogeneous materials deposited onto the surface of the electrode. Conditions: scan rate of 100 mV/s, BDD disk as working electrode, Pt mesh counter electrode and AgCl as reference electrode.

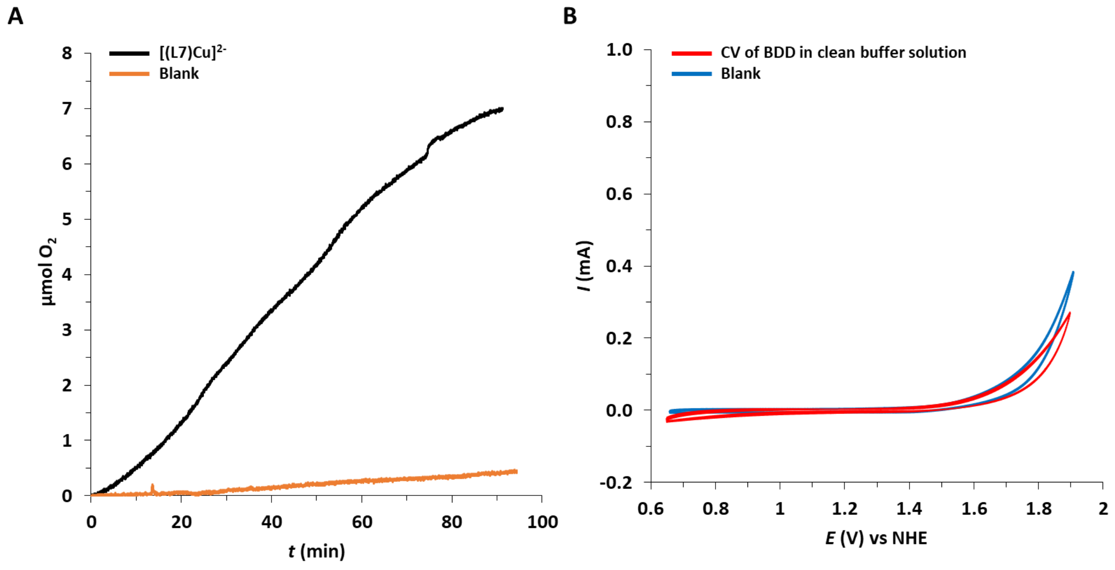

Figure S52. (**A**) Oxygen evolution measurements given in µmol O_2 vs. time (min) for complex **[(L7)Cu]$^{2-}$** at 1.5 mM concentration (V_{total}= 3 mL) using a Clark probe electrode during a CPE at 1.45 V vs. NHE in 0.1 M phosphate buffer pH 11.6 V. Blank data in the absence of complex is shown in orange. (**B**) Comparison of CVs of a blank solution after a CPE at 1.45 V during 94 min and of the mechanically polished BDD electrode under a blank solution. Conditions: scan rate of 100 mV/s, BDD disk as working electrode, Pt mesh counter electrode and AgCl as reference electrode. Q = 4.98 C, moles e^-= 5.16 x 10^{-5} mol, FE= 52 %. TON= (µmol O_2)/(µmol cat.)= 1.35. TON of 130409 was obtained using the methodology developed by Savéant and coworkers based on the electroactive catalyst (i.e. catalyst in contact with the electrode).[4]

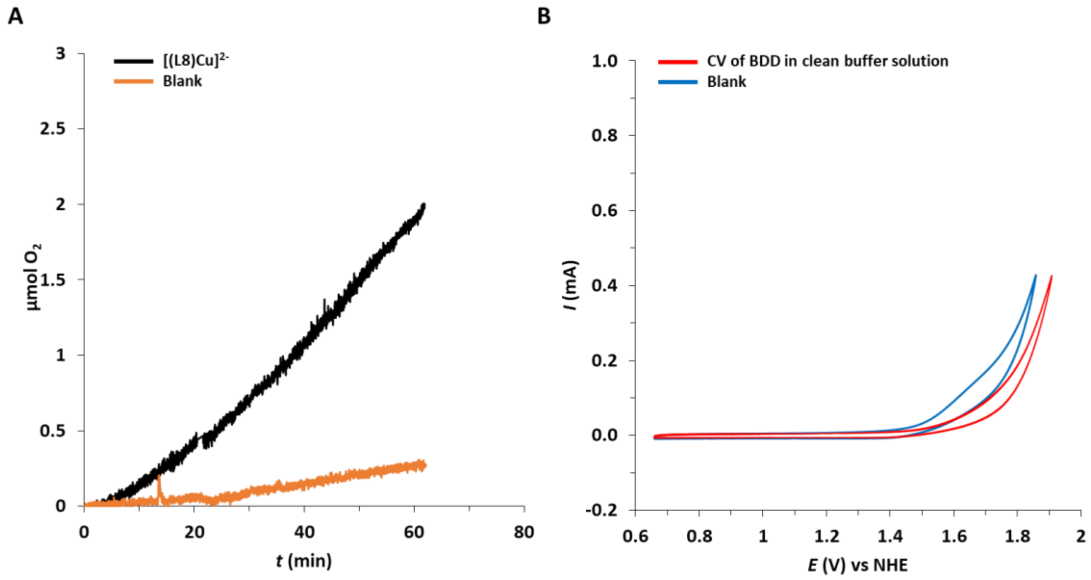

Figure S53. (**A**) Oxygen evolution measurements given in µmol O_2 vs. time (min) for complex **[(L8)Cu]$^{2-}$** at 1.5 mM concentration (V_{total}= 3 mL) using a Clark probe electrode During a CPE at 1.45 V vs. NHE in 0.1 M phosphate buffer pH 11.6. Blank data in the absence of complex is shown in orange. (**B**) Comparison of CVs of a blank solution after a CPE at 1.45 V during 95 min and of the mechanically polished BDD electrode under a blank solution. Conditions: scan rate of 100 mV/s, BDD disk as working electrode, Pt mesh counter electrode and AgCl as reference electrode. Q= 1.84 C, moles e^-= 1.9 x 10^{-5} mol, FE= 40%. TON= (µmol O_2)/(µmol cat.)= 0.4. A TON of 97964 was obtained using the methodology developed by Savéant and coworkers based on the electroactive catalyst (i.e. catalyst in contact with the electrode).[4]

Scanning Electron Microscopy

A

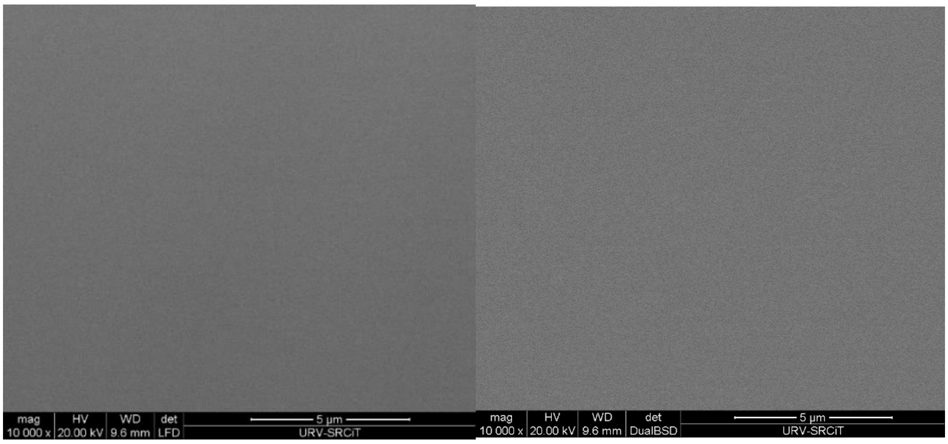

B

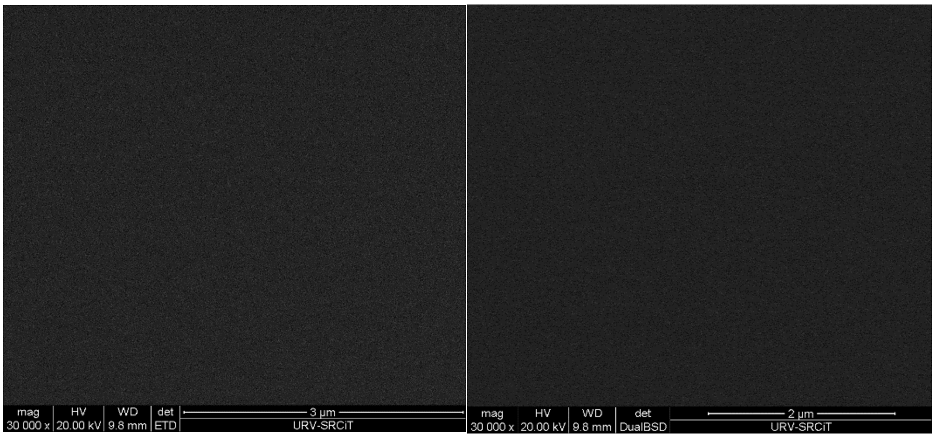

C

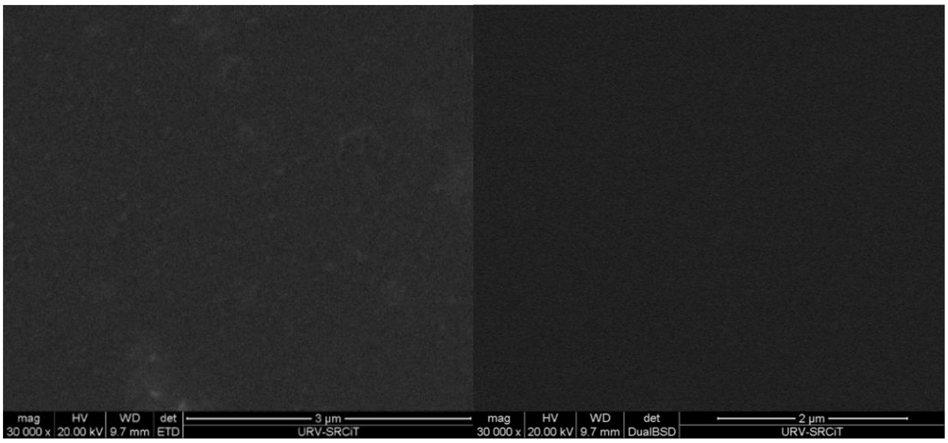

Figure S54. SEM micrographs (left) and corresponding back-scattered electron micrographs (right) of a glassy carbon plate after a 30 minute electrolysis of a 1.5 mM solution of the complexes in phosphate buffer pH 11.6 at 1.6 V vs. NHE. Code: (**A**) **[(L4)Cu]$^{2-}$**,(**B**) **[(L7)Cu]$^{2-}$**,(**C**) **[(L8)Cu]$^{2-}$**.There is no appreciable presence of copper oxide nanoparticles or deposited materials on the electrode.

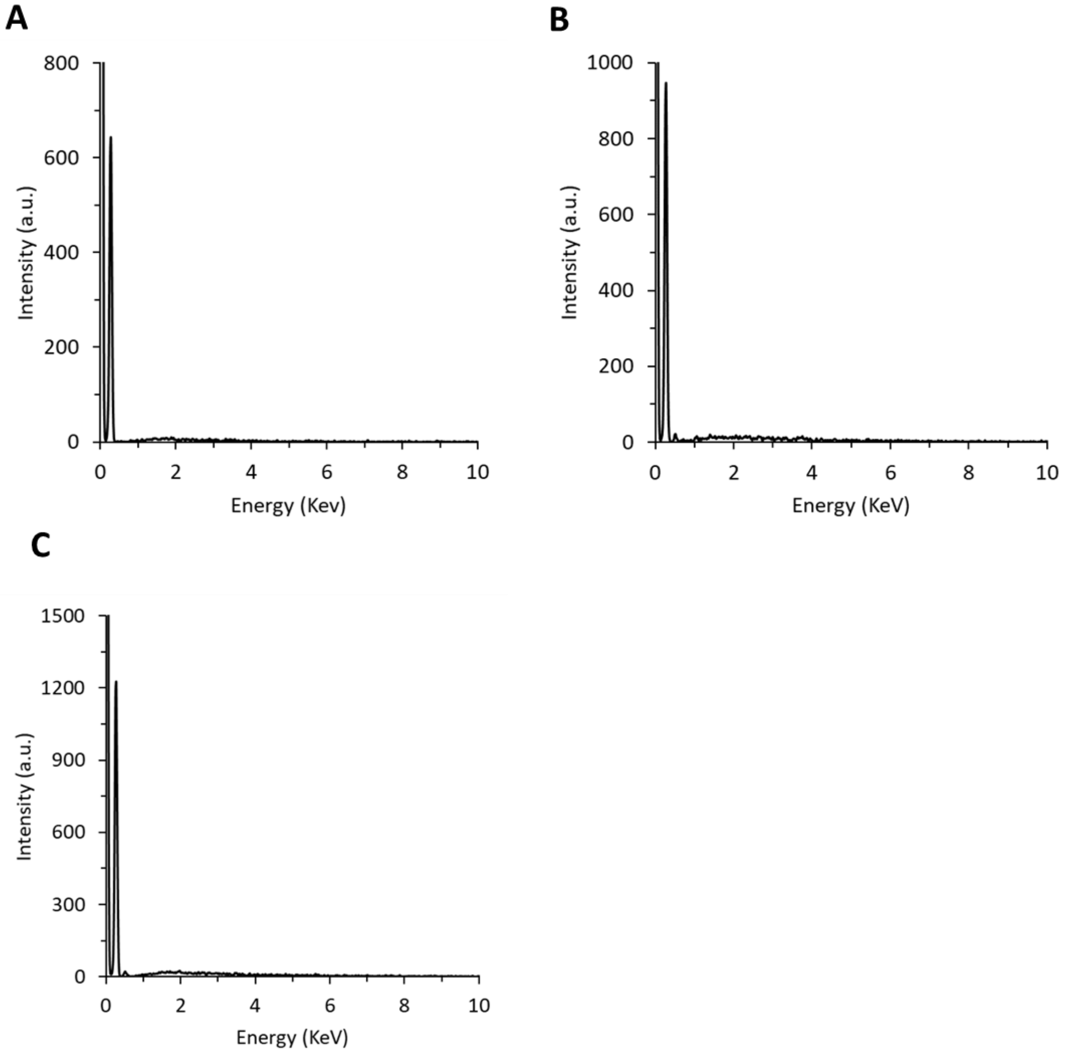

Figure S55. EDX spectra of the glassy carbon electrodes after a 30 minute electrolysis in phosphate buffer pH 11.6 at 1.6 V vs. NHE. Code: (**A**) **[(L4)Cu]**$^{2-}$, (**B**) **[(L7)Cu]**$^{2-}$, (**C**) **[(L8)Cu]**$^{2-}$.There is no appreciable presence of copper on the surface of the electrode (Cu expected peaks, Kα= 8.04 KeV and Lα= 0.930 KeV).

Computational studies

Computational Details

All calculations were carried out with the Gaussian09 program package[13] using DFT methodology. We used B3LYP as the functional, including D3 empirical dispersion correction developed by Grimme (B3LYP-D3).[14,1514,15] The basis set was split into 6-31+G(d) for C, N, S, O and H[16,17,18] and LANL2TZ(f) for Cu.[19,20,21,22,23] Implicit solvation was introduced through the SMD model,[24] with water as the solvent. All geometry optimizations were computed in solution without symmetry restrictions. The nature of all computed stationary points as minima or transition states was confirmed through vibrational frequency calculations. Free energy corrections were calculated at 298.15 K and 105 Pa pressure, including zero point energy corrections (ZPE). In addition, a correction term of 1.89 kcal/mol (at 298 K) was added when necessary to account for the standard state concentration of 1 M, except for water, whose concentration was considered to be 55.6 M and its correction term 4.3 kcal/mol. Unless otherwise mentioned, all reported energy values are free energies in solution. In addition, stability of the wave function was checked for the calculations (stable option in G16).

The reaction energy barriers of the Minimum Energy Crossing Points (MECP) were estimated from potential energy relaxed scan from the crossing point of the quartet and doublet potential energy surfaces, along the O-O internal reaction coordinate, when the transition states could be found (or do not exist), applying entropic corrections from the minima to compute an estimated free energy change.

In the transformation from free energies to electrochemical magnitudes the values of 4.28 V for the absolute potential of the standard hydrogen electrode[25] and -11.72 eV for the free energy of the proton in aqueous solution at pH=0 were taken from the literature.[26] The value for the free energy of the proton was translated to the experimental pH value by adding a correction term of -0.059*pH, following the same procedure described elsewhere.[27]

The functional for the DFT calculations was B3LYP-D3 based on the calibration carried out in a previous work on related systems, [6,28] where its performance was compared with that of M06, M06-D3, M06L, M06-2X, ωB97xD and B97D. In order to validate this DFT methodology, the calculated optimized structures were compared to the X-Ray ones. Table S4 summarizes all the main metrics for the coordination environment of the copper metal center. In addition, we have recalculated as single points all the species involved in Figure 3 using a larger basis set (6-311++G(3d,2p) for all the atoms except Cu/LANL2TZ(f) for Cu) and no significant differences were found (see Figure S65).

Table S4. Comparison of the main metrics for the X-Ray structure and the DFT optimized structure of complexes **$[(L4)Cu]^{2-}$** and **$[(L5)Cu]^{2-}$**.

COMPLEX	METRIC	X-RAY (SOLID)	DFT OPTIMIZED (WATER)
**$[(L4)Cu]^{2-}$**	$Cu-N_{bpy}$	1.95 Å, 1.95 Å	2.00 Å, 2.00 Å
	$Cu-N_{amide}$	2.00 Å, 2.00 Å	2.04 Å, 2.05 Å
	$N_{bpy}-Cu-N_{bpy}$	78.27°	77.55°
	$N_{amide}-Cu-N_{amide}$	119.69°	123.15°
**$[(L5)Cu]^{2-}$**	$Cu-N_{bpy}$	1.95 Å, 1.95 Å	1.99 Å, 1.99 Å
	$Cu-N_{amide}$	2.00 Å, 2.00 Å	2.02 Å, 2.03 Å
	$N_{bpy}-Cu-N_{bpy}$	78.49°	78.02°
	$N_{amide}-Cu-N_{amide}$	120.78°	123.01°

Table S5. Comparison of the experimental and calculated redox potential for the Cu(III)/Cu(II) couples in complexes **$[(L4)Cu]^{2-}$** and **$[(L5)Cu]^{2-}$**.

COMPLEX	$E_{1/2}^{O}$ (EXP)	$E_{1/2}^{O}$ (CALC)
**$[(L4)Cu]^{2-}$**	1.37 V	1.27 V
**$[(L5)Cu]^{2-}$**	1.27 V	1.14 V

Speciation for complex $[(L4)Cu]^{2-}$ in water

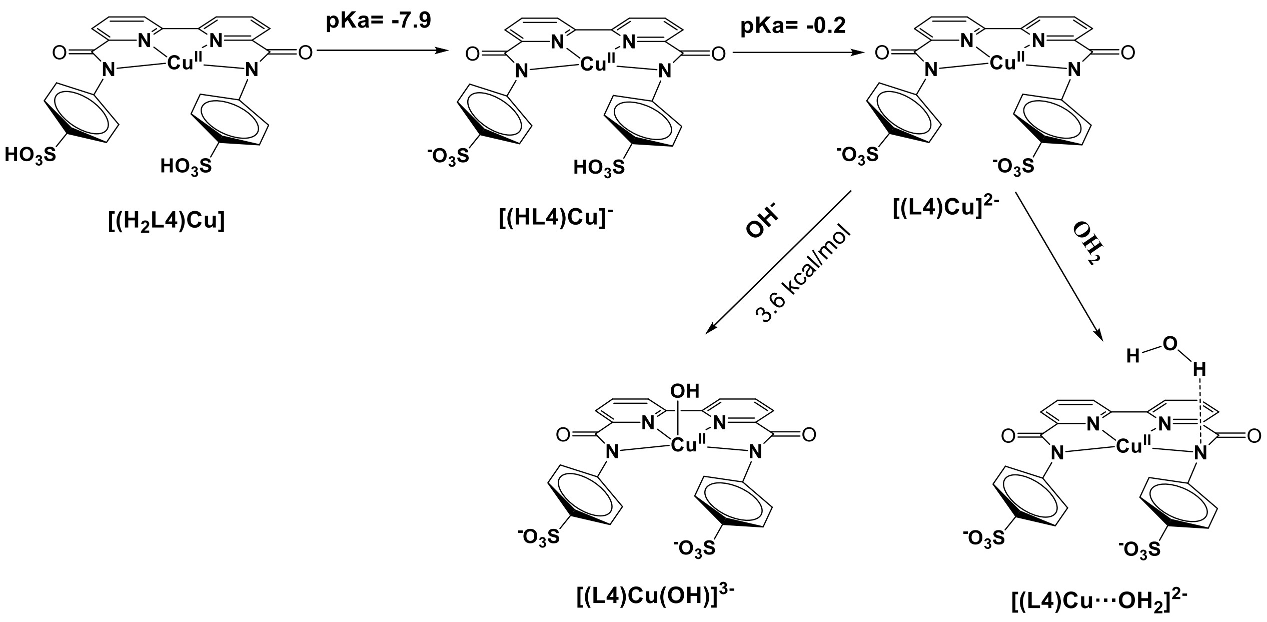

Figure S56. Deprotonation processes in complex **$[(L4)Cu]^{2-}$** and apical coordination of hydroxo or water molecules. The corresponding pK_a values or the free energy changes for each process are indicated above the arrows.

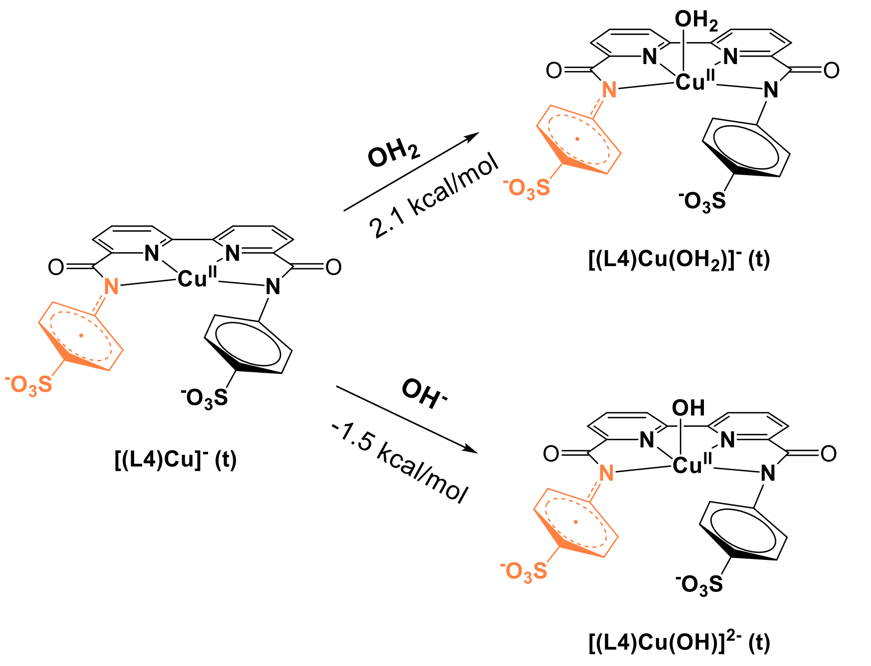

Figure S57. Apical coordination of hydroxo or water molecules in the one electron oxidized complex **$[(L4)Cu]^{2-}$**. The corresponding values of the free energy changes for each process are indicated above the arrows.

Electrochemical activation for complex [(L4)Cu]$^{2-}$

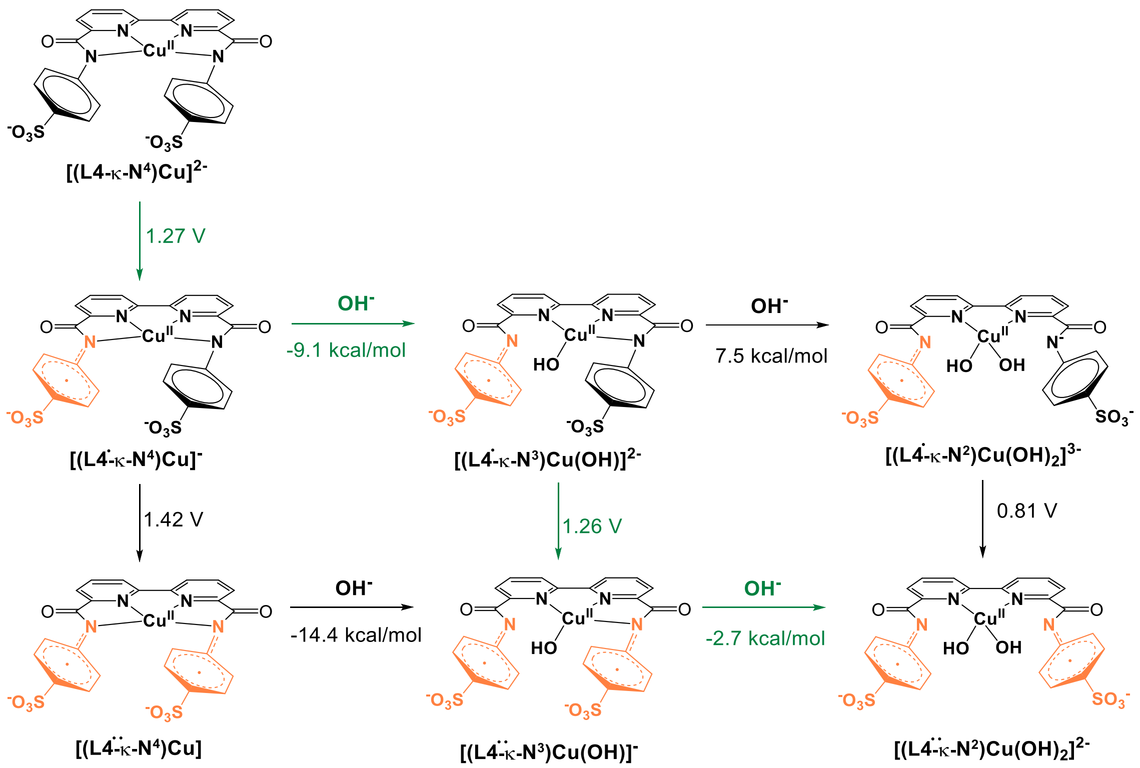

Figure S58. Possible pathways for the oxidation of catalyst **[(L4)Cu]$^{2-}$** to generate an active species for the O-O bond formation step.

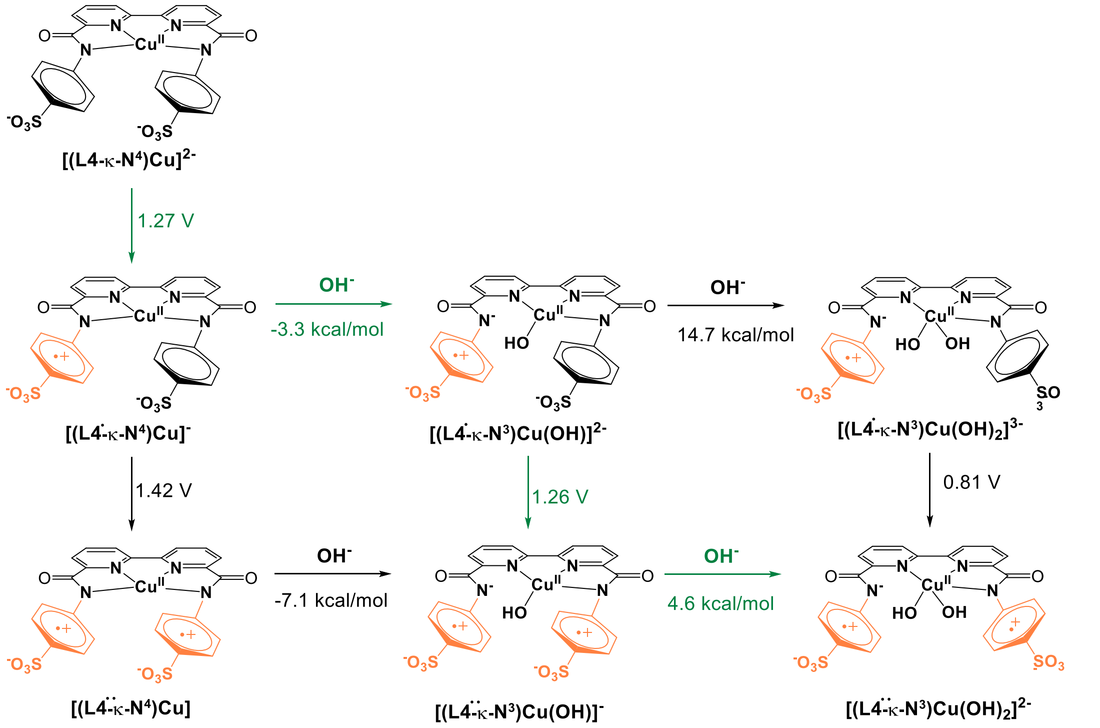

Figure S59. Possible pathways for the oxidation of catalyst **[(L4)Cu]$^{2-}$** to generate an active species for the O-O bond formation step using the experimental value for the energy of OH$^-$ solvation at standard state (-102.8 kcal·mol^{-1}).[29] This results suggest that the energy of solvation for OH$^-$ might be underestimated by SMD calculations, leading to less favorable coordination of the OH$^-$ to the Cu center. However, the calculated speciation in the different oxidation states is not substantially altered from that proposed using SMD, with the exception of formation of complex [(L4$^{\cdot\cdot}$-k-N^3)Cu(OH)$_2$]$^{2-}$ that is now slightly uphill but still accessible at room temperature towards formation of O_2. Therefore, the proposed reaction pathways are still supported by DFT but the calculated barrier for the O-O bond formation step might be overestimated when using the SMD model.

Potential energy relaxed scan for [(L4)Cu(OH)]⁻ and [(L4)Cu(OH)2]

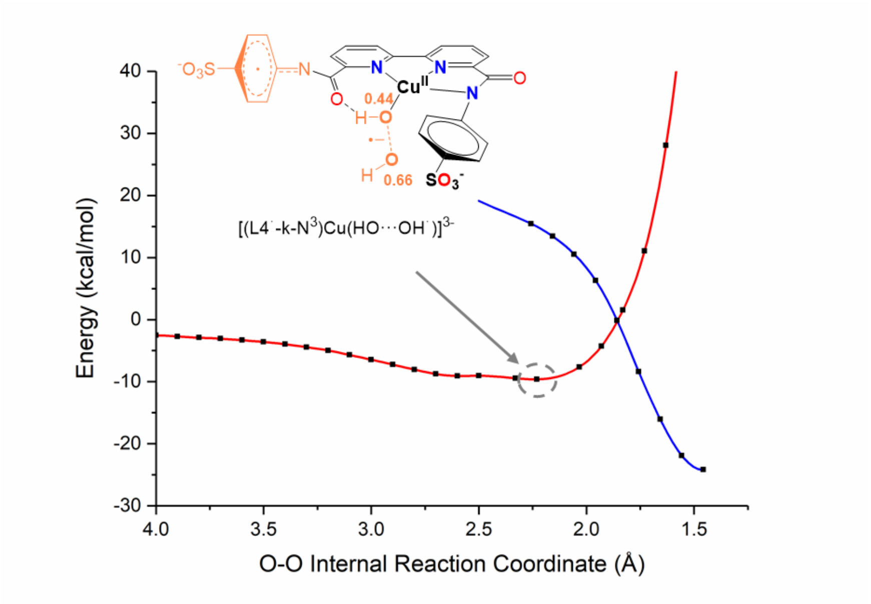

Figure S60. Potential energy relaxed scan for catalyst **[(L4)Cu]$^{2-}$** of the O-O reaction coordinate considering a monohydroxylation pathway. Red color represents the quartet state while blue color indicates the doublet state. The inset represents the optimized structure of the 2c-3e$^-$ intermediate.

.

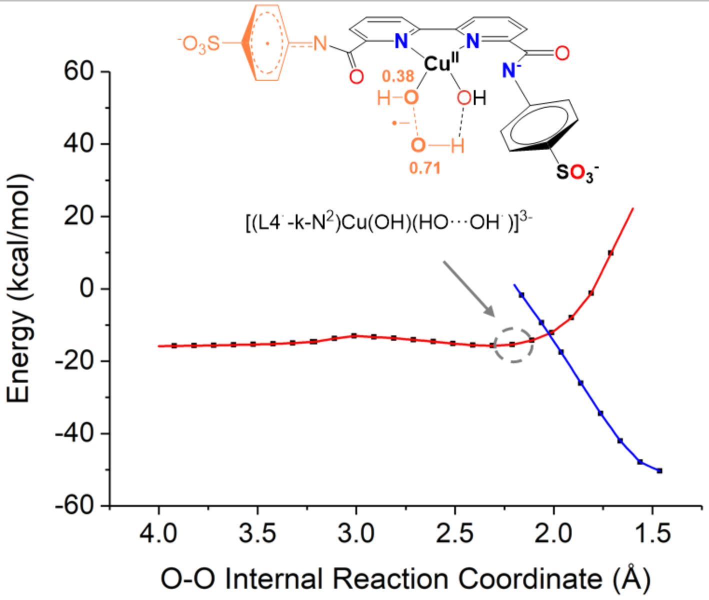

Figure S61. Potential energy relaxed scan for catalyst of **[(L4)Cu]$^{2-}$** of the O-O reaction coordinate considering a dihydroxylation pathway. Red color represents the quartet state while blue color indicates the doublet state. The inset represents the optimized structure of the 2c-3e$^-$ intermediate.

Structure and spin density distribution of 2c-3e intermediates

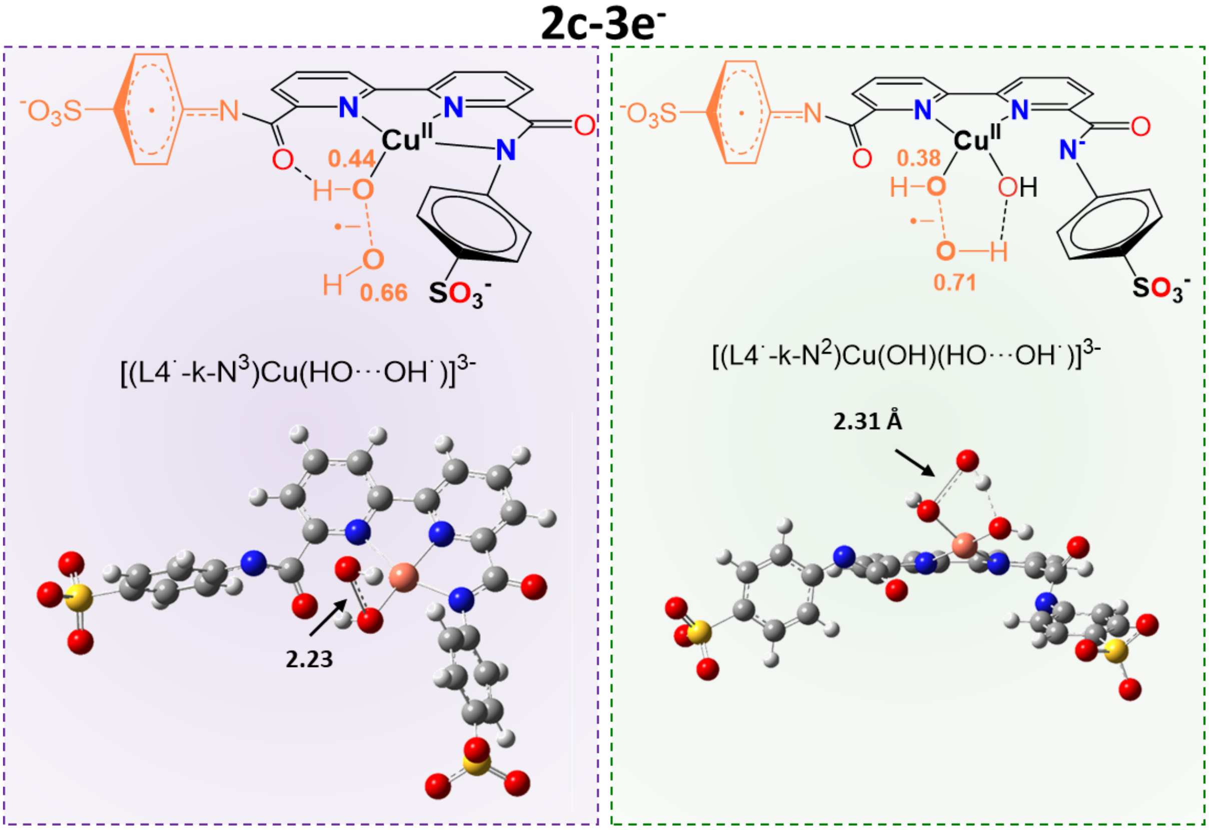

Figure S62. Schematic drawing with the spin distribution of each oxygen atom in orange (top) and optimized structure (bottom) of the 2c-3e intermediates for **[(L4˙-κ-N^3)Cu(OH)]$^{2-}$** (left) and **[(L4˙-κ-N^2)Cu(OH)$_2$]$^{3-}$** (right) catalysts.

Intramolecular relaxed scan for $[(L4\text{-}\kappa\text{-}N^2)Cu(OH)_2]^{2-}$

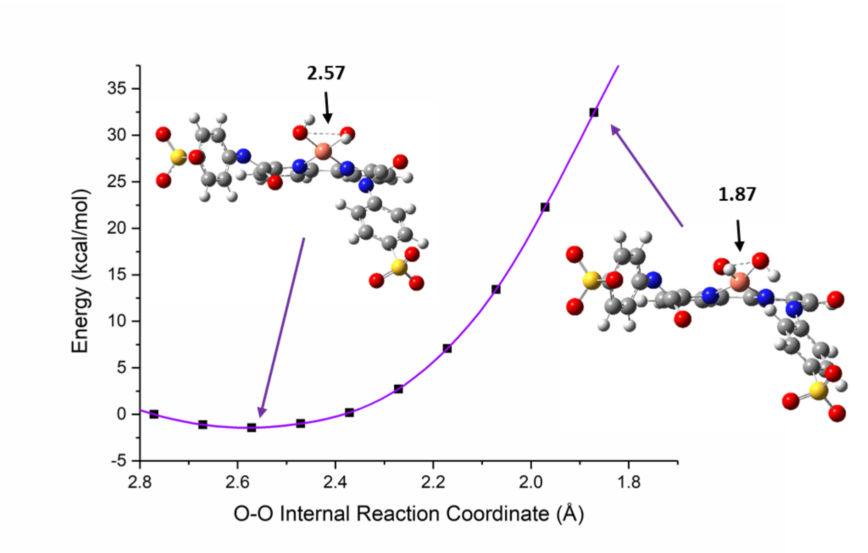

Figure S63. Potential energy relaxed scan of the intramolecular HO---OH coupling using catalyst $\mathbf{[(L4^{\cdot\cdot}\text{-}\kappa\text{-}N^2)Cu(OH)_2]^{2-}}$.

Structure of complex $[(L5)Cu]^{2-}$ and its oxidized species

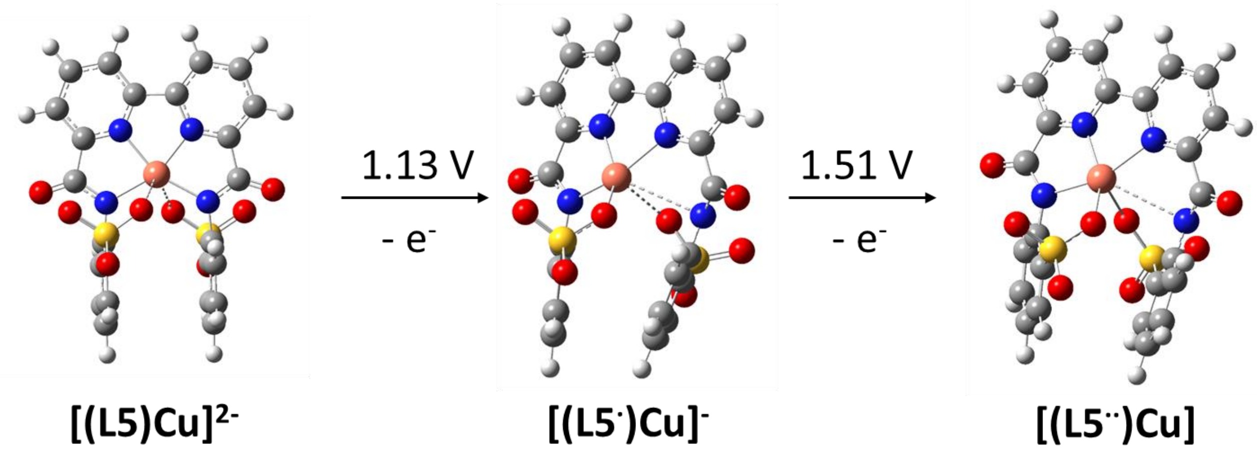

Figure S64. Optimized structures of complex $\mathbf{[(L5)Cu]^{2-}}$ and its oxidized species with the calculated redox potential above the arrows. All the structures feature coordination of the copper center to at least one of the sulfonate groups.

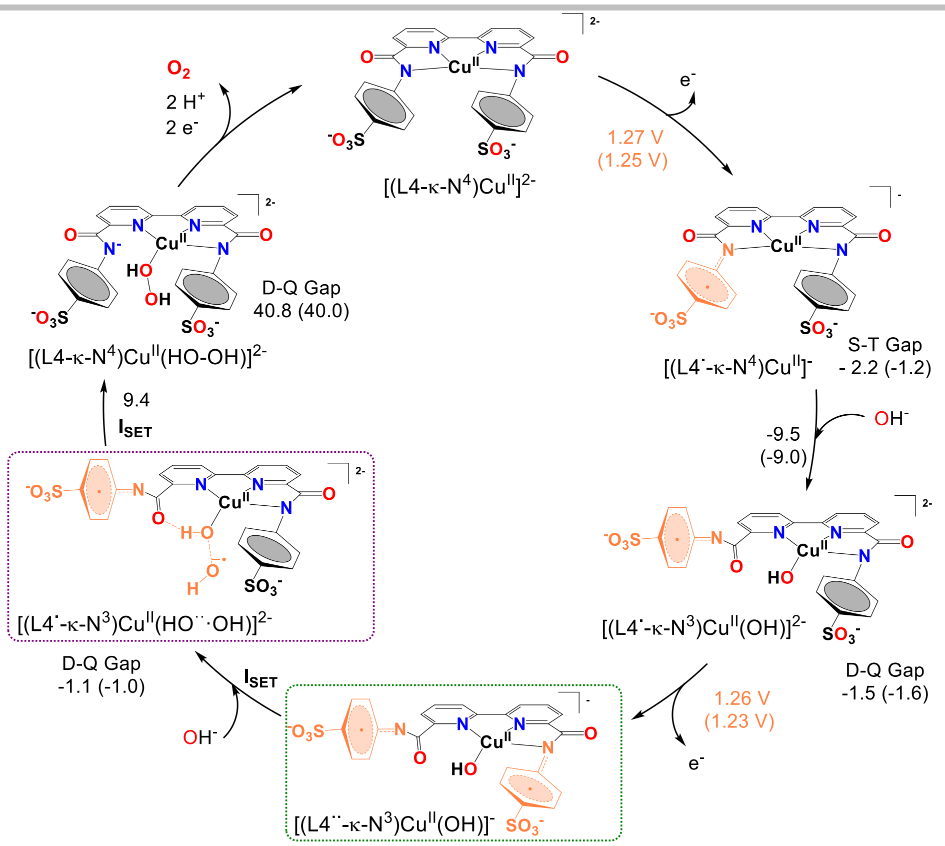

Figure S65. Influence of the basis set size in the catalytic cycle depicted in Figure 3 of the main text. Small basis set (6-31+G(d)/LANL2TZ(f)) and large basis set (6-311++G(3d,2p)) shown in brackets, calculated energies are very similar and no influence is observed in oxidation potentials, OH coordination or spin state gap. Energies in kcal/mol.

Effect of explicit solvation in spin state and reaction profile for the SET-WNA mechanism

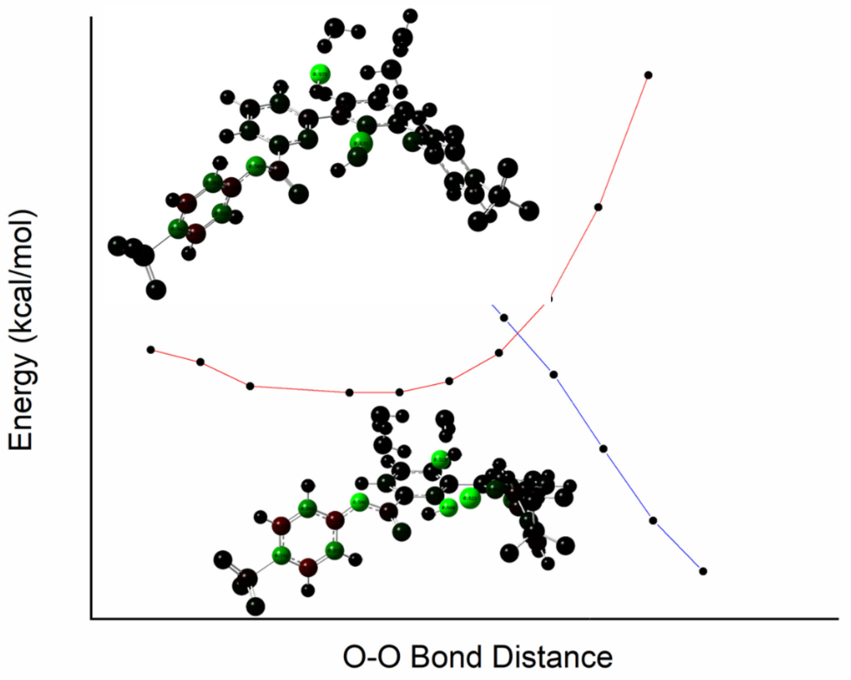

Figure S66. Potential energy relaxed scan of the intramolecular $(H_2O)_3$HO---OH SET- WNA using catalyst for **[(L4$^{\cdot}$-*κ*-N^3)Cu(OH)]$^{2-}$** in the quartet state.

Water Nucleophilic Attack potential energy surface

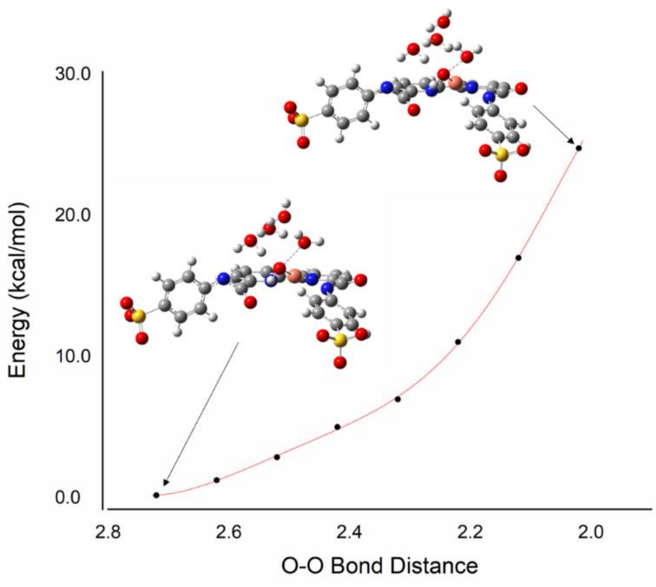

Figure S67. Potential energy relaxed scan of the intramolecular $(H_2O)_3H_2O$---OH Water Nucleophilic Attack using catalyst for **[(L4$^{\cdot}$-*κ*-N^3)Cu(OH)]$^{2-}$** in the quartet state.

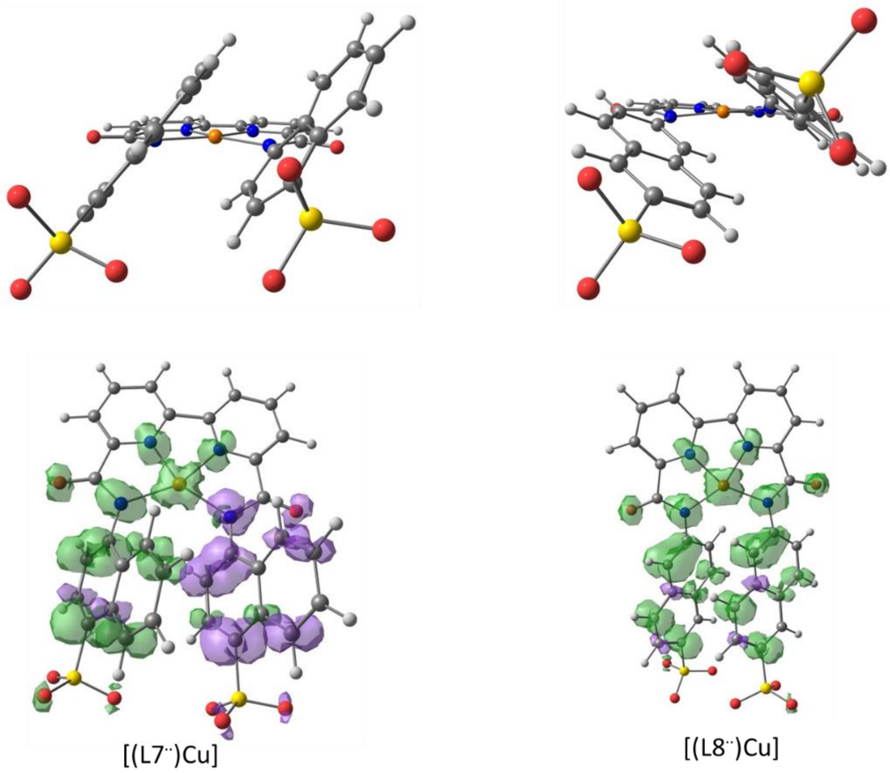

Figure S68. DFT optimized structures with representation of the spin distribution for the species **[(L7$^{\cdot\cdot}$)Cu] and [(L8$^{\cdot\cdot}$)Cu]** showing the delocalization of the radicals over the naphtyl-amide moiety.

Table S6. Summary of energy data

Complex (multiplicity)	Ep (H)	ZPVCs (H)	G (H)
**OH^-**	-75.947690	-0.00776	-75.95545
**$[(L4)Cu]^{2-}$ (d)**	-2736.833258	0.290922	-2736.542336
**$[(H_2L4)Cu]$ (d)**	-2737.702827	0.316735	-2737.386092
**$[(HL4)Cu]^-$ (d)**	-2737.275645	0.303058	-2736.972587
**$\{[(L4)Cu]\cdots H_2O\}^{2-}$ (d)**	-2813.280029	0.310897	-2812.969132
**$[(L4)Cu(OH)_{apical}]^{3-}$ (d)**	-2812.786660	0.297645	-2812.489015
**$[(L4)Cu]^-$ (s)**	-2736.628191	0.293373	-2736.334818
**$[(L4)Cu]^-$ (t)**	-2736.629034	0.290744	-2736.33829
**$[(L4)Cu(OH_2)_{apical}]^-$ (s)**	-2813.074690	0.315265	-2812.759425
**$[(L4)Cu(OH_2)_{apical}]^-$ (t)**	-2813.071800	0.310034	-2812.761766
**$[(L4)Cu(OH)_{apical}]^{2-}$ (s)**	-2812.585820	0.301714	-2812.284106
**$[(L4)Cu(OH)_{apical}]^{2-}$ (t)**	-2812.592184	0.299074	-2812.29311
**$[(L4)Cu(OH)]^{3-}$ (d)**	-2812.787172	0.296464	-2812.490708
**$[(L4)Cu(OH)]^{2-}$ (s)**	-2812.585856	0.299376	-2812.28648
**$[(L4)Cu(OH)]^{2-}$ (t)**	-2812.602386	0.294792	-2812.307594
**$[(L4)Cu(OH_2)]^-$ (s)**	-2813.036962	0.312095	-2812.724867
**$[(L4)Cu(OH_2)]^-$ (t)**	-2813.069746	0.309626	-2812.76012
**$[(L4)Cu(OH)]^-$ (d)**	-2812.397681	0.295942	-2812.101739
**$[(L4)Cu(OH)]^-$ (q)**	-2812.400366	0.296262	-2812.104105
**$[(L4)Cu(OH_2)]$ (d)**	-2812.847401	0.310589	-2812.536812
**$[(L4)Cu(OH_2)]$ (q)**	-2812.858131	0.308672	-2812.549459
**$[(L4)Cu(O)]^{2-}$ (d)**	-2811.927027	0.285831	-2811.641196
**$[(L4)Cu(O)]^{2-}$ (q)**	-2811.926833	0.28338	-2811.643453
**$[(L4)Cu(HO\cdots OH)]^{2-}$ (d)**	-2888.362321	0.304879	-2888.057442
**$[(L4)Cu(HO\cdots OH)]^{2-}$ (q)**	-2888.363391	0.304178	-2888.059213
**$[(L4)Cu(HO\text{-}OH)]^{2-}$ (d)**	-2888.386577	0.311244	-2888.075334
**$[(L4)Cu(HO\text{-}OH)]^{2-}$ (q)**	-2888.316567	0.306258	-2888.01031
**$[(L4)Cu(OH)_2]^{4-}$ (d)**	-2888.732095	0.304238	-2888.427857
**$[(L4)Cu(OH)_2]^{3-}$ (s)**	-2888.530842	0.307416	-2888.223427
**$[(L4)Cu(OH)_2]^{3-}$ (t)**	-2888.548855	0.300718	-2888.248137
**$[(L4)Cu(OH)_2]^{2-}$ (dos)**	-2888.350618	0.308289	-2888.042329
**$[(L4)Cu(OH)_2]^{2-}$ (q)**	-2888.364446	0.303684	-2888.060762

**[(L4)Cu(OH)(O)]$^{3-}$ (d)**	-2887.887073	0.297118	-2887.589955
**[(L4)Cu(OH)(O)]$^{3-}$ (q)**	-2887.874387	0.291281	-2887.583106
**[(L4)Cu(OH)(HO···OH)]$^{3-}$ (d)**	-2964.313943	0.313294	-2964.000649
**[(L4)Cu(OH)(HO···OH)]$^{3-}$ (q)**	-2964.314770	0.313757	-2964.001013
**[(L4)Cu(OH)(HO-OH)]$^{3-}$ (d)**	-2964.379201	0.32112	-2964.058081
**[(L4)Cu(OH)(HO-OH)]$^{3-}$ (q)**	-2964.298809	0.317063	-2963.981746
**[(L5)Cu]$^{2-}$ (d)**	-2736.83333164	0.292585	-2736.5407
**[(L5)Cu(OH)]$^{3-}$ (d)**	-2812.783247	0.296405	-2812.4868
**[(L5)Cu]$^{-}$ (s)**	-2736.633847	0.294614	-2736.3392
**[(L5)Cu]$^{-}$ (t)**	-2736.632465	0.290809	-2736.3417
**[(L5)Cu(OH)]$^{2-}$ (s)**	-2812.585527	0.301584	-2812.2839
**[(L5)Cu(OH)]$^{2-}$ (t)**	-2812.590105	0.299515	-2812.2906
**[(L5)Cu] (d)**	-2736.422273	0.293613	-2736.1287
**[(L5)Cu] (q)**	-2736.4213	0.292433	-2736.1289
**[(L7)Cu] (dos)**	-3043.74310150	0.381056	-3043.362046
**[(L8)Cu] (q)**	-3043.74912970	0.379864	-3043.369266

Cartesian coordinates (Å) and calculated potential energies (atomic units)

OH^- E= -75.947690 H

O	-1.77132600	0.29368800	0.00000000
H	-2.09552100	1.20918600	0.00000000

Complex $[(L4)Cu]^{2-}$ and related species:

$[(L4)Cu]^{2-}$ (d) E= -2736.833258 H

Cu	11.11623100	10.97931500	17.36338100
S	8.72740800	11.35298500	10.43890800
S	13.48464600	12.18400600	10.49693100
N	9.40666100	10.49060200	16.34875600
N	9.97455400	10.33433600	18.86902400
N	12.29167600	11.27054700	18.95008500
N	12.79833600	11.66855500	16.44441500
O	7.27965100	9.58900900	16.71571200
O	14.92505600	12.49733400	16.94821200
O	7.23564200	11.38690600	10.24231100
O	9.35512000	10.18326200	9.73702700
O	9.37956800	12.65925900	10.10167100
O	12.89332600	13.49785200	10.07698300
O	12.81858600	11.00500600	9.85539400
O	14.97985000	12.16089300	10.32275500
C	9.23456100	10.66480500	14.95970300
C	9.60350000	11.89271500	14.38667100
C	9.47847700	12.10918200	13.01432900
C	8.98801000	11.08612300	12.19949800
C	8.63958300	9.84629200	12.74971900
C	8.76422900	9.63648500	14.12064100
C	8.41837900	9.96295200	17.09526900
C	8.76038400	9.88023800	18.56789000
C	7.92177200	9.40434800	19.57615500
C	8.39764900	9.43356300	20.89245200
C	9.67547600	9.92742600	21.18298500
C	10.46015300	10.38153400	20.12199300
C	11.83295300	10.94645100	20.17107500
C	12.64136300	11.16169800	21.28821400
C	13.91123500	11.71658100	21.08584800
C	14.35719800	12.03913700	19.79827200
C	13.49652200	11.78991900	18.72907800
C	13.79972500	12.03462800	17.26596100
C	12.95206000	11.81387000	15.04820500
C	13.27182700	13.05088300	14.46023600
C	13.39399600	13.16500300	13.07769000
C	13.19239100	12.04131000	12.26733000
C	12.84761900	10.81147700	12.83375400
C	12.72246000	10.70448500	14.21926500
H	14.55703800	11.89628900	21.93986600
H	15.33883800	12.46592000	19.62698700
H	12.29331300	10.90545900	22.28308200
H	10.04693600	9.95993400	22.20153900
H	7.76861400	9.07230500	21.70027600
H	6.93259400	9.03035200	19.33788200
H	8.50444100	8.67283900	14.54497600
H	10.00112800	12.67438300	15.02708400
H	9.76921700	13.06136500	12.58407400
H	8.27439200	9.04506800	12.11377100
H	12.43831400	9.75749500	14.66893800
H	13.42010700	13.92097300	15.09154800
H	13.64318300	14.12442600	12.63446800
H	12.67022700	9.94733700	12.20221900

$[(H_2L4)Cu]$ (d) E= -2737.702827 H

Cu	11.16912300	10.98999100	17.38722000
S	9.04134800	11.38747200	10.47216700
S	13.18105500	12.15055900	10.54984000
N	9.42014900	10.61566600	16.36417900
N	9.99772400	10.37382100	18.87769200
N	12.35073900	11.22157200	18.96968400
N	12.86424000	11.63668700	16.47288500
O	7.26590600	9.78472100	16.71940600
O	15.03040200	12.35536000	16.97461400
O	7.68642400	11.11410900	9.98214000
O	9.94220500	10.20608300	9.84042000
O	9.65344600	12.69125800	10.12070400
O	12.27592000	13.43762200	10.18968000
O	12.56592500	10.95225100	9.93115300
O	14.53309600	12.52401900	10.12180900
C	9.26672300	10.78812500	14.97958000
C	9.64521400	12.01654100	14.40893800
C	9.58825100	12.21464500	13.03279400
C	9.14555200	11.16316600	12.22439000
C	8.75737500	9.92916900	12.76391000
C	8.81984900	9.74736700	14.13940700
C	8.41455500	10.11007200	17.10618100
C	8.75590400	9.99959100	18.57740200
C	7.88803500	9.58043300	19.58566700
C	8.36487800	9.57983000	20.90204600
C	9.67087200	9.99308600	21.19242800
C	10.48266300	10.39723000	20.13189700
C	11.87961200	10.89848500	20.18603000
C	12.69653800	11.06414300	21.30544200
C	13.98845500	11.56809000	21.10973200
C	14.44692800	11.89137400	19.82648300
C	13.57646200	11.69401400	18.75493800
C	13.88890800	11.94663500	17.29498500
C	12.99801600	11.77199600	15.08203000
C	13.46420000	12.95920800	14.48113200
C	13.51329200	13.07560200	13.09786000
C	13.09119900	11.99563300	12.31031400
C	12.62504200	10.80622800	12.87876000
C	12.58150400	10.70554100	14.26551700
H	14.64229700	11.70687500	21.96518700
H	15.44584400	12.27836700	19.66055600
H	12.33813400	10.80826400	22.29659700
H	10.04189400	10.00676600	22.21159000
H	7.71333000	9.26242900	21.71035500
H	6.87578800	9.27405600	19.34768000
H	8.54253800	8.79144100	14.56908300
H	10.01013200	12.80878200	15.05467200
H	9.90018500	13.15838300	12.59929200
H	8.42385300	9.11905100	12.12258400
H	12.19689100	9.80444700	14.73256800
H	13.76806400	13.79319400	15.10352500
H	13.86467100	13.99661200	12.64270500
H	12.28462200	9.98587500	12.25665900
H	10.91538000	10.41899800	9.92455200
H	11.30410300	13.20659300	10.22691600

$[(HL4)Cu]^-$ (d) E= -2737.275645 H

Cu	11.15877700	10.98593500	17.38530900
S	8.94947500	11.53170300	10.49633600
S	13.30102800	12.04483500	10.49896300
N	9.42244800	10.56864300	16.36691800
N	9.99960200	10.35588900	18.88169000
N	12.33640500	11.24487200	18.96775400
N	12.84179900	11.64138600	16.46661200
O	7.27385600	9.72087300	16.72918000
O	14.99020300	12.41774700	16.95862800

O	7.61400200	11.11087200	10.03683300
O	9.95276400	10.48022800	9.81580300
O	9.39500700	12.90293900	10.19164600
O	12.76053500	13.37634800	10.09134200
O	12.49786800	10.89276000	9.92611000
O	14.75521800	11.87835900	10.18813000
C	9.26336000	10.77272600	14.98651600
C	9.69813800	11.99422100	14.44145700
C	9.61753700	12.23876600	13.07383000
C	9.09395000	11.24391300	12.24330200
C	8.66323100	10.01362000	12.75718600
C	8.75033300	9.78157600	14.12513500
C	8.42239900	10.05491300	17.11032300
C	8.76896500	9.94701600	18.58104200
C	7.91509500	9.49931600	19.58919400
C	8.39334100	9.50916800	20.90507400
C	9.68705700	9.95969300	21.19558800
C	10.48593300	10.38907600	20.13502100
C	11.87162400	10.92236900	20.18658800
C	12.68480200	11.11609100	21.30415800
C	13.96504200	11.64750500	21.10318500
C	14.41666000	11.96894500	19.81695600
C	13.55102600	11.74205400	18.74707200
C	13.85642400	11.98365600	17.28282400
C	12.98345100	11.75650400	15.06808300
C	13.34678000	12.96595800	14.44906900
C	13.41593700	13.05413000	13.06088600
C	13.12131000	11.93001800	12.28058200
C	12.75213600	10.72174500	12.87782600
C	12.67982100	10.64302400	14.26747000
H	14.61514100	11.80955300	21.95744900
H	15.40660000	12.37704800	19.64776400
H	12.33266500	10.86140400	22.29793100
H	10.05882900	9.98090500	22.21436000
H	7.75248800	9.16957700	21.71296600
H	6.91210300	9.16343100	19.35183800
H	8.43357800	8.82734400	14.53016700
H	10.11909000	12.74479900	15.10296400
H	9.96341400	13.18059000	12.66290300
H	8.26856800	9.24434500	12.10043000
H	12.36812800	9.71907300	14.74558500
H	13.55882400	13.83781200	15.05908800
H	13.68813600	13.99443400	12.59119200
H	12.50670200	9.85809200	12.26890900
H	10.93842800	10.74246400	9.92904100

$\{[(L4)Cu]\cdots H_2O\}^{2-}$ (d) E= -2813.280029 H

Cu	11.07591900	10.98853100	17.35803700
S	8.69338600	11.34579700	10.42733800
S	13.46328100	12.13197800	10.48624400
N	9.36925500	10.51275400	16.33934100
N	9.93155100	10.33312200	18.85552300
N	12.24610800	11.27843600	18.94887900
N	12.76874900	11.66961500	16.43941600
O	7.25132900	9.58599300	16.68330800
O	14.91882400	12.43237300	16.95725500
O	7.20044000	11.37868100	10.23793400
O	9.31897300	10.17339100	9.72934400
O	9.34166200	12.65185900	10.08124600
O	12.88013400	13.44849100	10.06092500
O	12.78129600	10.95550300	9.85735100
O	14.95663000	12.09694700	10.30193400
C	9.20166700	10.68406700	14.94876100
C	9.46594400	11.94277000	14.38637400
C	9.34106500	12.15150300	13.01273600
C	8.96329300	11.08919700	12.18803900
C	8.72166600	9.82073200	12.72941100
C	8.84063700	9.62007200	14.10233700
C	8.38245400	9.97056700	17.07533800
C	8.71712700	9.88280400	18.54935100
C	7.87540700	9.40370600	19.55318100
C	8.34881500	9.42487100	20.87066600
C	9.62794200	9.91225800	21.16616200
C	10.41568900	10.37031900	20.10910800
C	11.79238700	10.92495500	20.16346000
C	12.61300900	11.09376300	21.27967500
C	13.89476700	11.62200700	21.08125500
C	14.34235500	11.95725000	19.79755400
C	13.46791700	11.75680100	18.72920900
C	13.78115300	12.00324700	17.26829900
C	12.92862200	11.80712600	15.04300200
C	13.40431300	12.99363900	14.45377500
C	13.53284800	13.09172900	13.07101500
C	13.18181500	12.00585700	12.25945200
C	12.68075600	10.83156500	12.82607900
C	12.55026800	10.74044800	14.21217100
H	14.55129700	11.76393200	21.93417100
H	15.33733000	12.35252000	19.62791800
H	12.26729900	10.81692000	22.26976300
H	9.99794200	9.93694900	22.18548900
H	7.71685900	9.06188800	21.67541400
H	6.88576800	9.03351300	19.31081200
H	8.66321900	8.63593400	14.52334000
H	9.78211700	12.75439000	15.03529700
H	9.54688400	13.12858800	12.58866300
H	8.44506100	8.99148800	12.08503800
H	12.13991200	9.84078400	14.66086200
H	13.66893300	13.83684200	15.08255200
H	13.90457900	14.01133800	12.62875500
H	12.38563900	9.99974600	12.19547000
O	13.01143300	8.44482600	17.21317000
H	12.48552600	8.61511600	18.01326600
H	13.05586900	9.31908200	16.78450500

$[(L4)Cu(OH)_{apical}]^{3-}$ (d) E= -2812.786660 H

Cu	11.24523900	10.66285000	17.32412900
S	8.68520600	11.36095400	10.44551600
S	13.48163800	12.22351500	10.48058700
N	9.39721300	10.41446400	16.33937700
N	10.00522500	10.24926500	18.85403300
N	12.31186000	11.22937200	18.93607300
N	12.85706000	11.66433900	16.43429500
O	7.24555000	9.58386100	16.74365100
O	14.96565400	12.51676400	16.98590500
O	12.06202800	8.64792000	17.03442200
O	7.29032700	10.90254400	10.12113400
O	9.72370400	10.52680900	9.75009200
O	8.87680400	12.83015500	10.21950000
O	12.46016200	13.22606800	10.02358600
O	13.26737200	10.86402700	9.88621100
O	14.88604800	12.72609800	10.28362300
C	9.20316100	10.60721000	14.95873000
C	9.58045600	11.83862600	14.39635300
C	9.42999300	12.08087300	13.03122900
C	8.90172500	11.08187700	12.20993500
C	8.54780300	9.83728300	12.74630400
C	8.69742300	9.60215900	14.11097200
C	8.40957000	9.92021400	17.09904400
C	8.76733200	9.84860100	18.56824300
C	7.90032200	9.46377800	19.59282200
C	8.36857600	9.52800200	20.90998400
C	9.66251000	9.98497400	21.18605900
C	10.47402900	10.35169000	20.11053500
C	11.84647900	10.91601200	20.15802100
C	12.63639300	11.15777200	21.28375500
C	13.90086400	11.72741100	21.09255700
C	14.35290500	12.04738400	19.80737500
C	13.50963700	11.77349800	18.72868200
C	13.83630000	12.03179000	17.27440700
C	13.02220700	11.81727900	15.04396500

C	13.42168200	13.03601300	14.46014800
C	13.54381100	13.15169900	13.07819600
C	13.26770300	12.04960700	12.25845200
C	12.84296700	10.84259200	12.81822300
C	12.71723200	10.73325700	14.20350100
H	12.95180800	8.85188400	16.70302800
H	14.53444800	11.92411000	21.95211200
H	15.32784800	12.49192500	19.64288100
H	12.27891800	10.90978500	22.27738800
H	10.02338200	10.06253400	22.20608800
H	7.71810500	9.23648500	21.72903300
H	6.89273100	9.13550900	19.36432100
H	8.42562700	8.63766800	14.52597200
H	10.00012400	12.60575700	15.03999500
H	9.72198000	13.03772500	12.61210100
H	8.15021200	9.05512900	12.10607600
H	12.37212100	9.80556600	14.64976200
H	13.63252100	13.89036800	15.09459700
H	13.85844300	14.09612200	12.64333800
H	12.60957300	9.99574300	12.18164200

[(L4)Cu]⁻ (s) E= -2736.628191 H

Cu	11.15923000	10.95481100	17.46817700
S	8.94358300	10.83494500	10.42884700
S	13.22360000	12.67087700	10.60349300
N	9.56617400	10.74079500	16.37695700
N	9.97453000	10.49527400	18.87726000
N	12.36190500	11.10855500	18.92917100
N	12.73207700	11.43790900	16.43450200
O	7.34615800	10.09396400	16.59475100
O	14.94456200	12.06293900	16.76145000
O	7.50464800	10.41835700	10.30711200
O	9.87637100	9.83041400	9.82285900
O	9.18505500	12.23279400	9.95452700
O	12.29229100	13.79631500	10.26794700
O	12.94880100	11.42064200	9.83040400
O	14.66593500	13.08898300	10.53779300
C	9.52529200	10.76573800	14.97353200
C	9.58496900	11.99096200	14.29019400
C	9.45879400	12.02118700	12.90125200
C	9.27862400	10.82480900	12.20337700
C	9.27055100	9.59071600	12.87013100
C	9.40901900	9.56159400	14.25063900
C	8.46279200	10.33573400	17.07597600
C	8.71174200	10.24075800	18.55725200
C	7.79470200	9.92888200	19.55669400
C	8.25667000	9.90260600	20.87859900
C	9.59541000	10.18326500	21.18351300
C	10.46137500	10.48841800	20.13603800
C	11.89290400	10.83936100	20.16544600
C	12.76994000	10.92282600	21.24468700
C	14.10104100	11.28102200	20.99199700
C	14.54260000	11.55228400	19.69083400
C	13.61580700	11.44980400	18.65801900
C	13.84102300	11.69482500	17.19049400
C	12.74883000	11.72500300	15.06053900
C	12.84820500	13.05995000	14.62073300
C	12.95568300	13.33780200	13.26545400
C	12.93324600	12.28211800	12.34193700
C	12.77326400	10.95933800	12.76078300
C	12.67935900	10.68079100	14.12436000
H	14.79924200	11.34846600	21.82012900
H	15.56806300	11.83340400	19.47891800
H	12.42278900	10.71057500	22.25003300
H	9.95568500	10.17212200	22.20624000
H	7.56766900	9.66578600	21.68280800
H	6.76196500	9.71668000	19.30391600
H	9.39780400	8.61908800	14.78941400
H	9.70930600	12.91062500	14.85237300
H	9.49076900	12.96484000	12.36839700
H	9.15134100	8.66485000	12.31613200
H	12.57222700	9.65865000	14.47244300
H	12.86986100	13.86021000	15.35421400
H	13.06073800	14.36372000	12.92669300
H	12.73120000	10.15558000	12.03425400

[(L4)Cu]⁻ (t) E= -2736.629034 H

Cu	11.14799100	11.05888300	17.35713600
S	8.72937700	11.30409100	10.44428700
S	13.51195000	12.15348500	10.49495600
N	9.33567700	10.56632300	16.32285700
N	10.01586900	10.30050600	18.83701000
N	12.31040500	11.32329000	18.94299500
N	12.82170100	11.75836900	16.45071600
O	7.26799500	9.56492900	16.76532100
O	14.91543100	12.65116600	16.96941400
O	7.24627000	11.34622600	10.23809600
O	9.36463500	10.09498100	9.83276000
O	9.41485700	12.58283100	10.09976600
O	12.89681200	13.45090400	10.06085100
O	12.84947500	10.95414200	9.88847500
O	15.00399900	12.14313000	10.29992100
C	9.17432900	10.66800800	14.98269800
C	9.80597400	11.78703400	14.34495400
C	9.66475400	12.00186600	12.98846000
C	8.93332100	11.07795300	12.22565900
C	8.34355800	9.93662400	12.81792200
C	8.44307100	9.73269300	14.17481300
C	8.38985100	9.91788400	17.12783500
C	8.81535300	9.79001100	18.56050800
C	8.02450000	9.22832100	19.56270600
C	8.52865600	9.22844700	20.86707200
C	9.78186100	9.78453400	21.14254000
C	10.51918700	10.32339900	20.08511900
C	11.86063500	10.95352600	20.15306300
C	12.65302500	11.18230800	21.27908900
C	13.89649000	11.79969600	21.09569800
C	14.33642500	12.16212500	19.81705400
C	13.49464800	11.89510700	18.73798700
C	13.80399700	12.15746300	17.28048600
C	12.99782800	11.87366500	15.05373600
C	13.25498800	13.11112700	14.43807500
C	13.37830100	13.19572400	13.05344200
C	13.24482600	12.04102700	12.27237000
C	12.97789100	10.80678800	12.86876000
C	12.84688400	10.72888600	14.25570800
H	14.52722500	11.99420900	21.95756100
H	15.29959100	12.63355800	19.65830700
H	12.31294400	10.89143600	22.26714600
H	10.17569200	9.79841300	22.15266100
H	7.94201100	8.80023700	21.67349400
H	7.05113100	8.81397000	19.32699100
H	8.01975500	8.84533000	14.62528500
H	10.35777600	12.48277700	14.96659400
H	10.12260700	12.86337300	12.51664800
H	7.81830900	9.21451300	12.20121800
H	12.61783600	9.77945400	14.73123300
H	13.34449600	14.00413600	15.04822400
H	13.56934700	14.15629000	12.58485700
H	12.85869200	9.91704700	12.25947800

[(L4)Cu(OH$_2$)$_{apical}$]⁻ (s) E= -2813.074690 H

Cu	11.09202300	11.05388900	17.47512000
S	9.05682800	10.57263700	10.43733600
S	13.13257200	12.87915600	10.64706300
N	9.48364400	10.95111300	16.39325200
N	9.88942400	10.66799900	18.88802400
N	12.31977300	11.09570200	18.92159400

N	12.69478700	11.34856500	16.41504800
O	7.24965500	10.35889800	16.60719600
O	14.93804800	11.86283200	16.72748200
O	12.28502500	8.15390000	17.10176200
O	7.65290400	10.05984400	10.28116700
O	10.07810900	9.58610800	9.95734000
O	9.23931200	11.94306600	9.86687500
O	12.14519300	13.97394700	10.37427400
O	12.90684500	11.65506400	9.81853500
O	14.55316700	13.36596900	10.58730000
C	9.47240600	10.87404900	14.99091400
C	9.53663700	12.04300400	14.21708900
C	9.44738200	11.96402700	12.82676300
C	9.30609100	10.71451800	12.21950600
C	9.29449200	9.53562900	12.97915600
C	9.38952200	9.61540000	14.36086900
C	8.36801500	10.57921800	17.09314000
C	8.61415800	10.47467100	18.57357200
C	7.69423200	10.17004600	19.57242700
C	8.16783300	10.08003700	20.88733800
C	9.52221300	10.28319600	21.18495200
C	10.39145200	10.58187000	20.13790400
C	11.84677200	10.82509400	20.15601100
C	12.74486700	10.79372600	21.22088200
C	14.09916700	11.03887700	20.95596700
C	14.54578500	11.30085300	19.65439600
C	13.59761000	11.31143100	18.63547400
C	13.82332900	11.55044600	17.16661500
C	12.70951100	11.70091000	15.05420300
C	12.78654500	13.05783500	14.68177200
C	12.88071700	13.40551100	13.34185700
C	12.87648800	12.39628700	12.36790900
C	12.74690600	11.05105900	12.72110200
C	12.65969100	10.70298000	14.06948500
H	12.58930700	8.97308300	16.67092300
H	14.81211400	11.01934300	21.77399600
H	15.59036200	11.48718400	19.43160300
H	12.39498200	10.57702600	22.22440100
H	9.89435900	10.21052600	22.20099600
H	7.47775700	9.84410200	21.69087500
H	6.65228100	10.00290600	19.32357600
H	9.37834700	8.71645100	14.96954200
H	9.63186100	13.00493200	14.70976400
H	9.48133800	12.86456400	12.22405700
H	9.20810000	8.56786100	12.49496400
H	12.57390700	9.66335900	14.36862600
H	12.79481600	13.82103800	15.45408600
H	12.96047800	14.44957000	13.05534800
H	12.71816300	10.28489700	11.95453700
H	11.76710900	8.47602600	17.85946900

[(L4)Cu(OH$_2$)$_{apical}$]$^-$ (t) E= -2813.071800 H

Cu	11.23570200	10.94872700	17.32289000
S	9.02812800	10.55797100	10.46109100
S	13.43921800	12.76470700	10.56540000
N	9.12372200	10.87111800	16.40181100
N	9.95367000	10.46785200	18.87097600
N	12.38986400	11.17492100	18.92627400
N	12.91351000	11.63903300	16.44336900
O	6.99450200	10.12237800	16.96864100
O	15.06607300	12.36855500	16.95166300
O	11.30299900	8.88086400	16.29575700
O	7.64242900	10.15082200	10.06993900
O	10.04970300	9.50155000	10.16780300
O	9.41543900	11.91094200	9.96376300
O	12.45073600	13.84789400	10.24410600
O	13.16726900	11.48674600	9.83257200
O	14.85995700	13.23269100	10.40259000
C	9.02403100	10.76622500	15.06084800
C	9.76081800	11.72245500	14.28855800
C	9.73187500	11.69067400	12.90955000
C	9.00346100	10.67584800	12.26450800
C	8.29858400	9.69587200	12.99773900
C	8.29218900	9.73817100	14.37476300
C	8.17561400	10.33699100	17.26000100
C	8.66985600	10.15784100	18.66493700
C	7.84696800	9.73401600	19.70836700
C	8.39982800	9.65096000	20.98870800
C	9.73516500	10.00241900	21.19791200
C	10.49936700	10.41422300	20.10225600
C	11.91608400	10.84999400	20.13895200
C	12.74394300	10.97225200	21.25742200
C	14.04760500	11.44331800	21.06646200
C	14.50609000	11.78031500	19.78768900
C	13.62750400	11.62364900	18.71743200
C	13.93462900	11.92189700	17.26814400
C	13.05482700	11.89956000	15.05971200
C	13.10788900	13.22223300	14.58692300
C	13.20147000	13.47817700	13.22189800
C	13.24097400	12.40777700	12.31914600
C	13.18085200	11.08974800	12.77427500
C	13.08461700	10.83872600	14.14499800
H	12.22442600	8.59266700	16.41873000
H	14.70737600	11.55062800	21.92183900
H	15.51137700	12.15063000	19.62242800
H	12.38540000	10.71574900	22.24790800
H	10.17078100	9.95822900	22.18981200
H	7.78987400	9.32349200	21.82459100
H	6.80939400	9.48544600	19.51806700
H	7.77610000	8.97441100	14.94275400
H	10.31938700	12.48622100	14.81681200
H	10.27337600	12.42985900	12.33082200
H	7.76567900	8.90742100	12.47658300
H	13.02773800	9.81797300	14.50936500
H	13.06734000	14.04299300	15.29714800
H	13.23801300	14.50264100	12.86370500
H	13.19917700	10.26558100	12.06915500
H	10.78181400	8.29817900	16.87586700

[(L4)Cu(OH)$_{apical}$]$^{2-}$ (s) E= -2812.585820 H

Cu	11.16187400	10.83682400	17.43880600
S	9.06001300	10.59122700	10.44080000
S	13.19372200	12.80996500	10.61050600
N	9.49175200	10.93850800	16.39960000
N	9.95558700	10.54587400	18.87237800
N	12.36816400	11.03811700	18.90438100
N	12.71953500	11.47918100	16.42526900
O	7.27680800	10.27529800	16.62783900
O	14.95584000	12.01813700	16.74403600
O	11.55016100	8.67508200	17.17163200
O	7.64854300	10.10000400	10.27946900
O	10.06619500	9.59141600	9.95543500
O	9.26387800	11.96122600	9.87543500
O	12.22640700	13.91043000	10.28999200
O	12.95736600	11.56370500	9.81750500
O	14.62235500	13.27430100	10.54584000
C	9.46916700	10.86541600	14.99802300
C	9.56453600	12.03478300	14.22784600
C	9.47503200	11.96434100	12.83654400
C	9.30890100	10.72136000	12.22299600
C	9.26803800	9.54004800	12.97838800
C	9.35609100	9.61218200	14.36079000
C	8.40262200	10.50474200	17.10154300
C	8.67476000	10.36174800	18.57121800
C	7.76295100	10.05206100	19.57730900
C	8.24584900	9.95220900	20.88726100
C	9.60141200	10.16091600	21.17396500
C	10.46275400	10.46377700	20.12109100
C	11.90706800	10.75414100	20.13966500
C	12.79763200	10.78860700	21.21140700

C	14.13276600	11.12705700	20.95415200
C	14.56268600	11.43034600	19.65650900
C	13.62353700	11.37124000	18.62982100
C	13.84072700	11.66114200	17.17001200
C	12.73818800	11.78081900	15.05326100
C	12.79853300	13.12358600	14.63311400
C	12.90324900	13.42641000	13.28185700
C	12.91567000	12.38614800	12.34217500
C	12.79431900	11.05294300	12.74151100
C	12.70470200	10.75026500	14.10078300
H	12.25442600	8.66786600	16.50263700
H	14.84079700	11.16007500	21.77609300
H	15.58932500	11.70537800	19.44178100
H	12.45571000	10.56139400	22.21538900
H	9.97976300	10.09549100	22.18827700
H	7.56210400	9.71359200	21.69547500
H	6.71781200	9.89342000	19.33607900
H	9.32272300	8.71208900	14.96609200
H	9.68354600	12.99247300	14.72364500
H	9.53037100	12.86682400	12.23832800
H	9.16200800	8.57626400	12.48992000
H	12.62455000	9.72025400	14.43301900
H	12.79304000	13.91365800	15.37799500
H	12.97751600	14.46082900	12.96068800
H	12.77692800	10.25972500	12.00237500

[(L4)Cu(OH)$_{apical}$]$^{2-}$ (t)E= -2812.592184 H

Cu	11.13347900	10.88375000	17.31941200
S	8.98076100	10.75542100	10.41091600
S	13.36891900	12.67344600	10.53311600
N	9.30663600	10.77257000	16.39523500
N	9.93665500	10.44966900	18.88982900
N	12.34051400	11.15335600	18.92487400
N	12.79863900	11.59539700	16.41548500
O	7.16183900	9.95449800	16.78054300
O	14.99971100	12.20744900	16.86545700
O	11.57760500	8.95698400	16.93291000
O	7.56228600	10.36691000	10.10275200
O	9.96111000	9.71892300	9.94470600
O	9.31856800	12.13638100	9.94340700
O	12.41776800	13.78185700	10.18440200
O	13.08314700	11.40132400	9.79647500
O	14.80406400	13.10674000	10.40524800
C	9.21540300	10.75307000	14.99916700
C	9.55820300	11.91373900	14.28260400
C	9.49348400	11.93053300	12.89040600
C	9.10260900	10.77525200	12.20874400
C	8.79400800	9.59825800	12.90642600
C	8.85018200	9.58619700	14.29392000
C	8.30999300	10.25749300	17.16407900
C	8.67099800	10.14237000	18.62439400
C	7.80826300	9.75986500	19.65070900
C	8.32207000	9.71862500	20.95251800
C	9.65627100	10.05685600	21.20671500
C	10.46033100	10.42880500	20.12760500
C	11.88629600	10.83509800	20.14745100
C	12.74881700	10.91388100	21.24224200
C	14.06930200	11.31969700	21.01212100
C	14.50914500	11.63644300	19.72084200
C	13.58937900	11.53356600	18.67756100
C	13.86202800	11.81629900	17.21779300
C	12.91086600	11.84211500	15.02729100
C	13.15782600	13.14429700	14.55539100
C	13.26679800	13.38816300	13.19049800
C	13.12357200	12.32989400	12.28388300
C	12.84328700	11.03956700	12.73586100
C	12.73245700	10.79952800	14.10793000
H	12.50219700	9.00571800	16.62504200
H	14.75989700	11.38738100	21.84706600
H	15.52908900	11.94896600	19.52780700
H	12.40384600	10.66455900	22.23987700
H	10.05840500	10.03407600	22.21377000
H	7.67896600	9.42520200	21.77636200
H	6.77656200	9.50522100	19.43629700
H	8.61783100	8.67964100	14.84263000
H	9.87270000	12.79845600	14.82731200
H	9.75337500	12.82921100	12.34223200
H	8.51011100	8.69769000	12.36996400
H	12.50177800	9.80262600	14.46881600
H	13.26879100	13.95745700	15.26619000
H	13.46599600	14.39515300	12.83591600
H	12.70717700	10.22927400	12.02776100

[(L4)Cu(OH)]$^{3-}$ (d) E= -2812.787172 H

Cu	11.51383100	10.87527900	17.08771600
S	5.86325100	7.66600700	10.88428500
S	14.70938800	12.55893600	10.63674800
N	8.37939600	8.57885800	16.27216600
N	10.00329000	10.23408500	18.57098700
N	12.35439600	11.36085400	18.82366900
N	13.27754400	11.75899600	16.42308600
O	7.64522100	10.78264100	16.64639500
O	15.16040900	12.86202900	17.24961100
O	10.85701200	10.36889600	15.38953500
O	5.02732200	8.87321400	10.56510600
O	5.01891400	6.43150600	11.05880800
O	6.98103100	7.45711400	9.90532900
O	13.49612000	13.18470800	10.00768000
O	15.02414300	11.20859900	10.06576400
O	15.89336300	13.48529700	10.61965200
C	7.77626200	8.44413300	15.01258700
C	8.52851700	7.87906200	13.96309800
C	7.96686000	7.65872000	12.70524800
C	6.62866100	7.99197000	12.47647800
C	5.85366300	8.53693800	13.50906200
C	6.42242100	8.76426900	14.75941900
C	8.21769800	9.68846800	16.96569600
C	8.80444000	9.66485800	18.36059400
C	8.03630200	9.14300000	19.41016700
C	8.51832500	9.23500100	20.71064800
C	9.74692600	9.85848500	20.93635600
C	10.46480100	10.34746700	19.84556500
C	11.77007500	11.03423200	19.98694400
C	12.39217200	11.36216800	21.19831000
C	13.61609300	12.03085600	21.16360300
C	14.20404100	12.35682600	19.93784500
C	13.53513600	11.99288600	18.77280200
C	14.06182800	12.25557700	17.38411100
C	13.64145700	11.95492500	15.07254900
C	13.72543600	13.24680800	14.51906700
C	14.05369300	13.42308300	13.17761400
C	14.30060500	12.30393100	12.37151500
C	14.21507000	11.01548800	12.90253400
C	13.88510100	10.84578600	14.24916200
H	10.10478800	9.76929100	15.52489000
H	14.11070600	12.29733700	22.09268700
H	15.15493900	12.87336000	19.87933500
H	11.93566500	11.10929500	22.14778500
H	10.13390300	9.95584900	21.94368000
H	7.94638000	8.83696300	21.54344800
H	7.07732300	8.68369200	19.19237800
H	5.81675900	9.18634700	15.55464900
H	9.56884100	7.62036000	14.14195700
H	8.56891000	7.23427000	11.90827400
H	4.81105500	8.78970900	13.33786900
H	13.81257900	9.84711300	14.66862200
H	13.52898200	14.10977100	15.14812400
H	14.11529000	14.42591000	12.76470900
H	14.40544500	10.15137800	12.27502700

$[(L4)Cu(OH)]^{2-}$ (s) E= -2812.585856 H

Cu	11.64512900	10.77920800	17.32850500
S	6.34180200	8.04066800	10.65608900
S	13.87408800	12.16024800	10.64357500
N	8.61978400	8.79448400	16.17495700
N	10.13527100	10.26508600	18.60755000
N	12.45364500	11.32258200	18.94431000
N	13.28386700	11.49077500	16.56678300
O	7.71801400	10.91835900	16.64968200
O	15.15808500	12.70268200	17.17604100
O	11.13392700	10.19298400	15.68909900
O	5.52986400	9.26538700	10.34174700
O	5.48165700	6.80841500	10.74857300
O	7.50367800	7.85759800	9.72507900
O	12.46871400	12.33598400	10.14595900
O	14.52705200	10.92025800	10.11756900
O	14.70854700	13.39123200	10.44695500
C	8.06375800	8.68500600	14.88806300
C	8.88051500	8.20672100	13.84488700
C	8.37583500	8.02380800	12.55686000
C	7.03268000	8.30567600	12.29403900
C	6.19468700	8.75986600	13.32121500
C	6.70557600	8.95037500	14.60214500
C	8.35762900	9.85414600	16.91470600
C	8.90566100	9.80351200	18.32845800
C	8.04292800	9.37698700	19.35000500
C	8.45610600	9.44614600	20.67197700
C	9.71310500	9.98415200	20.96384800
C	10.52693400	10.39308700	19.91686500
C	11.84005300	11.02657000	20.10120700
C	12.47461800	11.36387000	21.30207500
C	13.71787400	11.99746200	21.25168500
C	14.32413600	12.28614900	20.02250800
C	13.64865700	11.91418600	18.86974900
C	14.11398100	12.09631000	17.45901800
C	13.43761000	11.64957400	15.18169700
C	13.14885500	12.88679100	14.57446900
C	13.30247300	13.03899200	13.20224400
C	13.72940300	11.94696200	12.43162900
C	14.00432600	10.71046300	13.01974300
C	13.85252100	10.56124000	14.39815500
H	10.36110500	9.60123300	15.81748800
H	14.21749600	12.26723400	22.17658300
H	15.28900700	12.77583800	19.95378200
H	12.00915400	11.13919100	22.25437200
H	10.04655500	10.08611300	21.98999300
H	7.80745300	9.10755200	21.47366600
H	7.06003800	9.00319400	19.08261800
H	6.05086100	9.30050400	15.39311600
H	9.92435400	7.98497500	14.05090200
H	9.02553200	7.66792300	11.76392900
H	5.14753800	8.97063900	13.12301000
H	14.05693900	9.60869000	14.87571000
H	12.81402300	13.71638600	15.18972500
H	13.08902500	13.99532900	12.73486400
H	14.32965800	9.87379800	12.41147000

$[(L4)Cu(OH)]^{2-}$ (t) E= -2812.602386 H

Cu	12.07759000	11.10599500	16.53725000
S	3.28686500	6.63162400	13.01567300
S	16.56449900	13.71113300	11.30541600
N	8.65641400	8.21291500	15.02048300
N	10.26729500	10.06883500	17.60469800
N	12.33469200	11.49448200	18.47058100
N	13.78464700	12.26847400	16.43595500
O	9.06979500	10.46393700	15.03416600
O	15.16329800	13.55018700	17.81245700
O	11.96245800	10.70473600	14.70055900
O	2.51274200	7.88727600	12.76588100
O	2.73219300	5.82247300	14.15096500
O	3.51664700	5.81974000	11.78047800
O	15.83100700	14.88432500	10.72375800
O	16.51366800	12.49669000	10.42530400
O	17.96979200	14.06532700	11.70869500
C	7.43681200	7.91534100	14.55703900
C	7.28079400	6.71390900	13.79151700
C	6.03923400	6.34470600	13.31126400
C	4.92185400	7.15843300	13.57561400
C	5.04212500	8.34160400	14.33032500
C	6.27670000	8.72188400	14.81709100
C	8.97599600	9.39392600	15.64409900
C	9.28329900	9.29637300	17.11209400
C	8.47182600	8.49223700	17.91666000
C	8.66962500	8.51842600	19.29648700
C	9.66645300	9.33559200	19.81744000
C	10.45452500	10.09616700	18.94158700
C	11.54266000	10.98047500	19.42692000
C	11.76742700	11.29544200	20.77277700
C	12.82420900	12.14495700	21.09861100
C	13.64024400	12.65959800	20.08936400
C	13.36251900	12.30463100	18.77229300
C	14.18770900	12.77960300	17.60158400
C	14.44803200	12.62650100	15.24354900
C	14.50728100	13.96271400	14.80871900
C	15.13195600	14.28975800	13.60646700
C	15.69877200	13.27725300	12.82350400
C	15.64636000	11.94396700	13.24055400
C	15.01924600	11.62342000	14.44473500
H	11.03242900	10.48538600	14.53038000
H	13.00947900	12.40241200	22.13684400
H	14.47265500	13.31822400	20.30745100
H	11.13069200	10.89629300	21.55299100
H	9.83668900	9.37037200	20.88665600
H	8.05323000	7.91442200	19.95463600
H	7.69457700	7.87990700	17.47302700
H	6.37615200	9.63063100	15.40210200
H	8.15784300	6.10424400	13.59850000
H	5.92675100	5.43753700	12.72745200
H	4.16803200	8.95391400	14.52564800
H	14.96115900	10.58961800	14.77091800
H	14.05344600	14.74086600	15.41485500
H	15.16726500	15.32360100	13.27742800
H	16.08212400	11.16010900	12.62945300

$[(L4)Cu(OH_2)]^{-}$ (s) E= -2813.036962 H

Cu	11.68746300	10.91979500	17.15037000
S	6.30062900	7.43151700	10.80583500
S	13.98052400	12.93670700	10.71737000
N	8.21209600	8.55696400	16.39606300
N	10.12420100	10.40168800	18.49512400
N	12.58303200	11.16882700	18.81289600
N	13.52096300	11.27138800	16.43672900
O	8.45699800	10.86809400	16.08718100
O	15.51670200	12.16986200	17.16259400
O	10.91690200	10.80312400	15.35970700
O	5.50893500	8.62152200	10.34704600
O	5.44353400	6.20194100	10.94487300
O	7.51778900	7.18199400	9.96419500
O	12.96727400	14.02456700	10.54336400
O	13.70086600	11.72504800	9.88907700
O	15.39032200	13.42991700	10.58348600
C	7.77893200	8.35597300	15.06982800
C	8.54552100	7.55755300	14.20304600
C	8.11313800	7.29634400	12.90223800
C	6.89846400	7.82586300	12.45595100
C	6.11333800	8.60968000	13.30989200
C	6.55456600	8.87516200	14.60468900
C	8.47350600	9.77411600	16.77837700
C	8.90237600	9.91521300	18.22086200

C	8.01250500	9.57034800	19.24805100
C	8.39968100	9.73446200	20.57216200
C	9.67999200	10.21436000	20.85200300
C	10.52398100	10.52692800	19.79067500
C	11.90773300	10.98602500	19.96212100
C	12.56702000	11.21549800	21.17633400
C	13.89415000	11.64166700	21.16409100
C	14.56122200	11.82960300	19.94909100
C	13.85753100	11.57415700	18.78245100
C	14.38964400	11.73988100	17.40488300
C	13.64969300	11.66985900	15.11488900
C	13.72843200	13.04297500	14.76954000
C	13.82476100	13.41375200	13.44022900
C	13.83168700	12.41914000	12.44499000
C	13.74640800	11.06035600	12.76786700
C	13.64329100	10.68388900	14.10206200
H	9.90861300	10.88156900	15.49309600
H	14.40844300	11.82586700	22.10164700
H	15.58916100	12.17037800	19.89546900
H	12.05028400	11.05811300	22.11554300
H	10.01412800	10.33240300	21.87581000
H	7.72133600	9.48413800	21.38158400
H	7.03289600	9.18517900	18.98919600
H	5.95082900	9.48813300	15.26778600
H	9.49040700	7.14952600	14.55149100
H	8.72243200	6.68914400	12.24020400
H	5.16844200	9.01907100	12.96578500
H	13.56219100	9.63893000	14.38155100
H	13.70423200	13.79202000	15.55458400
H	13.88186200	14.46344500	13.17084300
H	13.74895600	10.31066800	11.98475400
H	11.16699800	11.59575800	14.84447800

[(L4)Cu(OH$_2$)]$^-$ (t) E= -2813.069746 H

Cu	12.11174300	11.06049100	16.62462700
S	3.35418400	6.68527000	13.04582500
S	16.37661300	13.66179000	11.25663700
N	8.76515100	8.20331800	14.97429700
N	10.34905400	10.04983900	17.59803900
N	12.35576700	11.48661700	18.52748400
N	13.79089900	12.22035600	16.48932500
O	9.34431600	10.41304800	14.95060500
O	15.16336600	13.54043300	17.83136500
O	12.04654500	10.46777600	14.69645000
O	2.58746200	7.94607800	12.80158500
O	2.81567100	5.88906500	14.19769600
O	3.55427200	5.86250400	11.81264700
O	15.60709400	14.82241600	10.69810800
O	16.30592900	12.44122600	10.38753300
O	17.78970700	14.03364800	11.61020000
C	7.54202100	7.92574700	14.51360600
C	7.35410700	6.70420300	13.78710700
C	6.10126900	6.34985300	13.32721700
C	5.00480000	7.19875600	13.57127800
C	5.15894400	8.40655800	14.27952500
C	6.40507400	8.77322300	14.74626200
C	9.13896200	9.36791500	15.58397200
C	9.36991500	9.29336300	17.06780100
C	8.49982100	8.52943100	17.84966600
C	8.62370500	8.58373800	19.23707700
C	9.61125400	9.38843200	19.79284700
C	10.46592600	10.10397200	18.94476000
C	11.54816500	10.97172400	19.46658900
C	11.76378000	11.28107900	20.81442600
C	12.82321700	12.12640400	21.14739000
C	13.65116000	12.64403600	20.14757500
C	13.38392600	12.29180800	18.82843100
C	14.19871400	12.75736800	17.64786800
C	14.41969100	12.58123700	15.27734600
C	14.45951900	13.91757900	14.83938300
C	15.03792600	14.24118300	13.61428600
C	15.57267100	13.22733500	12.80979800
C	15.53630000	11.89513900	13.22800900
C	14.96166900	11.57644100	14.45975800
H	11.09415200	10.52520600	14.46201000
H	13.00215400	12.37961900	22.18766100
H	14.48263000	13.30051300	20.37608700
H	11.12096700	10.88000800	21.58862800
H	9.72392700	9.45149600	20.86847600
H	7.95643100	8.01186200	19.87371000
H	7.73144700	7.92408800	17.38282400
H	6.53276400	9.70068100	15.29574700
H	8.21457200	6.06599400	13.61308300
H	5.96257500	5.42519100	12.77735400
H	4.30134900	9.04713000	14.45577900
H	14.92469100	10.54349500	14.79347100
H	14.02750600	14.69586500	15.46062700
H	15.06014100	15.27439700	13.28230400
H	15.94753400	11.11037800	12.60154000
H	12.51386600	11.07378000	14.09235000

[(L4)Cu(OH)]$^-$ (d) E= -2812.397681 H

Cu	11.70722400	10.75989200	16.83312900
S	5.16694700	6.16492900	11.82874000
S	15.11515900	13.69338500	11.00189600
N	8.60453500	9.20087800	15.61383100
N	9.95414200	10.48477800	18.11917500
N	12.45688600	11.14672600	18.63104700
N	13.76102000	11.40490600	16.33239600
O	7.62730300	11.26730300	15.91853600
O	15.66757500	12.07214000	17.46218700
O	11.24062000	10.34488500	15.08157400
O	4.39953900	7.20787000	11.07570600
O	4.29151400	5.32092900	12.70423600
O	6.08400100	5.36386200	10.96291000
O	14.16486500	14.83710200	10.82297700
O	14.92362900	12.59955000	10.00434500
O	16.53937600	14.13688600	11.13411800
C	7.79171200	8.55136700	14.76657100
C	8.39638000	7.66835400	13.81560000
C	7.61540000	6.95518300	12.92444300
C	6.21794500	7.09019900	12.97121700
C	5.59331700	7.93902900	13.91062500
C	6.36002900	8.66144500	14.79892900
C	8.23513800	10.28478200	16.35531100
C	8.66963200	10.26218600	17.79932100
C	7.66971800	10.06747100	18.75636500
C	8.02224400	10.09919600	20.10150100
C	9.34567900	10.36701200	20.44570000
C	10.28558600	10.57161100	19.43343000
C	11.69218900	10.93429100	19.71580400
C	12.23595100	11.08321500	20.99900500
C	13.56877000	11.46572800	21.13245800
C	14.34290900	11.69232500	19.99274400
C	13.74305100	11.51371100	18.75042200
C	14.48110200	11.72964300	17.47068900
C	14.06814600	11.95948700	15.13451400
C	14.59461900	13.28815600	15.00708400
C	14.89621300	13.79309700	13.76246600
C	14.69561000	12.99294500	12.61499100
C	14.17065900	11.69500200	12.71084100
C	13.84013200	11.18577600	13.95383900
H	14.00081600	11.58666600	22.12058300
H	15.38039000	11.99879100	20.05509000
H	11.63374900	10.90199000	21.88071000
H	9.63385700	10.42455600	21.48806400
H	7.27692200	9.93623900	20.87331500
H	6.64675200	9.89472100	18.43867500
H	5.88832900	9.30731300	15.53114700
H	9.47851100	7.58428900	13.80158700

H	8.07403700	6.29581200	12.19621400
H	4.51134800	8.02153500	13.93843200
H	13.43853800	10.18713300	14.06065000
H	14.72772300	13.89925100	15.89209000
H	15.28275100	14.80239800	13.66495500
H	14.02948200	11.09724200	11.81751600

[(L4)Cu(OH)]- (q) E= -2812.400366 H

Cu	12.34102300	10.45297100	16.68810100
S	3.65335300	6.64766700	12.67674600
S	15.32798600	14.58617300	11.30451800
N	8.86485700	8.02824500	15.18158300
N	10.42766300	10.00392800	17.65378900
N	12.58846700	11.22281300	18.51198000
N	14.07576600	11.80143600	16.41842900
O	9.59041100	10.18587000	14.89851200
O	15.82019500	12.46227700	17.81495600
O	12.54833400	9.36195000	15.17610500
O	3.60665600	7.46003700	11.41705100
O	2.61210600	7.06354100	13.67024600
O	3.67585700	5.17407100	12.42695100
O	14.52129900	15.84655800	11.38124300
O	14.89586300	13.67287800	10.20493100
O	16.80358000	14.83594400	11.32673400
C	7.68719300	7.76330400	14.60643900
C	7.48539300	6.45658100	14.05460100
C	6.27457100	6.11556600	13.48009400
C	5.23574100	7.06085000	13.44249100
C	5.40462300	8.35605000	13.97590300
C	6.60698100	8.70979000	14.55082600
C	9.25643800	9.26046800	15.64105500
C	9.35009500	9.38771800	17.13948800
C	8.28500600	8.95040700	17.93081700
C	8.32898100	9.19561100	19.30231100
C	9.43164500	9.85511000	19.83687500
C	10.47225700	10.24119300	18.98485000
C	11.68479000	10.94542300	19.46548100
C	11.92567900	11.32060300	20.79406600
C	13.11788000	11.97129500	21.10884600
C	14.05035100	12.24033900	20.10440900
C	13.74120000	11.84444000	18.80637400
C	14.65771100	12.09521600	17.65187100
C	14.41292600	12.45764500	15.28858200
C	15.14225600	13.69796100	15.26376800
C	15.40912000	14.31270600	14.06125600
C	14.97474300	13.72333100	12.85286000
C	14.24704200	12.52175500	12.84943300
C	13.95480900	11.90141200	14.04838900
H	13.31822200	12.26667800	22.13365000
H	14.98756700	12.74368900	20.31154700
H	11.20250000	11.10852300	21.57190600
H	9.48133500	10.06103000	20.89913400
H	7.51292300	8.88112000	19.94483100
H	7.43702300	8.44831000	17.47857600
H	6.74165000	9.70095500	14.97262100
H	8.30121600	5.74199000	14.09925100
H	6.12337300	5.12493200	13.06585400
H	4.58871500	9.07127500	13.94277700
H	13.38643900	10.97664500	14.08673000
H	15.45480000	14.16391100	16.18891100
H	15.94779800	15.25469200	14.04555700
H	13.91405500	12.08964600	11.91245400

[(L4)Cu(OH$_2$)] (d) E= -2812.847401 H

Cu	12.11523300	11.04334900	16.60515200
S	3.34888400	6.69648000	13.15744900
S	16.35626200	13.66491900	11.25941700
N	8.75273500	8.26897100	14.87817800
N	10.33112900	10.03876600	17.57152300
N	12.33495000	11.46571400	18.51587600
N	13.79799800	12.19829700	16.49778500
O	9.37497500	10.44303400	14.92478000
O	15.13509600	13.54020400	17.85162400
O	12.10326100	10.43824200	14.67404000
O	2.73332000	7.92084000	12.57859200
O	2.79257800	6.30321200	14.48645500
O	3.50877400	5.57239700	12.20225200
O	15.45794300	14.68961500	10.63072000
O	16.45247400	12.40230900	10.45653100
O	17.70542800	14.22867300	11.60799500
C	7.55298500	7.99732900	14.47650400
C	7.37432000	6.75331900	13.72446500
C	6.12366900	6.37263800	13.32409000
C	5.02549000	7.21361200	13.62806400
C	5.15525300	8.44423900	14.33513800
C	6.38807900	8.84306600	14.75596000
C	9.13831500	9.42280100	15.56041100
C	9.34749000	9.29138400	17.03408500
C	8.48640600	8.50266000	17.79934600
C	8.62482200	8.52437200	19.18818700
C	9.60577500	9.32704100	19.75347600
C	10.44975800	10.07245700	18.91500500
C	11.51949800	10.94864400	19.44749700
C	11.71117700	11.26598800	20.79653800
C	12.76002800	12.12039700	21.14127500
C	13.59906300	12.63732400	20.15116500
C	13.35318800	12.27813200	18.82912700
C	14.18216400	12.74584100	17.65931400
C	14.43438900	12.56562300	15.29179300
C	14.43525900	13.89798700	14.83938800
C	15.01041700	14.22471100	13.61425000
C	15.58092400	13.21828000	12.82393500
C	15.58898700	11.89188800	13.25912500
C	15.01777800	11.56968600	14.49249800
H	11.20759400	10.59117100	14.31338500
H	12.92112100	12.38097800	22.18258000
H	14.42291400	13.30035300	20.38817200
H	11.05768800	10.86611400	21.56250200
H	9.72607200	9.36630900	20.82931800
H	7.96930900	7.92954600	19.81547900
H	7.71414800	7.90253000	17.33272100
H	6.52579600	9.77292200	15.29864000
H	8.25430900	6.15293000	13.51741900
H	5.96381900	5.45208100	12.77488600
H	4.27766500	9.05068200	14.53002900
H	15.01798500	10.54071600	14.84124800
H	13.97434800	14.66879700	15.44919300
H	15.00036800	15.25423800	13.26985100
H	16.03080800	11.11436500	12.64496800
H	12.71679400	10.94193100	14.10665300

[(L4)Cu(OH$_2$)] (q) E= -2812.858131 H

Cu	12.04526300	11.11923600	16.58068500
S	3.38303900	6.60588700	12.83153300
S	16.55370700	13.55517100	11.26740500
N	8.65813200	8.08936900	15.13269600
N	10.33454600	10.03665400	17.63356900
N	12.35277400	11.55304000	18.47932200
N	13.81174300	12.33117400	16.41763200
O	9.07143700	10.33482700	15.05704000
O	15.16491800	13.60484900	17.83034100
O	11.66232000	10.83679000	14.63179000
O	2.63022800	7.87507900	12.58869200
O	2.77092900	5.76151800	13.90950400
O	3.67000500	5.83573400	11.58196700
O	16.09160400	14.91774100	10.86267000
O	16.15227000	12.47832600	10.31531200
O	18.00471400	13.51332600	11.63720400
C	7.46262100	7.81202500	14.60144900

C	7.32706800	6.61084300	13.83137500
C	6.10794400	6.26610100	13.28227900
C	4.99260700	7.10051000	13.48802900
C	5.09228900	8.28032600	14.25135100
C	6.30497500	8.63859600	14.80408100
C	9.00956000	9.27604800	15.70338900
C	9.37260100	9.22088900	17.15950700
C	8.59969600	8.40345500	17.98851400
C	8.80712400	8.45378600	19.36501700
C	9.77454800	9.31702900	19.86397600
C	10.52595400	10.09163900	18.97034900
C	11.57456900	11.02545800	19.43886700
C	11.77683900	11.37952700	20.78022200
C	12.79080000	12.27830700	21.10257900
C	13.59465600	12.80616500	20.09142300
C	13.34141500	12.41264800	18.78161200
C	14.18624900	12.88448200	17.64335300
C	14.43861300	12.67034400	15.26499400
C	15.03556800	13.95390600	15.01901900
C	15.63883100	14.20891600	13.80702300
C	15.69377900	13.20232000	12.81868000
C	15.12645900	11.93348400	13.03783100
C	14.48842300	11.67636500	14.23242300
H	10.68022800	10.73342000	14.61051500
H	12.95419000	12.56238800	22.13698000
H	14.40025000	13.49952800	20.30189100
H	11.15056100	10.96978500	21.56268500
H	9.95203300	9.37533300	20.93064300
H	8.21944500	7.83637100	20.03645500
H	7.83891300	7.76026000	17.56077200
H	6.38978100	9.54430800	15.39579800
H	8.20218900	5.98530100	13.68695100
H	6.01059600	5.36193800	12.69109400
H	4.21946100	8.90683400	14.40109900
H	14.06033900	10.70130600	14.43370800
H	14.95646900	14.73628600	15.76136000
H	16.05604500	15.19059700	13.60824700
H	15.18859400	11.16742100	12.27311100
H	11.84709700	11.64713200	14.12003600

[(L4)Cu(O)]$^{2-}$ (d) E= -2812.858131 H

Cu	12.03666300	11.05819700	16.72376300
S	3.70216800	6.65976900	12.61428000
S	16.04303600	13.57290800	11.14742800
N	8.82933700	8.38291900	15.08660900
N	10.30186000	10.16530000	17.74929800
N	12.37530200	11.48868400	18.62493900
N	13.73488600	12.22092000	16.53057500
O	9.08459700	10.66409200	15.16732500
O	15.23553400	13.43504600	17.84677800
O	11.83684600	10.50881300	14.95997400
O	3.27591100	7.80022300	11.74043700
O	2.77894000	6.44521200	13.77568200
O	3.98929100	5.40754800	11.85005200
O	14.97904200	14.37451000	10.45057500
O	16.33106600	12.27425600	10.45561000
O	17.28210200	14.38476200	11.39939600
C	7.66175000	8.03884900	14.52909900
C	7.61718300	6.84162400	13.74415900
C	6.43328500	6.42524200	13.16172600
C	5.26612300	7.18363000	13.34929700
C	5.27623400	8.36356200	14.12296500
C	6.44941000	8.79003400	14.70806300
C	9.04399300	9.57295900	15.73899900
C	9.28987300	9.45829300	17.22069000
C	8.40321100	8.71236000	18.00060600
C	8.55321500	8.73706900	19.38664300
C	9.58357000	9.48932700	19.94307000
C	10.45106500	10.18621000	19.09472800
C	11.58835600	10.99615600	19.59299700
C	11.87180500	11.27376400	20.93470700
C	12.97801700	12.07259800	21.23105100
C	13.78041100	12.57445500	20.20254000
C	13.44369900	12.25059600	18.89051700
C	14.22373600	12.70531300	17.68079500
C	14.31654500	12.55917300	15.29367400
C	14.60762300	13.89142800	14.93771400
C	15.13537100	14.18845300	13.68408900
C	15.38022500	13.15645600	12.76838200
C	15.08816800	11.83202300	13.10107200
C	14.55854100	11.53903500	14.35911700
H	13.21247800	12.30261700	22.26578900
H	14.64490800	13.19592500	20.40497500
H	11.24856800	10.88507000	21.73104200
H	9.71772400	9.52318100	21.01775400
H	7.87446300	8.17978900	20.02437000
H	7.60429200	8.14856200	17.53136400
H	6.46196700	9.69273000	15.31031900
H	8.53217100	6.27244200	13.61385000
H	6.40320000	5.52117200	12.56395200
H	4.36305100	8.93356600	14.26344500
H	14.32761300	10.51246900	14.62819000
H	14.40667000	14.69076800	15.64285500
H	15.35174300	15.22044000	13.42315600
H	15.27283100	11.03466800	12.38921700

[(L4)Cu(O)]$^{2-}$ (q) E= -2811.926833 H

Cu	12.03879000	11.04318600	16.71851700
S	3.67326200	6.65563500	12.67367500
S	15.70291700	13.93736800	11.10578100
N	8.79708300	8.49023500	15.07179100
N	10.32109300	10.14937400	17.75992500
N	12.47267200	11.35540000	18.62221200
N	13.74646300	12.18326500	16.51202500
O	8.98509400	10.77501100	15.24197600
O	15.37101200	13.23397200	17.82109300
O	11.74327500	10.59842600	14.93881900
O	3.18376100	7.80587700	11.84697700
O	2.78896200	6.37363300	13.85073200
O	3.98402800	5.43763000	11.86488600
O	14.57812000	14.76286100	10.54471600
O	15.96114400	12.69761000	10.30308400
O	16.94309200	14.75556500	11.32857400
C	7.62899900	8.12248000	14.53080000
C	7.60848900	6.95170500	13.70603900
C	6.42600500	6.50947300	13.14003100
C	5.23622100	7.21415000	13.38501800
C	5.22267500	8.36683900	14.19861600
C	6.39398600	8.81827100	14.76831400
C	8.99072700	9.65945500	15.76612000
C	9.27687200	9.48764900	17.23535100
C	8.39954700	8.72592400	18.01068200
C	8.59747700	8.68222900	19.39010200
C	9.66611800	9.38178400	19.94373800
C	10.52018700	10.09881700	19.09921300
C	11.69998200	10.84846900	19.59394200
C	12.03708300	11.05755700	20.93575700
C	13.18298100	11.80050700	21.22709400
C	13.96948000	12.31835000	20.19388200
C	13.57514800	12.06887600	18.88169500
C	14.31804500	12.56324500	17.66419800
C	14.26417600	12.60540300	15.27245400
C	14.57848200	13.95468300	15.01257000
C	15.02447100	14.34669100	13.75352400
C	15.16295200	13.39447300	12.73501800
C	14.85126100	12.05434400	12.97355600
C	14.40510800	11.66575500	14.23799800
H	13.46005700	11.97628200	22.26192200
H	14.86268500	12.89878500	20.39355400

H	11.42448000	10.66067000	21.73640300
H	9.84201300	9.35651400	21.01264300
H	7.92855900	8.10995800	20.02494200
H	7.57432600	8.19941100	17.54369500
H	6.38875300	9.69906500	15.40230600
H	8.54029200	6.42234400	13.53345800
H	6.41418000	5.62490000	12.51309200
H	4.29254300	8.89541400	14.38312100
H	14.16152600	10.62559500	14.43329300
H	14.45745300	14.69420400	15.79667500
H	15.25745600	15.39110300	13.56727500
H	14.95690100	11.31810300	12.18388300

[(L4)Cu(HO···OH)]$^{2-}$ (d) E= -2888.362321 H

Cu	12.14961800	11.07702500	16.64937800
S	3.40297900	6.74463500	12.89334400
S	16.23704700	13.86433700	11.26794000
N	8.72938200	8.14991600	15.12976700
N	10.38626400	9.97605300	17.71053700
N	12.48173800	11.35685800	18.57992400
N	13.85191800	12.21560100	16.53089200
O	9.15980600	10.39674700	15.14573400
O	15.28757300	13.43472400	17.90279400
O	11.93084700	10.84400100	14.76188000
O	2.96972200	7.93806700	12.09805900
O	2.54328800	6.51129500	14.09914200
O	3.59623900	5.51803500	12.06109500
O	15.17374700	13.50988100	10.26471300
O	17.48578900	13.05357700	11.07505000
O	16.50147400	15.33934100	11.32338500
C	7.52046000	7.87981500	14.62496500
C	7.37002300	6.69207000	13.83874200
C	6.13838100	6.35052000	13.30950900
C	5.02835100	7.17632700	13.55243200
C	5.14328200	8.34848600	14.32985000
C	6.36492300	8.70155300	14.86202700
C	9.06884800	9.32262700	15.75221500
C	9.39562800	9.21303800	17.21489200
C	8.59161800	8.39584800	18.01393800
C	8.80528100	8.39243500	19.39119900
C	9.81758700	9.18824100	19.91477200
C	10.59920600	9.96154900	19.04472600
C	11.71308200	10.80911500	19.53578200
C	11.98875500	11.05803800	20.88599500
C	13.06425400	11.88305800	21.21342000
C	13.84943600	12.44029100	20.20172900
C	13.52476200	12.14736600	18.88074700
C	14.30480600	12.67308700	17.70111300
C	14.45635300	12.63163100	15.32484700
C	14.44896500	13.98001400	14.93128200
C	15.00791400	14.36528200	13.71239800
C	15.57960200	13.39903400	12.87908700
C	15.59184400	12.05132800	13.25612900
C	15.03142600	11.67308100	14.47476300
H	10.99778900	10.59371700	14.63222500
H	13.28780300	12.08942000	22.25549100
H	14.69342700	13.08329300	20.42195700
H	11.37676200	10.62706300	21.66884900
H	10.00547400	9.19589400	20.98147100
H	8.19263400	7.77958700	20.04460400
H	7.80717900	7.79535100	17.56667300
H	6.45992300	9.59898100	15.46485900
H	8.24297600	6.07051900	13.66617200
H	6.02764300	5.45398000	12.70997800
H	4.27333800	8.97172600	14.51198100
H	15.03549900	10.63005400	14.77645500
H	13.99850000	14.72338100	15.58208600
H	14.99811300	15.40889900	13.41565800
H	16.03629000	11.30189800	12.60841600
O	12.47125500	8.66518500	14.89034300
H	13.34763100	9.06329500	15.04723200

[(L4)Cu(HO···OH)]$^{2-}$ (q) E= -2888.363391 H

Cu	12.15960800	11.06851400	16.63283600
S	3.41191400	6.69312500	12.91624900
S	16.20786000	13.91731800	11.25708400
N	8.73797900	8.14067500	15.12619400
N	10.37887500	9.98199500	17.70239000
N	12.48733800	11.34863300	18.56736300
N	13.86039600	12.20768400	16.51681700
O	9.17241600	10.38631900	15.12787600
O	15.30402100	13.41352200	17.89148300
O	11.94084300	10.83873500	14.74744500
O	2.96600200	7.88115600	12.11983400
O	2.55936700	6.45670600	14.12654500
O	3.61027900	5.46568800	12.08644800
O	15.02971500	13.89495700	10.32196000
O	17.25426900	12.91120500	10.87833300
O	16.76358300	15.30030300	11.43457400
C	7.52912600	7.86104100	14.62656700
C	7.38169000	6.66613500	13.85069800
C	6.15004000	6.31507200	13.32776400
C	5.03715800	7.13825800	13.56658200
C	5.14918700	8.31742800	14.33372500
C	6.37080400	8.68000900	14.85960400
C	9.07251900	9.31789400	15.74304000
C	9.38662900	9.22014800	17.20906400
C	8.57525400	8.41282800	18.01072800
C	8.78493200	8.41801900	19.38867600
C	9.80174100	9.20987600	19.90957900
C	10.59009400	9.97327200	19.03652100
C	11.71040500	10.81456600	19.52444700
C	11.98252600	11.07121300	20.87404900
C	13.06318900	11.88977600	21.20028800
C	13.85558400	12.43488800	20.18773300
C	13.53324200	12.13589000	18.86748800
C	14.31655800	12.65803100	17.68836900
C	14.46319200	12.63069700	15.31232500
C	14.45383100	13.98317800	14.92906700
C	15.00786500	14.37722700	13.71242500
C	15.57662300	13.41684500	12.86800200
C	15.59104000	12.06756300	13.23391400
C	15.03628900	11.68002900	14.45392500
H	11.00743100	10.58243600	14.62702600
H	13.28414300	12.10179600	22.24178300
H	14.70240900	13.07465400	20.40650700
H	11.36321000	10.65155900	21.65734700
H	9.98807300	9.22163900	20.97650700
H	8.16677700	7.81338400	20.04455400
H	7.78933400	7.81293800	17.56527200
H	6.46376100	9.58294700	15.45454200
H	8.25670500	6.04661800	13.68129800
H	6.04141100	5.41303500	12.73614400
H	4.27719700	8.93868200	14.51281200
H	15.04414300	10.63494700	14.74840400
H	14.00541800	14.72098800	15.58756600
H	14.99645200	15.42396700	13.42532800
H	16.03326700	11.32518400	12.57729400
O	12.47888100	8.70594200	15.11597300
H	13.38186200	9.06903300	15.05827100

[(L4)Cu(HO-OH)]$^{2-}$ (d)E= -2888.386577 H

Cu	11.69255900	11.00398900	17.20650100
S	5.84031600	7.67243800	10.95600800
S	14.39134600	12.96226200	10.74119300
N	8.31722600	8.28683200	16.40086000
N	10.30356700	10.03964900	18.57670300
N	12.64383600	11.15979200	18.92284200
N	13.40497900	11.87382700	16.56806400

O	8.50126200	10.62261300	16.34022500
O	15.40987900	12.74232400	17.36987000
O	10.78552100	11.09568900	15.38770800
O	5.47993900	9.04885100	10.47334100
O	4.61994900	6.82161100	11.18274400
O	6.85355300	6.99651400	10.08037400
O	13.05323900	12.72198000	10.09954600
O	15.42942100	11.98371000	10.27870900
O	14.83635300	14.38966100	10.61700200
C	7.75702400	8.20918100	15.11370800
C	8.34348000	7.35577200	14.16239100
C	7.78266800	7.20022100	12.89329100
C	6.61442900	7.89206300	12.56402600
C	6.00333100	8.73325000	13.50353100
C	6.57230000	8.89205200	14.76391700
C	8.60189400	9.45743700	16.90194600
C	9.12556500	9.44125000	18.31764700
C	8.36041000	8.84908700	19.33043000
C	8.82144500	8.88744300	20.64150300
C	10.04573800	9.49797800	20.91085000
C	10.76601200	10.05858000	19.85670100
C	12.08389000	10.70609700	20.05163200
C	12.75938700	10.86438700	21.26837700
C	14.00587100	11.49221400	21.26398800
C	14.56560300	11.94954900	20.06550900
C	13.83842300	11.76388000	18.89507100
C	14.29261800	12.18373900	17.51854700
C	13.69422300	12.15820400	15.21332900
C	13.50410200	13.44543200	14.68936400
C	13.73938100	13.69776700	13.33761100
C	14.16324900	12.65765000	12.50344400
C	14.35370000	11.36906600	13.01093200
C	14.12354100	11.12423100	14.36582400
H	9.79849000	10.91904400	15.63791300
H	14.54331800	11.62303900	22.19809200
H	15.53346600	12.43644900	20.03592000
H	12.32883600	10.51003600	22.19719800
H	10.42997600	9.53424200	21.92320100
H	8.23983200	8.44781300	21.44594900
H	7.41527600	8.38207100	19.07819300
H	6.09931300	9.54485500	15.49096700
H	9.25097000	6.81740900	14.42205800
H	8.25389600	6.54594200	12.16717000
H	5.09188300	9.26824400	13.25236700
H	14.27513500	10.12910300	14.77463900
H	13.16919000	14.24341200	15.34560200
H	13.59435300	14.69666700	12.93854100
H	14.68572800	10.56483100	12.36235600
O	11.11298700	9.90560500	14.61316200
H	11.88823300	10.23583700	14.11471300

$[(L4)Cu(HO\text{-}OH)]^{2-}$ (q)E= -2888.316567 H

Cu	12.21782100	10.88513900	16.59602300
S	3.08955700	7.66878100	13.87610500
S	16.35475900	13.93422100	11.46497200
N	8.99030500	7.55038200	14.69820100
N	10.70481700	9.54658300	17.34120800
N	12.23570400	11.34475000	18.49012900
N	13.64694300	12.32007800	16.58024600
O	10.02685900	9.56164900	14.48319300
O	14.60963800	13.92655100	17.96523000
O	12.25722700	10.62121100	14.59302700
O	2.76308700	9.01508600	13.30287300
O	2.52828800	7.48155000	15.25563900
O	2.75748800	6.53912800	12.95278800
O	15.43190900	13.41847300	10.39562600
O	17.69533800	13.26425000	11.43510800
O	16.44789500	15.43242500	11.45820700
C	7.66032700	7.63600800	14.50546500
C	7.01575600	6.57059300	13.80173400
C	5.64533300	6.57935200	13.59867500
C	4.88137200	7.65119500	14.08596500
C	5.48525700	8.71931800	14.77832200
C	6.85087800	8.71923500	14.98565900
C	9.67543700	8.62736300	15.30219600
C	9.93645000	8.57511500	16.70448200
C	9.30645600	7.54869100	17.47410100
C	9.35296100	7.56731200	18.84663500
C	10.03087400	8.62877700	19.48925000
C	10.68960800	9.56924300	18.71419400
C	11.46751200	10.65776300	19.34531600
C	11.46862200	11.01860000	20.70221500
C	12.27226200	12.08072100	21.11364300
C	13.07367400	12.76844300	20.19019800
C	13.02918000	12.35987200	18.86481800
C	13.83970400	12.95555900	17.74142600
C	14.32846000	12.74670900	15.41784000
C	13.93331600	13.90931000	14.73633100
C	14.56475600	14.28165900	13.55081200
C	15.59760600	13.48775900	13.03802900
C	16.00356700	12.32941800	13.70561100
C	15.37246600	11.96615900	14.89742700
H	11.34383300	10.12537500	14.47743000
H	12.27695300	12.37524300	22.15860000
H	13.71095100	13.59295800	20.48860000
H	10.85052100	10.49427900	21.42100400
H	10.05864900	8.68383500	20.57060900
H	8.86510300	6.79254900	19.42992300
H	8.76594000	6.76227300	16.95982300
H	7.31975200	9.53809900	15.52201600
H	7.62306100	5.75016000	13.43154700
H	5.16368500	5.76677800	13.06601700
H	4.88073800	9.54192700	15.14806100
H	15.68875800	11.07428800	15.43164000
H	13.12433300	14.51196700	15.13888800
H	14.25270700	15.18225400	13.03089000
H	16.80871900	11.72031200	13.30814100
O	13.22464700	9.58706500	14.25196800
H	13.99429500	10.14907500	14.02922400

$[(L4)Cu(OH)_2]^{4-}$ (d) E= -2888.732095 H

Cu	11.10198700	10.48303400	16.69061100
S	5.41432900	6.51523000	11.21341700
S	16.12547900	16.35132500	11.76030200
N	7.95998600	8.03970100	16.44272900
N	9.88080800	10.06375700	18.32922500
N	12.19141400	11.43247300	18.36488800
N	13.77479500	13.83128300	16.68663700
O	7.85266900	10.37656600	16.20891500
O	14.35276300	11.59490900	16.25813600
O	11.04124500	8.71346000	15.84385100
O	4.64212900	7.69998100	10.70744500
O	4.50962600	5.36422300	11.56665600
O	6.51762500	6.09944900	10.28389700
O	16.85491600	17.56108800	12.28240000
O	14.98533500	16.72538900	10.85831200
O	17.06394400	15.36063300	11.13180100
C	7.37456400	7.76914900	15.19843700
C	8.00436700	6.84585600	14.33900400
C	7.42971000	6.48296500	13.12147000
C	6.20304000	7.03761200	12.74049100
C	5.55129300	7.94749100	13.58240300
C	6.13448100	8.31250400	14.79381800
C	8.10307200	9.29200500	16.83313000
C	8.68571500	9.45147300	18.22130700
C	7.96849400	9.04313100	19.34838800
C	8.50136700	9.29417300	20.61034400
C	9.72543400	9.95149800	20.71692200
C	10.39901800	10.32968900	19.55038700
C	11.68681800	11.07128300	19.57016900

C	12.33067100	11.40906200	20.76503700
C	13.50405100	12.15932600	20.71426100
C	13.99816700	12.55851100	19.47687200
C	13.31794000	12.16751000	18.31819100
C	13.87590200	12.54915200	16.96557700
C	14.32741700	14.33036200	15.50020300
C	15.66097200	14.07952700	15.10382100
C	16.19588900	14.67751100	13.96529900
C	15.40385700	15.53102800	13.18625600
C	14.08209600	15.79601100	13.55872800
C	13.55560500	15.20210600	14.70551800
H	10.19367400	8.28078200	16.04913900
H	14.01820900	12.43253700	21.63103500
H	14.89920500	13.15678900	19.39325600
H	11.93076700	11.09924100	21.72234800
H	10.13642100	10.16865400	21.69473300
H	7.96607600	8.99214700	21.50570700
H	7.00995900	8.55070700	19.22674000
H	5.62666400	9.01848800	15.44333900
H	8.95830800	6.41780200	14.63575800
H	7.93706200	5.77705300	12.47121100
H	4.59673700	8.37568300	13.29110800
H	12.52942200	15.40867200	14.99790500
H	16.27997500	13.42132800	15.70515800
H	17.22440800	14.47469800	13.68132400
H	13.46530500	16.45708800	12.95801200
O	11.61846800	11.44424800	15.10942200
H	12.57241500	11.61476000	15.19735500

[(L4)Cu(OH)$_2$]$^{3-}$ (s) E= -2888.530842 H

Cu	11.57908900	9.99367000	17.21417400
S	5.96521900	7.36260900	10.85370000
S	15.10351500	15.72986600	11.36662400
N	8.25794500	8.47617500	16.30058300
N	10.08491100	9.97939500	18.50709400
N	12.40297900	11.17695900	18.60667800
N	13.34411900	13.37500700	16.61355700
O	7.78444100	10.77439600	16.50887300
O	15.04301000	11.75458600	16.79509400
O	11.05736800	8.48125800	16.31449300
O	5.63052300	8.68930400	10.23062000
O	4.73333300	6.53496400	11.10077000
O	7.01531200	6.61277500	10.08906500
O	15.67609400	17.08236900	11.68928700
O	13.88695200	15.81093000	10.49201900
O	16.14778600	14.79819800	10.81463300
C	7.71181500	8.29913800	15.01997300
C	8.41416500	7.48040100	14.11385200
C	7.90235100	7.19893800	12.84730500
C	6.66545000	7.72755200	12.46875100
C	5.94132000	8.53239500	13.35852800
C	6.46006300	8.81801500	14.61915100
C	8.21706800	9.64995000	16.90160900
C	8.79622400	9.64747600	18.30609200
C	7.94306800	9.41583500	19.39015600
C	8.43815400	9.52708400	20.68408600
C	9.75317900	9.95282400	20.87913400
C	10.55330300	10.20200800	19.76806200
C	11.86794500	10.85954200	19.82455000
C	12.47890000	11.26674400	21.00656000
C	13.62862900	12.05519500	20.93831500
C	14.11183300	12.44247300	19.69528200
C	13.47214300	11.98856800	18.53611900
C	14.01528800	12.41362300	17.19043200
C	13.79451100	13.88168100	15.38278200
C	15.09888700	14.39209000	15.20378200
C	15.48355900	14.95906400	13.99225400
C	14.56985300	15.02267100	12.93101300
C	13.27384000	14.52544200	13.08747300
C	12.89049200	13.96671000	14.30856400
H	10.07295300	8.38519100	16.36719000
H	14.11913800	12.38371100	21.84925300
H	14.97385400	13.09390400	19.59748300
H	12.05868500	10.99317000	21.96664700
H	10.13214900	10.11488200	21.88050400
H	7.80043100	9.32273900	21.53840800
H	6.90845600	9.15195200	19.19772600
H	5.89185400	9.43587200	15.30603100
H	9.37219600	7.06469300	14.41402100
H	8.46245000	6.57259200	12.16088700
H	4.97512100	8.93821500	13.07145300
H	11.88027300	13.58728700	14.43668200
H	15.80401600	14.35028500	16.02839500
H	16.49028700	15.35100000	13.87675700
H	12.56610400	14.57704900	12.26671100
O	12.93755500	10.00935400	15.98348000
H	13.73510200	10.44153600	16.35289400

[(L4)Cu(OH)$_2$]$^{3-}$ (t) E= -2888.548855 H

Cu	11.87343300	9.68925500	16.93038300
S	5.42032500	6.91077600	11.14670100
S	14.69641500	16.91351200	12.37387900
N	7.92744600	8.49893600	16.37716400
N	10.19929500	10.21727000	18.19612700
N	12.70166700	11.06862700	18.28152400
N	13.76266600	12.18744900	15.86614700
O	8.52961500	10.70093300	15.81696300
O	15.80584400	11.54531600	16.71887500
O	11.05948200	8.39313000	15.76211400
O	5.26023000	8.14960300	10.31126900
O	4.08932600	6.32235400	11.53337600
O	6.32626000	5.89584700	10.51254900
O	14.21582300	18.07686400	13.18902200
O	13.89393000	16.69489700	11.13178600
O	16.17333900	16.95255200	12.12713100
C	7.37578900	8.20124200	15.12268300
C	7.75292500	7.00135800	14.48694300
C	7.18499200	6.61518600	13.27286700
C	6.21722500	7.42613400	12.67204600
C	5.81559300	8.61770900	13.29032600
C	6.39332300	9.00279100	14.49769800
C	8.40462200	9.70823600	16.60751900
C	8.89650700	9.93506000	18.02095000
C	7.99149100	9.95472700	19.08829800
C	8.45294800	10.29096600	20.35717400
C	9.79614700	10.62740000	20.52783800
C	10.64593100	10.59000100	19.41973200
C	12.06083700	11.04192100	19.46985500
C	12.69018600	11.48143700	20.64317900
C	13.99345600	11.96396600	20.57446800
C	14.63174600	12.03387200	19.33607700
C	13.94425600	11.58152100	18.20959500
C	14.59730800	11.76532000	16.86796200
C	14.02675700	13.25222600	15.09459800
C	15.03210000	14.23185500	15.40355400
C	15.22127400	15.31673600	14.57393700
C	14.42688500	15.46435500	13.41757600
C	13.42867200	14.53034700	13.09556300
C	13.22299500	13.44167600	13.92441200
H	10.10595900	8.31625600	15.93239600
H	14.49922300	12.30119400	21.47374300
H	15.63040900	12.44265900	19.23114300
H	12.17574200	11.45004900	21.59536800
H	10.15860400	10.92575100	21.50381400
H	7.77423100	10.31251500	21.20458000
H	6.94742900	9.72115100	18.90996500
H	6.07878700	9.92634000	14.97321300
H	8.50565500	6.37282800	14.95548700
H	7.49782600	5.69173600	12.79614400
H	5.05986100	9.24731500	12.82937100

H	12.45674500	12.70750000	13.69707200
H	15.63550300	14.11743800	16.29711800
H	15.98101800	16.05449100	14.81210200
H	12.82673400	14.66310500	12.20347900
O	13.62049600	8.93365600	16.45287000
H	14.28198000	9.15396300	17.12788000

[(L4)Cu(OH)$_2$]$^{2-}$ (dos) E= -2888.350618 H

Cu	11.61907400	9.73420700	17.20824900
S	5.74976600	7.07951000	11.05778500
S	15.01869500	16.56538500	12.02514300
N	8.36072300	8.65928500	16.23878400
N	10.08443400	10.10199400	18.42596800
N	12.52301200	11.02839800	18.40496100
N	13.45558300	12.39256300	15.97078100
O	7.43991100	10.79643200	16.54532800
O	15.47713800	11.46766100	16.62508300
O	11.00114200	8.21156900	16.43265200
O	5.66404900	8.32604900	10.21817400
O	4.39017100	6.54814600	11.41275200
O	6.63741800	6.03944100	10.43960600
O	15.27448600	17.80586600	12.82512000
O	13.87854600	16.70264400	11.06902600
O	16.26176500	16.01956900	11.39016500
C	7.72840800	8.37064300	15.01953200
C	8.47516900	7.71227900	14.02218100
C	7.88381400	7.31381700	12.82386800
C	6.52354200	7.55422800	12.60941100
C	5.75997100	8.20118800	13.58922100
C	6.35639800	8.60630500	14.78092200
C	8.13205900	9.78900800	16.88036300
C	8.76285300	9.91204600	18.25816500
C	7.89091600	9.96189500	19.35528800
C	8.39511200	10.20353300	20.62525200
C	9.75322300	10.49592700	20.77282000
C	10.57137800	10.46997600	19.64982300
C	11.96080700	10.96158700	19.63775300
C	12.64698700	11.42289400	20.76406400
C	13.91810200	11.96706300	20.60313900
C	14.46405400	12.06971800	19.32412400
C	13.73275400	11.59049800	18.23677800
C	14.31069800	11.80197000	16.86039700
C	13.85278100	13.34533500	15.11044200
C	15.06849100	14.09962700	15.24824000
C	15.39228500	15.06632800	14.32048300
C	14.52754400	15.31622900	13.23388500
C	13.32208300	14.61143300	13.08433300
C	12.97926500	13.64829300	14.01659200
H	10.00277500	8.32332800	16.37378800
H	14.47046900	12.32556100	21.46575100
H	15.43270800	12.52622000	19.15365100
H	12.19617000	11.36002900	21.74690500
H	10.15366500	10.77083100	21.74093200
H	7.73815000	10.21117000	21.48925200
H	6.83018000	9.80986400	19.18656400
H	5.75597400	9.09146500	15.54282700
H	9.52791600	7.50882500	14.19702100
H	8.47573200	6.80714400	12.06844200
H	4.70053500	8.37963900	13.42991700
H	12.05240300	13.09138500	13.92411500
H	15.72379400	13.91297900	16.09085300
H	16.30943800	15.63650200	14.42991500
H	12.66726100	14.82444600	12.24676600
O	13.15938700	9.42075600	16.30484000
H	12.93409600	8.66063000	15.73741300

[(L4)Cu(OH)$_2$]$^{2-}$ (q) E= -2888.364446 H

Cu	12.65833500	9.44445500	16.88617900
S	3.67170200	7.34682700	12.53725600
S	14.94986600	17.39960700	12.57841700
N	8.88564500	7.53531800	15.38741400
N	10.69449300	9.73529500	17.56343500
N	12.78443900	11.23404400	18.14092300
N	14.09333700	12.61869900	16.01467600
O	10.32257000	9.22305500	14.80954700
O	15.99041300	12.31884900	17.29098700
O	12.43957200	7.52273000	16.60733400
O	3.75802400	8.34066700	11.42086400
O	2.67963400	7.74459000	13.58981700
O	3.49248300	5.93927800	12.06447100
O	14.19501700	18.49260000	13.27363400
O	14.39475700	17.06450300	11.23159700
O	16.42914400	17.63631100	12.56725500
C	7.72862700	7.53530300	14.71695700
C	7.13134100	6.27120600	14.40322800
C	5.92178500	6.21149600	13.73587400
C	5.27797000	7.40334500	13.36126600
C	5.83914200	8.66274200	13.65793900
C	7.04486200	8.73685300	14.32356500
C	9.62166400	8.66551600	15.65641400
C	9.58286900	9.15066000	17.08239700
C	8.39885600	9.09025600	17.81742700
C	8.36847200	9.68339500	19.08065000
C	9.50596300	10.32522800	19.55973200
C	10.66450000	10.33353600	18.77097200
C	11.90276000	11.06318800	19.14918100
C	12.13077500	11.59740300	20.42288800
C	13.29065700	12.33780900	20.64357100
C	14.17089300	12.56146600	19.58536700
C	13.87203000	11.99557000	18.34379000
C	14.75740800	12.32973900	17.17608200
C	14.33810500	13.70457400	15.26709600
C	15.07247700	14.84800000	15.73466300
C	15.24949600	15.94121500	14.91317700
C	14.70969000	15.93592400	13.60955600
C	13.97969100	14.83739600	13.12778200
C	13.78670000	13.73822600	13.94567500
H	11.57265100	7.23467700	16.93686400
H	13.49491300	12.75267100	21.62557400
H	15.06051700	13.17038000	19.70246400
H	11.42814500	11.43513000	21.23147800
H	9.48443100	10.82307700	20.52150100
H	7.46057800	9.66263900	19.67486400
H	7.51854500	8.61217600	17.40345500
H	7.48332700	9.70226800	14.55627800
H	7.64886800	5.36494000	14.70174300
H	5.47319900	5.25351100	13.49726400
H	5.32615600	9.57231000	13.36184000
H	13.22349400	12.87858300	13.59674800
H	15.47593400	14.85110700	16.74123600
H	15.80179800	16.80419700	15.27186600
H	13.57124600	14.85398400	12.12355800
O	14.48845000	9.36132900	16.28898400
H	15.07684100	9.74145800	16.96083500

[(L4)Cu(OH)(O)]$^{3-}$ (d) E= -2887.952624 H

Cu	11.47604200	10.02067900	16.42655100
S	5.08513200	5.60568400	11.85292000
S	15.84554200	17.50531100	14.44828800
N	8.03985700	8.36158200	16.31117600
N	9.99277700	10.45162200	17.78839900
N	12.46460500	11.13865900	17.59390500
N	13.47716900	12.04448700	14.90510300
O	7.59994800	10.59374200	15.70551600
O	15.23079900	10.55414500	15.49892300
O	10.76749200	8.81393600	15.24477900

O	4.35144300	6.66410800	11.07908400
O	4.13877300	4.65601600	12.53757700
O	6.11113000	4.88790700	11.02623600
O	15.70217500	18.04053100	15.84429700
O	15.01438000	18.25020700	13.45098600
O	17.28033800	17.37055100	14.03211800
C	7.34554500	7.78507700	15.23414900
C	7.96210400	6.74825400	14.50692100
C	7.29271600	6.09479800	13.47168500
C	5.98331500	6.46395200	13.15161400
C	5.34119700	7.47784700	13.87498900
C	6.01682600	8.13242000	14.90137800
C	8.04530200	9.67226900	16.45925900
C	8.68089200	10.16725000	17.74299800
C	7.84271700	10.41941700	18.83848500
C	8.37317200	10.98081300	19.99200500
C	9.72885300	11.31715600	20.02242400
C	10.51154200	11.05142200	18.90574500
C	11.92840400	11.42699900	18.79558600
C	12.72992300	12.03620300	19.76040700
C	14.06483200	12.30665300	19.44165200
C	14.57661100	11.99844500	18.17783600
C	13.72576900	11.40509700	17.24899900
C	14.00783300	10.97205500	15.78960900
C	14.12228700	13.22449900	14.79030100
C	15.39970500	13.56831300	15.35020700
C	15.91118600	14.84600600	15.21720700
C	15.18762000	15.82847000	14.51540100
C	13.95781400	15.52056700	13.91645900
C	13.44310700	14.23932000	14.03907800
H	9.91183000	8.53085800	15.62893800
H	14.70913900	12.76070800	20.18830700
H	15.61238900	12.19544000	17.92940800
H	12.33302200	12.28104000	20.73847700
H	10.16437900	11.78740700	20.89633300
H	7.74291900	11.17323400	20.85462500
H	6.78890000	10.17235700	18.76042600
H	5.51547400	8.91654500	15.45964100
H	8.97948500	6.45945400	14.75687700
H	7.78991600	5.30715400	12.91485200
H	4.32050300	7.76188700	13.63523000
H	12.48975100	13.98947100	13.58351300
H	15.98411100	12.81255900	15.85592100
H	16.87682000	15.08671500	15.65203400
H	13.41515400	16.27931600	13.36296800
O	13.12677800	9.82638400	15.59985400

[(L4)Cu(OH)(O)]$^{3-}$ (q) E= -2887.874387 H

Cu	11.79698800	9.83972100	16.88437800
S	5.34385800	6.73498600	11.23307000
S	15.00328900	16.90889500	12.39926400
N	8.04324400	8.39767000	16.34455800
N	10.14305000	10.23119700	18.18239300
N	12.64868600	11.11014300	18.29459300
N	13.77054100	12.26981700	15.91340900
O	8.24371200	10.69506000	15.88538200
O	15.77265900	11.49807300	16.76249600
O	11.05881300	8.74681200	15.47201500
O	5.16166900	7.97008000	10.39481400
O	4.02296100	6.15492400	11.66157600
O	6.22979300	5.71700300	10.57733800
O	14.79158700	18.10688300	13.27545700
O	14.04917300	16.84387900	11.25040300
O	16.43470500	16.74183600	11.98790900
C	7.43819000	8.07995600	15.11944400
C	7.95609200	7.01675900	14.35500700
C	7.33985400	6.60971900	13.17043200
C	6.18256400	7.25927100	12.73287300
C	5.64139100	8.31383800	13.48211100
C	6.26477800	8.72012500	14.65819300
C	8.33406000	9.65469600	16.61795200
C	8.85082000	9.89797700	18.01941900
C	7.95315300	9.87817900	19.09359600
C	8.40897400	10.22537000	20.36028300
C	9.74163400	10.60492200	20.52316800
C	10.58596900	10.60276700	19.41123800
C	11.99652600	11.06704900	19.47561500
C	12.61980300	11.48747800	20.65933100
C	13.93055800	11.95195500	20.60901100
C	14.58597900	12.02125700	19.37968000
C	13.90259400	11.59820000	18.23957200
C	14.57660400	11.78124500	16.90746000
C	14.10660000	13.30919500	15.13503700
C	15.18783100	14.21018100	15.42546800
C	15.44208800	15.27857900	14.59199900
C	14.63901500	15.48656400	13.45063100
C	13.57051300	14.62850900	13.14506100
C	13.30028800	13.55685500	13.97772000
H	10.16805000	8.45204800	15.73000100
H	14.43111300	12.27220100	21.51736100
H	15.59502100	12.40819700	19.29256900
H	12.09778200	11.45217400	21.60708300
H	10.10070700	10.90377000	21.49999800
H	7.73639000	10.21742300	21.21276200
H	6.91820400	9.60262500	18.92055200
H	5.84090200	9.53325000	15.23942100
H	8.85420100	6.50957200	14.69746100
H	7.75913000	5.79240400	12.59290600
H	4.73829900	8.81863900	13.14980300
H	12.47767100	12.88162100	13.76518000
H	15.79560500	14.05170800	16.30915700
H	16.25661700	15.95976100	14.81760700
H	12.96273500	14.80785500	12.26523600
O	13.52612600	9.12716700	16.44221400

[(L4)Cu(OH)(HO···OH)]$^{3-}$ (d) E=-2964.313943 H

Cu	12.36614300	10.13490200	16.72694500
S	3.51221600	6.99501800	12.81703300
S	15.97506700	16.41137700	12.04423800
N	8.93562200	7.87869400	15.08736800
N	10.52353800	9.82461800	17.63767600
N	12.48474500	11.39406300	18.45037300
N	13.54894200	13.56811300	16.75299500
O	9.83056200	9.97916000	14.89491500
O	15.50781700	12.60874900	17.63375600
O	12.29826700	8.33934000	15.84566200
O	3.16011500	8.23653700	12.05756700
O	2.65301700	6.79405500	14.03028400
O	3.59850800	5.77692700	11.95312300
O	15.50029700	17.83425900	12.17882700
O	15.44381800	15.74940100	10.80694100
O	17.47016000	16.31030400	12.16104900
C	7.71714100	7.73342500	14.55676100
C	7.38766700	6.47058700	13.96423000
C	6.13277500	6.25182400	13.42660500
C	5.17491100	7.27991300	13.46169500
C	5.46821900	8.53293700	14.03798300
C	6.71618600	8.76455800	14.57781000
C	9.42679800	9.05469600	15.60353800
C	9.50096400	9.12907700	17.10649400
C	8.48323300	8.59178100	17.89318800
C	8.52906200	8.80244500	19.27328400
C	9.56812100	9.54926200	19.81619100
C	10.56119300	10.05694100	18.96540800
C	11.67110600	10.92659900	19.42959500
C	11.83982900	11.30694100	20.76134000
C	12.87024100	12.19367700	21.08204300
C	13.67397300	12.70004900	20.06654900
C	13.43778500	12.29214600	18.74811600

C	14.25454900	12.87729700	17.61964800
C	14.18753400	14.18694200	15.67026900
C	15.27412400	15.07814300	15.82122200
C	15.81916100	15.73650400	14.72181200
C	15.29372400	15.51158400	13.44209300
C	14.21655100	14.63767300	13.26862100
C	13.67066800	13.98594300	14.37507100
H	11.91163700	7.70500300	16.47388200
H	13.02499400	12.49749700	22.11292600
H	14.46529000	13.41330100	20.27203900
H	11.18758200	10.93016800	21.53960500
H	9.60236000	9.73578800	20.88231400
H	7.75267500	8.39856000	19.91525600
H	7.66819700	8.03961200	17.43954700
H	6.94826700	9.72686500	15.02310100
H	8.14397400	5.69206100	13.94821300
H	5.88840500	5.29620000	12.97587600
H	4.71611500	9.31530300	14.05535700
H	12.83251100	13.30705700	14.24252900
H	15.68058100	15.25687800	16.81198700
H	16.65256600	16.41941800	14.86099800
H	13.80747800	14.46381400	12.27863200
O	14.07538000	10.37269300	15.86420200
H	14.63009300	11.02419800	16.32355300
O	14.43891100	7.71379000	16.48896300
H	14.45336100	8.68726800	16.27333100

[(L4)Cu(OH)(HO···OH)]$^{3-}$ (q) E=-2964.314770 H

Cu	12.36723500	10.13364800	16.72295700
S	3.51419700	6.99340800	12.81501500
S	15.97165200	16.41581800	12.04391500
N	8.93643600	7.87715900	15.08812200
N	10.52498000	9.82415500	17.63679700
N	12.48458500	11.39688200	18.44849700
N	13.55010200	13.56763600	16.75199300
O	9.83250400	9.97677000	14.89321100
O	15.51024400	12.61127600	17.63308700
O	12.29047000	8.33881600	15.84014900
O	3.16228400	8.23490400	12.05542800
O	2.65457700	6.79236200	14.02795500
O	3.60090400	5.77532500	11.95113500
O	15.49609700	17.83831300	12.17980800
O	15.44006300	15.75435500	10.80648600
O	17.46690600	16.31563300	12.15969300
C	7.71824100	7.73181200	14.55685000
C	7.38889600	6.46880400	13.96462200
C	6.13432300	6.25010700	13.42623300
C	5.17662300	7.27839300	13.46032700
C	5.46981700	8.53158000	14.03629700
C	6.71747600	8.76315000	14.57686700
C	9.42789100	9.05354400	15.60302500
C	9.50102700	9.13046700	17.10595300
C	8.48099000	8.59734100	17.89245500
C	8.52558700	8.81112400	19.27214300
C	9.56585200	9.55656300	19.81459100
C	10.56157700	10.05939900	18.96397200
C	11.67349900	10.92660300	19.42829200
C	11.84691800	11.30082500	20.76122500
C	12.87993800	12.18434500	21.08249000
C	13.68189300	12.69310500	20.06674700
C	13.44078200	12.29116300	18.74733100
C	14.25654700	12.87786000	17.61893400
C	14.18788100	14.18748400	15.66945400
C	15.27288700	15.08063800	15.82057100
C	15.81676500	15.74019400	14.72130900
C	15.29177700	15.51460100	13.44151400
C	14.21623600	14.63872800	13.26784100
C	13.67152100	13.98576800	14.37414400
H	11.91285700	7.70478000	16.47443000
H	13.03852700	12.48334200	22.11420500
H	14.47560300	13.40349900	20.27291000
H	11.19700700	10.92124700	21.54009200
H	9.59889400	9.74606300	20.88022800
H	7.74722700	8.41087700	19.91400700
H	7.66517900	8.04614500	17.43901600
H	6.94942200	9.72552800	15.02208400
H	8.14505400	5.69011700	13.94938100
H	5.89006900	5.29436600	12.97568800
H	4.71784300	9.31408500	14.05292000
H	12.83456000	13.30543800	14.24142300
H	15.67894600	15.26000100	16.81138700
H	16.64889400	16.42462900	14.86069700
H	13.80748200	14.46436300	12.27780900
O	14.07358100	10.37103200	15.85725800
H	14.62485800	11.02796400	16.31286600
O	14.40452800	7.71022700	16.52791700
H	14.44601500	8.67817700	16.29887800

[(L4)Cu(OH)(HO-OH)]$^{3-}$ (d) E= -2964.369951 H

Cu	10.76396200	10.64121400	17.08006400
S	5.39426900	7.27558700	11.07044400
S	15.75148900	15.88261600	11.78384300
N	7.70413200	7.90402500	16.58225900
N	10.04856200	9.63098700	18.61403400
N	12.12592400	11.20942200	18.69375400
N	13.42924600	13.52651100	16.80658800
O	9.34685100	9.50833000	16.12261500
O	14.52738600	11.45129500	16.75636600
O	13.29968800	9.39253100	15.49707500
O	5.86627400	8.42250700	10.22521000
O	3.90736600	7.31534800	11.30161400
O	5.84657500	5.94216300	10.55029100
O	15.73971500	17.36619300	12.03889000
O	14.84118400	15.50225300	10.65110600
O	17.14395900	15.34769600	11.61640700
C	7.24496400	7.80861000	15.26220000
C	6.93422400	6.52718800	14.76265700
C	6.40643100	6.35961000	13.48390900
C	6.16393400	7.48031600	12.68236900
C	6.44490200	8.76408900	13.16413800
C	6.98756900	8.92601900	14.43741100
C	8.64872700	8.74280800	16.91084000
C	9.01382900	8.81396600	18.36817900
C	8.38683500	8.13742200	19.41563000
C	8.86428900	8.33976300	20.71190200
C	9.94575600	9.19460800	20.94310300
C	10.53104700	9.83998500	19.85051800
C	11.69473800	10.75643400	19.90146600
C	12.32889800	11.12003900	21.08859500
C	13.43740200	11.96712200	21.02776600
C	13.89237700	12.40331800	19.78791700
C	13.21272000	11.99411000	18.63367600
C	13.77293500	12.35040600	17.27554600
C	14.00423300	13.99340800	15.61171300
C	15.39626400	13.99942300	15.38316300
C	15.92940300	14.55517300	14.22103900
C	15.07710000	15.10554900	13.25766700
C	13.69295300	15.11495600	13.46594600
C	13.16674700	14.56943800	14.63511000
H	13.77376500	10.10969800	16.00211900
H	13.94260800	12.27111600	21.93961000
H	14.76315300	13.04456800	19.69726000
H	11.97124500	10.75203900	22.04306500
H	10.31726600	9.34792200	21.94932100
H	8.39475400	7.83024300	21.54778600
H	7.55015700	7.47903900	19.21690300
H	7.20272100	9.92340100	14.80443700
H	7.11554300	5.65896500	15.39037700
H	6.18613500	5.36279900	13.11477700
H	6.24931000	9.63529800	12.54648500

H	12.09277900	14.58293500	14.80141600
H	16.06086200	13.57312000	16.12766100
H	17.00323600	14.55403100	14.06314200
H	13.02733100	15.54368400	12.72254200
O	11.12756700	11.71881300	15.54937800
H	11.71679500	12.44211400	15.82740500
O	12.79708600	10.13080200	14.33947400
H	12.11095100	10.75393900	14.77792000

$[(L4)Cu(OH)(HO\text{-}OH)]^{3-}$ (q) E= -2964.298809 H

Cu	11.51086800	10.16462100	16.45172900
S	3.52490500	7.81695300	12.98580300
S	16.48200800	16.24210100	12.22930900
N	8.99906300	7.04150200	15.20580100
N	10.33340600	9.30848400	17.73494900
N	11.96995100	11.25471500	18.29659400
N	13.28587800	13.56240200	16.56560700
O	10.53869300	8.77794700	15.17829500
O	14.92874600	12.08204100	17.35955500
O	14.78480400	9.42675600	16.83209700
O	3.59520300	9.04189800	12.12352400
O	2.65348800	8.01240000	14.19149600
O	3.19482100	6.57625900	12.21794100
O	16.81829300	17.60631500	12.76634300
O	15.60329800	16.30244500	11.01432700
O	17.71560900	15.41056300	12.00852800
C	7.77249700	7.26929000	14.70674100
C	7.11349100	6.18998900	14.03361100
C	5.84274900	6.34988800	13.50823500
C	5.19296800	7.58817800	13.63486500
C	5.81322700	8.67286300	14.28814400
C	7.07990800	8.52411200	14.81554300
C	9.67940200	8.07815700	15.86130900
C	9.51819300	8.30753200	17.25055500
C	8.61372100	7.65783800	18.13251300
C	8.57312600	8.05168100	19.45430400
C	9.41364700	9.09696900	19.92262400
C	10.28037100	9.70591200	19.02172900
C	11.21393700	10.81154700	19.34068200
C	11.33065200	11.37016700	20.61420100
C	12.25250700	12.39567100	20.82358400
C	13.04024700	12.82613100	19.76034300
C	12.87034900	12.23021800	18.50543600
C	13.77999400	12.65541400	17.37545900
C	14.08629300	14.12823900	15.55955100
C	15.39542800	14.60962700	15.78952400
C	16.10979200	15.24796300	14.78008100
C	15.53392400	15.41458600	13.51281300
C	14.24085100	14.94891200	13.26308500
C	13.52454900	14.31982700	14.28248800
H	14.85198900	10.38194900	17.11433800
H	12.35639800	12.84535000	21.80658500
H	13.77874700	13.61172200	19.88476800
H	10.71622400	11.01336300	21.43230900
H	9.36875900	9.40602700	20.95952700
H	7.88958200	7.56760100	20.14558400
H	7.96668900	6.87135400	17.75780800
H	7.56094000	9.35659600	15.31933300
H	7.63295100	5.24075500	13.94500600
H	5.35057900	5.52756000	13.00087600
H	5.29791000	9.62458500	14.37511700
H	12.51423100	13.96728200	14.09214100
H	15.84317200	14.48976300	16.77045800
H	17.11213700	15.61567900	14.98196700
H	13.79172700	15.08099700	12.28441900
O	12.56758000	10.85719600	15.02136000
H	12.82039300	11.76463900	15.26093300
O	14.78507400	9.54963100	15.37424200
H	13.90218600	10.04679800	15.20800900

Complex $[(L5)Cu]^{2-}$ and related species:

$[(L5)Cu]^{2-}$ (d) E= -2736.83333164 H

Cu	9.00221100	14.67040800	0.97793100
C	9.62349300	11.46738400	2.74352500
C	9.86844100	10.09983600	2.58627800
H	10.81957600	9.68606400	2.90219500
C	8.89403300	9.27287700	2.02284600
H	9.09567500	8.21357600	1.89190700
C	7.66833700	9.81640000	1.62731000
H	6.90450400	9.18063100	1.18764400
C	7.42760900	11.18279200	1.77494200
H	6.49064900	11.61867000	1.44346600
C	8.40067400	12.03092300	2.32351600
C	7.46764100	13.99227600	3.32339400
C	7.43171500	15.50610300	3.21901400
C	6.77756200	16.38067000	4.08607600
H	6.20967400	16.00063000	4.92776600
C	6.88332500	17.75435000	3.83301600
H	6.38682200	18.46128900	4.49073200
C	7.62236900	18.23160500	2.74288700
H	7.70753100	19.29452900	2.54421600
C	8.24815800	17.29726100	1.91573600
C	9.07757300	17.54488200	0.70552100
C	9.42559300	18.76834100	0.13105400
H	9.08802500	19.70276000	0.56646600
C	10.21873100	18.75181100	-1.02323400
H	10.50113500	19.69099700	-1.48914900
C	10.64830400	17.54269000	-1.58509800
H	11.25956900	17.51820000	-2.48013500
C	10.26130800	16.35872100	-0.95775800
C	10.59746900	14.94948700	-1.41010300
C	10.24606800	12.64255900	-0.91698800
C	11.43367800	11.99362300	-0.54855400
H	12.22204200	12.58256300	-0.09047700
C	11.58947000	10.62037800	-0.73931900
H	12.51451800	10.13717000	-0.43625100
C	10.55312900	9.86823900	-1.30063100
H	10.66191900	8.79618300	-1.43763800
C	9.36811600	10.50000400	-1.68478300
H	8.56029900	9.92656500	-2.12539800
C	9.21521300	11.87771200	-1.50057700
N	8.19439600	13.42590500	2.35536400
N	8.12588700	15.98743600	2.18984700
N	9.50963900	16.40438000	0.14035800
N	10.06474800	14.00960800	-0.62320600
O	6.86492400	13.41950300	4.26938600
O	11.29807300	14.77976800	-2.44331000
O	11.30887600	13.54394400	2.54961500
O	10.23731600	13.08986000	4.75501600
O	12.01050800	11.56778000	3.88676300
O	7.03485600	13.30700700	-0.87721400
O	6.85104400	11.57594900	-2.65423200
O	8.10486700	13.68593900	-3.09728900
S	10.88567700	12.49808600	3.53733000
S	7.68943200	12.67447800	-2.06867400

$[(L5)Cu(OH)]^{3-}$ (d) E= -2812.783247 H

Cu	7.77402000	12.64565100	1.83947300
C	9.02465500	9.12933000	1.75208800
C	9.63861200	8.14622200	0.96977900
H	10.61959900	7.77701100	1.24722900
C	8.99120700	7.64630800	-0.16236700
H	9.47796400	6.89130500	-0.77304400
C	7.72174100	8.12374900	-0.50251300
H	7.20973800	7.74047600	-1.38112600
C	7.11355400	9.11057500	0.27381900
H	6.14104000	9.50818800	0.00150000
C	7.75716400	9.63761900	1.40333200

C	6.18964000	10.51390200	2.97634000
C	5.72573200	11.79344700	3.64613000
C	4.72502800	11.90408200	4.61085000
H	4.18925700	11.02550600	4.95203300
C	4.44119800	13.17909200	5.11663300
H	3.66752500	13.29897100	5.86895100
C	5.13988300	14.30512100	4.66307900
H	4.92199100	15.29463600	5.05029200
C	6.12775300	14.11866600	3.69500700
C	6.99704300	15.14283000	3.05615600
C	7.02653200	16.51874300	3.28685400
H	6.35745600	16.97605600	4.00789700
C	7.94203900	17.28870600	2.55840100
H	7.98446600	18.36126600	2.72151100
C	8.79810700	16.69503400	1.62265700
H	9.50745700	17.28117600	1.04946000
C	8.70861400	15.31532400	1.44149600
C	9.52215500	14.48153900	0.47011000
C	9.80968700	12.27275900	-0.38371500
C	11.06487500	11.72744200	-0.07806700
H	11.57277600	12.07133600	0.81717400
C	11.63402200	10.74351200	-0.88727100
H	12.60224500	10.32550600	-0.62481100
C	10.95253400	10.28455100	-2.01856200
H	11.38236400	9.50618000	-2.64255300
C	9.70820300	10.82834000	-2.34630300
H	9.17536900	10.48456200	-3.22591900
C	9.14214000	11.82004600	-1.53966400
N	7.18689400	10.71543100	2.11062700
N	6.37644700	12.88028200	3.23529600
N	7.83584800	14.60697300	2.15395500
N	9.19487200	13.18609800	0.49705800
O	5.64089000	9.42052600	3.27850600
O	10.38238800	15.04228300	-0.25946500
O	10.09459700	11.17076700	3.13028600
O	8.91998800	9.36939100	4.39201200
O	11.13345700	8.91975200	3.33636300
O	7.79307300	14.02088000	-2.16761400
O	6.56957900	12.23415800	-0.93058100
O	7.18358400	11.89601600	-3.31843100
S	9.85145300	9.69758800	3.26046400
S	7.55305800	12.54787500	-2.01744800
O	5.40619400	15.57574600	-0.37183300
H	6.12255500	14.97649100	-0.64182300

[(L5)Cu]$^-$ (s) E= -2736.633847 H

Cu	8.98671100	14.72427000	0.96857300
C	9.83026300	11.61300700	2.84805300
C	10.16373000	10.26094300	2.85985000
H	11.05823400	9.93139300	3.37603100
C	9.35495900	9.33588400	2.18829100
H	9.62716700	8.28459300	2.19406800
C	8.21780400	9.76428200	1.49744600
H	7.60289800	9.04927100	0.95931000
C	7.87723700	11.11530200	1.48830100
H	7.00167500	11.47113400	0.95555900
C	8.66201600	12.04948500	2.17973000
C	7.52880400	13.90387700	3.20192300
C	7.44398500	15.40780000	3.16316800
C	6.76840200	16.25113200	4.03978700
H	6.19449900	15.83853600	4.86191900
C	6.86412000	17.63124400	3.82061300
H	6.35091400	18.31635000	4.48769800
C	7.61171300	18.14582200	2.75246600
H	7.68675000	19.21330600	2.57611400
C	8.25954200	17.24769700	1.90740000
C	9.08898100	17.50140800	0.71355800
C	9.47504300	18.70574300	0.12920800
H	9.15476100	19.64914300	0.55768400
C	10.28261600	18.65993300	-1.01549800
H	10.59250300	19.58880900	-1.48340000
C	10.69480500	17.43880700	-1.56448200
H	11.31937000	17.38752400	-2.44933700
C	10.27192500	16.27189200	-0.93551800
C	10.55298300	14.84688600	-1.33815500
C	9.98511500	12.57551300	-0.80010400
C	11.01901200	11.77134100	-0.29795300
H	11.77238600	12.23364000	0.33091600
C	11.07026800	10.41386000	-0.60681800
H	11.87527700	9.80118200	-0.21243900
C	10.07952600	9.84383600	-1.41133000
H	10.11175000	8.78490300	-1.65013200
C	9.02633500	10.62810000	-1.89718400
H	8.24666700	10.18021600	-2.50274000
C	8.96831000	11.98442600	-1.58576200
N	8.29080400	13.40152500	2.19116600
N	8.14551100	15.92687000	2.16273000
N	9.50620400	16.35083600	0.14586400
N	9.96456100	13.94590100	-0.50496400
O	6.96055400	13.24237100	4.08294500
O	11.24264100	14.57777800	-2.33289700
O	11.38299500	13.75552400	2.61011200
O	10.09165900	13.48226100	4.72544600
O	12.03989500	11.99853700	4.24917400
O	6.90429500	13.53834600	-0.96617100
O	6.69509100	12.02736700	-2.93573700
O	8.15672200	14.04389400	-3.06302100
S	10.91848500	12.80772500	3.67412400
S	7.57392400	12.97937700	-2.18448200

[(L5)Cu]$^-$ (t) E= -2736.632465 H

Cu	9.27508500	14.55644500	1.43686500
C	9.58154900	11.34198300	2.44569900
C	9.73419000	9.97047800	2.23650700
H	10.72834100	9.53848000	2.20812900
C	8.60633100	9.16930600	2.04904400
H	8.72294200	8.10362200	1.87637300
C	7.33493100	9.75304200	2.06677800
H	6.45269700	9.14019700	1.90305600
C	7.18437100	11.12392300	2.27514400
H	6.19867000	11.57584300	2.26104500
C	8.30573800	11.94844700	2.47551100
C	7.26586700	13.94272400	3.35986500
C	7.25974500	15.44484900	3.18186300
C	6.48135600	16.34265300	3.90693400
H	5.80069800	15.98585100	4.67141100
C	6.61584100	17.70583100	3.61787700
H	6.02691700	18.43446600	4.16629200
C	7.50235100	18.14355300	2.62924300
H	7.60177100	19.20024600	2.40988300
C	8.24713300	17.18519600	1.93316100
C	9.21489400	17.42574300	0.83230000
C	9.56464000	18.70041600	0.37785200
H	9.14751800	19.58532300	0.84395700
C	10.45911200	18.81515200	-0.68434400
H	10.75315500	19.79476600	-1.04763100
C	10.94842000	17.66042300	-1.29420100
H	11.61543000	17.70838900	-2.14704500
C	10.54014500	16.41896800	-0.80330200
C	10.96109500	15.16155100	-1.52801900
C	10.30277900	12.85072600	-1.33638400
C	11.61043400	12.32613900	-1.05305100
H	12.43749600	13.01964600	-0.96236300
C	11.81070800	10.96946600	-0.88734700
H	12.80569400	10.59180000	-0.67422800
C	10.72441700	10.08388700	-0.96028600
H	10.87537900	9.02031000	-0.80471600
C	9.42389100	10.56569800	-1.21582700
H	8.59247400	9.87172600	-1.24976100
C	9.20506300	11.91369100	-1.40955200

N	8.19873300	13.34156100	2.59740300
N	8.09009900	15.89012200	2.23414000
N	9.72258300	16.30799100	0.25860700
N	10.04336800	14.15197200	-1.47007600
O	6.47202500	13.40469300	4.16540500
O	11.97116400	15.16169100	-2.24642600
O	10.94872200	13.44959900	1.61082900
O	10.98893100	12.93047900	4.05637300
O	12.23819100	11.50491100	2.42691300
O	7.14580900	13.43156200	-0.65465200
O	6.67077800	11.25116000	-1.75155100
O	7.57930500	13.14058600	-3.09998800
S	11.04642500	12.35664400	2.68083800
S	7.51699800	12.48687900	-1.75344300

[(L5)Cu(OH)]$^{2-}$ (s) E= -2812.585527 H

Cu	7.72095700	12.66880100	1.81369500
C	9.19239200	9.23414000	2.03221300
C	9.91102700	8.18789200	1.45858600
H	10.77919400	7.78734300	1.96947500
C	9.51917500	7.66973600	0.21840100
H	10.08928500	6.85943300	-0.22638600
C	8.41463300	8.20351600	-0.45199300
H	8.12338100	7.81331100	-1.42254900
C	7.69024200	9.24698500	0.12102100
H	6.83151900	9.67757000	-0.38324900
C	8.05572100	9.75858300	1.37443100
C	6.27402500	10.49441700	2.80341100
C	5.74108700	11.71974900	3.49877800
C	4.73243300	11.79093500	4.45431100
H	4.20648600	10.89359900	4.76077900
C	4.43685500	13.04771700	4.99774200
H	3.65709400	13.13739000	5.74730400
C	5.13183100	14.19496700	4.59104600
H	4.90778100	15.16998000	5.00967600
C	6.12841100	14.05640400	3.62805600
C	7.01331700	15.07125000	3.02614500
C	7.11040400	16.43716500	3.27996400
H	6.45975000	16.90523800	4.01057400
C	8.06478200	17.17883100	2.57102300
H	8.15496900	18.24400200	2.75804100
C	8.89989500	16.56884600	1.62696400
H	9.64132300	17.12825100	1.06755400
C	8.74991600	15.20174700	1.41777100
C	9.50158200	14.32538600	0.45046200
C	9.53508600	12.09443100	-0.44340000
C	10.66936800	11.31124800	-0.18498700
H	11.17623400	11.43484400	0.76616700
C	11.12935300	10.40178100	-1.13548900
H	12.00801100	9.79959300	-0.92513000
C	10.45103900	10.25711600	-2.34913300
H	10.80230200	9.54551000	-3.09055100
C	9.30187800	11.01316000	-2.61006000
H	8.76350600	10.88699900	-3.54259800
C	8.83632800	11.91924100	-1.66056200
N	7.30240400	10.78506900	1.96161900
N	6.37909200	12.82561800	3.13421900
N	7.84025400	14.52628100	2.10982000
N	9.09667300	13.02724400	0.50789100
O	5.80658900	9.36689000	3.02232400
O	10.37754400	14.78233900	-0.29830200
O	10.07443900	11.36391500	3.35041800
O	8.58855600	9.77417200	4.56959300
O	10.93220900	9.12359500	4.02100400
O	7.70509900	14.30714000	-1.94943700
O	6.35789200	12.48806900	-0.90248900
O	6.85696600	12.42207000	-3.34348600
S	9.73532500	9.92917200	3.61852400
S	7.32024400	12.85990500	-1.98853500
O	5.42819200	15.93070600	-0.14810100
H	6.11621300	15.32573400	-0.47286300

[(L5)Cu(OH)]$^{2-}$ (t) E= -2812.590105 H

Cu	7.74883800	12.62275500	1.87990400
C	9.03417300	9.12499700	1.74112800
C	9.63866000	8.15329000	0.93749600
H	10.63018200	7.79312800	1.18803700
C	8.96841000	7.65278600	-0.18100100
H	9.44798900	6.90683600	-0.80824900
C	7.68587900	8.11846700	-0.48660200
H	7.15649800	7.73493800	-1.35476000
C	7.08665200	9.09363800	0.31106200
H	6.10330500	9.48182700	0.06530300
C	7.75259600	9.62068700	1.42773400
C	6.19586300	10.47591500	3.02457200
C	5.72209300	11.75038000	3.69765100
C	4.72468200	11.85060200	4.66728100
H	4.20326100	10.96580500	5.01454200
C	4.42396100	13.12340300	5.16845300
H	3.65267600	13.23499300	5.92446700
C	5.10236200	14.25821400	4.70533300
H	4.86978000	15.24634300	5.08764600
C	6.08718600	14.08205000	3.73232300
C	6.92977900	15.11697200	3.07500300
C	6.91536000	16.49994700	3.27488300
H	6.23796600	16.94983700	3.99273000
C	7.79488800	17.28366600	2.52000900
H	7.80144800	18.36059000	2.65678200
C	8.66185800	16.69758800	1.58754200
H	9.34411500	17.29346600	0.99224700
C	8.62268300	15.30945900	1.44340100
C	9.45820900	14.48275700	0.48332400
C	9.76532900	12.27752400	-0.36951500
C	11.03334700	11.75070700	-0.08686000
H	11.55277300	12.10172100	0.79878900
C	11.60098600	10.77471400	-0.90674000
H	12.57957000	10.37063600	-0.66173700
C	10.90635700	10.30517700	-2.02564200
H	11.33629600	9.53294300	-2.65696400
C	9.64910700	10.83094300	-2.33209500
H	9.10604600	10.48010200	-3.20251800
C	9.08518900	11.81402400	-1.51373800
N	7.18794500	10.68825500	2.15484800
N	6.35382600	12.84499800	3.27856400
N	7.77947200	14.58856700	2.18016200
N	9.14829100	13.18318800	0.51713100
O	5.65739100	9.37771600	3.32570600
O	10.30734900	15.05361400	-0.24971300
O	10.11678700	11.16882600	3.10639400
O	8.99160200	9.34679100	4.38291200
O	11.18426500	8.92821400	3.27083300
O	7.71867900	14.00269600	-2.13629400
O	6.51362600	12.22993300	-0.86491300
O	7.09803900	11.87150100	-3.26030900
S	9.89272500	9.69222300	3.23222700
S	7.48208800	12.52069600	-1.96709000
O	6.64021300	15.95782300	-0.52481100
H	7.04161200	15.19615600	-1.03716300

[(L5)Cu] (d) E= -2736.422273 H

Cu	9.13762900	14.51843300	1.37391500
C	9.26918600	11.28250900	2.36518100
C	9.24401800	9.93493700	2.09560200
H	10.16694500	9.39807300	1.90736400
C	8.00791100	9.24874500	2.06508900
H	7.99721700	8.18876100	1.83206400
C	6.80498700	9.92711800	2.32233700
H	5.86229700	9.39187000	2.27662800
C	6.81445900	11.27998500	2.60024600
H	5.88684900	11.81935100	2.74407200
C	8.05131500	12.00790500	2.62721100
C	7.14785400	14.05580700	3.50645800

C	7.15094400	15.51356200	3.17994800
C	6.35563000	16.44562700	3.83690800
H	5.66841400	16.12599200	4.61166400
C	6.48225600	17.78697800	3.46539900
H	5.87860000	18.54611100	3.95210100
C	7.39130200	18.15651900	2.47457100
H	7.49405700	19.19827800	2.19663500
C	8.16037300	17.16380200	1.85077600
C	9.16603800	17.38989900	0.78641000
C	9.52828800	18.67400000	0.36812500
H	9.09219500	19.55028300	0.83162100
C	10.46550100	18.81371000	-0.64995300
H	10.76999300	19.80016700	-0.98452400
C	10.98320100	17.66975600	-1.25032100
H	11.68472100	17.73202200	-2.07339100
C	10.56803000	16.41485400	-0.79735600
C	11.05872200	15.20105800	-1.55382000
C	10.64564600	12.84138500	-1.39648500
C	12.01910600	12.42397400	-1.34470200
H	12.79622900	13.17827000	-1.36007900
C	12.34508000	11.08263000	-1.28914000
H	13.38767400	10.78100400	-1.26364100
C	11.33359300	10.10840200	-1.24100900
H	11.59280000	9.05599900	-1.17999100
C	9.97588700	10.48657700	-1.23567900
H	9.20449500	9.73002100	-1.14971100
C	9.63053100	11.81990500	-1.31438500
N	8.12013000	13.34084200	2.81081600
N	8.00241400	15.88277700	2.21159900
N	9.70312700	16.27577000	0.22872200
N	10.24419200	14.11102900	-1.46537900
O	6.42464400	13.58037800	4.38031400
O	12.03260900	15.30168300	-2.31323700
O	10.75911000	13.27299300	1.49092500
O	10.95922000	12.62110500	3.89805300
O	11.90449800	11.16589500	2.09645300
O	7.75180700	13.22250200	-0.06525600
O	7.13247300	11.01703700	-1.01723600
O	7.55909100	12.94097400	-2.54229700
S	10.85649700	12.13355200	2.49656800
S	7.88261100	12.28998100	-1.23508700

[(L5)Cu] (q) E= -2736.633847 H

Cu	9.13908200	14.51404500	1.37495800
C	9.23637800	11.27575200	2.33849100
C	9.20648700	9.92857200	2.06512800
H	10.12487200	9.39718900	1.84221200
C	7.97609600	9.23335200	2.08891800
H	7.96030300	8.17540300	1.84742400
C	6.78666900	9.89955000	2.42593200
H	5.84669800	9.35743300	2.43253300
C	6.80019200	11.25044100	2.71645900
H	5.87619700	11.77946000	2.91039300
C	8.02632200	11.99497000	2.65162200
C	7.13675200	14.06380200	3.50256000
C	7.14147100	15.52036000	3.16573400
C	6.34360300	16.45331600	3.81912800
H	5.65552900	16.13511900	4.59366400
C	6.47033900	17.79416900	3.44599500
H	5.86285300	18.55330500	3.92789500
C	7.38427100	18.16275200	2.45950600
H	7.48430400	19.20343200	2.17705200
C	8.15898000	17.16939700	1.84313800
C	9.17058000	17.39605500	0.78374100
C	9.54514500	18.68112400	0.37852100
H	9.11464600	19.55717100	0.84793700
C	10.48957700	18.82205600	-0.63272600
H	10.80522300	19.80882800	-0.95586400
C	11.00115200	17.67921000	-1.24031200
H	11.70939400	17.74287900	-2.05749300
C	10.57313600	16.42390000	-0.80032400
C	11.06166100	15.21252900	-1.56184000
C	10.66038100	12.85140100	-1.40858200
C	12.03645600	12.44255300	-1.35651400
H	12.80881000	13.20181900	-1.36593600
C	12.37140000	11.10305300	-1.30797300
H	13.41596500	10.80830200	-1.28202100
C	11.36636700	10.12195300	-1.26710700
H	11.63237600	9.07094100	-1.21159800
C	10.00633700	10.49128200	-1.26157700
H	9.23980100	9.72922300	-1.18092400
C	9.65209800	11.82280500	-1.33300500
N	8.07723000	13.33629600	2.77103900
N	7.99888300	15.88874700	2.20220100
N	9.70135300	16.28271800	0.21950700
N	10.25142000	14.11920700	-1.47284100
O	6.43192700	13.60035100	4.39613100
O	12.03162400	15.31670600	-2.32607500
O	10.75164100	13.26917600	1.48395600
O	10.93197600	12.58411300	3.88036800
O	11.87044700	11.14126300	2.06408600
O	7.76179300	13.21358300	-0.08841600
O	7.15990400	11.00141000	-1.03586600
O	7.57490100	12.92496000	-2.56563400
S	10.83062500	12.11325500	2.47296100
S	7.90076800	12.28010900	-1.25546300

[(L7)Cu] (dos) E= -3043.74310150 H

Cu	11.15256800	11.17616200	17.33940200
N	9.33021100	10.80249600	16.29087900
N	9.96955300	10.49936500	18.80116500
N	12.41283900	11.13208900	18.89538700
N	12.96852100	11.88198500	16.44857200
O	7.17774300	10.01714900	16.71113400
O	15.17612300	12.30597700	17.05938400
C	8.30273100	10.35945300	17.09542000
C	8.70028700	10.19340600	18.53229600
C	7.84469100	9.74027700	19.53444800
C	8.36064700	9.61445900	20.82762300
C	9.69787600	9.92798000	21.08891500
C	10.49517600	10.37737100	20.03414000
C	11.92838900	10.74201100	20.08815900
C	12.76764200	10.71101400	21.20363000
C	14.10409200	11.08949100	21.04313300
C	14.58020100	11.49124400	19.79166100
C	13.68233200	11.49943500	18.72539600
C	14.03438300	11.91058400	17.32755300
H	14.77359800	11.07143600	21.89718800
H	15.61089100	11.79067200	19.64118900
H	12.39024600	10.40036400	22.17139000
H	10.10934500	9.82143400	22.08629300
H	7.72317700	9.26623000	21.63399800
H	6.81333600	9.49944900	19.30389000
C	9.13344400	11.12361200	14.98576600
C	8.05891000	11.96700100	14.50085200
C	10.06909000	10.59916200	14.05294100
C	7.28438100	12.74170700	15.38174900
C	7.85238800	12.09494800	13.08639100
C	9.85758900	10.71309900	12.69014800
H	10.91991200	10.04569200	14.43646200
C	6.28980100	13.58981000	14.90241800
H	7.48363100	12.70864400	16.44669500
C	6.81967000	12.94951600	12.62831300
C	8.74693500	11.41167100	12.20117600
H	10.54768100	10.24772400	11.99659600
C	6.04939000	13.68108600	13.52450300
H	5.70763800	14.18475400	15.59944900
H	6.63827900	13.05873500	11.56710900
H	5.27181800	14.33917500	13.14887300
S	8.49210200	11.43048600	10.39562800
O	7.09657400	10.92307800	10.21208200
O	8.65919100	12.85740000	9.98275500

O	9.52975400	10.52275700	9.83004500
C	13.20701600	12.01986300	15.11512100
C	12.29383000	12.77527200	14.28843500
C	14.31707600	11.37346200	14.49744300
C	11.22854900	13.49806000	14.86015600
C	12.49285000	12.82551400	12.86774700
C	14.49313000	11.41326600	13.12466000
H	14.98957800	10.78035200	15.10437300
C	10.35411700	14.23719400	14.06539600
H	11.08382600	13.47270300	15.93496300
C	11.57542400	13.56756100	12.08508800
C	13.60566000	12.12179100	12.30861100
H	15.31923200	10.87369600	12.67659800
C	10.52378100	14.26119800	12.67712900
H	9.52888500	14.77046200	14.52595500
H	11.68531500	13.60268600	11.00944600
H	9.82764800	14.81045400	12.05187000
S	13.90915600	12.10066300	10.51357700
O	12.68366300	11.47651000	9.92913200
O	14.08177100	13.53551900	10.12755300
O	15.14236300	11.28426000	10.31490500

[(L8)Cu] (q) E= -3043.74912970 H

Cu	11.23093400	11.01393500	17.27515800
N	9.62035300	10.23780000	16.15356800
N	10.18197200	10.07302200	18.67932700
N	12.17730300	11.59533700	18.92011000
N	12.86086000	12.03484500	16.45370100
O	7.88423000	8.68795500	16.39077500
O	14.57573200	13.47381100	17.14868000
C	8.83256300	9.33585200	16.84662100
C	9.16216300	9.29404100	18.31618900
C	8.45356400	8.56463300	19.27004000
C	8.84012000	8.69124300	20.60874800
C	9.90193000	9.52844600	20.96827700
C	10.57321100	10.21946100	19.95767800
C	11.72624000	11.14345400	20.10283400
C	12.35245300	11.55566700	21.28133500
C	13.43436500	12.43822900	21.18407800
C	13.88423700	12.88281700	19.93592100
C	13.21756100	12.42047900	18.80199200
C	13.61460100	12.73496800	17.38235300
H	13.93340500	12.77429500	22.08743700
H	14.72870900	13.55570500	19.84074300
H	12.00597600	11.19985400	22.24534800
H	10.20155700	9.63672200	22.00502500
H	8.30884500	8.14067900	21.37853900
H	7.62611000	7.93042900	18.97341200
C	9.44827900	10.50080900	14.83097900
C	9.03124300	9.52848800	13.87045400
C	9.78515800	11.81982100	14.38159500
C	8.94453000	9.86216100	12.50929600
H	8.84772800	8.50863700	14.18451500
C	9.64876700	12.16646500	13.06711200
H	10.11967600	12.53904400	15.12183700
C	8.58557500	8.88739800	11.53498700
C	9.23247400	11.20386200	12.08580500
H	9.86822100	13.17900900	12.74081700
C	8.48150000	9.23281700	10.19778200
H	8.38807300	7.86878600	11.85594800
C	9.12361000	11.53451400	10.73119300
C	8.73863700	10.55869000	9.80624500
H	8.20517700	8.49008600	9.45760400
H	9.34247600	12.54599700	10.40379100
C	13.10362300	12.06844800	15.11443100
C	12.66227400	10.91897500	14.38553300
C	13.72420600	13.13745800	14.38948200
C	12.80277100	10.81897800	12.99578200
H	12.22552400	10.09633100	14.94225500
C	13.83817100	13.05921300	13.02511100
H	14.05539000	14.02149900	14.91579800
C	12.32529900	9.68540000	12.28324500
C	13.38762000	11.91392200	12.28141500
H	14.26762200	13.89070800	12.47263300
C	12.42296900	9.63426400	10.90177400
H	11.86627800	8.86983700	12.83349900
C	13.48602800	11.84179300	10.88722200
C	13.00969900	10.71024000	10.21552900
H	12.04289100	8.77917700	10.35429800
H	13.92560800	12.66589900	10.33364400
S	8.46062600	11.04375300	8.08407300
S	13.24408400	10.60134300	8.42470500
O	7.01951500	11.46068800	8.04682100
O	8.73411700	9.82571900	7.26279700
O	9.40057100	12.17432600	7.81732900
O	12.22656800	9.62352200	7.93570700
O	13.04417600	11.98873800	7.90243200
O	14.65400500	10.11492000	8.25794200

References

[1] G. Bozoklu, C. Marchal, C. Gateau,; J. Pécaut ; D. Imbert, M. Mazzanti, *Chem. - A Eur. J.* **2010**, *16*, 6159–6163.
[2] A. Courtin, H.-R. von Tobel, *Helv. Chim. Acta* **1977**, *60*, 1994–1999.
[3] R. Matheu, S. Neudeck, F. Meyer, X. Sala, A. Llobet, *ChemSusChem* **2016**, *9*, 3361–3369.
[4] C. Costentin, S. Drouet, M. Robert, J. M. Savéant, *J. Am. Chem. Soc.* **2012**, *134*, 11235–11242.
[5] J. Shao, Y. H. Qiao, H. Lin, H. K. Lin, *J. Lumin.* **2008**, *128*, 1985–1988.
[6] P. Garrido-Barros, I. Funes-Ardoiz, S. Drouet, J. Benet-Buchholz, F. Maseras, A. Llobet, *J. Am. Chem. Soc.* **2015**, *137*, 6758–6761.
[7] Data Collection with APEX II Version V2013.4-1. Bruker (2007). Bruker AXS Inc., Madison, Wisconsin, USA.
[8] Data Reduction with Bruker SAINT Version V8.30c. Bruker (2007). Bruker AXS Inc., Madison, Wisconsin, USA.
[9] R. H. Blessing, *Acta Crystallogr. Sect. A* **1995**, *51*, 33–38.
[10] G. M. Sheldrick, *Acta Crystallogr. Sect. A* **2015**, *71*, 3–8.
[11] C. B. Hübschle, G. M. Sheldrick, B. Dittrich, *J. Appl. Crystallogr.* **2011**, *44*, 1281–1284.
[12] G. M. Sheldrick, *Acta Crystallogr. Sect. C* **2015**, *71*, 3–8.
[13] Gaussian 09, Revision A.02, M. J. Frisch, G. W. Trucks, H. B. Schlegel, G. E. Scuseria, M. A. Robb, J. R. Cheeseman, G. Scalmani, V. Barone, G. A. Petersson, H. Nakatsuji, X. Li, M. Caricato, A. Marenich, J. Bloino, B. G. Janesko, R. Gomperts, B. Mennucci, H. P. Hratchian, J. V. Ortiz, A. F. Izmaylov, J. L. Sonnenberg, D. Williams-Young, F. Ding, F. Lipparini, F. Egidi, J. Goings, B. Peng, A. Petrone, T. Henderson, D. Ranasinghe, V. G. Zakrzewski, J. Gao, N. Rega, G. Zheng, W. Liang, M. Hada, M. Ehara, K. Toyota, R. Fukuda, J. Hasegawa, M. Ishida, T. Nakajima, Y. Honda, O. Kitao, H. Nakai, T. Vreven, K. Throssell, J. A. Montgomery, Jr., J. E. Peralta, F. Ogliaro, M. Bearpark, J. J. Heyd, E. Brothers, K. N. Kudin, V. N. Staroverov, T. Keith, R. Kobayashi, J. Normand, K. Raghavachari, A. Rendell, J. C. Burant, S. S. Iyengar, J. Tomasi, M. Cossi, J. M. Millam, M. Klene, C. Adamo, R. Cammi, J. W. Ochterski, R. L. Martin, K. Morokuma, O. Farkas, J. B. Foresman, and D. J. Fox, Gaussian, Inc., Wallingford CT, 2016.
[14] A. D. Becke, *J. Chem. Phys.* **1993**, *98*, 5648‐5652.
[15] S. Grimme, J. Antony, S. Ehrlich, H. Krieg, *J. Chem. Phys.* **2010**, *132*, 154104.
[16] P. C. Hariharan, J. A. Pople, *Theor. Chim. Acta* **1973**, *28*, 213–222.
[17] W. J. Hehre, K. Ditchfield, J. A. Pople, *J. Chem. Phys.* **1972**, *56*, 2257.
[18] M. M. Francl,; W. J. Pietro, W. J. Hehre, J. S. Binkley, M. S. Gordon, D. J. DeFrees, J. A. Pople, *J. Chem. Phys.* **1982**, *77*, 3654–3665.
[19] P. J. Hay, W. R. Wadt, *J. Chem. Phys.* **1985**, *82*, 270.
[20] W. R. Wadt, P. J. Hay, *J. Chem. Phys.* **1985**, *82*, 284.
[21] P. J. Hay, W. R. Wadt, *J. Chem. Phys.* **1985**, *82*, 299.
[22] D. Feller, *J. Comput. Chem.* **1996**, *17*, 1571–1586.
[23] K. L. Schuchardt, B. T. Didier, T. Elsethagen, L. Sun, V. Gurumoorthi, J. Chase, J. Li, T. L. Windus, *J. Chem. Inf. Model.* **2007**, *47*, 1045–1052.
[24] A. V.Marenich, C. J. Cramer, D. G. Truhlar, *J. Phys. Chem. B* **2009**, *113*, 6378–6396.
[25] A. Lewis, J. A. Bumpus, D. G. Truhlar, C. J. Cramer, *J. Chem. Educ.* **2004**, *81*, 596–604.
[26] A. V. Marenich, A. Majumdar, M. Lenz, C. J. Cramer, D. G. Truhlar, *Angew. Chemie Int. Ed.* **2012**, *51*, 12810–12814.
[27] S. G. Winikoff, C. J. Cramer, *Catal. Sci. Technol.* **2014**, *4*, 2484–2489.
[28] I. Funes-Ardoiz, P. Garrido-Barros, A. Llobet, F. Maseras, *ACS Catal.* **2017**, *7*, 1712–1719.
[29] A. A. Voityuk, S. F. Vyboishchikov *Phys. Chem. Chem. Phys.* **2020**, *22*, 14591-14598.
